# Range of hurdles and opportunities for developing PPPs in diagnostics: a contextual analysis, Ethiopia

**DOI:** 10.1186/s12913-026-14354-z

**Published:** 2026-03-28

**Authors:** Getnet Mitike Kassie, Rewina Tilahun Gessese, Tsegereda Abebe Andargie, Mohammed Abseno, Mazengia Ayale Mekonnen, Jennifer Applegate

**Affiliations:** 1 International Institute for Primary Health Care- Ethiopia, Addis Ababa, Ethiopia; 2 Ministry of Health, Addis Ababa, Ethiopia; 3 Johns Hopkins Bloomberg School of Public Health, Baltimore, MD USA

**Keywords:** Public-private partnership, Healthcare, Diagnostics services, Ethiopia, Policy

## Abstract

**Background:**

Public-Private Partnerships (PPPs) have emerged as a strategic approach to enhancing healthcare service delivery, particularly in resource-limited settings. In the diagnostics sector, PPPs can help address challenges such as inadequate infrastructure, workforce shortages, and supply chain inefficiencies. In Ethiopia, while private sector engagement in healthcare has long been recognized, the integration of PPPs in the Ethiopian diagnostics sector remains at an early stage, necessitating an exploration of both enabling conditions and barriers.

**Objective:**

This study aims to examine the challenges and opportunities influencing the development of PPPs in Ethiopia’s diagnostics services.

**Methods:**

A qualitative contextual analysis was conducted using 16 key informant interviews with government representatives, association and private sector representatives, alongside a review of major policy documents and regulatory frameworks. Thematic analysis was applied to identify key themes and sub-themes, capturing insights on opportunities and challenges of PPP in diagnostics. Atlas.ti software was used for the coding process and organized the data efficiently for analysis.

**Results:**

The study revealed three primary themes and eleven sub-themes. First, Ethiopia’s diagnostic service environment is marked by significant growth through centralization, international collaborations, and automation, yet it continues to face profound challenges, including supply chain inefficiencies, critical shortages of reagents and skilled personnel, inadequate preventive maintenance, and geographic inequities in access. Second, opportunities for PPPs exist through a combination of an established legal and policy framework that supports structured partnerships, operational flexibility, a centralized governance model aimed at ensuring alignment with national priorities, and the potential for capacity building and knowledge transfer. Partnerships with experienced private and international actors were found to facilitate professional development of local staff, exposure to international best practices, and gradual implementation of modern technologies and services, thereby strengthening local expertise over time. Third, major challenges impede PPP implementation, including regulatory rigidity and bureaucratic delays, deep-seated mistrust between sectors, insufficient stakeholder representation in policy-making, financial constraints deterring local investment, and concerns over weak monitoring and evaluation mechanisms. Importantly, the study identified that informal, trust-based collaborations between public and private entities already exist and function pragmatically, suggesting a foundational willingness to collaborate that has not yet been systematized into formal PPPs.

**Conclusion and Recommendation:**

While PPPs hold significant potential for strengthening Ethiopia’s diagnostics sector, addressing regulatory complexities, collaboration, and developing sustainable financing models are essential for long-term success. Strengthening governance structures, improving transparency in policy implementation, and fostering trust between public and private entities will be critical in optimizing PPPs to enhance diagnostics service delivery.

**Supplementary Information:**

The online version contains supplementary material available at 10.1186/s12913-026-14354-z.

## Introduction

Diagnostic services, encompassing laboratory testing and medical imaging, are core components of effective health systems. They facilitate accurate disease detection, inform clinical decision-making, support treatment monitoring, and ultimately improve patient outcomes [1, 2]. Ensuring access to high-quality diagnostic services is critical for advancing universal health coverage (UHC) and enhancing the resilience of health systems [3, 4]. In Ethiopia, however, access to advanced diagnostic services remains severely constrained. Public health facilities frequently face shortages of essential equipment, reagents, and skilled personnel, resulting in service interruptions. Poor maintenance of technologies such as MRI, CT scan, and X-ray machines further limits functionality, while significant geographic disparities compel rural populations to travel long distances, incurring substantial financial and time costs [5–7]. These systemic gaps delay diagnosis, hinder timely treatment, and compromise quality of care, underscoring the need for innovative strategies to strengthen the diagnostic sector [8, 9].

Traditional public financing mechanisms have proven insufficient to address these systemic challenges. In response, PPPs have increasingly been adopted globally as a strategy to mobilize private sector investment, technology, and managerial expertise to complement public service delivery [10]. PPPs are generally defined as structured, long-term collaborations between government entities and private organizations for the provision of public infrastructure or services, characterized by shared risks, performance-based contracts, and clearly defined accountability mechanisms [11].

Historically, PPPs have been widely applied in large-scale infrastructure sectors such as transportation and energy. More recently, their use has expanded into health systems, including hospital construction, service outsourcing, and specialized clinical services. Experiences from countries such as the United Kingdom, India, and South Africa demonstrate that PPPs can contribute to improved infrastructure development and operational performance when supported by strong governance structures [12–14]. However, empirical evidence remains mixed. For example, evaluations of Private Finance Initiative projects in the United Kingdom have raised concerns regarding long-term fiscal burdens and value for money [15]. Similarly, assessments by the European Court of Auditors have documented cost overruns and limited efficiency gains in some European PPP projects [16].

The effectiveness of PPPs is shaped not only by resource availability but also by the maturity and socio-institutional legitimacy of the PPP ecosystem. Market maturity involves clear regulatory structures, strong institutional capacity, and standardized contracting mechanisms that reduce uncertainty and facilitate equitable risk allocation [17, 18]. Legitimacy refers to stakeholder trust, public acceptance, and alignment with broader policy objectives factors that are critical for sustaining partnerships and ensuring accountability [19]. In low- and middle-income countries (LMICs), where institutional capacity may be evolving, deficiencies in trust, regulatory clarity, and monitoring systems can constrain PPP performance. Understanding these institutional dimensions is therefore critical when assessing the feasibility of PPPs in new sectors [20–22].

In Ethiopia, private sector participation in healthcare was formally recognized in the 1993 National Health Policy, which endorsed a mixed health system in which private actors complement public provision [23]. Subsequent national strategies, including the Health Sector Transformation Plan (HSTP II), further emphasized structured private sector engagement in service delivery [24]. However, the formal institutionalization of PPPs as a dedicated service delivery mechanism is relatively recent. Ethiopia enacted its PPP Proclamation and supporting legal frameworks in 2017, establishing the policy and regulatory foundation for structured partnerships [25–27]. Since then, PPPs have primarily been implemented in sectors such as road construction and energy, while application within the health remains at an early stage [28, 29].

Diagnostic services represent a particularly strategic area for PPP engagement in Ethiopia. By mobilizing private investment, advanced technologies, and managerial capacity, PPPs have the potential to expand service availability, reduce equipment downtime, and improve operational efficiency. Experiences from other settings suggest that carefully structured PPP models can enhance access to specialized diagnostics while supporting long-term financial and operational sustainability [30, 31].

This study forms part of the African Health Diagnostics Platform (AHDP), a multi-country initiative led by the Bill & Melinda Gates Foundation, the European Investment Bank, and the Clinton Health Access Initiative, aimed at strengthening laboratory services across sub-Saharan Africa through PPP models. As part of this initiative, a multi-country evaluation was conducted in Ethiopia, Ghana, and Kenya. The present manuscript focuses on the Ethiopian case and reports findings from the first, pre-bid phase of data collection. This phase provides a baseline assessment of the diagnostic services landscape, existing private sector engagement, and emerging opportunities and challenges for PPPs implementation.

Building on this context, the study addresses the following research question:

What is the current context of public–private partnerships in Ethiopia’s diagnostic sector, including the diagnostic services environment, opportunities for PPPs engagement, and challenges to implementation?

## Methods

### Study design

This study employed a qualitative contextual analysis to explore the challenges and opportunities influencing the development of PPPs in Ethiopia’s diagnostic services. The analysis was based on data from the first-phase Key Informant Interviews (KIIs) of the AHDP project. While the AHDP KII guide was not based on a formal published framework, it was informed by existing research and literature on PPPs and health service delivery, providing a structured approach to guide interviews and support systematic analysis.

Although the AHDP KIIs captured detailed aspects of PPP design, tendering, and financing, this manuscript focuses specifically on contextual and system-level insights relevant to PPP feasibility. The analysis draws on stakeholders’ experiences with the diagnostic service environment, existing policy frameworks, historical public-private relationships, and preparatory conditions. Detailed evaluation of project-specific PPP design or implementation will be addressed in a subsequent case study of the Integrated Diagnostic Center (IDC) PPP.

By concentrating on contextual and system-level factors, this study highlights the enabling conditions and barriers for PPP development in Ethiopia’s diagnostics sector, providing a foundation for future implementation-focused research.

### Study setting

This study was conducted in Addis Ababa, the capital city of the Federal Democratic Republic of Ethiopia, which serves as the country’s political, economic, and healthcare hub. According to projections from the Central Statistical Agency (CSA), Addis Ababa had an estimated population of 4.8 million in 2020. The city provides healthcare services to both its residents and a large number of patients from across Ethiopia who seek specialized care. Healthcare services in Addis Ababa are primarily delivered by the public sector, private providers, and not-for-profit non-governmental organizations (NGOs). The public healthcare system consists of 12 public hospitals, which offer advanced tertiary care, including diagnostics, pathology, and radiology services. Additionally, 98 health centers provide primary healthcare and basic diagnostic services to the population. Alongside public healthcare facilities, Addis Ababa has a high concentration of private healthcare providers, playing a crucial role in diagnostics and specialized medical services. Excluding pharmacies, in 2025, the Private sector includes 12 diagnostic centers with branches, 25 hospitals, and 759 clinics.

### Participants and sampling

The study included key stakeholders engaged in PPPs within Ethiopia’s healthcare sector, with a specific focus on diagnostic services. A purposive sampling strategy was employed to ensure the inclusion of participants with direct and decision-relevant experience across the PPP ecosystem. Selection was guided by the study objective of examining the institutional, regulatory, and operational conditions shaping PPP feasibility, and therefore targeted individuals involved in policy formulation, regulation, financing, service provision, and PPP implementation.

Initial participants were identified based on their formal roles and active engagement in PPP-related functions within key organisations, including the Ministry of Health, Ministry of Finance, Addis Ababa Health Bureau, Ethiopian Investment Commission, Ethiopian National Accreditation Office, as well as technical agencies such as the Ethiopian Public Health Institute and Ethiopian Pharmaceutical Supply Services. To ensure representation of implementation-level realities, participants were also drawn from the private sector, including diagnostic service providers (Arsho Medical Diagnostics, International Clinical Laboratories, Pioneer Diagnostics Center), major public hospitals (Yekatit 12 Hospital, St. Paul’s Millennium Medical College, St. Peter’s Specialized Hospital), and professional associations such as the Ethiopian Medical Diagnostics Association. To further capture networked and less visible actors within the PPP landscape, snowball sampling was applied, whereby initial participants identified additional individuals with relevant expertise or operational influence.

Data saturation was assessed through an iterative and concurrent process of data collection and analysis. After 14 interviews, no substantively new codes or themes were identified, with recurring patterns observed across key domains, including the diagnostic service environment, regulatory and institutional arrangements, and stakeholder interactions. Two additional interviews were conducted to confirm thematic redundancy, resulting in a final sample of 16 key informants. Saturation was therefore determined based on the point at which additional data no longer contributed new conceptual insights to the analysis.

### Data collection

Data were collected between November 2, 2021, and February 28, 2022, through key informant interviews (KIIs) and a review of relevant documents to explore the background, evolution, key stakeholders, policy environment, and related challenges of (PPPs) in the provision of diagnostic services in Ethiopia. Additionally, policy documents and regulatory frameworks were reviewed to provide supplementary contextual insights (Supplementary material 1–4).

Before data collection, data collectors received training on the study objectives, ethical considerations, interview protocols, and qualitative research techniques to ensure consistency and methodological rigor. The interview guide was developed specifically for this multi-country study, including Ethiopia, with an English-language version provided as a supplementary file. For the Ethiopian context, the guide was contextualized and adapted into Amharic to ensure relevance and comprehensibility for different types of respondents (Supplementary material 5 and 6).

The study team was actively involved in all aspects of the data collection process, including the development and pretesting of tools, supervision of data collectors, coordination of fieldwork, and ensuring adherence to the study protocol and ethical standards. This manuscript focuses on the first set of interviews, which explored the broader context and background of PPPs in Ethiopia’s diagnostic services. Findings from subsequent interviews, which examined process evaluation and implementation experiences, will be reported separately in future manuscripts.

### Data analysis

The transcribed interviews were coded using ATLAS.ti version 22 [32]. Initial coding was carried out based on predefined thematic categories aligned with the interview guide, alongside inductive concepts emerging from the data. These codes were then grouped into sub-themes and further organized into overarching themes through iterative team discussions. Beyond the coding process, the study team held analysis meetings to review and refine the thematic structure, ensure consistency in interpretation, and align emerging findings with the study objectives.

## Results

### Socio-demographic characteristics of the participant

A total of 16 participants were included in the study, representing various stakeholders such as government and private organizations, and associations, the Ministry of Health agencies, and public hospitals. The group consisted predominantly of male respondents, with female participants making up approximately one-third of the total (Table 1).

**Table 1:** Socio-demographic characteristics of participants

Respondent ID	Sex	Organization	Role / Position
R1	Female	Ministry of Health	Director, Partnership and Cooperation Directorate
R2	Male	Ministry of Finance	Partnership and Corporate Directorate
R3	Male	International Clinical Laboratory (ICL)	Director
R4	Male	Ethiopian Medical Laboratory Association (EMLA)	President
R5	Male	St. Paul Hospital Millennium Medical College (SPHMMC)	Head of Pathology Department
R6	Male	SPHMMC	Head of Radiology Department
R7	Female	Ethiopian Pharmaceutical Supply Services (EPSS)	Supply Chain Advisor
R8	Female	SPHMMC	Vice Provost
R9	Female	Pioneer Diagnostic Center	Marketing Manager
R10	Male	Ethiopian Public Health Institute	Associate Researcher, HIV/Infant Testing Focal Person
R11	Female	Arsho Laboratories	Managing Director
R12	Female	Ethiopian Medical Association	Executive Director
R13	Male	Addis Ababa Health Bureau (AAHB)	Deputy Lead
R14	Male	Yekatit 12 Hospital Medical College	Medical Services Quality Director
R15	Male	Ethiopian Investment Office (EIC)	Director, Registration and Licensing
R16	Male	Ethiopian National Accreditation Office (ENAO)	Deputy Director, Accreditation Services

### Themes and subthemes

The findings of this study are organized into three primary themes and eleven sub-themes. The themes are: (1) The Diagnostic Services Environment in Ethiopia, (2) PPPs in Diagnostics Services, and (3) Challenges in Implementing PPPs in Diagnostics.

### Theme 1: Diagnostic service landscape and system readiness for PPPs

This theme examines the evolution, current constraints, and structural characteristics of diagnostic services in Ethiopia. The findings indicate that while there has been measurable progress in expanding capacity, the system remains constrained by institutional, operational, and financial limitations that directly shape the feasibility of PPPs.

### Sub-theme 1: Evolution and recent reforms in diagnostic services

The findings illustrate a significant evolutionary shift in Ethiopia’s diagnostic landscape, transitioning from a historical reliance on basic, centralized testing toward a more sophisticated, technologically integrated ecosystem. Respondents noted that the sector’s origins were anchored in the Ethiopian Public Health Institute (EPHI), where diagnostic capacity was primarily restricted to essential disease identification. This historical phase was characterized by a lack of advanced instrumentation, which severely constrained the system’s ability to manage complex clinical conditions:

“Historically, as a country, what we know is EPHI… lots of tests were done in Pasteur [EPHI]” (13).

Participants detailed a gradual expansion of services, driven by institutional scaling and international academic partnerships. A primary example of this centralization-to-outreach model is the St. Paul’s Millennium Medical College, which now serves as a diagnostic hub for approximately 45 health organisations. Collaborations with global entities, such as Michigan and Zurich Universities, have introduced digital pathology and slide-scanning capabilities, facilitating virtual consultations and reducing diagnostic turnaround times.

A critical operational mechanism identified was the adoption of placement agreements with multinational suppliers. These strategic procurement models have allowed public hospitals to bypass high upfront capital expenditures, securing high-end diagnostic machinery while ensuring continuous service availability. This shift toward reagent-based placement models serves as a foundational precursor for more complex PPP arrangements.

Furthermore, automation and digitization have emerged as core drivers of diagnostic efficiency. Several organisations have successfully integrated automated systems across the patient continuum, including triage, medical records, and outpatient services. One respondent highlighted the impact of this phased digital transition over a three-year period:

“Currently, our hospital is fully automated… We took small steps… reception first, then medical records, then outpatient services. Now all services in the hospital are automated” (R14).

Despite these advancements, the findings suggest a readiness gap in the broader modernization effort. While digitized reporting and synoptic workflows are being introduced, participants indicated that approximately 80% of planned pathology improvements remain unfulfilled. Highly specialized services, such as immunohistochemistry and molecular cytogenetics, remain largely inaccessible, highlighting a discrepancy between sectoral ambition and current operational reality.

### Sub-theme 2: Existing service gaps and barriers in diagnostics services

Despite efforts to expand capacity, the sector faces persistent barriers in supply chain management, maintenance, and staffing. The diagnostic supply chain remains underdeveloped, with a lack of dedicated local suppliers for high-quality reagents and chemicals. Furthermore, a significant salary disparity where public sector professionals may earn five times less than their private-sector counterparts leads to high attrition.

A critical constraint identified was the maintenance-decoupling mechanism, where the public sector successfully procures high-end technology but lacks the technical ecosystem to sustain it. Many devices, including MRI and X-ray machines, remain nonfunctional for extended periods due to a lack of maintenance contracts and spare parts. As one respondent emphasized:

“Preventive maintenance… is a major issue… Machines often remain dysfunctional for long periods due to lack of spare parts and technical expertise” (R6).

From a PPP perspective, these gaps influence feasibility by creating a strong entry point. Such models could shift technical and operational risks to private partners, directly addressing the public sector’s current inability to manage equipment lifecycles.

### Sub-theme 3: Informal collaborations

In the absence of a formalized national PPP framework, Ethiopia’s diagnostic sector relies on ad-hoc collaborations to navigate public-sector deficiencies. This institutional bypass mechanism depends on personal social networks and informal arrangements to maintain service continuity. For instance, private providers such as Wudase Diagnostic Center often absorb costs for indigent patients by waiving fees and providing complimentary ambulance transport for high-cost imaging:

“When a patient can’t afford a CT scan… doctors contact Wudase… They sometimes waive half or even the full fee and even send an ambulance to pick up the patient” (R14).

This integration demonstrates the feasibility of coordinated referral networks, as facilities like Kadisco and St. Luke utilize private transport to move samples to public labs at highly subsidized rates often 250 ETB compared to market rates of 2000 ETB:

“Instead of carrying the sick patients on a bus… they bring them in their ambulances once a week… what we spend for the supplies… is very little” (R5).

At a systemic level, this collaboration manifests as mutual resource sharing, where public and private entities (including EPHI) exchange reagents to mitigate chronic stockouts. This reflects strong pragmatic coordination and indicates that the private sector possesses a latent readiness for formal partnerships…

“Whenever they [EPHI] encounter a shortage of commodities, they ask and we give them… Although it is informal, it is a well-established fact.” (R11).

The scalability of this partnership was evidenced during the COVID-19 pandemic, where the private sector’s existing molecular infrastructure allowed for rapid expansion:

“Because we already had the infrastructure and trained personnel, we quickly scaled up… we trained 20 new staff and acquired 14 PCR machines in just a few weeks” (R3).

While these non-contractual arrangements ensure service continuity, their reliance on individual relationships rather than institutional policy makes them fragile. These findings suggest that while the operational foundation exists, the lack of a legal framework prevents these informal successes from becoming a standardized national diagnostic strategy.”

### Sub-theme 4: Financing structure

Diagnostic services are primarily financed within the broader health system budget rather than through dedicated allocations, leading to budgetary invisibility. Public hospitals operate under general regional and federal budgets, which limits the ability to prioritize diagnostic-specific resources. While the government ensures access for vulnerable populations through subsidies and Community-Based Health Insurance (CBHI), the lack of a standalone budget line obscures the true “cost-per-test.”

Respondents noted that patients with certificates of indigency:

“have the right to free medical treatment… as long as they maintain the referral system” (R1).

While this ensures social equity, the current financing structure hinders PPP feasibility. Without transparent unit-cost data, it is difficult for the government to calculate “Value for Money” (VfM) or negotiate fair, performance-based payment models with private investors. This financial opacity remains a primary barrier to establishing the predictable revenue streams necessary for long-term private sector engagement.

### Theme 2: Opportunities for PPPs in diagnostics services

This theme identifies the structural and legal foundations within the Ethiopian system that facilitate private sector engagement. The findings suggest that Ethiopia’s shift from ad-hoc projects to a centralized, programmatic framework is fostering institutional legitimacy and building a foundation for PPP market maturity.

### Sub-theme 1: Existence of a legal framework for PPPs

The transition from informal collaborations to a programmatic legal architecture serves as the primary driver of institutional legitimacy in Ethiopia’s diagnostic sector. The government has adopted a structured PPP Framework to ensure consistency across sectors, defining policies and foundations that ensure transparency and efficiency [26]. This architecture is anchored in the PPP Policy (2017) designed to mobilize private investment and foster innovation and is reinforced by Proclamation No. 1076/2018 [33].Unlike previous strategic directions, the Proclamation mandates how projects are identified, approved, and procured, formalizing competitive bidding, contract structuring, and dispute resolution.

This formalization mechanism is further refined by specific directives from the Ministry of Finance and Economic Cooperation [25] and the PPP Guidelines [27, 34]. Which emphasize value-for-money analysis. Governance is managed through a PPP Board and a PPP Unit, which require every diagnostic project to demonstrate four critical benchmarks: financial benefit (Value for Money), financial sustainability **(**Affordability**)**, alignment with national priorities (Public Interest), and long-term viability (Sustainability).

This evolution exists because the government recognized that closing diagnostic gaps required a shift toward structured risk-sharing. For diagnostic services, this framework creates substantial feasibility by reducing the sovereign risk that often deters private capital. By aligning these legal foundations with the Healthcare Financing Policy [35] and the Health Sector Transformation Plan II (HSTP II) [24], the government has created a roadmap for strengthening health systems. In terms of market maturity, this creates a model of regulatory certainty, moving the sector from a reliance on informal goodwill toward a credible, legally enforceable environment.

### Sub-theme 2: Structural and operational opportunities in Ethiopia’s PPPs model

A significant operational opportunity lies in the dual-mechanism of centralized procurement and adaptive governance. While procurement is centralized within the Ministry of Finance to ensure alignment with national priorities, the framework maintains the flexibility to respond to urgent social demands. As one respondent noted:

“Our law about PPP is centralized… the core of procurement… is done within the PPP unit at the Ministry of Finance” (R2).

This adaptive governance mechanism directly fosters as evidenced by the rapid pivot toward medical oxygen production during the COVID-19 pandemic. R2 further highlighted:

“There is flexibility according to the demand, even though priority areas were set as infrastructure.”

This adaptability influences diagnostic PPP feasibility by allowing for diverse concessional models such as Build-Operate-Transfer (BOT) or Design-Build-Finance-Operate-Maintain-Transfer (DBFOMT) [34].

### Sub-theme 3: Policy-Led Capacity Building and Health System Strengthening

Finally, Ethiopia’s framework emphasizes capacity development as a core component of system sustainability. Policies such as the HSTP II [24], and the Healthcare Financing Policy [35] focus on knowledge transfer and workforce participation to ensure that partnerships are not merely “extractive” but contribute to the long-term growth of the domestic health system.

This capacity-building mechanism exists because the government recognizes that persistent shortages in specialized diagnostic professionals and high infrastructure costs require a move toward local content and technical training. This directly influences PPP feasibility by ensuring that the local workforce is prepared to operate advanced diagnostic systems alongside private partners. By prioritizing these elements, the government is attempting to advance the diagnostic sector toward greater operational and technical readiness, where local expertise is developed alongside private investment, ensuring that diagnostic services remain a sustainable, domestically managed capability.

### Theme 3: Challenges in implementing PPPs in Ethiopia’s healthcare system

While the legal framework creates opportunities, the findings reveal significant barriers that impede effective execution. These challenges reflect the “early-stage” nature of Ethiopia’s PPP market maturity, where gaps in inter-sectoral trust, procedural legitimacy, and institutional capacity create friction in project implementation.

### Sub-theme 1: Legal barriers and operational inefficiencies

Participants highlighted that while a legal and regulatory framework exists, its operationalization often creates delays in project initiation. Regulatory approvals are slowed by infrequent board meetings and complex bureaucracy:

“Regulatory approval delays and infrequent board meetings prolonged the process” (R1).

Additionally, the framework is perceived as an inflexible “one-size-fits-all” model:

“They tend to focus on ‘you can do this’ or ‘don’t do that.’ This often paralyzes action…” (R3).

Although the framework provides a formal basis for PPPs, its rigidity and slow implementation undermine regulatory legitimacy, as the private sector experiences government oversight more as a bottleneck than a supportive mechanism, thereby limiting PPP feasibility.

### Sub-theme 2: Trust deficits and historical influences

Trust is a critical factor for successful partnerships, yet it is often undermined by historical ideological residue and ambiguous financial arrangements. This mistrust is rooted in Ethiopia’s legacy of socialism, which fosters a public sector perception that private entities prioritize profit over public welfare. As one respondent clarified:

“There is a trust, but it is below optimal… there should be a clearly written law and hence the government itself will have a trust…” (R3).

This trust deficit directly influences PPP feasibility by creating friction during negotiations; without mutual confidence, the risk-sharing essential to market maturity is replaced by defensive posturing.

### Sub-theme 3: Limited stakeholder representation and coordination

A major issue identified was the exclusionary governance mechanism, where the private sector was largely excluded from the development of initial PPP policies. This led to a lack of alignment between the regulatory framework and operational realities. As one respondent put it:

“This happened because… the practice of having the public and the private organizations work together did not exist” (R11).

This lack of representation hinders the social legitimacy of the PPP system, as the laws fail to accommodate the practicalities of diagnostic operations.

### Sub-theme 4: Weak monitoring and evaluation mechanisms

The findings reveal a transparency deficit mechanism, where M&E systems are either underdeveloped or poorly enforced. This results in inefficiencies and a lack of accountability:

“There has to be a proper system for monitoring and evaluation… without this, there is a risk of abuse of resources” (R4).

In the context of PPP feasibility, weak M&E increases the risk for both parties, as it becomes difficult to verify service quality or justify the “Value for Money” of the partnership, a key requirement for an evolving PPP market.

### Sub-theme 5: Financial constraints and workforce skill gaps

Finally, PPP implementation is hindered by a structural capacity-misalignment. Local investors often lack the financial resources and expertise for complex diagnostic projects:

“The budget and the experts required… may be difficult to handle by the local investors” (R2).

This is compounded by workforce challenges and outdated curricula:

“Inadequate workforce training in PPPs is exacerbated by outdated educational curricula” (R3).

Addressing these capacity development gaps is essential for market maturation and local participation.

## Discussion

This study provides a contextual analysis of the diagnostic service landscape and the institutional readiness for PPPs in Ethiopia. The findings reveal a healthcare system in a state of transition; while a robust legal architecture has established institutional legitimacy, persistent deficits in inter-sectoral trust and local technical capacity indicate that Ethiopia’s diagnostic PPP market is currently in an emerging stage of maturity [36].

The diagnostic landscape in Ethiopia is in an emerging stage of maturity and is undergoing a strategic realignment toward a more decentralized, technology-driven system. However, our findings indicate that this progress remains concentrated in specific centers of excellence. Institutions such as St. Paul’s are advancing rapidly by adopting high-throughput automation and digital pathology, yet this level of capacity is not yet representative across the country. This modernization reflects strong sectoral ambition and aligns with the national goal of universal hospital digitalization by 2030 [37]. Despite these advances, progress is hindered by a maintenance-decoupling mechanism, in which the procurement of advanced diagnostic assets is separated from the resources required to sustain them. Our findings align with existing evidence showing that over 50% of capital medical equipment in Ethiopian general hospitals is non-functional at any given time due to this misalignment [38]. These results suggest that the traditional procurement model has reached its limits. In this context, PPPs may help address critical operational weaknesses. Beyond mobilizing additional resources, partnerships with private actors could strengthen supply chain management, institutionalize preventive maintenance, and build local technical capacity, directly mitigating the gaps identified in the current system [39, 40].

The findings reveal that Ethiopia’s diagnostic sector is supported by three primary strategic opportunities that facilitate a conducive environment for PPP integration: legal formalization, centralized oversight, and adaptive flexibility. The transition from informal arrangements to the 2017 PPP Proclamation represents a shift toward institutional legitimacy. This aligns with World Bank and OECD principles, which suggest that legal clarity is the primary mechanism for reducing the sovereign risk that historically deters private capital in LMICs [41, 42]. By mandating benchmarks like “Value-for-Money” and Affordability, the Ethiopian framework moves beyond ad hoc goodwill into a state of regulatory certainty, which peer-reviewed research identifies as a prerequisite for lowering transaction costs and fostering competitive bidding [43–45].

Centralized PPP governance offers a strategic opportunity for Ethiopia’s emerging health PPP market. National-level oversight ensures standardized procedures, strong accountability, and reduced corruption risk, which can arise in decentralized systems with limited local transparency [46, 47]. It also enhances fiscal credibility, risk management, and investor confidence by providing uniform regulations and predictable processes, critical in early-stage PPP environments [48, 49].Experiences from countries like Brazil, India, and Australia show that centralized oversight is advantageous in low-maturity PPP markets, while decentralization can follow once subnational entities develop sufficient capacity and fiscal strength [46, 48] Thus, Ethiopia’s centralized PPP approach provides a solid foundation for scaling health partnerships while mitigating risks and strengthening governance.

However, decentralization also offers potential advantages. Subnational or municipal PPP governance can increase responsiveness to local needs, and foster innovation, once market maturity and institutional capacity improve. Experiences from Brazil, Canada, and India demonstrate that regional or municipal PPP cells can complement central oversight, allowing projects to be tailored to local contexts while still benefiting from centralized guidance and risk-sharing mechanisms [50, 51]. Finally, the adaptive flexibility of the framework demonstrated by the sector’s pivot during the COVID-19 pandemic confirms that the system is a resilient mechanism for priority-setting. This operational agility reflects global recommendations for sector-responsive frameworks that enable governments to reprioritize resources during health crises [52].

A key challenge for PPPs in Ethiopia is the regulatory environment. Although a legal framework for PPPs exists, it was originally designed for general infrastructure rather than health and diagnostic services, and its operationalization is often rigid and slow. Prolonged procurement procedures, bureaucratic delays, and a one-size-fits-all approach were reported to delay project approvals, increase transaction costs, and reduce private sector engagement. These findings align with evidence from LMICs, including sub-Saharan Africa, where poorly tailored regulatory frameworks have consistently slowed implementation, dampened investor interest, and restricted innovation in health PPPs [17, 53, 54].

Beyond regulatory hurdles, a profound socio-institutional barrier to PPP implementation in Ethiopia is the pervasive trust deficit between public and private actors, further exacerbated by constrained local capacity. This mistrust appears rooted in historical ideological path-dependency, where a legacy of state-centric governance fosters skepticism toward private sector profit motives within essential health services. Such perceptions undermine the collaborative synergy and equitable risk-sharing mechanisms indispensable for partnership sustainability. Consistent with global frameworks, the literature identifies trust, institutional legitimacy, and capacity development as the three pillars of a functional PPP ecosystem [55, 56]. In the Ethiopian context, deficiencies in these dimensions reflect a low state of market maturity, effectively acting as a systemic deterrent to long-term private sector engagement. This aligns with evidence from other LMICs, where trust deficits are shown to stifle negotiation, marginalize private participation, and ultimately compromise the operational resilience of health partnerships [57–60].

The analysis further highlights that local investors face significant financial barriers, representing a major constraint to effective PPP implementation in Ethiopia. High project costs limit the ability of domestic actors to participate meaningfully, reducing competition and engagement in early-stage partnerships. This reflects a system-level gap in capacity development, where the broader enabling environment does not provide adequate financial support, risk-sharing mechanisms, or incentives to foster local private sector involvement. International experience shows that governments often use targeted financial support such as grants, viability gap funding, equity contributions, tax incentives, and output-based subsidies to share risk and enhance the bankability of PPP projects, particularly in contexts where capital markets are less mature and private investor confidence is low [61, 62]. These deficiencies not only reduce private sector participation but also threaten the sustainability and scalability of PPP initiatives [17, 63]. Similar financial constraints have been documented in other low-income countries, including Malawi, emphasizing that strengthening financial access and capacity for local actors is essential for developing a mature and resilient PPP market [64].

Our study identified the absence of strong M&E systems for PPP projects in Ethiopia as a major barrier to effective implementation. Without systematic monitoring, it is difficult to track performance, ensure accountability, and identify operational issues early. Global evidence shows that robust M&E frameworks are critical for adaptive management, continuous improvement, and sustainability of PPPs. For example, in Kenya, participatory M&E improved project planning, performance, and stakeholder accountability, reducing conflicts and enhancing private sector confidence [65].

While this study provides important insights into PPP implementation for diagnostic services in Ethiopia, it is not without limitations. First, it focused on national-level stakeholders in Addis Ababa, so it may not capture challenges that could arise at regional or local levels. Second, the sample mainly included senior policymakers and technical experts, potentially underrepresenting perspectives from frontline healthcare providers. Thus, findings should be interpreted cautiously when considering broader or future PPP implementation.

Despite these limitations, the findings offer important insights for LMICs. Ethiopia’s diagnostic PPP experience shows that flexible, sector-specific frameworks, inclusive stakeholder engagement, strengthened local capacity, and a balance of centralized oversight with operational flexibility are critical to enhance PPP feasibility.

## Conclusion and recommendation

This study examines Ethiopia’s diagnostic PPP landscape, highlighting opportunities such as a formal legal framework, centralized oversight ensuring national alignment and procedural standardization, and potential collaborations with private actors to strengthen service delivery, supply chains, and workforce capacity. Challenges include rigid regulations, trust deficits between public and private actors, limited local financial and operational capacity, and insufficient stakeholder inclusion. To improve PPP effectiveness, policymakers should: (i) allow sector-specific flexibility in legal and governance frameworks; (ii) build trust and legitimacy through early, inclusive stakeholder engagement and (iii) strengthen local capacity and financial mechanisms to support meaningful private-sector participation. Addressing these factors holistically can make PPPs in Ethiopia more feasible, scalable, and aligned with broader health system goals.

## Supplementary Information

Below is the link to the electronic supplementary material.

Supplementary Material 1

Supplementary Material 2

Supplementary Material 3

Supplementary Material 4

Supplementary Material 5

Supplementary Material 6

## Data Availability

The datasets used and/or analyzed during the current study are available from the corresponding author on reasonable request.

## Abbreviations

AHDP: African Diagnostic Platform
LMIC: Low-Middle Income Country
PPP: Public Private Partnership
