# Supplementary material for: Range of hurdles and opportunities for developing PPPs in diagnostics: a contextual analysis, Ethiopia
Source: BMC Health Serv Res. 2026 Mar 28;26:651. doi: 10.1186/s12913-026-14354-z (PMC13151300; doi:10.1186/s12913-026-14354-z)
Supplement: Supplementary file 1 — Supplementary Material 1 [file 12913_2026_14354_MOESM1_ESM.pdf]

የኢትዮጵያ ፌደራላዊ ዲሞክራሲያዊ ሪፐብሊክ

ፌደራል ነጋሪት ጋዜጣ

FEDERAL NEGARIT GAZETTE

OF THE FEDERAL DEMOCRATIC REPUBLIC OF ETHIOPIA

ሃያ አራተኛ ዓመት ቁጥር ፳፰
አዲስ አበባ የካቲት ፲፭ ቀን ፪ሺ፲ ዓ.ም

በኢትዮጵያ ፌደራላዊ ዲሞክራሲያዊ ሪፐብሊክ
የሕዝብ ተወካዮች ምክር ቤት ጠባቂነት የወጣ

24th Year No 28
ADDIS ABABA 22nd Feburary,2018

ማውጫ

አዋጅ ቁጥር ፩ሺ፸፮/፪ሺ፲ ዓ.ም

የመንግሥት እና የግል አጋርነት አዋጅ......ገጽ ፲ሺ፪፻፴፭

Content

Proclamation No. 1076/2018

Public Private Partnership Proclamation.....Page10235

አዋጅ ቁጥር ፩ሺ፸፮/፪ሺ፲

ስለመንግሥት እና የግል አጋርነት የወጣ አዋጅ

የመሠረተ ልማት ሥርዓቱን ጨምሮ የአገሪቱን የልማት ግቦች ከማሳካት አንጻር የግሉ ሴክተር ወሳኝ ስትራቴጂያዊ አጋር በመሆኑ፤

ግልጽነትን፣ ፍትሐዊነትን እና ዘላቂነትን በማስፈን እና በግሉ ዘርፍ የፋይናንስ አቅም የሚገነቡ የመሠረተ-ልማት ፕሮጀክቶችን ለማበረታታት የሚረዳ አመቺ የሕግ ማዕቀፍ በማስፈለጉ፤

ለመሠረተ-ልማት ግንባታ ፕሮጀክቶች የጨረታ አሸናፊዎችን ለመምረጥ የሚያስችል የተለየ ሥነ-ሥርዓት በመዘርጋት መንግሥታዊ አካላት የጨረታ አሸናፊዎችን በመምረጥ ረገድ ሊከተሉት የሚገባውን የግልጽነት፣ ኢኮኖሚያዊነት እና የፍትሐዊነት አጠቃላይ መርህ ማዳበር በማስፈለጉ፤

በኢትዮጵያ ፌደራላዊ ዲሞክራሲያዊ ሪፐብሊክ ህገመንግሥት አንቀጽ ፶፭ (፩) መሠረት የሚከተለው ታውጇል፡-

Proclamation No. 1076/2018

A PROCLAMATION TO PROVIDE FOR THE PUBLIC PRIVATE PARTNERSHIP

WHEREAS, the participation of the private sector is recognized an essential strategy to realize the country's development objectives, including the infrastructure system

WHEREAS, it is desirable to establish a favorable legislative framework to promote and facilitate the implementation of privately financed infrastructure projects by enhancing transparency, fairness and long-term sustainability;

WHEREAS, it has become necessary to further develop the general principles of transparency, economy and fairness in the award of contracts by public entities through the establishment of specific procedures for the award of infrastructure projects;

NOW, THEREFORE, in accordance with Article 55(1) of the Constitution of the Federal Democratic Republic of Ethiopia, it is hereby proclaimed as follows:

ክፍል አንድ

ጠቅላላ

፩. አጭር ርዕስ

ይህ አዋጅ "የመንግሥት እና የግል አጋርነት አዋጅ ቁጥር ፩ሺ፸፮/፪ሺ፲" ተብሎ ሊጠቀስ ይችላል።

፪. ትርጓሜ

የቃሉ አገባብ ሌላ ትርጉም የሚያሰጠው ካልሆነ በቀር በዚህ አዋጅ ውስጥ፦

፩/ "ቦርድ" ማለት በዚህ አዋጅ መሠረት የሚቋቋም ቦርድ ነው፤

፪/ "ተዋዋይ ባለስልጣን" ማለት ከግል ባለሀብት ጋር የመንግሥት እና የግል አጋርነት ስምምነት ለማድረግ የሚፈልግ የመንግሥት መሥሪያ ቤት፣ የመንግሥት የልማት ድርጅት ነው፤

፫/ "የመሠረተ-ልማት አውታር" ማለት በቀጥታም ሆነ በተዘዋዋሪ የሕዝብ አገልግሎት ተግባራትን የሚያከናውኑ ግዙፋዊ ሀልወት ያላቸው አውታሮች እና ስርዓቶች ናቸው፤

፬/ "ሚኒስቴር" ወይም "ሚኒስትር" ማለት እንደአግባብነቱ የገንዘብና ኢኮኖሚ ትብብር ሚኒስቴር ወይም ሚኒስትር ነው፤

፭/ "ሰው" ማለት የተፈጥሮ ሰው ወይም በሕግ የሰውነት መብት የተሰጠው አካል ነው፤

፮/ "የግል ባለሀብት" ማለት ከተዋዋይ ባለስልጣን ጋር የመንግሥት እና የግል አጋርነት ስምምነት የሚያደርግ ወገን ነው፤

፯/ "የፕሮጀክት ስምምነት" ማለት በተዋዋይ ባለስልጣን እና በግል ባለሀብት መካከል ለፕሮጀክቱ ዓላማ የሚደረግ የመንግሥት እና የግል አጋርነት ስምምነትና ሌሎች ስምምነቶች ናቸው፤

CHAPTER ONE

GENERAL

1. Short Title

This Proclamation may be cited as "Public Private Partnership Proclamation No. 1076/2018".

2. Definitions

In this Proclamation unless the context requires otherwise:

1. "Board" means the board established pursuant to this Proclamation.
2. "Contracting Authority" means a Public Body or a Public Enterprise which intends to enter into a Public Private Partnership Agreement with a Private Party.
3. "Infrastructure Facility" means any physical facility and systems that directly or indirectly provide Public Service Activity.
4. "Ministry" or "Minister" means the Ministry or Minister of Finance and Economic Cooperation, respectively.
5. "Person" means any natural or juridical person.
6. "Private Party" means a party that enters into a Public Private Partnership Agreement with a Contracting Authority.
7. "Project Agreement" means the Public Private Partnership Agreement and other agreements entered into between the Contracting Authority or another Public Entity and the Private Party for the purpose of the project.

፰/ "የፕሮጀክት ኩባንያ" ማለት በኢትዮጵያ ፌደራላዊ ዲሞክራሲያዊ ሪፐብሊክ ሕጎች መሠረት በአሸናፊ ተጫራች በኢትዮጵያ ውስጥ የሚመሰረትና ዓላማውም የመንግሥት እና የግል አጋርነት ስምምነቱን እና እንደአግባብነቱም ሌሎች የፕሮጀክት ስምምነቶችን የሚተገብርና የሚያስፈጽም የሕግ ሰውነት የተሰጠው ኩባንያ ነው፤

፱/ "የመንግሥት መሥሪያ ቤት" ማለት ሙሉ በሙሉ በፌደራል መንግሥት በጀት የሚተዳደር ማናቸውም የፌደራል መንግሥት መስሪያ ቤት ነው፤

፲/ "የመንግሥት የልማት ድርጅት" ማለት ሙሉ በሙሉ በፌደራል መንግሥት ባለቤትነት ሥር የሆነና አግባብነት ባላቸው በኢትዮጵያ ፌደራላዊ ዲሞክራሲያዊ ሪፐብሊክ ሕጎች የተቋቋመ ድርጅት ነው፤

፲፩/ "መንግሥታዊ ተቋም" ማለት የመንግሥት መስሪያ ቤትን እና የመንግሥት የልማት ድርጅትን ያጠቃልላል፤

፲፪/ "የመንግሥት እና የግል አጋርነት" ወይም "መግአ" ማለት በተዋዋይ ባለስልጣን እና በግል ባለሀብት መካከል የሚደረግ ለረጅም ጊዜ የሚቆይ ስምምነት ሲሆን ይህም ስምምነት የግል ባለሀብቱ፦

ሀ) በተዋዋይ ባለስልጣን ሊከናወን ይችል የነበረን የሕዝብ አገልግሎት ሥራ ለማከናወን የውል ግዴታ የሚገባበት፤

ለ) በሚከተለው አኳኋን ጥቅም የሚያገኝበት ነው፦

(፩) በተዋዋይ ባለስልጣን ወይም ስለተዋዋይ አካል ሆኖ የተፈጸመን ክፍያ፤

(፪) ከአገልግሎት ተጠቃሚዎች ወይም ደንበኞች በታሪፍ ወይም በክፍያ መልክ የሚሰበሰብ ገንዘብ፤ እና

8. "Project Company" means the legal entity incorporated under the laws of the Federal Democratic Republic of Ethiopia by the successful bidder whose sole purpose shall be to execute and implement the Public Private Partnership Agreement and other Project Agreements, if any.

9. "Public Body" means any organ of the Federal Government, which is wholly financed by the Federal Government budget.

10. "Public Enterprise" means an enterprise fully owned by the Federal Government and defined under the relevant laws of the Federal Democratic Republic of Ethiopia.

11. "Public Entity" means a Public Body or a Public Enterprise.

12. "Public Private Partnership" or "PPP" means a long-term agreement between a Contracting Authority and a Private Party under which a Private Party:

a) undertakes to perform a Public Service Activity that would otherwise be carried out by the Contracting Authority;

b) receives a benefit by way of :

(1) compensation by or on behalf of the Contracting Authority;

(2) tariffs or fees collected by the Private Party from users or consumers of a service; and

(፫) እነዚህን ታሪፎች ወይም ክፍያዎች በጣምራ፤

ሐ/ በፕሮጀክት ስምምነትመሠረት ከሥራው እንቅስቃሴ ወይም በመንግሥት ንብረት ከመጠቀም ጋር ለተያያዙ ሥጋቶች ኃላፊነት የሚወስድበት ነው።

፲፫/ "የመንግሥት እና የግል አጋርነት ስምምነት" ማለት የመንግሥት እና የግል አጋርነቱን የውል ቃሎችና ሁኔታዎች ለመወሰን በተዋዋይ ባለስልጣን እና በግል ባለሀብቱ መካከል የሚደረግ ውል ነው፤

፲፬/ "የሕዝብ አገልግሎት ተግባር" ማለት ለሕዝብ ጥቅም አስፈላጊ በመሆኑ እና በግል ዘርፍ ብቻ በብቃት ሊሰራ እንደማይችል የታመነበት በመሆኑ ምክንያት በመንግሥት የሚከናወን ማናቸውም ተግባር ነው፤

፲፭/ "የመንግሥት እና የግል አጋርነት ዳይሬክቶሬት ጄኔራል" ወይም "የመግአ ዳይሬክቶሬት ጄኔራል" ማለት በዚህ አዋጅ መሠረት የሚቋቋም ተጠሪነቱ ለሚኒስትሩ የሆነ የሥራ ክፍል ነው፤

፲፮/ "የክልል መንግሥታት" ማለት በኢትዮጵያ ፌደራላዊ ዲሞክራሲያዊ ሪፐብሊክ ሕገ-መንግሥት አንቀጽ ፵፯(፩) የተዘረዘሩት የክልል መንግሥታት ሲሆኑ ለዚህ አዋጅ አፈጻጸም የአዲስ አበባ እና የድሬዳዋ ከተማ አስተዳደሮችንም ይጨምራል፤

፲፯/ "በራስ ተነሳሽነት የቀረበ የፕሮጀክት ሀሳብ" ማለት በጨረታ ውድድር ሥነ-ሥርዓት በመግአ ዳይሬክቶሬት ጄኔራል ሳይጠየቅ ወይም ሳይጋበዝ የመንግሥት እና የግል አጋርነት ፕሮጀክት በራስ ተነሳሽነት የቀረበ ሀሳብ ነው፤

(3) a combination of such compensation and such charges or fees.

c) is generally liable for risks arising from the performance of the activity or use of the state property in accordance with the terms of the Project Agreements.

13. "Public Private Partnership Agreement" means a contract concluded between the Contracting Authority and a Private Party setting forth the terms and conditions of the Public Private Partnership.

14. "Public Service Activity" means any activity the government has decided to perform for the reason that it has deemed it to be necessary in the general interest of the public and considered that private initiative was inadequate for carrying it out.

15. "Public Private Partnership Directorate General" or "PPP Directorate General" means the unit established within the Ministry pursuant to this Proclamation;

16. "Regional States" means any region referred to in Article 47(1) of the Constitution of the Federal Democratic Republic of Ethiopia and includes the Addis Ababa and Dire Dawa city administrations;

17. "Unsolicited Proposal" means any proposal undertaking a Public Private Partnership project that is not submitted in response to a request or solicitation issued by the Contracting Authority within the context of a competitive selection procedure.

፲፰/ "ለገንዘብ ተመጣጣኝ ዋጋ ማስገኘት" ማለት በተዋዋዩ ባለስልጣን ሊከናወን ይገባ የነበረን የሕዝብ አገልግሎት ተግባር በመንግሥት እና በግል አጋርነት መሠረት በግል ባለሀብት በሚከሄድበት ወቅት የሕዝብ አገልግሎት ተግባሩ ወይም የመንግሥት ንብረቱ ካሉት ሌሎች አማራጮች አንጻር ሲመዘን ሊያስገኝ የሚችለውን የተሻለ ኢኮኖሚያዊ ጠቀሜታ ግምት ውስጥ በማስገባት በተለይም የህዝብ አገልግሎት ተግባሩ ለተዋዋዩ ባለስልጣን ወይም ለተጠቃሚዎች ከወጪ፣ ከዋጋ፣ ከጥራት፣ ከብዛት፣ ከጊዜ እና ከሌሎች መመዘኛዎች አንጻር የሚያስገኘው የጠቀሜታ መጠን ነው፤

፲፱/ በዚህ አዋጅ ውስጥ ማንኛውም በወንድ ጾታ የተገለጸው የሴትንም ጾታ ይጨምራል።

፫. ዓላማ

የመንግሥትና የግል አጋርነት ሥርዓት የሚከተሉት ዓላማዎች ይኖሩታል፤

፩/ የኢትዮጵያን የኢኮኖሚ ዕድገት ለማገዝ የሚችሉና በግል ባለሀብቶች ፋይናንስ የሚደረጉ ፕሮጀክቶችን ለማበረታታትና ለመደገፍ የሚያስችል አመቺ ማዕቀፍ እንዲኖር ማድረግ፤

፪/ ግልጽነትን፣ፍትሀዊነትን፣ ለገንዘብ ተመጣጣኝ ዋጋ ማስገኘትን፣ ቅልጥፍናን እና ዘላቂነትን ማበረታታት፤

፫/ የሕዝብ አገልግሎት ተግባርን የአቅርቦት መጠን እና ጥራት ማሻሻል፤ እና

፬/ የመንግሥት ዕዳ ዕድገትን በመቀነስ የተረጋጋ ማክሮ ኢኮኖሚእንዲኖር ማድረግ።

18. "Value for Money" means that the undertaking of a Public Service Activity of the Contracting Authority by a Private Party under a Public Private Partnership results in a net benefit accruing to that Contracting Authority or consumer defined in terms of cost, price, quality, quantity, timeliness of implementation and other factors which influence the determination of the best economic value compared to other options of delivering this Public Service Activity or use of government property.

19. any expression in the masculine gender includes the feminine.

3. **Objective**

This Public Private Partnership Scheme shall have the following objectives:

1/ to create a favorable framework for promoting and facilitating the implementation of privately financed projects to support Ethiopian economic growth;

2/ to enhance transparency, fairness, Value for Money, efficiency and long-term sustainability;

3/ to improve quality of Public Service Activity; and

4/ to maintain macroeconomic stability by reducing growth in public debt.

፬. የተፈጻሚነት ወሰን

፩/ ይህ አዋጅ በመንግሥት መስሪያ ቤቶችና በመንግሥት የልማት ድርጅቶች በሚተገበሩ የመንግሥትና የግል አጋርነት ፕሮጀክቶች ላይ ተፈፃሚ ይሆናል።

፪/ ይህ አዋጅ በሚከተሉት ላይ ተፈጻሚ አይሆንም፦

ሀ) ነዳጅ፣ ማዕድን፣ ሚኒራል፣ የአየር ክልል መብት፣ እና

ለ) የመንግሥት የልማት ድርጅትን ወይም በመንግሥት ባለቤትነት ሥር ያለን መሠረተ-ልማት እና ወደ ግል ማዛወር ወይም ሽያጭ።

፭. የመንግሥት እና የግል አጋርነት ዓይነቶች

፩/ የመንግሥት እና የግል አጋርነት የሚከተሉትን ተግባራት በተናጠል ወይም በማጣመር ሊይዝ ይችላል፦

ሀ) የአዳዲስ የመሠረተ-ልማት አውታሮችን ዲዛይን፣ ግንባታ፣ ፋይናንስ፣ ጥገና እና ሥራ ማስኬድ፤

ለ) የነባር የመሠረተ-ልማት አውታሮችን መልሶ ግንባታ፣ ማዘመን፣ ፋይናንስ፣ ማስፋፋት፣ ጥገና እና ሥራ ማስኬድ፤ እና/ወይም

ሐ) የአዲስ ወይም ነባር የመሠረተ-ልማት አውታሮችን አስተዳደር፣ ሥራ አመራር፣ ሥራ ማስኬድ ወይም ጥገና።

፪/ ተዋዋዩ ባለስልጣን በእያንዳንዱ ስምምነት ላይ ሊኖሩ የሚችሉ ስጋቶችን እና ኃላፊነቶችን በመደልደል ረገድ አግባብነት አለው ብሎ የሚያምንበትን የውል ዓይነት መምረጥ ይኖርበታል።

፮. ተዋዋይ ባለስልጣን

፩/ የመንግሥት እና የግል አጋርነትን ሊያስፈጽም የሚችለው ተዋዋይ ባለስልጣን በመንግሥት እና የግል አጋርነት አማካኝነት ሊሰራ ለታቀደው የመሠረተ-ልማት አገልግሎት በሕግ ኃላፊነት የተሰጠው መንግስታዊ ተቋም ነው።

4. Scope of Application

1/ This Proclamation shall apply to Public Private Partnership projects of Public Bodies and Public Enterprises;

2/ The following activities are expressly excluded from this Proclamation:

a) oil, mines, minerals, rights of air space; and

b) Privatization or divestiture of public infrastructure or public enterprises.

5. Forms of Public Private Partnerships

1/ Public private partnerships may involve any of the following activities or any combination thereof:

a) The design, construction, financing, maintenance or operation of new Infrastructure Facilities.

b) The rehabilitation, modernization, financing, expansion, maintenance or operation of existing Infrastructure Facilities; and/or

c) The administration, management, operation or maintenance pertaining to new or existing Infrastructure Facilities.

2/ The Contracting Authority shall select the form of contract which reflects the desired allocation of risks and responsibilities for each agreement.

6. Contracting Authority

1/ The Contracting Authority for a Public Private Partnership shall be the Public Entity legally mandated to be responsible for the infrastructure to be delivered by way of the Public Private Partnership.

፪/ ሊሰራ የታቀደው የመንግሥት እና የግል አጋርነት ፕሮጀክት የሚመለከታቸው የመሠረተ-ልማት አውታሮች እና/ወይም አገልግሎቶች ከአንድ በላይ በሆነ የመንግሥት ተቋም ኃላፊነት ሥር የሚወድቁ በሚሆንበት ጊዜ ከእነዚህ ድርጅቶች መካከል ቦርዱ ተለይቶ የቀረበውን ፕሮጀክት ለማስፈጸም የበለጠ አግባብነት አለው ብሎ የሚያምንበትን የመንግሥት ተቋም ይወስናል።

፯. **የመንግሥት እና የግል አጋርነት ፈቃድ የመስጠት ሥልጣን**

ተዋዋዩ ባለስልጣን በተቋቋመበት ሕግ በኃላፊነቱ ሥር እንዲሆኑ የተደረጉትን የመንግሥት ንብረት የሆኑ የመሠረተ-ልማት አውታሮችን የመጠቀም ወይም የሥራ ማካሄድ መብትን እና/ወይም የመንግሥት አገልግሎት ተግባራትን የማከናወን መብትን በዚህ አዋጅ መሠረት ለግል ባለሀብት መስጠት ይችላል።

ክፍል ሁለት

የመንግሥት እና የግል አጋርነት አስተዳደር

፰. **የመንግሥት እና የግል አጋርነት ቦርድ**

፩/ ከዚህ በታች የተዘረዘሩትን አካላት የያዘ የመንግሥት እና የግል አጋርነት ቦርድ በዚህ አዋጅ ተቋቁሟል፤

ሀ) የገንዘብ እና ኢኮኖሚ ትብብር ሚኒስቴር፤

ለ) የኢትዮጵያ ብሔራዊ ባንክ፤

ሐ) የውሃ፣ መስኖና ኤሌክትሪክ ሚኒስቴር፤

መ) የትራንስፖርት ሚኒስቴር፤

ሠ) የመንግሥት የልማት ድርጅቶች ሚኒስቴር፤

ረ) የብሔራዊ ፕላን ኮሚሽን፤

ሰ) የፌደራል እና አርብቶ አደር ጉዳዮች ሚኒስቴ፤

2. In the event that a Public Private Partnership project involves infrastructures and/or services which are the responsibility of more than one Public Entity, the Board shall among Public entities select the appropriate Contracting Authority for this specific project.

7. **Authorization to grant rights for Public Private Partnerships**

Rights to use or operate publicly owned Infrastructure Facilities and/or provide Public Service Activities may be granted by the Contracting Authority which by its establishing legislation is responsible for such activities, to a Private Party in accordance with this law.

CHAPTER TWO

ADMINISTRATION OF PUBLIC PRIVATE PARTNERSHIPS

8. **Public Private Partnership Board**

1/ It is hereby established the Public Private Partnership Board which shall consist of:

a) The Ministry of Finance and Economic Cooperation;

b) The National Bank of Ethiopia;

c) The Ministry of Water, Irrigation and Electricity;

d) The Ministry of Transport;

e) The Ministry of Public Enterprises;

f) The National Planning Commission;

g) The Ministry of Federal and Pastoralist Affairs;

ሸ) የግሉ ሴክተር ከሚወክሉ ተቋማት በሚኒስትሩ አማካኝነት የሚመረጡ ሁለት አባላት።

፪/ የገንዘብ እና ኢኮኖሚ ትብብር ሚኒስቴር ሚኒስትር ቦርዱን በሰብሳቢነት ይመራል።

፫/ የመግአ ዳይሬክቶሬት ጄኔራል ዋና ዳይሬክተር የቦርዱ ፀሐፊ ሆኖ ይሰራል።

፱. <u>የቦርዱ ኃላፊነት</u>

፩/ ቦርዱ የሚከተሉት ኃላፊነቶች ይኖሩታል፤

ሀ) የጨረታው ሂደት ከመጀመሩ አስቀድሞ አግባብነት ያላቸውን የአዋጭነት ጥናቶችን ጨምሮ የመግአ ፕሮጀክትን አወቃቀር ያጸድቃል፤

ለ) በጨረታው ሂደት ወቅት ወይም ስምምነት ከመፈረሙ በፊት በመጀመሪያው የፕሮጀክቱ አወቃቀር ላይ በስጋት ድልድል እና በገንዘብ ፍሰት ረገድ የተደረጉ መሠረታዊ ለውጦችን መርምሮ ያጸድቃል፤

ሐ) በፕሮጀክት ስምምነት ላይ የሚደረጉ መሠረታዊ ማሻሻያዎችን መርምሮ ያጸድቃል፤

መ) አስፈላጊ ሆኖ ሲገኝ ለተለዩ ፕሮጀክቶች አግባብነት ያለውን ተዋዋይ ባለስልጣን ይሰይማል፤

ሠ) ለተለዩ ፕሮጀክቶች የታክስ ወይም ሌላ ማበረታቻ ማድረግ የሚያስፈልግ ሆኖ ሲያገኘው ለሚመለከተው የመንግሥት አካል የውሳኔ ሃሳብ ያቀርባል፤

ረ) በመንግሥት እና በግል አጋርነት ተሳታፊ የሚሆኑ የግል ባለሀብቶችን ለመምረጥ የተካሄደውን ጨረታ ወይም ድርድር ውጤት ያፀድቃል፤

ሰ) በዚህ አዋጅ አንቀጽ ፵፯ መሠረት የቀረበን የመንግሥት ድጋፍ እና ዋስትና ጥያቄ በሚመለከት የውሳኔ ሃሳብ ያቀርባል።

h) Two members from the private sector appointed by the Minister.

2/ The Minister of Finance and Economic Cooperation shall chair the Board.

3/ The PPP Directorate General shall act as secretariat to the Board.

9. <u>Responsibilities of the Board</u>

1/ The Board shall have the following responsibilities:

a) approve the PPP project structure (including any feasibility study related thereto) prior to the commencement of the tendering process;

b) approve significant changes to the risk allocation or expected cash flow from that envisioned under the initial structure pursuant to changes during the tendering process or prior to signing;

c) approve any significant amendment to a Project Agreement;

d) identify the appropriate Contracting Authority when required for specific projects;

e) recommend specific tax or other incentives for a particular project to the relevant authority;

f) approve the tender or negotiation results carried out to select the private sector which can participate in the Public Private Partnership;

g) Recommend government support requested in accordance with Article 47.

፪/ ቦርዱ ለገንዘብ ተመጣጣኝ ዋጋ የማያስገኙ ወይም የመግአ ዳይሬክቶሬት ጀኔራል የሚያወጣቸውን ዝቅተኛ መመዘኛዎች የማያሟሉ የመንግሥት እና የግል አጋርነት ፕሮጀክቶችን የሚመለከቱ መግአ ውሎችን ተዋዋዩ ባለስልጣን እንዳይፈርም ለመከልከል ይችላል።

፫/ ቦርዱ የመንግሥት መሥሪያ ቤት ወይም የመንግሥት የልማት ድርጅት አንድን ፕሮጀክት በመንግሥት እና በግል አጋርነት ፕሮጀክት መልክ እንዲያካሂድ ለማዘዝ ሥልጣን አለው። የመንግሥት መሥሪያ ቤቱ ወይም የመንግሥት የልማት ድርጅቱም የቦርዱን ትዕዛዝ የማክበር ግዴታ አለበት።

፬/ ቦርዱ እንደ አስፈላጊነቱ ሥልጣንና ኃላፊነቱን ለሚኒስቴሩ ወይም ለመንግሥት እና ለግል አጋርነት ዳይሬክቶሬት ጀኔራል በውክልና ለመስጠት ይችላል።

፭/ ቦርዱ ኃላፊነቱን ለመወጣት በየሩብ ዓመቱ ወይም እንደአስፈላጊነቱ ከዚያም ባነሰ ጊዜ ስብሰባ ያደርጋል።

፮/ ቦርዱ በዚህ አዋጅ የተሰጠውን ሥልጣንና ኃላፊነት ለመወጣት የሚያስችል መመሪያ ሊያወጣ ይችላል።

፲. የመግአ ዳይሬክቶሬት ጀኔራል

፩/ የመግአ ዳይሬክቶሬት ጀኔራል በዚህ አዋጅ በሚኒስቴሩ ውስጥ ተቋቁሟል።

፪/ ሚኒስቴሩ በመግአ ዳይሬክቶሬት ጀኔራል ውስጥ በቂ የሰው ኃይል መመደቡን የማረጋገጥ እንዲሁም ባለሙያዎችን ለማበረታታት የሚያስችል የማበረታቻ ሥርዓት እንዲኖር የማድረግ ኃላፊነት አለበት።

፲፩. የዳይሬክቶሬት ጀኔራሉ ዓላማ

የዳይሬክቶሬት ጀኔራሉ ዓላማ የመንግሥትና የግል አጋርነት በዚህ አዋጅ የተዘረጋውን ሥርዓት ተከትሎ እንዲፈፀም በማድረግ የአገሪቱን የልማት ግቦች ማሳካት ይሆናል።

2/ The Board shall have the power to prohibit a Contracting Authority from issuing any solicitation document and/or formalizing any agreement for any Public Private Partnership project which does not demonstrate Value for Money or meet minimum standards set forth by the PPP Directorate General.

3/ The Board shall have the power to instruct a Public Body or a Public Enterprise to carry out a project as a Public Private Partnership project. Such Public Body or a Public Enterprise shall comply with the instruction of the Board.

4/ The Board may consider delegating its responsibilities to the Ministry or the PPP Unit as it may consider appropriate.

5/ The Board shall meet quarterly, or more frequently as necessary, to carry out its responsibilities.

6/ The Board shall issue a guideline prescribing its working procedures.

10. PPP Directorate General

1/ A PPP Directorate General is hereby established within the Ministry.

2/ The Ministry shall be responsible to adequately staff the PPP Directorate General and for ensuring that there is an incentive mechanism to employees of the Directorate General.

11. Objectives of the Directorate General

The Directorate General shall have the objectives of achieving the country's development objectives by ensuring that public private partnerships are carried out as per the provisions of this Proclamation.

፲፪. <u>የዳይሬክቶሬት ጄኔራሉ ተግባርና ኃላፊነት</u>

ዳይሬክቶሬት ጄኔራሉ የሚከተሉት ተግባርና ኃላፊነቶች ይኖሩታል፦

፩/ የግሉ ዘርፍ በሕዝብ አገልግሎት ተግባራት ላይ በፋይናንሲንግ፣ በኮንስትራክሽን፣ በእድሳት እና በመምራት በኩል እንዲሳተፍ ያበረታታል፤

፪/ የመንግሥት እና የግል አጋርነትን በተመለከተ በሁሉም ጉዳዮች ለቦርዱ፣ለሚኒስቴሩና ለተዋዋይ ባለስልጣን የቴክኒክ ድጋፍና እርዳታ ያደርጋል፤

፫/ የመንግሥት እና የግል አጋርነትን የሕግ፣ የቁጥጥር፣ የመዋቅር እና የፖሊሲ ማዕቀፍ የሚመለከቱ የውሳኔ ሀሳቦችን ለቦርዱ ያቀርባል፤

፬/ በዚህ አዋጅ መሠረት የመንግሥት እና የግል አጋርነትን ተግባራዊ ለማድረግ በፕሮጀክት ዝግጅት፣ በግዥ ሥነ-ሥርዓት፣ በጨረታ ሰነድ ዝግጅትና በፕሮጀክት ስምምነት ወይም እንደ አስፈላጊነቱ ሌሎች ርዕሶችን የሚመለከቱ አስገዳጅነት ያላቸውን መመሪያዎች ያዘጋጃል፤ ሲፀድቅም ሥራ ላይ እንዲውሉ ያደርጋል፤

፭/ በዚህ አዋጅ ዓላማዎች መሠረት ፕሮጀክቶችን ይቀርጻል፤ ይለያል፤ በደረጃ ይወስናል፤

፮/ ሊተገበሩ የታቀዱ ፕሮጀክቶችን አዋጭነት ይመረምራል፤ አስተያየት ያቀርብባቸዋል፤ ለቦርዱ እና ለሚኒስቴሩ የውሳኔ ሃሳብ ያቀርባል፤

፯/ በመንግሥት እና የግል አጋርነት እንዲከናወኑ የተመረጡ ፕሮጀክቶችን መረጃዎች ያሰራጫል፤

፰/ በዚህ አዋጅ በተዘረጋው ሥርዓት መሠረት በመንግሥትና በግል አጋርነት ተሳታፊ የሚሆኑ የግል ባለሀብቶችን በመምረጥ ለቦርዱ ያቀርባል፤

12. <u>Duties and Responsibilities of the Directorate General</u>

The PPP Directorate General shall have the following responsibilities:

1/ promote the participation of the private sector in the financing, construction, maintenance and operation of Public Service Activities;

2/ provide technical assistance and support to the Board, the Ministry and the contracting authorities on all matters relating to Public Private Partnerships;

3/ make recommendations regarding the Public Private Partnership legal, regulatory, institutional and policy framework;

4/ develop and implement guidance, mandatory or otherwise, related to project preparation, procurement procedures, preparation of bid documents and Project Agreements or other topics as may be necessary to support the implementation of Public Private Partnerships in accordance with this Proclamation;

5/ conceptualize, identify and categorize projects for purposes of this Proclamation;

6/ review and issue opinions regarding the viability of proposed projects and make recommendations to the Board, the Ministry and contracting authorities;

7/ disseminate information regarding projects contemplated as potential Public Private Partnerships;

8/ select private sector candidates that may participate in public private partnerships in accordance with the procedures provided in this Proclamation and submit its recommendations to the Board;

፱/ የመንግሥት እና የግል አጋርነትን አጠቃላይ የአፈጻጸም ሂደት ይከታተላል፤

፲/ በመንግስትና በግል አጋርነት የሚከናዱ ፕሮጀቶችን በተመለከተ በቦርዱ በሚወሰን የጊዜ ልዩነት ለህዝብ ይፋ መረጃ ይሰጣል፤

፲፩/ የመንግሥት እና የግል አጋርነት ሥራዎችን ያስተባብራል።

፲፪/ በቦርዱ የሚሰጡትን ሌሎች ተግባራት ያከናውናል።

ክፍል ሦስት

የተዋዋይ ባለስልጣን ተግባርና ኃላፊነት

፲፫. ተዋዋይ ባለስልጣን

፩/ በዚህ አዋጅ ውስጥ የተመለከቱት ሌሎች ድንጋጌዎች እንደተጠበቁ ሆነው ተዋዋዩ ባለስልጣን የሚያስተዳድራቸውን የመንግሥት እና የግል አጋርነት ፕሮጀክቶችን በሚመለከት ሀሳብ የማመንጨት፣ ጥናት የማድረግ፣ ውል የመፈረምና ውሉን የማስተዳደር ጠቅላላ ኃላፊነት አለበት።

፪/ በዚህ አዋጅ መሠረት በሚወጣው መመሪያ ከሚመለከቱት በተጨማሪ ተዋዋዩ ባለስልጣን የሚከተሉት ዋና ዋና ኃላፊነቶች ይኖሩታል፦

ሀ) ሥራ ላይ ሊውሉ የታቀዱ ፕሮጀክቶችን የአዋጭነትና አማራጭ ጥናቶችን፣ የፋይናንሺያል ሞዴሎችን፣ ለገንዘብ ተመጣጣኝ ዋጋ መገኘቱን የሚያረጋግጡ ትንተናዎችን እና በቴክኒክ እና በፋይናንስ ረገድ አዋጭ የሆኑ ፕሮጀክቶችን ለመቅረጽ የሚያስችሉ ጥናቶችን ያከሂዳል፤

ለ) በዚህ አዋጅ በተደነገገው መሠረት የፕሮጀክት ሃሳቦችን ለዳይሬክተር ጀኔራሉና ለቦርዱ ያቀርባል፤

9/ monitor and evaluate the progress of implementation of Public Private Partnerships;

10/ at certain intervals, determined by the Board provide information to the public on the implementation of public private partnership projects.

11/ coordinate activities relating to Public Private Partnerships.

12/ Carry cut activities delegated to it by the Board;

CHAPTER THREE

DUTIES AND RESPONSBLTIES OF CONTRACTING AUTHORITIES

13. Contracting Authority

1/ Subject to other provisions of this Proclamation, the Contracting Authority shall have overall responsibility for the initiation, development, procurement, signing of agreements and implementation of Public Private Partnership projects it administers.

2. In addition to those specified in the directive, the Contracting Authority shall have the following detailed responsibilities:

a) Execute preparatory activities for a potential project, including all relevant feasibility and options studies, financial models, Value for Money analysis and other studies necessary to structure a technically and financially viable project;

b) Submit proposed projects to the Ministry and the Board as provided in this Proclamation;

ሐ) የአፈጻጸም ሂደቱን ለመከታተል የሚያስችለው ቴክኒካዊ ብቃት ያለው የፕሮጀክት አስተዳደር ቡድን ያቋቁማል፤

መ) ከዳይሬክቶሬት ጄኔራሉ የሚላክለትን የመንግሥትና የግል አጋርነት ውል ይፈርማል፤

ሠ) ሚኒስቴሩ፣ ቦርዱ እና የመንግሥት እና የግል አጋርነት ዳይሬክቶሬት ጄኔራል በሚጠይቁበት ወቅት ተገቢውን መረጃ ይሰጣል፤

ረ) በፕሮጀክት ስምምነቱ በሌላ ሁኔታ ካልተመለከተ በቀር የግል ባለሀብቱ በፕሮጀክት ስምምነቱ መሠረት መፈጸሙን ይቆጣጠራል።

፲፬. በተዋዋይ ባለስልጣን ሥር ስለሚገኘው የፕሮጀክት አስተዳደር ቡድን

፩/ የመንግሥት እና የግል አጋርነት ውል ለመፈጸም የሚያቅድ ተዋዋይ ባለስልጣን በሚያስፈልገው እና ለእያንዳንዱ ፕሮጀክት አስፈላጊ ይሆናል በሚለው መጠን የፋይናንስ፣ የቴክኒክ እና የሕግ ባለሙያዎችን ያቀፈ የፕሮጀክት አስተዳደር ቡድን ማቋቋም ይኖርበታል። የፕሮጀክት አስተዳደር ቡድኑ የመንግሥት እና የግል አጋርነት ዳይሬክቶሬት ጄኔራል ተወካይን በአባልነት ያካተተ ሊሆን ይገባል፤

፪/ የፕሮጀክት አስተዳደር ቡድኑ ተዋዋይ አካሉን በመወከል፦

ሀ) የመንግሥት እና የግል አጋርነት ፕሮጀክቶችን የሕግ፣ የቁጥጥር፣ የማህበራዊ፣ የፋይናንስ፣ የኢኮኖሚ እና የንግድ አዋጭነት ለማረጋገጥ የመንግስትና የግል አጋርነት ፕሮጀክቶችን ያዘጋጃል፣ ይገመግማል፤

ለ) በመግአ ዳይሬክቶሬት ጄኔራል የተዘጋጁ መመሪያዎችንና መደበኛ ሰነዶችን መሠረት በማድረግ ፕሮጀክቶችን ይቀርጻል፤

c) Establish a technically qualified project management team to oversee the transaction process;

d) Sign public private partnership agreements referred to it by the PPP Directorate General;

e) Provide information, as reasonably requested, by the Ministry, the Board and the PPP Directorate General;

f) Unless otherwise stipulated in the Project Agreements, supervise the Private Party and ensure its strict compliance with the terms of the Project Agreements.

14. Project Management Team within the Contracting Authority

1/ A Contracting Authority that intends to enter into a Public Private Partnership shall establish a project management team dedicated to a particular project which shall consist of such financial, technical and legal personnel as that Contracting Authority shall consider necessary for the performance of its functions. The project management team shall include a member of the PPP Directorate General.

2/ The project management team shall, on behalf of the Contracting Authority:

a) prepare and appraise the Public Private Partnership project to ensure its, legal, regulatory, social, financial, economic and commercial viability;

b) prepare the project in accordance with guidelines and standard documents issued by the PPP Directorate General;

ሐ) ተዋዋይ ባለሥልጣን እና የግል ባለሀብቱ ይህን አዋጅ መሠረት በማድረግ ስራውን ማከናወኑን ያረጋግጣል፤

መ) ከተዋዋይ ባለስልጣን ጋር የተገባው የመንግስ ስምምነት በትክክል ተግባራዊ መሆኑን ይከታተላል፤

ሠ) በፕሮጀክቱ አፈጻጸም ወቅት ከባለ ድርሻ አካላት ጋር ግንኙነት ያደርጋል፤

ረ) ከተዋዋይ ባለስልጣን ጋር በተገባው የፕሮጀክት ስምምነት መሠረት የፕሮጀክቱን አመራር ይከታተላል፤

ሰ) ተዋዋይ ባለሥልጣኑ ያደረገውን የፕሮጀክት ስምምነት አፈጻጸም አስመልክቶ የመንግ ዳይሬክቶሬት ጄኔራል በሚጠይቀው መሠረት በየዓመቱ ወይም በሌላ የጊዜ ወሰን ለመንግሥት እና ለግል አጋርነት ዳይሬክቶሬት ጄኔራል ሪፖርት ያቀርባል፤

ሸ) ተዋዋይ ባለስልጣን የመንግሥት እና የግል አጋርነት ፕሮጀክቶችን አስመልክቶ የሚፈጽማቸውን ስምምነቶችና ሌሎች ማናቸውንም ሰነዶች ይይዛል፤

ቀ) የመንግሥት እና የግል አጋርነት ስምምነቶች የንብረት መተላለፍን የሚመለከቱ ድንጋጌዎችን የያዙ በሚሆንበት ጊዜ ስምምነቶቹ ሲጠናቀቁ ወይም ከውሉ መጠናቀቂያ ጊዜ አስቀድሞ በሚቋረጡበት ወቅት በስምምነቱ መሠረት ንብረቶች መተላለፋቸውን ያረጋግጣል፤

በ) በተዋዋዩ ባለስልጣን በሚወስነው መሠረት ሌሎች ተግባሮች ያከናውናል።

፫/ የፕሮጀክት አስተዳደር ቡድኑ በዚህ አንቀጽ ንዑስ አንቀጽ (፪) ላይ የተጠቀሱትን ተግባሮች በሚያከናውንበት ወቅት፦

c) ensure that the Private Party comply with the provisions of this Proclamation;

d) monitor the implementation of the Public Private Partnership Agreement entered into with the Contracting Authority;

e) liaise with all key stakeholders during the project cycle;

f) oversee the management of a project in accordance with the Project Agreements entered into by the Contracting Authority;

g) submit to the PPP Directorate General, annual or such other period reports requested by the PPP Directorate General on the Project Agreements entered into by the Contracting Authority;

h) maintain a record of all documentation and agreements entered into by the Contracting Authority relating to the Public Private Partnership project;

i) ensure that the transfer of assets at the expiry or early termination of a Public Private Partnership Agreement is consistent with the terms and conditions of such agreement, where this agreement involves a transfer of assets; and

j) carry out such other functions as may be assigned to it by the Contracting Authority.

3/ In performing its functions under sub-article (2) of this Article, a project management team shall:

ሀ) የመንግሥት እና የግል አጋርነት ዳይሬክቶሬት ጄኔራል የሚያቀርባቸውን ሃሳቦችና መመሪያዎች ስራ ላይ ያውላል፤

ለ) የመንግሥት እና የግልአጋርነት ዳይሬክቶሬት ጄኔራል ወይም የሚኒስቴሩ ሌሎች ክፍሎች የሚጠይቋቸውን መረጃዎች ይሰጣል፤

ክፍል አራት
የመንግሥት እና የግል አጋርነት ፕሮጀክት ስለማዘጋጀት እና የማፀደቅ ሂደት

፲፭. ፕሮጀክቶችን ስለመለየት

፩/ የመንግስት እና የግል አጋርነት ፕሮጀክቶችን የማመንጨት ሥራ በተዋዋይ ባለስልጣን፣ በመንግሥት ተቋም ወይም በመንግሥት እና በግል አጋርነት ዳይሬክቶሬት ጄኔራል ሊካሄድ ይችላል።

፪/ ፕሮጀክቶችን በመንግሥት እና በግል አጋርነት አማካኝነት ለማከናወን የሚፈልግ ተዋዋይ ባለስልጣን በሌሎች አንቀጾች የተደነገገው እንደተጠበቀ ሆኖ ፕሮጀክቱን ከመቅረጽ አንስቶ የውሉን አስተዳደር የማስፈፀም ኃላፊነት አለበት።

፫/ ተዋዋይ ባለስልጣን ፕሮጀክቶችን የመቅረጽ፣ የመለየትና ቅደም ተከተል የማስያዝ ስራ በሚያከናውንበት ወቅት የተቋሙ ግንባታ ወይም አገልግሎት የመስጠቱ ተግባር በተዋዋዩ ባለስልጣን ከሚከናወን ይልቅ በመንግሥትና የግል አጋርነት ቢከናወን ለመንግሥት ገንዘብ ሊገኝ የሚችለውን ተመጣጣኝ ዋጋ፣ ስትራቴጂካዊና የስራ አመራር ጠቀሜታ ከግምት ውስጥ ማስገባት አለበት።

፲፮. በቦርዱ ስለሚሰጥ የቅድሚያ ፈቃድ

ተዋዋይ ባለስልጣን ፕሮጀክቱን በመንግሥት እና በግል አጋርነት አማካይነት ሥራ ላይ ሊያውል በሚያቅድበት ወቅት ጥልቅ ጥናት ከማድረጉ አስቀድሞ ፕሮጀክቱን እንዲያጸድቅለት ለሚኒስቴሩ ጥያቄ ማቅረብ አለበት።

a) Implement the recommendations and guidelines issued by the PPP Directorate General;

b) Submit such information as shall be required by the PPP Directorate General or other Directorates of the Ministry.

CHAPTER FOUR
PUBLIC PRIVATE PARTNERSHIP PROJECT DEVELOPMENT AND APPROVAL PROCESS

15. Project identification

1/The identification of potential Public Private Partnership projects shall be carried out by the Contracting Authority, a Public Entity or the PPP Directorate General.

2/ A Contracting Authority which intends to implement a project through a Public Private Partnership shall be responsible for conceptualizing and administering the contract of the project.

3/ In conceptualizing, identifying and prioritizing potential projects, a Contracting Authority shall consider the strategic and operational benefits of entering into a Public Private Partnership compared to the development of the facility or the provision of the service by the Contracting Authority itself.

16. Prelminiary Approval of the Board

Where a Contracting Authority considers it appropriate to potentially implement a project through a Public Private Partnership, it shall submit an application to the Ministry for approval before undertaking an in-depth analysis of the project under a Public Private Partnership scheme.

፲፯. የአዋጭነት ጥናት

፩/ ፕሮጀክቱ በሚኒስቴሩ ከጸደቀ በኋላ ተዋዋዩ ባለስልጣን ከዚህ ቀጥሎ ለተመለከቱት ዓላማዎች የአዋጭነት ጥናት ማካሄድ ይኖርበታል፦

ሀ) ፕሮጀክቱ ተግባራዊ ሊሆን የሚችል መሆኑን ለማረጋገጥ፤ እና

ለ) የመንግሥት እና የግል አጋርነት አማራጭ ለተዋዋዩ ባለስልጣን ከሁሉም የተሻለ ኢኮኖሚያዊ ጠቀሜታ የሚያስገኝ አማራጭ መሆኑን ለማረጋገጥ።

፪/ በዚህ አንቀጽ በንዑስ አንቀጽ (፩) ላይ የተመለከተውን የአዋጭነት ጥናት በሚያካሂድበት ወቅት ተዋዋዩ ባለስልጣን የሚከተሉትን ከግምት ውስጥ ማስገባት ይኖርበታል፦

ሀ) ለፕሮጀክቱ የሚያስፈልጉ ቴክኒካዊ ሁኔታዎችን፤

ለ) የፕሮጀክቱ ተዋዋይ ወገኖች ሊያሟሏቸው የሚገባ የሕግ ሁኔታዎችን፤

ሐ) የፕሮጀክቱን ማኅበራዊ፣ ኢኮኖሚያዊና አካባቢያዊ እንድምታዎች፤

መ) ከሌሎች የግዥ አማራጮች ጋር ሲነጻጸር ፕሮጀክቱ ለገንዘብ ተመጣጣኝ ዋጋ የሚያስገኝ መሆኑን፤

ሠ) የፕሮጀክቱ ወጭ በተዋዋዩ ባለስልጣን ወይም በተጠቃሚዎች ሊሸፈን የሚችል መሆኑን፤

ረ) ፕሮጀክቱ ለተዋዋይ ወገኖች ዘላቂነት ጠቀሜታ የሚያስገኝ መሆኑን፤

ሰ) ተዋዋዩ ባለስልጣን ፕሮጀክቱን ለማዘጋጀት፣ ጨረታውን ለማካሄድ፣ ፕሮጀክቱን ሥራ ላይ ለማዋልና ለመከታተል ድርጅታዊ አቅም ያለው ስለመሆኑ።

፫/ የፕሮጀክቱ የአዋጭነት ጥናት በዚህ አንቀጽ ንዑስ አንቀጽ (፪) ላይ የተመለከተውን ትንተና ከግምት ውስጥ በማስገባት ከዚህ በታች የተጠቀሱትን ጨምሮ የፕሮጀክቱን ታሳቢ ቅርጽ ማሳየት ይኖርበታል፦

17. Feasibility Study

1/ Upon approval by the Ministry, the Contracting Authority shall undertake a feasibility study of the project for the purpose of:

a) determining the viability of the project; and

b) ensuring that a Public Private Partnership approach is the most economically advantageous to the Contracting Authority.

2/ In undertaking a feasibility study under Sub-Article (1) of this Article, a Contracting Authority shall consider:

a) the technical requirement of the project;

b) the legal requirement to be met by the parties to the project;

c) the social, economic and environmental impact of the project;

d) the Value for Money of the project compared to other procurement method;

e/ the affordability of the project by the Contracting Authority and/or end-users;

f/ the sustainability of the project for both parties;

g/ the institutional capacity of the Contracting Authority to prepare, tender, implement and monitor the project.

3/ Taking into account the analysis carried out pursuant to sub-article (2) of this Article, the feasibility study shall describe the proposed structure for the project including:

ሀ) በተለያዩ አማራጮች ለፕሮጀክቱ የሚያስፈልገውን ካፒታልና የሥራ ማስኬጃ ወጭ፤

ለ) ተዋዋዩ ባለስልጣን እና የግል ባለሀብቱ በየድርሻቸው የሚኖርባቸውን ኃላፊነት የሚቀበሉዋቸውን የስጋት ዓይነቶች፤

ሐ) አስፈላጊ ሆኖ በተገኘ ጊዜ የመንግሥትን ተሳትፎና ድጋፍ።

፲፰. በቦርድ ስለማጽደቅ

፩/ ተዋዋዩ ባለስልጣን በዚህ አዋጅ አንቀጽ ፲፯ መሠረት ያዘጋጀውን የአዋጭነት ጥናት ለቦርዱ እና ለሚኒስቴሩ ያቀርባል።

፪/ ቦርዱ የመንግሥት እና የግል አጋርነት ዳይሬክቶሬት ጀኔራል በሚያደርግለት ድጋፍ ታግዞ የአዋጭነት ጥናቱን ይመረምራል። ቦርዱ ጥናቱን በሚመረምርበት ወቅት ስለዕዳ አስተዳደርና ዋስትና ጉዳይ ኃላፊነት ያለበት አግባብ ያለው የሚኒስቴሩ የሥራ ክፍል ያደረገውን ግምገማ ከግምት ውስጥ ማስገባት የሚኖርበት ሲሆን የሚሠጠውም ውሳኔ ከዚህ ግምገማ ጋር የሚጣጣም መሆኑን ማረጋገጥ ይኖርበታል።

፫/ ቦርዱ ፕሮጀክቱ ወደ ጨረታ ሂደት እንዲገባ ስለመፈቀዱ ውሳኔ ከማስተላለፉ በፊት ተጨማሪ ጥናቶች በመንግሥት እና በግል አጋርነት ዳይሬክቶሬት ጀኔራል ወይም በተዋዋዩ ባለስልጣን እንዲካሄድ ለማዘዝ ይችላል።

፬/ ፕሮጀክቱ የመንግሥት ድጋፍ ወይም ዋስትና የሚያስፈልገው ከሆነ ቦርዱ ከአስተያየቱ ጋር ጉዳዩን ለሚመለከተው አካል ይልካል።

፭/ ፕሮጀክቱ የሚፈለገውን የአዋጭነት መስፈርት የሚያሟላ እና ለገንዘብ ተመጣጣኝ ዋጋ የሚያስገኝ መሆኑን ከረጋገጠ ቦርዱ ወደ ጨረታ ሂደት እንዲገባ ለመፍቀድ ይችላል።

a) project capital and operating costs under various scenarios;

b) proposed responsibilities and risks assumed by each the Contracting Authority and the Private Party;

c) proposed government participation and support, if any.

18. Approval of the Board

1/ The Contracting Authority shall submit the feasibility study prepared under Article 17 to the Board and the Ministry.

2/ The Board shall review the feasibility study with the support of the PPP Directorate General. The Board shall in its review take into account the assessment of the departments of the Ministry responsible for debt management and guarantees and take a decision that complies with this assessment.

3/ The Board may require additional studies be carried out either by the PPP Directorate General or the Contracting Authority prior to issuing a determination as to whether the project is authorized to proceed to tender.

4/ If specific incentives or government support is required, the Board shall with its suggestion submit the matter to the appropriate authority.

5/ If the Board is satisfied that the project meets the desired expectations of viability and Value for Money, the Board may authorize to proceed to tender the project.

ክፍል አምስት

የግል ባለሀብቱን ስለመምረጥ

፲፱. በግልጽ ጨረታ ዘዴ ባለሀብትን ስለመምረጥ

፩/ በዚህ አዋጅ በሌላ ሁኔታ ካልተደነገገ በስተቀር ፕሮጀክቶች ለግል ባለሀብት የሚሰጡት የቅድመ ብቃት ማረጋገጫን ባካተተ የግልጽ ጨረታ ዘዴ ብቻ ነው።

፪/ የመንግሥት እና የግል አጋርነት የግዥ ሂደትን በማከናወንና አሸናፊውን ባለሀብት በመምረጥ ሂደት የመግአ ዳይሬክቶሬት ጄኔራል በግልጽ፣ ነጻ እና ፍትሀዊ ውድድር እንዲሁም ለሁሉም እኩል ዕድል በመስጠት መርህ መመራት ይኖርበታል።

፳. የቅድመ-ብቃት ማረጋገጫ

፩/ በዚህ አዋጅ በአንቀጽ (፲፰) መሠረት ቦርዱ ጥናቱን ካፀደቀው የመግአ ዳይሬክቶሬት ጄኔራል ፕሮጀክቱን በአስተማማኝ ሁኔታ ተግባራዊ ሊያደርጉ የሚችሉ ብቁ ተጫራቾችን ለመለየት የሚያስችለውን የቅድመ-ብቃት ማረጋገጫ ሥራ ማከናወን ይኖርበታል።

፪/ የመግአ ዳይሬክቶሬት ጄኔራል ለመጫረት የሚችሉ ተወዳዳሪዎች በቅድመ-ብቃት ማረጋገጥ ሂደት ውስጥ ተሳታፊ እንዲሆኑ፦

ሀ) ሰፊ ሽፋን ባለው ጋዜጣ የጥሪ ማስታወቂያ ማውጣት ይኖርበታል።

ለ) ዝርዝሩ በሚያወጣው መመሪያ የሚወሰን ሆኖ፣ ከጋዜጣ ጥሪ በተጨማሪ ለመጫረት እንደሚችሉ ለይቶ ለሚያውቃቸው ተወዳዳሪዎች ጥሪውን በቀጥታ መላክ ይችላል።

፫/ ተዳዳሪዎች በቅድመ ብቃት ማረጋገጥ ሂደት ውስጥ ተሳታፊ የሚሆኑበት አሰራር በመመሪያ ይወሰናል።

CHAPTER FIVE

SELECTION OF PRIVATE PARTY

19. Procurement Through Open Bidding Process

1/ Except as otherwise provided for under this Proclamation, all projects shall be procured through an open bidding process with prequalification.

2/ In procuring and awarding a Public Private Partnership Agreement to a Private Party, the Directorate General shall be guided by the principles of transparency, free and fair competition and equal opportunity.

20. Prequalification

1/ Upon approval of the Board pursuant to Article (18) the Directorate General shall engage in prequalification proceedings with a view to identifying bidders that are suitably qualified to successfully implement the project.

2/ The Directorate General shall issue a notice of request for qualifications:

a) in a widely circulated publication inviting potential bidders to participate in a prequalification proceeding ;

b) in addition to publication in a newspaper the content of which is to be determined in a directive, may send the request for qualifications to potential bidders identified by the Directorate General.

3/ The concents of such request shall be detetrmined by directive.

፳፩. የቅድመ-ብቃት ማረጋገጫ መስፈርት

፩/ ለቅድመ-ብቃቱ ማረጋገጫ ሂደት ብቁ ሆኖ ለመገኘት ፍላጎት ያላቸው ተጫራቾች የመግለ ዳይሬክቶሬት ጀኔራል በጥሪው ማስታወቂያ ለእያንዳንዱ ፕሮጀክት አግባብ ነው ብሎ ያስቀመጣቸውን መመዘኛዎች በተጨባጭ ሁኔታ የሚያሟሉ መሆናቸውን ማረጋገጥ አለባቸው።

፪/ በዝቅተኛ ደረጃ ሊሟሉ የሚገባቸውን የቅድመ ብቃት ማረጋገጫ መመዘኛዎች ለመወሰን የመንግሥትና የግል አጋርነት ዳይሬክቶሬት ጀኔራል ለቅድመ ብቃት የሚወዳደሩ ተጫራቾች፦

ሀ) የተጣራ ሀብትን ወይም ፋይናንስ አቅምን ለመመዘን በሚያስችል መለኪያ በመጠቀም ፕሮጀክቱን ለመፈፀም የሚያስችል በቂ የፋይናንስ አቅም፤ እና

ለ) በተመሳሳይ ሥራ ላይ ቀዳሚ የሥራ ልምድን በቴክኒክ ብቃት መመዘኛነት በመጠቀም ፕሮጀክቱን ለመፈፀም የሚያስችል በቂ የቴክኒክ ያላቸው መሆኑን ያረጋግጣል።

፫/ በቅድመ ብቃት ማረጋገጫ ሂደት አስፈላጊ ናቸው የሚባሉ በዝቅተኛ ደረጃ ሊሟሉ የሚገባቸው መመዘኛዎች በዝርዝር መመሪያ ይወሰናል።

፳፪. በኅብረት ስለመሳተፍ

፩/ የመግለ ዳይሬክቶሬት ጀኔራል ተጫራቾችን ለቅድመ-ብቃት ማረጋገጫ ሲጋብዝ በኅብረት ሆነው ለመሳተፍ እንዲችሉ መፍቀድ አለበት፤ በቅድመ-ብቃት ማረጋገጫው ጨረታ ሂደት ከተጫራቾች የሚጠየቀው መረጃ ኅብረቱን እንደኅብረት እና የኅብረቱን አባላት በተናጠል የሚያካትት መሆን አለበት።

፪/ በቅድመ-ብቃት ማረጋገጫው ማስታወቂያ በተለየ ሁኔታ ካልተገለጸ በስተቀር፣ አንድ የኅብረት አባል በቀጥታም ሆነ በተዘዋዋሪ አባል

21. Prequalification criteria

1/ In order to qualify for the pre-selection proceedings, interested bidders must meet objectively justifiable criteria that the Directorate General considers appropriate in the particular proceedings of the project, as stated in the request for qualifications.

2/ In determining the minimum prequalification criteria, the PPP Directorate Generel shall enuser that prequalified bidders demonstrate:

a) Sufficient financial capacity to undertake the project,through evidence of net worth or other appropatiate measure ; and

b) Sufficient technical capacity to undertake the project, through evidence previous appropriate experience.

3/ The details of the minimum prequalification criteria shall be further prescribed by the relevant directive.

22. Participation of consortia

1/ The Directorate General, when first inviting the participation of bidders in the prequalification proceedings, shall allow them to form bidding consortia. The information required in the request for qualifications from members of bidding consortia shall relate to the consortium as a whole as well as to its individual participants.

2/ Unless stated in the request for qualifications, each member of a consortium may participate, either

መሆን የሚችለው በአንድ ኅብረት ብቻ ነው። ይህን ድንጋጌ መጣስ የኅብረቱ አባልም ሆነ ኅብረቱ ከቅድመ-ብቃት ማረጋገጫ ሂደት እንዲሰረዙ የማድረግ ውጤት ይኖረዋል።

፫/ የመግአ ዳይሬክቶሬት ጄኔራል የሕብረቱን ብቃት ሲገመግም እያንዳንዱ የኅብረቱ አባል ያለውን ብቃት እና አባላቱ ተቀናጅተው ሁሉም የፕሮጀክቱ ደረጃዎች የሚፈልጉትን ብቃት የሚያሟሉ መሆኑን ማረጋገጥ አለበት።

፳፫. <u>በቅድመ-ብቃት ማረጋገጫ ላይ ስለሚሰጥ ውሳኔ</u>

፩/ የመግአ ዳይሬክቶሬት ጄኔራሉ፦

ሀ) ለቅድመ-ብቃት ማረጋገጫ ያመለከተውን የእያንዳንዱን ተጫራች ብቃት በሚመለከት ውሳኔ ይሰጣል።

ለ) ይህን ውሳኔ የሚሰጠው በጥሪ ማስታወቂያው ላይ ያስቀመጣቸውን መመዘኛዎች በመጠቀም ይሆናል።

ሐ) በቅድመ-ብቃት ማረጋገጫ የተመረጡ ተጫራቾች የመጫረቻ ሰነድ እንዲያስገቡ ጥሪ ሊያደርግላቸው ይገባል።

፪/ በዚህ አንቀጽ ንዑስ አንቀጽ (፩) የተመለከተው ድንጋጌ ቢኖርም በጥሪ ማስታወቂያው ላይ ይህን የተመለከተ መረጃ እስከሰጠ ድረስ የመግአ ዳይሬክቶሬት ጄኔራል የቅድመ ብቃት ማረጋገጫውን ሂደት ካጠናቀቀ በኋላ የቅድመ-ብቃቱን መመዘኛዎች ካሟሉት ተጫራቾች መካከል የተወሰኑት ብቻ የመጫረቻ ሰነድ እንዲያቀርቡ የመጠየቅ መብት አለው።

፫/ በዚህ አንቀጽ በንዑስ አንቀጽ (፪) ላይ የተመለከተውን ለማስፈጸም እንዲረዳ የመግአ ዳይሬክቶሬት ጄኔራል የተጫራቾቹን ብቃት

directly or indirectly, in only one consortium at the same time. A violation of this rule shall cause the disqualification of the consortium and of the individual members.

3/ When considering the qualifications of bidding consortia, the PPP Directorate General shall consider the capabilities of each of the consortium members and assess whether the combined qualifications of the consortium members are adequate to meet the needs of all phases of the project.

23. <u>Decision on prequalification</u>

1/ The PPP Directorate General shall:

a) make a decision with respect to the qualifications of each bidder that has submitted an application for prequalification.

b) shall apply only the criteria set forth in the request for qualifications.

c) Invite all prequalified bidders to submit their bids.

2/ Notwithstanding Sub-Article (1) of this Article, the PPP Directorate General may, provided that it has made an appropriate statement in the request for qualifications to that effect, reserve the right to request proposals upon completion of the prequalification proceedings only from a limited number of bidders that best meet the prequalification criteria.

3/ For the purpose of Sub-Article (2) of this Article, the Directorate General shall rate the bidders that meet the

ለመመዘን በቅድመ-ብቃት ማረጋገጫው ሂደት የተቀመጡትን መመዘኛዎች ተጠቅሞ የተወዳዳሪዎቹን ደረጃ በመመዘን ከቅድመ-ብቃት ማረጋገጫው በኋላ የመጫረቻ ሰነድ ሊያቀርቡ የሚችሉትን በቅደም ተከተል ያስቀምጣል፡፡

፬/ የመግአ ዳይሬክቶሬት ጄኔራል ይህን የቅደም ተከተል ደረጃ በሚወስንበት ወቅት የቅድመ-ብቃት ማረጋገጫ ጥሪ ባደረገበት ማስታወቂያ ላይ ያመለከታቸውን የመመዘኛ ነጥቦች መጠቀም አለበት፡፡

፳፬. የመወዳደሪያ ሀሳብ ስለመጠየቅ

፩/ የመግአ ዳይሬክቶሬት ጄኔራል ተጫራቾች የመወዳደሪያ ሀሳብ እንዲያቀርቡ የሚያስችል የመወዳደሪያ ሀሳብ መጠየቂያ ሰነድ ማዘጋጀት አለበት፡፡

፪/ የመወዳደሪያ ሀሳብ መጠየቂያው ቢያንስ ከዚህ በታች የተመለከቱትን መረጃዎች መያዝ ይኖርበታል፦

ሀ) የመወዳደሪያ ሀሳብ ለማዘጋጀት እና ለማቅረብ የሚረዳ ስለ ፕሮጀክቱ አጠቃላይ መረጃ የሚሰጥ መግለጫ፤

ለ) ተጫራቹ ሊያሟላቸው የሚገባ የቴክኒክና ፋይናንሺያል ሁኔታዎችን ያካተተ የፕሮጀክቱ ፍላጎት ዝርዝር መግለጫ፤

ሐ) የአካባቢ ጥበቃና ደህንነት ሁኔታዎችን ጨምሮ ተጫራቹ ሊያሟላቸው የሚገቡ የመግአ ዳይሬክቶሬት ጄኔራል እና አግባብነት ያላቸው ተቆጣጣሪ አካላት አስፈላጊ ናቸው ብለው የሚያምኑባቸው የመጨረሻው የምርት ውጤት ዝርዝር መግለጫ፣ የአገልግሎት ጥራት ደረጃ፣ የአፈጻጸም መለኪያዎች እንዲሁም ሌሎች መሥፈርቶች፤

prequalification criteria on the basis of the criteria applied to assess their qualifications and draw up the list of bidders that will be invited to submit proposals upon completion of the prequalification proceedings.

4/ In drawing up the list, the Directorate General shall apply only the manner of rating that is set forth in the request for qualifications.

24. Request for Proposals

1/ The PPP Directorate General shall prepare a request for proposals for the purpose of inviting bidders to submit proposals.

2/ The details of the request for proposals shall include at least the following information:

a) General information related to the project necessary for the preparation and submission of a proposal;

b) specifications of the project including the technical and financial conditions that should be met by a bidder;

c) specifications of the final product, level of services, performance indicators and such other requirements as the Directorate General and relevant regulatory bodies shall consider necessary including the safety, security and environment preservation require ments to be met by a bidder;

መ) የመግአ ዳይሬክቶሬት ጄኔራል በፕሮጀክት ስምምነቱ ውስጥ እንዲካተቱለት የሚያቀርባቸው የውል ሁኔታዎች፤

ሠ) የመወዳደሪያ ሀሳቡን ለመገምገም ጥቅም ላይ የሚውሉ መመዘኛዎች እና ዘዴዎች፤

ረ) ተጫራቹ እንዲያቀርብ የሚፈለገው የጨረታ ማስከበሪያ ገንዘብ መጠን፤ እና

ሰ) የመወዳደሪያ ሃሳቡ የሚቀርብበት ሁኔታና የጊዜ ገደብ፡፡

፫/ ሚኒስትሩ በሚያወጣው መመሪያ የመወዳደሪያ ሀሳብ ለመገምገም የሚያገለግሉ ተጨማሪ መስፈርቶችን ሊወስን ይችላል፡፡

፳፭. የጨረታ ማስከበሪያ

፩/ የጨረታ ማስታወቂያው የጨረታ ማስከበሪያው ከየት መቅረብ እንዳለበት እና ስለ ጨረታ ማስከበሪያው ዓይነት፣ ቅርጽ፣ መጠን እና ሌሎች ዋና ዋና ሁኔታዎች እና ቅድመ-ሁኔታዎች የሚገልጽ ሆኖ መዘጋጀት ይኖርበታል፡፡

፪/ የጨረታ ማስከበሪያው መጠን እና የሚቀርብበት አኳኋን ወይም የሚወረስበት ሁኔታ እና ሌሎች ተያያዥነት ያላቸው ጉዳዮች በመመሪያ ይወሰናሉ፡፡

፳፮. ስብሰባዎች፣ ማብራሪያዎችና ማሻሻያዎች

የመግአ ዳይሬክቶሬት ጄኔራል፦

፩/ ለተጫራቾች ስለፕሮጀክቱ መረጃ ለመስጠት፣ ለጥያቄዎቻቸው መልስ ለመስጠት እና ከተጫራቾች የሚቀርቡ ሃሳቦችን ለመቀበል የሚያስችለውን የአንድ ለአንድ ወይም የቡድን ስብሰባ የቅድመ-ብቃት ማረጋገጫውን ደረጃ ካለፉ ተጫራቾች ጋር ለማካሄድ ይችላል፤

፪/ ለማናቸውም አንድ ተጫራች የተሰጠ መረጃ ለሁሉም የቅድመ-ብቃት ማረጋገጫ ደረጃውን ላለፉ ተጫራቾች በጽሁፍ እንዲደርሳቸው መደረግ አለበት፡፡

d) the contractual terms of the Project Agreements proposed by the PPP Directorate General;

e) the criteria and method to be used in evaluating a proposal;

f) the value of the bid security required to be submitted by a bidder; and

g) the manner and the deadline for submitting proposals.

3/ The directive may prescribe additional requirements of the request for proposals.

25. Bid securities

1/ The request for proposals shall set forth the requirements with respect to the issuer and the nature, form, amount and other principal terms and conditions of the required bid security.

2/ The amount and the details of the ways a bid security is to be submitted or forfeited as the case may be and other related matters shall be prescribed by directive.

26. Meetings, clarifications and modifications

The PPP Directorate General:

1/ may hold one-on-one or group meetings with prequalified bidders to provide information on the project, respond to their enquiries and receive comments from the bidders.

2/ Shall communicate in writing to all bidders any information provided to a bidder.

፫/ የቅድመ-ብቃት ደረጃውን ያለፉ ተጫራቾች በጨረታ ማስታወቂያው ላይና እንዲሁም በፕሮጀክት ስምምነቱ አንቀጾች ላይ ሃሳባቸውን እንዲሰጡበት ሊጋብዙ ይችላሉ።

፬/ የቅድመ-ብቃት ደረጃውን ካለፉ ተጫራቾች ለሚቀርቡለት የማብራሪያ ጥያቄዎች ምላሽ መስጠት ይኖርበታል።

፭/ በራሱ ተነሳሽነት ወይም የቅድመ-ብቃት ማረጋገጫ ደረጃውን ካለፉ ተጫራቾች በሚቀርቡለት አስተያየቶች ወይም የማብራሪያ ጥያቄዎች በመነሳት የፕሮጀክት ስምምነቱን ጨምሮ የመወዳደሪያ ሃሳብ መጠየቂያውን ጥሪ እንደገና ለመመርመር ወይም እንደአግባብነቱ ለመከለስ ይችላል።

፮/ ማናቸውም በዚህ አንቀጽ (፭) መሠረት የሚደረግ ማሻሻያ የመጫረቻ ሰነድ የሚቀርብበት የጊዜ ገደብ ከመድረሱ ከበቂ ጊዜ በፊት በጽሑፍ ለሁሉም ተጫራቾች ማድረስ ይኖርበታል።

፳፯. የመወዳደሪያ ሀሳብ ስለማቅረብ

ለፕሮጀክት ሥራ መጫረት የሚፈልግ ማናቸውም ተጫራች የቴክኒክና የፋይናንሺያል የመወዳደሪያ ሃሳቦችን አዘጋጅቶ ማቅረብ የሚኖርበት ሲሆን በመወዳደሪያ ሃሳብ መጠየቂያ ጥሪ ላይ በሚመለከተው መሠረት የቴክኒክና የፋይናንሺያል የመወዳደሪያ ሀሳቦቹን በተለያዩ ኤንቬሎፖች በማሸግ ማቅረብ ይኖርበታል።

፳፰. የመወዳደሪያ ሀሳቦችን ስለመገምገም

፩/ በመወዳደሪያ ሀሳብ መጠየቂያው ሰነድ በሌላ ሁኔታ ካልተመለከተ በስተቀር የመግአ ዳይሬክቶሬት ጀኔራል ቴክኒካል ሰነዱን ከፍቶ የጨረታውን መስፈርት የሚያሟላ መሆኑን እስካላረጋገጠ ድረስ የመጫረቻ ዋጋውን መክፈት አይችልም።

፪/ የመግአ ዳይሬክቶሬት ጀኔራል እያንዳንዱን ሰነድ በግምገማ መስፈርቱ ያለም ከሆነ ለእያንዳንዱ መስፈርት ተፈፃሚ የሚሆን የነጥብ

3/ may invite prequalified bidders to provide comments on the request for proposals including on the terms of the Project Agreements.

4/ shall respond to request for clarification from prequalified bidders.

5/ may, whether on its own initiative or as a result of a comment or request for clarification of a bidder, review and, as appropriate, revise any element of the request for proposals, including the Project Agreements.

6/ shall communicate in writing any revision made subject to sub-article (5)of this to the bidders at a reasonable time prior to the deadline for submission of proposals.

27. Submission of proposals

A bidder who intends to bid for a project shall complete and submit a technical proposal and a financial proposal and enclose each proposal in a separate sealed envelope as may be specified in the request for proposals.

28. Evaluation of proposals

1/ Unless provided otherwise in the request for proposals, the PPP Directorate General shall not open the financial proposal unless it has opened and deemed the technical proposal to be responsive.

2/ The PPP Directorate General shall compare and evaluate each proposal in accordance with the evaluation criteria, the relative weight accorded to each

ክብደት እና በመወዳደሪያ ሀሳብ መጠየቂያው ሰነድ በተመለከተው የግምገማ ሂደት መሰረት ይገመገማል።

፫/ ለዚህ አንቀጽ ንዑስ አንቀጽ (፪) ድንጋጌ አፈጻጸም የመግአ ዳይሬክቶሬት ጄኔራል ጥራትን፣ ቴክኒክን፣ የመጫረቻ ዋጋን እና የገበያ ሁኔታን አስመልክቶ መሟላት ያለበትን ዝቅተኛ መለኪያ ሊወስን ይችላል። የወጣውን ዝቅተኛ መለኪያ የማያሟሉ የመወዳደሪያ ሀሳቦች እንዳልተሟሉ ተቆጥረው ከምርጫ ሥነ ሥርዓቱ እንዲሰረዙ ይደረጋል።

፬/ የመግአ ዳይሬክቶሬት ጄኔራል የአገር ውስጥ ተጫራቾችን ተሳትፎ ለመደገፍ ለዚህ አዋጅ አፈጻጸም በሚወጣው መመሪያ መሠረት ልዩ አስተያየት ሊያደርግ ይችላል።

፳፱. የግምገማ ሪፖርት

፩/ የመግአ ዳይሬክቶሬት ጄኔራል የመወዳደሪያ ሀሳቦችን የመገምገሙን ስራ እንዳጠናቀቀ እና አሸናፊውን ተጫራች ሳያስታውቅ በፊት የምዘና መስፈርቱን፣ከሌሎች ተጫራቾች ጋር ሲወዳደር አሸናፊው በመወዳደሪያ ሀሳብ መጠየቂያው የተመለከቱ የምዘና መስፈርቶችን በተሻለ ሁኔታ የሚያሟላ ሆኖ የተገኘበትን ምክንያት እና ሌሎች የመግአ ዳይሬክቶሬት ጄኔራል አስፈላጊ ናቸው የሚላቸውን መረጃዎች የሚገልፅ የግምገማ ሪፖርት ማዘጋጀት አለበት።

፪/ በዚህ አዋጅ አንቀጽ (፴፩) በተመለከተው መሠረት ከአሸናፊው ተጫራች ጋር ድርድር የተደረገ ከሆነ የግምገማው ሪፖርት የድርድሩን ውጤት እንዲያካትት ድርድሩ ከተካሄደ በኋላ በፕሮጀክት ስምምነቱ የተደረገ ለውጥ ካለ ይህንኑ ሊያመለክት ይገባል።

criterion if applicable and the evaluation process set forth in the request for proposals.

3/ For the purpose of Sub-Article (2) of this Article, the PPP Directorate General may establish thresholds with respect to quality, technical, financial and commercial aspects. Proposals that fail to achieve the thresholds shall be regarded as non-responsive and be rejected from the selection procedure.

4/ The PPP Directorate General may provide a preference margin to be granted to proposals reflecting local participation in the manner as described by directive.

29. Evaluation report

1/ The PPP Directorate General shall, upon evaluating the proposals and before declaring the first ranked bidder, prepare an evaluation report specifying the evaluation criteria, the manner in which the first ranked bidder satisfied the requirements specified in the request for proposals in comparison with the other bidders, and such other information as the PPP Directorate General shall consider necessary.

2/ If negotiations take place with the successful bidder in accordance with Article (31) the evaluation report shall be updated following negotiations and indicate any changes to the Project Agreements as a result of the negotiations.

፴. <u>ለቅድመ-ብቃት ማረጋገጫ ያለፉ ተጫራች በድጋሚ ብቁ መሆኑን የሚያሳይበት ሁኔታ</u>

፩/ የመግአ ዳይሬክቶሬት ጄኔራል በቅድመ-ብቃት ማረጋገጫ ሂደት ያሸነፈውን ተጫራች የቅድመ-ብቃት ማረጋገጫውን ለማካሄድ በዋለው ተመሳሳይ መስፈርት በድጋሚ ለጨረታው ብቃት ያለው መሆኑን እንዲያሳይ ሊጠይቅ ይችላል።

፪/ በዚህ አንቀጽ ንዑስ አንቀጽ (፩) መሰረት በድጋሚ እንዲያሳይ የተጠየቀውን ብቃት ለማሳየት ያልቻለውን ተጫራች የመግአ ዳይሬክቶሬት ጄኔራል ከጨረታው ውጭ እንዲሆን ማድረግ አለበት።

፴፩. <u>ድርድር</u>

፩/ በዚህ አዋጅ አንቀጽ ፳፮ የተመለከተው ስብሰባና ከተወዳዳሪዎች አስተያየት የማሰባሰብ ሂደት የመግአ ዳይሬክቶሬት ጄኔራሉ የፕሮጀክቱን ስምምነቶች አስመልክቶ የሚደረጉትን ድርድሮች ለመቀነስ ወይንም ከተቻለ ለማስቀረት የሚረዱት ሊሆኑ ይገባል።

፪/ ልዩ በሆነ ሁኔታና የመግአ ዳይሬክቶሬት ጄኔራል አስፈላጊ ነው ብሎ ባመነ ጊዜ ብቻ፦

ሀ) ከአሸናፊው ተጫራች ጋር ድርድር ሊያካሂድ፤ እና

ለ) የተቀመጠውን የሚያሟላ ጨረታ ያቀረበው ተጫራች የመጫረቻ ሰነዱን የማቆያ ጊዜ ከጨረታው አሸናፊ ጋር የሚደረገው ድርድር እስኪጠናቀቅ ድረስ እንዲያራዝም መጠየቅ ይችላል።

፫/ የመግአ ዳይሬክቶሬት ጄኔራል ከጨረታው አሸናፊ ጋር የሚያካሂደው ድርድር ያልተሳካ እንደሆነ የተጠየቀውን ጨረታ ካቀረቡ ተጫራቾች ጋር በግምገማው ባገኙት ውጤት ቅደም ተከተል መሠረት ድርድር ያደርጋል።

30. <u>Further demonstration of fulfillment of qualification criteria</u>

1/ The PPP Directorate General may require any bidder that has been prequalified to demonstrate again its qualifications in accordance with the same criteria used for prequalification.

2/ The PPP Directorate General shall disqualify any bidder that fails to demonstrate again its qualifications in accordance with sub article 1 of this Article if requested to do so.

31. <u>Negotiations</u>

1/ The process provided in Article 26 of meetings and receiving comments from the bidders on the request for proposals shall enable the PPP Directorate General to reduce and if possible avoid the need for negotiations of the Project Agrèements.

2/ However, in exceptional circumstances and only where deemed appropriate by the Directorate General, the Directorate General may:

a) enter into negotiations with the successful bidder; and

b) request the second ranked bidder to extend the validity of its bid pending the completion of negotiations with the successful bidder.

3/ Where the negotiations carried out with the successful bidder are unsuccessful, the PPP Directorate General shall enter into negotiations with subsequently ranked bidders in order of their ranking, provided that such bidder submitted a bid that was deemed responsive during the evaluation process

፴፪. የቦርዱ ማፅደቅ

፩/ ቦርዱ ጨረታው ከመካሄዱ በፊት ባፀደቀው የፕሮጀክት ቅርፅ ላይ ጨረታው እየተካሄደ ሳለ መሠረታዊ ለውጥ የተደረገ እንደሆነ የመግአ ዳይሬክቶሬት ጄኔራል ለዚህ ለውጥ የቦርዱን ፈቃድ ማግኘት አለበት። በፕሮጀክቱ ቅርፅ ላይ የተደረገውን ለውጥ በቦርዱ ለማፀደቅ የሚቀርብ ጥያቄ ጨረታው በሚካሄድበት ወቅት ወይም እና ጨረታው አልቆ የፕሮጀክት ስምምነቱ ከመፈፀሙ በፊት በዚህ አንቀጽ ንዑስ አንቀጽ (፪) መሰረት ሊደረግ ይችላል።

፪/ የመግአ ዳይሬክቶሬት ጄኔራል የጨረታውን አሸናፊ ከመግለጹ በፊት የጨረታውን አሸናፊ እንዲያጸድቀው ለቦርዱ ጥያቄ ማቅረብ አለበት። ለዚህ ዓላማ የመግአ ዳይሬክቶሬት ጄኔራል የመወዳደሪያ ሀሳቦችን የግምገማውን ሪፖርት፣ የፕሮጀክቱን ስምምነቶች እና ሌሎች አስፈላጊ ናቸው ተብለው የሚታሰቡ ሰነዶችን ለቦርዱ ማቅረብ አለበት።

፴፫. በሚስጥር ስለሚያዙ አሰራሮች

፩/ የመወዳደሪያ ሀሳቦቹ ከተከፈቱበት ጊዜ አንስቶ ከመወዳደሪያ ሀሳብ ምርመራ፣ ከማብራሪያ እና ከግምገማ እንዲሁም አሸናፊውን ተጫራች በሚመለከት ከሚቀርበው የውሳኔ ሀሳብ ጋር የተያያዙ መረጃዎች በሚስጥርነት መጠበቅ ያለባቸው ሲሆን የመንግሥት እና የግል አጋርነት አሸናፊው ተጫራች እስከሚገለፅ ድረስ ለተጫራቾች ወይም ከሥራው ሂደት ጋር ግንኙነት ለሌላቸው ሰዎች መገለፅ የለበትም።

፪/ የመግአ ዳይሬክቶሬት ጄኔራል የመወዳደሪያ ሀሳቦችን በውስጣቸው የያዙት ሀሳብ ሚስጥራዊነት እንደተጠበቀ እንዲቆይ ለማድረግ በሚያስችል እና በሌሎች ተጫራቾች ሊታወቁ በማይችሉበት ሁኔታ ሊያዝ ይገባል።

32. Approval of the Board

1/The PPP Directorate General shall require the approval of the Board if there is any material change to the project structure previously approved by the Board prior to the tender and which has changed during the tender process .This approval of the Board on changes to the project structure may be sought during the tender process and/or at the end prior to the award of the project in accordance with Sub-Article (2) of this Article.

2/ Prior to the award of the project in accordance the PPP Directorate General shall apply to the Board for approval. For this purpose, the PPP Directorate General shall submit to the Board the evaluation report of the proposals, the Project Agreements and other documents deemed relevant.

33. Process to be Confidential

1/ After opening of the proposals, information relating to examination, clarification, and evaluation of the proposals and recommendations for award must not be disclosed to bidders or other Persons not officially concerned with this process until the award of the Public Private Partnership is announced.

2/ The PPP Directorate General shall treat proposals in such a manner as to avoid the disclosure of their content to competing bidders.

፴፬. የተመረጠውን ተጫራች ስለመግለፅ እና ውል ስለመፈረም

፩/ የመግአ ዳይሬክቶሬት ጄኔራል የመወዳደሪያው ሀሳብ የሚጸናበት ጊዜ ከማለፉ በፊት እና በአንቀጽ (፴፪) መሠረት የቦርዱን ፈቃድ ካገኘ በኋላ በጨረታው የተመረጠውን ተጫራች መግለፅ አለበት። አሸናፊው ተጫራች የሚገለጽበት ማስታወቂያ ውሉ የሚፈረምበትን ቀን መግለጽ አለበት። በጨረታው ተሸናፊ የሆኑ ተጫራቾችም የተመረጠውን ተጫራች ማንነት እና ያልተመረጡበትን ምክንያት የሚገልፅ ማስታወቂያ በደብዳቤ ሊደርሳቸው ይገባል።

፪/ የመግአ ዳይሬክቶሬት ጄኔራል ተዋዋዩ ባለሥልጣን ውሉን እንዲፈርም በጨረታው የተመረጠው ተጫራች ማንነት ከተገለፀበት ቀን ጀምሮ በሁለት ቀናት ጊዜ ውስጥ የፕሮጀክቱን ውል ካስፈላጊ ሰነዶች ጋር ለተዋዋዩ ባለስልጣን መላክ አለበት።

፫/ ተዋዋዩ ባለስልጣን ላልተመረጡት ተጫራቾች አለመመረጣቸውን የሚገልፀው ማስታወቂያ ከመድረሱ ወይም አግባብ ባለው መመሪያ የሚመለከተው የጊዜ ገደብ ከማለፉ በፊት የፕሮጀክቶቹን ስምምነት መፈረም የለበትም።

፬/ ተዋዋይ ወገኖች ስምምነቱን ሲፈርሙ የመግአ ዳይሬክቶሬት ጄኔራል የጨረታውን ውጤት አግባብ ባለው መመሪያ በሚገለፀው መልኩ ይፋ ማድረግ አለበት።

፭/ አሸናፊው ተጫራች ከመገለፁ በፊት ተጫራቹ በቅድመ-ብቃት ማረጋገጫ የተመለከተውን ተፈላጊውን የፋይናንስ እና የቴክኒክ ብቃት የሚያሟላ መሆኑን ለማረጋገጥ የብቃት ማረጋገጫ ማጣሪያ ሊደረግ ይችላል።

34. Notification of Award and Signing of Contract

1/ Prior to the expiry of the period of the bid validity and upon receipt of the Board approval in accordance with Article (32) the PPP Directorate General shall notify the successful bidder that its proposal has been accepted. The notification of the award shall specify the time within which the Project Agreements must be signed. The unsuccessful bidders shall also be informed as to who the successful bidder is and why they have lost the bid.

2/ The PPP Directorate General shall within two days from the date of the award send the Project Agreement to the Contracting Authority together with the necessary documents for it to be signed by the Contracting Authority.

3/ The Projects Agreement shall not be signed by the Contracting Authority prior to the receipt of the notice by all unsuccessful bidders or before the period specified in the relevant directive has lapsed.

4/ Upon execution of a Project Agreement by the parties, the PPP Directorate General shall publish the results of the tender in the manner to be prescribed by directive.

5/ Re-verification of qualifications may be conducted prior to award to confirm that a bidder continues to possess the necessary financial and technical qualification stated in the request for qualifications and/or the request for proposals.

ምዕራፍ ስድስት

በሁለት ደረጃ ጨረታ የግል ባለሀብቱን ስለመምረጥ

፴፭. በሁለት ደረጃ ጨረታ የግል ባለሀብቱን ለመምረጥ መሟላት ያለባቸው ሁኔታዎች

፩/ በዚህ አዋጅ ምዕራፍ አምስት የተደነገጉት ቢኖሩም የመወዳደሪያ ሀሳብ ለማቅረብ በሚያስችል አኳኋን የፕሮጀክቱን ዝርዝር፣ የአፈፃፀም መመዘኛዎችን ወይም የውል ሁኔታዎችን በቂ በሆነ ሁኔታ በዝርዝር መስጠት የማይቻል ከሆነ የመንግሥ ዳይሬክቶሬት ጀኔራል ግዢውን በሁለት ደረጃ ጨረታ መፈፀም ይችላል።

፪/ የመንግሥ ዳይሬክቶሬት ጀኔራል በአንቀጽ (፳) እና (፳፫) መሰረት የቅድመ ማጣራት ሂደት በማድረግ በቅድመ ማጣራቱ ያለፉ ተጫራቾች ብቻ በሁለት ደረጃ ጨረታው ላይ እንዲሳተፉ ያደርጋል።

፴፮. በሁለት ደረጃ ጨረታ የግል ባለሀብት የሚመረጥበት ሥነ ሥርዓት

፩/ ጨረታው የሚከናወነው በሁለት ደረጃ የጨረታ ዘዴ ሲሆን በመጀመሪያው ደረጃ የመወዳደሪያ ሀሳብ መጠየቂያ ተጫራቾች የፕሮጀክት ዝርዝር፣ የአፈፃፀም መመዘኛ፣ የፋይናንስ ግዴታዎችን ወይም የፕሮጀክቱን ሌሎች ባህሪያት እንዲሁም የፕሮጀክት ስምምነቱን የውል ቃሎች የሚገልፅ የመነሻ የመወዳደሪያ ሀሳብ እንዲያቀርቡ መደረግ አለበት።

፪/ የመንግሥ ዳይሬክቶሬት ጀኔራል የመጀመሪያ የጨረታ ማስታወቂያውን ወይም የመጀመሪያ የመወዳደሪያ ሀሳብ መጠየቂያውን እና ተያያዥነት ያላቸውን ሰነዶች በተመለከተ የሚነሱ ጥያቄዎች ላይ ማብራሪያ ለመስጠት ተጫራቾችን ስብሰባ ሊጠራና ውይይት ሊያካሂድ ይችላል።

፫/ የመንግሥ ዳይሬክቶሬት ጀኔራል የቀረቡለትን የመነሻ የመወዳደሪያ ሀሳቦች ከመረመረ በኋላ ፍላጎቱን በተሻለ ሁኔታ የሚገልጽ ዝርዝር የመወዳደሪያ ሀሳብ መጠየቂያ ሰነድ ያዘጋጃል። ይህንንም ለማድረግ በመጀመሪያው

CHAPTER SIX

TWO-STAGE BIDDING

35. Conditions of use of Two-Stage Bidding

1/ Notwithstanding the provisions contained in Chapter Five of this Proclamation, if it is not feasible to formulate a request for proposals with adequate project specifications, performance indicators or contractual terms in a manner sufficiently detailed to enable the submission of final proposals, the PPP Directorate General may engage in a two-stage bidding.

2/ The PPP Directorate General shall run a prequalification procedure in accordance with Articles (20) to (23) to invite only prequalified bidders to participate in the two-stage bidding procedure.

36. Procedure for Two-Stage Bidding

1/ The initial request for proposals shall call upon the bidders to submit, in the first stage of the procedure, initial proposals relating to project specifications, performance indicators, financing requirements or other characteristics of the project as well as to the main contractual terms of the Project Agreement.

2/ The PPP Directorate General may convene meeting and hold discussion with any of the bidders to clarify questions concerning the initial request for proposals or the initial proposals and accompanying documents of the bidders.

3/ Following examination of the proposals received, the PPP Directorate General shall draw up a request for proposal which is more appropriate to its requirements. For

የመወዳደሪያ ሀሳብ ላይ የተመለከተውን የፕሮጀክት ዝርዝር የአፈጻፀም መለኪያ፣ የፋይናንስ ግዴታዎችን ወይም የፕሮጀክቱን ሌሎች ባህሪያት እንዲሁም የፕሮጀክት ስምምነቱን የውል ቃሎች ጨምሮ ማንኛውንም የመወዳደሪያ ሀሳቦችን እና አሸናፊውን ተጫራች ለመምረጥ የሚያገለግሉ መስፈርቶችን መሰረዝ ወይም ማሻሻል እንዲሁም ተጨማሪ ባህሪያትንና መስፈርቶችን ማከል ይችላል።

፬/ በጨረታው ሁለተኛ ደረጃ ላይ ባለሥልጣኑ ተጫራቾች በአንድ የፕሮጀክት ዝርዝር፣ የአፈጻፀም መመዘኛ ወይም የውል ቃሎች ከአንቀጽ (፳፬) እስከ (፴፬) በተደነገገው መሰረት የመጨረሻ ሰነድ እንዲያቀርቡ መጋበዝ አለበት።

፭/ የመግአ ዳይሬክቶሬት ጄኔራል የተመረጠውን ተጫራች ከማሳወቁ እና በአንቀፅ (፳፱)፣ (፴፪) እና (፴፬) መሰረት የፕሮጀክቱን ውል ከመፈጸሙ በፊት የግምገማ ሪፖርት በማዘጋጀት ከቦርዱ ፈቃድ ማግኘት አለበት።

ምዕራፍ ሰባት

በውድድር ላይ የተመሰረተ ውይይት

፴፯. በውድድር ላይ የተመሰረተ ውይይት የሚካሄድበት ሁኔታ

፩/ የመግአ ዳይሬክቶሬት ጄኔራል ልዩ እና ውስብስብነት ላላቸው ፕሮጀክቶች በአንድ ደረጃ ወይም በሁለት ደረጃ ጨረታ ግዢ ማካሄድ ለመንግሥትና ለግል አጋርነት ስምምነት አግባብነት የለውም ብሎ ሲያምን እና በቦርዱ ሲፈቀድ በውድድር ላይ የተመሰረተ ውይይት ማካሄድ ይችላል።

፪/ የመግአ ዳይሬክቶሬት ጄኔራል በውድድር ላይ የተመሰረተ ውይይት ለማድረግ የሚችሉ በአንቀፅ (፳) እና (፳፫) መሰረት የቅድመ-

this purpose it may revise the initial request for proposals by deleting, adding or modifying any aspect of the initial project specifications, performance indicators, financing requirements or other characteristics of the project, including the main contractual terms of the Project Agreements, and any criterion for evaluating and comparing proposals and for ascertaining the successful bidder, as well as by adding characteristics or criteria.

4/ In the second stage of the proceedings, the Directorate General shall invite bidders to submit final proposals with respect to a single set of project specifications, performance indicators or contractual terms in accordance with Articles (24) to (34).

5/ The Directorate General shall prepare an evaluation report and obtain the approval of the Board prior to award and execution of the Project Agreements in accordance with Articles (29), (32) and (34).

CHAPTER SEVEN

COMPETITIVE DIALOGUE

37. Conditions of use of Competitive Dialogue

1/ The PPP Directorate General may engage in competitive dialogue, upon approval by the Board, for particularly complex projects where the PPP Directorate General considers that the use of single stage or two-stage bidding procedure is not appropriate for the award of the Public Private Partnership Agreement.

2/ The PPP Directorate General shall run a prequalification procedure in accordance with Articles (20) to (23) to invite only

ብቃት ማረጋገጫ ውድድር አድርጎ ከመረጣቸው ተጫራቾች ጋር ብቻ ነው።

፴፰. <u>በውድድር ላይ የተመሠረተ ውይይት የሚመራበት ሥነ - ሥርዓት</u>

፩/ የመግአ ዳይሬክቶሬት ጄኔራል ፍላጎቱን እና መስፈርቶችን የሚይዝ ውል መፈጸም የሚፈልግ መሆኑን የሚገልጽ ሰነድ ማዘጋጀት አለበት።

፪/ የመግአ ዳይሬክቶሬት ጄኔራል የቅድመ-ብቃት ማረጋገጫውን ያለፉ ተጫራቾች ፍላጎቱን ለመለየት እና ለመወሰን እንዲቻል በሚካሄደው በውድድር ላይ የተመሰረተ ውይይት ተሳታፊ እንዲሆኑ ይጋብዛል። የመግአ ዳይሬክቶሬት ጄኔራል በውይይቱ ወቅት በፕሮጀክት ስምምነቱ ማናቸውም ባህርያት ላይ ከተጫራቾቹ ጋር ሊወያይ ይችላል።

፫/ የመግአ ዳይሬክቶሬት ጄኔራል በውይይቱ ወቅት ሁሉም ተጫራቾች እኩል ዕድል ያገኙ መሆኑን ሊያረጋግጥ ይገባል። በተለይም ለአንዱ ተጫራች ከሌላው ተጫራች የተሻለ ጥቅም ሊሰጥ በሚችል እና አድሎአዊ በሆነ ሁኔታ መረጃ ሊሰጥ አይገባም።

፬/ የመግአ ዳይሬክቶሬት ጄኔራል አንድ ተጫራች በውይይቱ ሂደት ያቀረበውን የመፍትሔ ሀሳብ ወይም ሌሎች ምስጥራዊነት ያላቸውን መረጃዎች ከተጫራቹ ስምምነት ውጪ ለሌሎች ተጫራቾች ማሳወቅ አይችልም።

፭/ በውይይቱ ወቅት የመግአ ዳይሬክቶሬት ጄኔራል አሸናፊ ተጫራቹን የመምረጫ መስፈርቶችን በማስታወቂያ በመግለጽ ወይም በመግለጫ ሰነዶች ላይ በማካተት በመፍትሄነት የሚቀርቡ ሀሳቦችን ቁጥር ለመቀነስ በተለያዩ ደረጃዎች በተከታታይ ስራ ላይ የሚውሉ ሥርዓቶችን ሊዘረጋ ይችላል። የማስታወቂያው ጥሪ ወይም የመግለጫው ሰነድ እንዲህ ዓይነቱ ስርአት ተፈጻሚ እንደሚሆን በግልጽ ማመልከት አለበት።

prequalified bidders to participate in the competitive dialogue procedure.

38. <u>Procedure for Competitive Dialogue</u>

1/ The PPP Directorate General shall establish a contract notice setting out its needs and requirements, which it shall define in that notice and/or in a descriptive document.

2/ The PPP Directorate General shall invite prequalified bidders to participate in a dialogue the aim of which shall be to identify and define the means best suited to satisfying its needs. The Directorate General may discuss all aspects of the Project Agreements with the prequalified bidders during this dialogue.

3/ During the dialogue, the PPP Directorate General shall ensure equality of treatment among all bidders. In particular, it shall not provide information in a discriminatory manner which may give some bidders an advantage over others.

4/ The PPP Directorate General shall not reveal to other bidders solutions proposed or other confidential information communicated by a bidder participating in the dialogue without his agreement.

5/ The PPP Directorate General may provide for the procedure to take place in successive stages in order to reduce the number of solutions to be discussed during the dialogue stage by applying the award criteria in the contract notice or descriptive document. The contract notice or descriptive document shall indicate that recourse may be had to this option.

፮/ የመንግሥት የግል አጋርነት ዳይሬክቶሬት ጄኔራል ፍላጎቱን የሚያሟላውን የመፍትሄ ሀሳብ እስኪያገኝ ድረስ ካስፈለገም የቀረቡትን የመወዳደሪያ ሀሳቦች ገምግሞ ካጠናቀቀ በኋላም በድርድር ላይ የተመሠረተውን ውይይት መቀጠል አለበት፡፡

፯/ በድርድር ላይ የተመሰረተው ውይይት ከተጠናቀቀ እና የመንግሥት የግል አጋርነት ዳይሬክቶሬት ጄኔራል ውይይቱ መጠናቀቁን ለተጫራቾች ከገለፀ በኋላ በውይይቱ የቀረበውን እና በግልፅ የተመለከተውን የመፍትሔ ሀሳብ መነሻ ያደረገ የመጨረሻ የመጫረቻ ሰነድ እንዲያቀርቡ ተጫራቾችን ይጋብዛል፡፡ እነዚህ የመወዳደሪያ ሀሳቦች የሚያስፈልገውን ዝርዝር ሁሉ የያዙ እንዲሁም ለፕሮጀክቱ አፈፃፀም አስፈላጊ የሆኑ ነገሮችን ሁሉ ያካተቱ ሊሆኑ ይገባል፡፡

፰/ በመንግሥት የግል አጋርነት ዳይሬክቶሬት ጄኔራል ጥያቄ አቅራቢነት የመወዳደሪያ ሀሳብ መጠየቂያዎች ሊብራሩ፣ ሊዘረዘሩ እና በተጣራ መልክ እንዲቀርቡ ሊደረግ ይችላል፡፡ ሆኖም እነዚህ ማብራሪያዎች፣ ዝርዝሮች ወይም ማጣሪያዎች ወይም ተጨማሪ መረጃዎች በመወዳደሪያ ሀሳብ መጠየቂያው ላይ መሰረታዊ የይዘት ለውጥ የሚያስከትሉ፣ ተወዳዳሪነትን የሚያዛቡ እና አድሎአዊ ውጤት ያላቸው ሊሆኑ አይችሉም፡፡

፱/ የመንግሥት የግል አጋርነት ዳይሬክቶሬት ጄኔራል የቀረቡለትን የመወዳደሪያ ሀሳቦች በማስታወቂያ ጥሪ ወይም ገላጭ ሰነድ ላይ በሠፈረው የመወዳደሪያ መስፈርት መሰረት ሊመረምር እና ከፍተኛ ኢኮኖሚያዊ ጠቀሜታ ያለውን የጨረታ ሰነድ ሊመርጥ ይገባል፡፡

፲/ የመወዳደሪያ ሀሳቡን ወይም የመወዳደሪያ ሀሳብ መጠየቂያውን መሠረታዊ ይዘት የማይለውጥ እና ተወዳዳሪነትን የማያዛንፍ ወይም አድልኦ የማይፈጥር እስከሆነ ድረስ የመንግሥት የግል አጋርነት ዳይሬክቶሬት ጄኔራል የላቀ ኢኮኖሚያዊ ጠቀሜታ ያለውን የመወዳደሪያ ሀሳብ ያቀረበው ተጫራች

6/ The PPP Directorate General shall continue such dialogue until it can identify the solution or solutions, if necessary after comparing them, which are capable of meeting its needs.

7/ Having declared that the dialogue is concluded and having so informed the bidders, the PPPDirectorate General shall ask them to submit their final proposals on the basis of the solution or solutions presented and specified during the dialogue. These proposals shall contain all the elements required and necessary for the performance of the project.

8/ The proposals may be clarified, specified and fine-tuned at the request of the PPP Directorate General. However, such clarification, specification, fine-tuning or additional information may not involve changes to the basic features of the proposal or the call for proposals, variations in which are likely to distort competition or have a discriminatory effect.

9/ The PPP Directorate General shall assess the proposals received on the basis of the award criteria laid down in the contract notice or descriptive document and shall choose the most economically advantageous proposal.

10/ At the request of the PPP Directorate General, the bidder identified as having submitted the most economically advantageous proposal may be asked to clarify aspects of the proposal or confirm commitments contained in the

በመወዳደሪያ ሀሳቡ ላይ የተገለፁ አንዳንድ ነጥቦችን እንዲያብራራ ወይም በሰነዱ ላይ የተመለከቱ ግዴታዎችን እንዲያረጋግጥ ሊጠይቅ ይችላል።

፲፩/ የመግአ ዳይሬክቶሬት ጄኔራል በውድድር ላይ በተመሰረተው ውይይት ለሚሳተፉ ተጫራቾች የሚከፈለውን ዋጋ ወይም የክፍያ ተመን ሊወስን ይችላል።

፲፪/ የመግአ ዳይሬክቶሬት ጄኔራል የዉሉ ረታውን አሸናፊ ከማሳወቁ እና የፕሮጀክት ስምምነቱን በአንቀፅ (፳፱)፣(፴፪) እና (፴፬) መሰረት ከመፈረሙ በፊት የግምገማ ሪፖርት በማዘጋጀት ለቦርዱ አቅርቦ ማስፀደቅ አለበት።

ምዕራፍ ስምንት

ቀጥተኛ ድርድር

፴፱ ቀጥተኛ ድርድር የሚካሄድባቸው ሁኔታዎች

፩/ በቦርዱ ሲፀድቅ የመግአ ዳይሬክቶሬት ጄኔራል በሚከተሉት ሁኔታዎች የመንግሥት እና የግል አጋርነት ቀጥተኛ ድርድር ማድረግ ይችላል፦

ሀ) የተፈጠረው አስቸኳይ ሁኔታ አስቀድሞ በተዋዋዩ ባለስልጣን ሊታወቅ የሚችል ወይም በቸልተኝነት ምክንያት የተፈጠረ ካልሆነ በስተቀር አገልግሎቱን ማቅረብ በአስቸኳይ የሚያስፈልግ እና ከአንቀጽ (፳) እስከ (፴፯) ድረስ የተመለከቱትን ለመተግበር ጊዜ የማይሰጥ ሲሆን፤

ለ) ፕሮጀክቱ ለአጭር ጊዜ የሚቆይ እና የተገመተው የኢንቨስትመንት መጠን አግባብ ባለው መመሪያ ከተመለከተው መጠን የማይበልጥ ከሆነ፤

proposal provided this does not have the effect of modifying substantial aspects of the proposal or the call for proposals and does not risk distorting competition or cause discrimination.

11/ The PPP Directorate General may specify prices or payments to bidders in the dialogue.

12/ The PPP Directorate General shall prepare an evaluation report and obtain the approval of the Board prior to award and execution of the Project Agreements in accordance with Articles (29), (32) and (34).

CHAPTER EIGHT

DIRECT NEGOTIATION

39. Conditions for use of Direct Negotiations

2/ The PPP Directorate General may engage in direct negotiation in respect of a Public Private Partnership, upon approval by the Board, only where the following conditions are satisfied:

a) Where there is an urgent need in the provision of the service and engaging in the procedures set forth in Articles (20) to (37) would be impractical, provided that the circumstances giving rise to the urgency where neither foreseeable by the Contracting Auathority nor the result of dilatory conduct on its part;

b) Where the project is of short duration and the anticipated initial investment value does not exceed the amount to be specified by directive.

ሐ)ፕሮጀክቱ ከአገር መከላከል ወይም ደህንነት ጋር ተያያዥ የመሠረተ-ልማት አቅርቦት ሲሆን፤

መ) አገልግሎቱ የአዕምሮአዊ ንብረትን፣ የንግድ ሚስጥሮችን ወይም ሌሎች በብቸኝነት የተያዙ መብቶችን ወይም በተወሰኑ ሰዎች ባለቤትነት ወይም ይዞታ ስር ያሉ ንብረቶችን የሚመለከት ሲሆን እና የተፈለገውን አገልግሎት ማግኘት የሚቻለው ከአንድ ምንጭ ብቻ ሲሆን፤

ሠ) የቅድመ ብቃት ማረጋገጫ ወይም የመወዳደሪያ ሀሳብ መጠየቂያ ጥሪ ቀርቦ ምንም አይነት የቅድመ-ብቃት ማረጋገጫ ማመልከቻ ወይም የመወዳደሪያ ሀሳብ ያልቀረበ እንደሆነ ወይም ሁሉም የመወዳደሪያ ሀሳቦች የመወዳደሪያ ሀሳብ በመጠየቂያው ማስታወቂያ የተመለከቱ መመዘኛዎችን የማያሟሉ ሲሆኑ እና የመግእ ዳይሬክቶሬት ጄኔራል አዲስ የቅድመ ብቃት ማረጋገጫ ወይም የመወዳደሪያ ሀሳብ መጠየቂያ ማስታወቂያ ቢወጣም ለፕሮጀክቱ ተስማሚ የሚሆነውን ተጫራች በሚፈለገው ጊዜ ውስጥ ማግኘት የማይቻል መሆኑን ሲያምን፤

ረ) የመንግሥት እና የግል አጋርነት ፕሮጀክቱ የተወሰነ ዋጋ፣ የጊዜ ቆይታው፣ ወይም አግባብ ባለው መመሪያ የሚወሰኑ ሌሎች ሁኔታዎች ከኢንዱስትሪያል እና ከሌላ የኢንቨስትመንት ስራ ጋር ተያያዥ ከሆኑ እና አገልግሎቱ በኢንዱስትሪ ወይም በኢንቨስትመንት ስራ በተሰማራው ባለሀብት መከናወን ያለበት ሲሆን።

፪/ በዚህ አንቀጽ ንዑስ አንቀጽ (1)(ሐ) ከተመለከተው የፕሮጀክት ስምምነት ውጪ የሆኑ የፕሮጀክት ስምምነቶችን በተመለከተ የመግእ ዳይሬክቶሬት ጄኔራል ድርድር ለመጀመር መዘጋጀቱን በማስታወቂያ ሊገልጽ ይገባል።

c) Where the project involves infrastructure projects related to national defense or national security;

d) Where there is only one source capable of providing the required service, such as when the provision of the service requires the use of intellectual property, trade secrets or other exclusive rights or assets owned or possessed by a certain Person or Persons;

e) When an invitation to the prequalification proceeding or a request for proposals has been issued but no applications for qualification or proposals were submitted or all proposals failed to meet the evaluation criteria set forth in the request for proposals and if, in the judgment of the PPP Directorate General, issuing a new invitation to the prequalification proceedings and a new request for proposals would be unlikely to result in a project award within the required time frame;

f) Where a Public Private Partnership project, of limited value, length or otherwise as specified by directive, is ancillary andthe necessary complement to a mining, industrial or other type of investment and must be carried out by the private sponsor of such mining, industrial or other type of investment.

2/ Except for Project Agreements negotiated pursuant to sub article (1) of this Article, the PPP Directorate General must publish a notice of its intention to commence negotiations in respect of Project Agreements.

፵. ቀጥተኛ የሆነ ድርድር የሚካሄድበት ሥነ- ሥርዓት

፩/ የመንግሥት አጋርነት ዳይሬክቶሬት ጄኔራል ፕሮጀክቱን ለመፈፀም ብቃት አላቸው ብሎ ከሚያምናቸው ቁጥራቸው አስፈላጊ ነው ብሎ ከሚወስነው ተጫራቾች ጋር ሁሉ ሁኔታው በሚፈቅደው መሰረት ድርድሩን ያካሂዳል። የመንግሥት አጋርነት ዳይሬክቶሬት ጄኔራል አስፈላጊ ነው ብሎ ካመነ የቅድመ-ብቃት ማረጋገጫ ሊያካሂድ ይችላል።

፪/ የመንግሥት አጋርነት ዳይሬክቶሬት ጄኔራል ፍላጎቱንና መስፈርቱን የሚገልፅ ሰነድ እንዲሁም የፕሮጀክት ስምምነቱን ረቂቅ ማዘጋጀትና የመወዳደሪያ ሀሳቦች የሚገመገሙበትን እና ደረጃ የሚወጣበትን የግምገማ መስፈርት ማዘጋጀት አለበት። ይህ ሰነድ ለተጫራቾች መገለፅ አለበት።

፫/ የመንግሥት አጋርነት ዳይሬክቶሬት ጄኔራል ዋጋን እና የክፍያ ዘዴን ጨምሮ በፕሮጀክቱ ሙሉ ይዘት ላይ በነፃነት ድርድር ሊያደርግ ይገባል።

፬/ በድርድሩ ወቅት የመንግሥት አጋርነት ዳይሬክቶሬት ጄኔራል ሁሉም ተጫራቾች በእኩልነት የሚስተናገዱበት ሁኔታ መኖሩን ማረጋገጥ አለበት። በተለይም የመንግሥት አጋርነት ዳይሬክቶሬት ጄኔራል ለተወሰኑ ተጫራቾች ከሌሎቹ በተሻለ ሁኔታ ጥቅም ሊያስገኝ በሚችል መልኩ አድልኦ ባለበት ሁኔታ መረጃ መስጠት የለበትም።

፭/ የመንግሥት አጋርነት ዳይሬክቶሬት ጄኔራል የግምገማ መስፈርቱን በመጠቀም በድርድሩ ተሳታፊ የሆኑ ተጫራቾችን ቁጥር ለመቀነስ እንዲችል ድርድሩ ደረጃ በደረጃ እንዲካሄድ ሊወስን ይችላል። ይህ አካሄድ የተመረጠ ከሆነ ለተጫራቾች የሚቀርበው የጨረታው ዝርዝር መግለጫ ይህንኑ አማራጭ ማመልከት አለበት።

፮/ የመንግሥት አጋርነት ዳይሬክቶሬት ጄኔራል የጨረታውን አሸናፊ ከማሳወቁ እና የፕሮጀክት ስምምነቱን በአንቀጽ ፳፱፣ ፴፪ እና ፴፬ መሰረት ከመፈጸሙ በፊት የግምገማ ሪፖርት በማዘጋጀት ከቦርዱ ፈቃድ ማግኘት አለበት።

40. Procedures for Direct Negotiation

1/ The PPP Directorate General may engage in negotiations with as many Persons as the PPP Directorate General Judges capable of carrying out the project as circumstances permit. The PPP Directorate General may run a prequalification procedure if it deems it appropriate.

2/The PPP Directorate General shall prepare a description of its needs and requirements, a draft of the Project Agreements and establish evaluation criteria against which proposals shall be evaluated and ranked. This document shall be communicated to the bidder(s).

3/ The Directorate General shall be free to negotiate to all aspects of the project including price and payment terms.

4/ During the negotiations, the PPP Directorate General shall ensure the equal treatment of all bidders. In particular, they shall not provide information in a discriminatory manner which may give some bidders an advantage over others.

5/ The PPP Directorate General may provide for the negotiated procedure to take place in successive stages in order to reduce the number of bidders to be negotiated by applying the evaluation criteria. The specifications communicated to the bidder(s) shall indicate whether recourse has been had to this option.

6/ The PPP Directorate General shall prepare an evaluation report and obtain the approval of the Board prior to award and execution of the Project Agreements in accordance with Articles 29, 32 and 34.

ምዕራፍ ዘጠኝ

በራስ ተነሳሽነት የሚቀርብ የፕሮጀክት ሀሳብ

፵፩. በራስ ተነሳሽነት የሚቀርብ የፕሮጀክት ሀሳብ ተቀባይነት የሚያገኝበት ሁኔታ

የመንግስ ዳይሬክቶሬት ጀኔራል ከተዋዋዩ ባለስልጣን ጋር በመመካከር በራስ ተነሳሽነት የሚቀርብን የተለየ የፕሮጀክት ሀሳብ ለመመርመር እና ለመቀበል ይችላል፤ ሆኖም የፕሮጀክቱ ሀሳብ ለጥናት ወይም ለትግበራ በቦርዱ ተቀባይነት ካገኘ የመንግሥትና የግል አጋርነት ፕሮጀክት ጋር ግንኙነት ያለው መሆን የለበትም።

፵፪. በራስ ተነሳሽነት የቀረበ የፕሮጀክት ሃሳብ

፩/ የመንግስ ዳይሬክቶሬት ጀኔራል በራስ ተነሳሽነት የተዘጋጀ የፕሮጀክት ሰነድ ሲደርሰው የቅድመ-ምርመራ ካካሄደ በኋላ አቅራቢው ያዘጋጀው ፕሮጀክት ለሕዝብ ጥቅም የሚያስገኝ ስለመሆን አለመሆኑ ለአቅራቢው መግለፅ አለበት።

፪/ ፕሮጀክቱ ሕዝባዊ ጠቀሜታ ይኖረዋል ተብሎ ሲታመን የመንግስ ዳይሬክቶሬት ጀኔራል የአቅራቢውን ብቃት፣ ፕሮጀክቱ በቴክኒክ እና በኢኮኖሚ ረገድ አዋጭ መሆኑን እና የመንግስ ዳይሬክቶሬት ጀኔራል ሊቀበለው በሚችለው ሁኔታ ስኬታማ በሆነ ሁኔታ ፕሮጀክቱ ተፈጻሚ መሆን የሚችል መሆን አለመሆኑን በሚገባ መመርመር እንዲችል አቅራቢው በራስ ተነሳሽነት ያቀረበውን የፕሮጀክት ሀሳብ የሚመለከት ተጨማሪ መረጃ እንዲያቀርብ ሊጠይቀው ይችላል።

፫/ የመንግስ አዳይሬክቶሬት ጀኔራል በራስ ተነሳሽነት የቀረበውን የፕሮጀክት ሀሳብ ሲመረምር ከሰነዱ የሚመነጨውን ወይም በሰነዱ የተጠቀሰውን የአዕምሮአዊ ንብረት፣ የንግድ ሚስጥር እና ሌሎች ተያያዥነት ያላቸውን የብቸኝነት መብቶች ማክበር አለበት።

CHAPTER NINE

UNSOLICITED PROPOSALS

41. Admissibility of Unsolicited Proposals

The PPP Directorate General in consultation with the Contracting Authority may review and accept Unsolicited Proposals Provided that such proposals do not related to the project that has already received approval by the Board for study or implementation as a PPP project.

42/ Unsolicited Proposal

1/ Following receipt and preliminary examination of an Unsolicited Proposal, the PPP Directorate General shall make use reasonable efforts to inform the proponent whether or not the project is considered to be potentially in the public interest.

2/ If the project is considered to be potentially in the public interest, the PPP Directorate General shall invite the proponent to submit as much information on the proposed project as is feasible to allow the Directorate General to make a proper evaluation of the proponent's qualifications and the technical and economic feasibility of the project and to determine whether the project is likely to be successfully implemented in the manner proposed in terms acceptable to the Directorate General.

3/ In considering an Unsolicited Proposal, the PPP Directorate General shall respect the intellectual property, trade secrets or other exclusive rights contained in, arising from or referred to in the proposal.

፵፫. በራስ ተነሳሽነት የቀረበ የፕሮጀክት ሀሳብ የሚታይበት ሥርዓት

፩/ የመግአ ዳይሬክቶሬት ጄኔራል በአንቀፅ ፵፪(፪) የተመለከተውን ሰነድ ከተረከበ በኋላ በራስ ተነሳሽነት የቀረበውን ፕሮጀክት የሚቀበል ስለመሆን አለመሆኑ ውሳኔ መስጠት አለበት፡፡

፪/ በራስ ተነሳሽነት የቀረበው የፕሮጀክት ሀሳብ በመግአ ዳይሬክቶሬት ጄኔራል ተቀባይነትን ካገኘ ጨረታ ከመውጣቱ በፊት የአዋጭነት ጥናት በተዋዋዩ ባለስልጣን ተዘጋጅቶ ቦርዱ እንዲያጸድቀው ይቀርባል፡፡

፫/ በራስ ተነሳሽነት የቀረበውን የፕሮጀክት ሀሳብ የሚያስፈጽመው አሸናፊ የሚመረጠው በዚህ አዋጅ ከምዕራፍ ፭ እስከ ፯ ድረስ በተመለከተው አኳኋን ውድድር ከተካሄደ በኋላ በሚወሰን ምርጫ ወይም በአንቀጽ (፴፱) የተመለከተውን መስፈርት የሚያሟላ ከሆነ በቀጥታ በሚደረግ ድርድር ይሆናል፡፡

፬/ በራስ ተነሳሽነት የፕሮጀክት ሃሳብ ያቀረበው ሰው በቀረበው ሀሳብ መነሻ በሚደረግ በማንኛውም የጨረታ ውድድር ላይ እንዲሳተፍ መጋበዝ አለበት፡፡ የመግአ ዳይሬክቶሬት ጄኔራል የጨረታ ግምገማ በሚያካሂድበት ወቅት የፕሮጀክት ሀሳብ አቅራቢው በቴክኒክ ወይም/እና በፋይናንስ ግምገማ ተጨማሪ ነጥብ እንዲሰጠው ወይም በጨረታው አሸናፊ የማይሆን ከሆነ ላቀረበው ጥናት በገንዘብ ካሳ እንዲከፈለው ሊወስን ይችላል፡፡ የሚሰጠው ተጨማሪ ነጥብ ወይም የካሣ ክፍያ መጠን በዚህ አንቀፅ ንዑስ አንቀጽ (፩) በተመለከተው መሰረት የፕሮጀክቱ ሀሳብ ተቀባይነት ሲያገኝ ስምምነት ሊደረግበት ይገባል፡፡ የፕሮጀክት ሀሳብ አቅራቢው ማንነት፣ ተጨማሪ ነጥብ የሚሰጥበት ሁኔታ እና የሚከፈለው የካሳ መጠን በጨረታ ሰነድ ላይ መገለፅ አለበት፡፡

43. Procedure for Unsolicited Proposal

1/ Following receipt of the documents provided in Article 42(2) the PPP Directorate General shall formally decide whether to accept or reject the potential project derived from the Unsolicited Proposal.

2/ If the Unsolicited Proposal is accepted by the PPP Directorate General, the Contracting Authority shall proceed to prepare a feasibility study and submit it to the Board prior to moving to tender.

3/ An Unsolicited Proposal shall be awarded through a competitive selection procedure in accordance with Chapters 5 to 7 and may be awarded through direct negotiations only if it meets the requirements set forth in Article 39.

4/ The proponent shall be invited to participate in any competitive selection procedure initiated in response to its unsolicited proposal. The PPP Directorate General may decide to award the proponent a bonus on its technical and/or financial score during the competitive selection procedure or award the proponent a financial compensation for studies undertaken by the proponent should the proponent not be awarded the project. The bonus point scheme principle or the amount of the financial compensation shall be agreed upon acceptance of the proposal in accordance with Sub-Article (1) of this Article. The identity of the proponent, the detailed allocation of bonus points or the amount of the potential compensation to the proponent shall be indicated in the competitive selection procedure documents.

ምዕራፍ አሥር

የመንግሥትና የግል አጋርነት ስምምነት ይዘትና አተገባበር

፵፬ የፕሮጀክት ኩባንያው አመሠራረት

፩/ የመግአ ዳይሬክቶሬት ጄኔራል የጨረታ አሸናፊው ብቸኛ አላማው የመንግሥትና የግል አጋርነት ስምምነቱንና ሌሎች የፕሮጀክቱን ስምምነቶች መተግበርና ማስፈፀም የሆነ በኢትዮጵያ ፌደራላዊ ዲሞክራሲያዊ ሪፐብሊክ ውስጥ የሚመሰረት ኩባንያ እንዲያቋቁም ማድረግ አለበት፡፡

፪/ የመንግሥትና የግል አጋርነት ስምምነት የሚከተሉትን ሊያካትት ይችላል፦

ሀ) የፕሮጀክት ኩባንያው ሊኖረው የሚገባውን የአክሲዮን ካፒታል መጠን፤

ለ) በፕሮጀክት ኩባንያው የመተዳደሪያ ደንብ፣ የባለአክስዮኖች ስምምነት እና ሌሎች ተመሳሳይ ስምምነቶች ላይ የሚደረግ ማሻሻያ መጽደቅ እንዳለበት፤ እና

ሐ) በዚህ አንቀጽ በንዑስ አንቀጽ (፫) የተደነገገውን ጨምሮ የፕሮጀክት ኩባንያውን አክሲዮን እና ሌላ የባለቤትነት ጥቅም ለሶስተኛ ወገን ማስተላለፍ ላይ ገደብ ማድረግን፡፡

፫/ ተዋዋዩ ባለስልጣን በፕሮጀክት ኩባንያው ወይም የኩባንያውን አክሲዮኖች በባለቤትነት በሚይዝ ኩባንያ ውስጥ የአነስተኛ አክሲዮን ባለቤት ሊሆን ይችላል፡፡

፬/ በመንግሥትና በግል አጋርነት ስምምነት ውስጥ በሌላ ሁኔታ ካልተመለከተ በስተቀር፣ አክሲዮንን ጨምሮ ማንኛውም በፕሮጀክት ኩባንያው ውስጥ በውሳኔ አሰጣጥ ረገድ ባለው ከፍተኛ ድርሻ ላይ ለውጥ የሚያስከትል የባለቤትነትን ጥቅም ማስተላለፍ ተግባር ከመፈጸሙ በፊት ከተዋዋዩ

CHAPTER TEN

CONTENT AND IMPLEMENTATION OF THE PUBLIC PRIVATE PARTNERSHIP AGREEMENT

44. Establishment of a Project Company

1/ The PPP Directorate General shall require that the successful bidder establish a Project Company incorporated under the laws of the Federal Democratic Republic of Ethiopia whose sole purpose shall be to execute and implement the Public Private Partnership Agreement and other Project Agreements, if any.

2/ The Public Private Partnership Agreement may:

a) establish requirements around the share capital of the Project Company;

b) require approval of changes to by-laws and shareholders' agreement and other related agreements of the Project Company; and

c) impose restrictions around the transfer of ownership interests, such as shares, in the Project Company, including in accordance with sub-article (3) of this Article.

3/ A Project Company may include a Public Entity as a minority shareholder in the Project Company or its holding.

4/ Except as otherwise provided in the Project Agreement, any ownership interests in the Project Company, such as shares, the transfer of which would result in a change of control of the Project Company shall not be permitted without

ባለስልጣን ፍቃድ ሊገኝ ይገባል። የመንግስትና የግል አጋርነት ስምምነት የባለስልጣኑ ፈቃድ ሊገኝ የሚችልበትን ሁኔታ ሊወስን ይችላል።

፵፭. የመንግሥትና የግል አጋርነት የሚቆይበት የጊዜ ገደብ

የመንግሥትና የግል አጋርነት የሚቆይበት የጊዜ ገደብ በፕሮጀክቱ ስምምነት ውስጥ ይወሰናል።

፵፮. ዋጋ

፩/ የግል ባለሀብቱ በሚከተለው አኳኋን የገንዘብ ጥቅም ሊያገኝ ይችላል፦

ሀ) የዚህ አንቀጽ ፊደል ተራ (ለ) ድንጋጌ እንደተጠበቀ ሆኖ፣ በተዋዋይ ባለስልጣን ወይም በተዋዋዩ ባለስልጣን ስም የሚከፈል ገንዘብ፤

ለ) ተጠቃሚዎች ወይም ሸማቾች ለሚያገኙት አገልግሎት ከሚከፍሉት ታሪፍና ክፍያ፣ እና/ወይም

ሐ) በዚህ አንቀጽ የተጠቀሰው ክፍያ እና የአገልግሎት ታሪፍ በጣምራ።

፪/ የግል ባለሀብቱ በመገልገያ ስፍራዎች ለመጠቀም እና የሚሰጣቸውን አገልግሎቶች ለማግኘት በፕሮጀክት ስምምነት በተመለከተው መሠረት ታሪፍ ወይም ክፍያን የመወሰን፣ የመቀበል ወይም የመሰብሰብ መብት አለው። የፕሮጀክቱ ስምምነት ከዚህም በተጨማሪ የታሪፍ ወይም የክፍያ አወሳሰንና የማስተካከያ ዘዴዎችን ቀመር ሊይዝ ይችላል።

፫/ በመገልገያ ስፍራዎች ለመጠቀም ወይም አገልግሎት ለማግኘት የሚጠየቀው ክፍያ የግል ባለሀብቱን የፋይናንስ አቋም የሚያስጠብቅ፣ ካፒታልን መሳብ የሚያስችል፣ ስራውን በተቀላጠፈ አኳኋን ማካሄድ የሚያስችል እና የሚቀበለውን ስጋት ማካካስ የሚያስችል መሆኑ እንደተጠበቀ ሆኖ የአገልግሎት ተቀባዩን የመክፈል አቅም ያገናዘበ እና ተዋዋዩ ባለስልጣን እና ተጠቃሚው ለሚከፍሉት ገንዘብ ተመጣጣኙን ዋጋ የሚያስገኝ መሆን አለበት።

the consent of the Contracting Authority. The Project Agreement shall set forth the conditions under which consent of the Contracting Authority shall be given.

45. Duration of Public Private Partnership

The duration of the Public Private Partnership shall be set forth in the Project Agreements.

46. Price

1/The Private Party shall receive financial compensation by way of:

a) compensation by or on behalf of the Cotracting Auathority subject to paragraph (b) of this Article;

b) tariffs or fees collected by the Private Party from users or consumers of a service provided to them; and/or

c) a combination of such compensation and such charges or fees.

2/ The Private Party shall have the right to charge, receive or collect tariffs or fees for the use of the facility or its services in accordance with the terms and conditions set forth in the Project Agreements, which shall additionally provide the methods and formulae for the establishment and adjustment of those tariffs or fees.

3/ The cost of delivering a facility or service shall be affordable and provide Value for Money to the Contracting Authority and end users while enabling the Private Party to maintain its financial integrity, attract capital, operate efficiently and compensate a Private Party for any assumed risk.

፵፯. ከመንግሥት የሚደረግ ድጋፍ

፩/ በበቂ ምክንያት የተደገፈና ለገንዘብ ተመጣጣኙን ዋጋ ለማስገኘት አስፈላጊ በመሆኑ ምክንያት አግባብ ባለው የመንግሥት አካል ሲፈቀድ የመንግሥት ዳይሬክቶሬት ጄኔራል ወይም/እና ሚኒስቴሩ የፕሮጀክቱን ቀጣይነት እና/ወይም የፋይናንስ አዋጭነቱን ለማረጋገጥ ሲባል ለግል ባለሀብቱ የኢኮኖሚ ድጋፍ ሊያደርጉ እና የገቢ ዋስትና ሊሰጡ ይችላሉ።

፪/ በዚህ አንቀጽ መሠረት የሚደረገው ድጋፍ በመወዳደሪያ ሀሳብ መጠየቂያው ሰነድ ውስጥ ሊመለከት እና በአንቀጽ (፱) እና (፲፰) መሠረት በቦርዱ ሊደገፍ ይገባል።

፫/ የኢኮኖሚ ድጋፉ ከዚህ ከታች በተዘረዘሩት ብቻ ሳይወሰን የሚከተሉትን ሊያጠቃልል ይችላል፦

ሀ) በመገልገያ ስፍራዎች ለመጠቀም ወይም አገልግሎት ለማግኘት የግል ባለሀብቱ ከሚቀበለው ታሪፍና ክፍያ በተጨማሪ ወይም በምትክነት የሚደረግ ቀጥተኛ ክፍያ፣ የዚህ ዓይነቱ ድጋፍ የመገልገያ ስፍራዎችን ወይም አገልግሎቱን ለተጠቃሚዎች ዝግጁ በማድረግ ብቻ የሚፈጸም ክፍያ፣ የገንዘብ ድጋፍ፣ የካፒታል ድጋፍ፣ ለዝቅተኛው የተገልጋይ ቁጥር እና ገቢ ዋስትና መስጠት እና በዝቅተኛ ደረጃ ጥቅም ላይ ሊውል ለሚችለው መጠን ወይም በተዘጋጀው አቅም ያህል ክፍያ መፈጸም እና ለግዢው ዋስትና መስጠት፤

ለ) ሀብትን ማስተላለፍ እና በመሬት የመጠቀም መብት መስጠትን ጨምሮ በዓይነት የሚደረግ መዋጮ፤

ሐ) የክፍያ ዋስትና፣ የዋስትና ሠነዶች፣ ግዴታ የመግቢያ ሰነድ ወይም አስገዳጅነት ያለው የመግባቢያ ደብዳቤ፤ እና

መ) ተዋዋይ ባለስልጣን በፕሮጀክት ስምምነቶች የገባውን ግዴታ የሚፈፅም ለመሆኑ ዋስትና መስጠት።

47. Government Support

1/ When duly justified and required on the basis of Value for Money and with the prior approval of the Board, or the appropriate authority if required, the Directorate General and/or the Ministry may provide economic support and guarantees to ensure sustainability, implementation and/or financial viability of the project.

2/ This economic support must be initially identified in the request for proposals documentation and must have been recommended by the Board in accordance with Article (9) and (18) of this Proclamation.

3/ The economic support may take any reasonable form, including (but not limited to):

a) Direct payments to the Private Party as a substitute for, or in addition to, tariffs or fees for the use of the facility or its services. These may include availability payments, cash subsidies, capital grants, minimum trafficking or revenue guarantees and minimum off-take or capacity payments and purchase guarantees;

b) Contributions in-kind, including asset transfers and land usage rights;

c) Payment guarantees, securities, undertaking or binding letters of comfort;

d) Guarantees for the performance of obligations of the Contracting Authority under the Project Agreement.

፬/ በዚህ አንቀጽ ንዑስ አንቀጽ ፫ መሠረት የሚሰጠው ድጋፍ በመግለ ስምምነት ውስጥ መመልከት አለበት።

፵፰. የንብረት ባለቤትነት

አግባብነት ባላቸው ህጎች የተደነገገው እንደተጠበቀ ሆኖ፣ የፕሮጀክቱ ስምምነት የትኞቹ ሀብቶች የመንግሥት ሀብት እንደሆኑ እና እንደማይሆኑ እንዲሁም የትኞቹ ሀብቶች የግል ባለሀብቱ ሀብቶች እንደሆኑ እና እንደሚሆኑ በግልጽ ማሳየት አለበት።

፵፱. ፕሮጀክቱ ከሚገኝበት ቦታ ጋር የተያያዘን መብት ስለማግኘት

፩/ ተዋዋዩ ባለስልጣን ወይም አግባብነት ያለው ሌላ መንግሥታዊ ተቋም በሕግ እና በመንግሥትና በግል አጋርነት ስምምነቱ መሠረት የግል ባለሀብቱ ፕሮጀክቱን ለማስፈፀም የሚያስፈልጉትን የፕሮጀክት ቦታና የይዞታ መብቶችን እንዲያገኝ መርዳት ይኖርባቸዋል።

፪/ ፕሮጀክቱን ለማስፈፀም የሚያስፈልግ መሬት አስገዳጅ በሆነ ሁኔታ የመውሰድ ተግባር መከናወን ያለበት አግባብ ባላቸው የንብረት መውረስ ሕጎች መሠረት ነው።

፫/ የግል ባለሀብቱ የመሬቱ ባለይዞታ በመሆን ፕሮጀክቱን ለማስፈፀም ሊጠቀምበት ይችላል። ከመሬት ሥርዓትና አጠቃቀም ጋር የተያያዙ እንዲሁም የጨረታ ስርዓትን የሚመለከቱ ገደቦች በፕሮጀክቱ ይዞታ ስር ባለ መሬት ላይ ተፈፃሚ አይሆኑም።

፬/ የግል ባለሀብቱ ከገንዘብ ተቋማት ብድር ለማግኘት ይረዳው ዘንድ በፕሮጀክቱ ባለቤትነት ስር ያለውን የማይንቀሳቀስ ንብረት በመያዣነት መስጠት ይችላል።

4/ The economic support to be granted in accordance with sub-article 3 of this Article shall be provided in the Project Agreement.

48. Ownership of Assets

Without prejudice to relevant laws, the Project Agreement shall specify, as appropriate, which assets are or shall be public property and which assets are or shall be the private property of the Private Party.

49. Acquisition of Rights related to the Project Site

1/ The Contracting Authority or other Public Entity under the terms of the law and the Public Private Partnership Agreement shall make available to the Private Party or, as appropriate, shall assist the Private Party in obtaining such rights related to the project site, including title thereto, as may be necessary for the implementation of the project.

2/ Any compulsory acquisition of land that may be required for the implementation of the project shall be carried out in accordance with the relevant law relating to expropriation.

3/ The Private Party may possess land and use it for the purpose of the implementation of the project. The restrictions in terms and tenure of land and bidding system shall not apply on land that is covered under the project.

4/ The Private Party shall have the right to mortgage the immovable property developed under the project in order to obtain loans from financial institutions.

፶. የመሠረተ-ልማት ሥራዎችን ስለማካሄድ

፩/የሚከተሉትን ማረጋገጥ እንዲቻል የፕሮጀክት ስምምነቱ እንደአስፈላጊነቱ የግል ባለሀብቱ የሚኖሩበትን ግዴታዎች መዘርዘር አለበት፦

ሀ) ለአገልግሎቱ ያለውን ፍላጎት ማሟላት በሚያስችል መጠን የአገልግሎት አቅርቦትን ለማሻሻል፤

ለ) የአገልግሎቱን ቀጣይነትለማረጋገጥ፤

ሐ) አገልግሎቱ ለሁሉም ተጠቃሚዎች በተመሳሳይ ሁኔታ የሚቀርብ መሆኑን ማረጋገጥ፤ እና

መ) ሌሎች አገልግሎት አቅራቢዎች ከአድልኦ በነፃ መልኩ በግል ባለሀብቱ የሚተዳደሩ የሕዝብ መሰረተ-ልማቶች የመጠቀም መብታቸውን ለማረጋገጥ፤

፪/ በተዋዋይ ባለስልጣን ወይም በሌላ ስልጣን በተሠጠው መንግሥታዊ ተቋም ፈቃድ የግል ባለሀብቱ የመገልገያ ስፍራዎችን ለማስተዳደር የሚያስችሉ ደንቦችን ለማውጣትና ስራ ላይ ለማዋል ይችላል።

፶፩. የሥራ አፈፃፀም ዋስትና

፩/ የመንግሥትና የግል አጋርነት ስምምነት የግል ባለሀብቱ በስምምነቱ በሚገባው ግዴታ መሠረት የሚፈጽም ስለመሆኑ ማረጋገጫ ለማግኘት ሊያቀርብ የሚገባውን ዋስትና ዓይነትና መጠን ሊወስን ይችላል።

፪/ የዚህ ዓይነቱን የሥራ አፈፃፀም ዋስትና ማቅረብ አስፈላጊ ሆኖ ሲገኝ፣ የመንግሥትና የግል አጋርነት ስምምነት፦

ሀ) ዋስትናውን ሊሰጥ የሚገባውን ተቋም፣ የዋስትናውን ባህርይ፣ ቅርጽ፣ መጠንና ሌሎች የዋስትናውን ዋና ዋና የውል ቃሎችና ሁኔታዎች፣ እና

ለ) የዋስትናውን የገንዘብ መጠን፣ እንደሁኔታው ዋስትናው የሚቀርብበትን ወይም የሚወረስበትን አኳኋን በዝርዝር የሚገልጽ መሆን አለበት።

50. Operation of Infrastructure

1/ The Project Agreements shall set forth, as appropriate, the extent of the Private Party's obligations to ensure:

a) The modification of the service so as to meet the demand for the service;

b) The continuity of the service;

c) The provision of the service under essentially the same conditions for all users; and

d) The non-discriminatory access of other service providers to any public infrastructure network operated by the Private Party.

2/ The Private Party shall have the right to issue and enforce rules governing the use of the facility, subject to the approval of the Contracting Authority or any other authorized Public Entity.

51. Performance Guarantee

1/ The Project Agreements may specify security to be provided by the Private Party to guarantee the performance of any of its obligations under the Project Agreements.

2/ If such security is required, the Project Agreements shall also set forth:

a. the requirements with respect to the issuer and the nature, form, amount and other principal terms and conditions of the required security; and

b. The amount and the details of the ways the security is to be submitted or forfeited as the case may be.

፶፪. በመያዣ ላይ ያለ ጥቅም

፩/ በፕሮጀክት ስምምነቱ የተቀመጡ ገደቦች እንደተጠበቁ ሆነው የግል ባለሀብቱ ከፕሮጀክቱ መሠረተ-ልማት በተጨማሪ የሚከተሉትን ጨምሮ ማንኛውንም ሀብቱን፣ መብቱን ወይም ጥቅሙን የፋይናንስ ፍላጎቱን ለማሟላት በመያዣነት ለመስጠት ይችላል፦

ሀ) የግል ባለሀብቱ የሚንቀሳቀሱ ወይም የማይንቀሳቀሱ ንብረቶችን ወይም በፕሮጀክቱ ሀብቶች ውስጥ ያለውን ጥቅም ማስያዝ፤

ለ) የግል ባለሀብቱ ለሚያቀርባቸው መገልገያዎች ወይም ለሚሰጣቸው አገልግሎት ከተሰበሰበው ወይም ከሚሰበሰበው ክፍያ የሚገኘውን ገቢ ማስያዝ።

፪/ የግል ባለሀብቱ ባለአክሲዮኖች ያላቸውን አክሲዮን በመያዣነት መስጠት ወይም ማናቸውንም ዓይነት የመያዣ መብት ሊፈጥሩበት ይችላሉ።

፫/ በዋስትና ሊሰጥ እንደማይችል በሕግ የተከለከለ የሕዝብ ንብረት ወይም ለሕዝብ አገልግሎት ለመስጠት የሚውል ንብረት፣ ሀብት ወይም መብት በዚህ አንቀጽ በንዑስ አንቀጽ(፩) መሠረት በዋስትና ሊሠጥ አይችልም።

፶፫. የፋይናንስ ሪፖርትና ኦዲት

፩/ የፕሮጀክት ኩባንያው ከፕሮጀክቱ ጋር በተያያዘ ተገቢ የሆኑ የሂሳብ መዛግብትና መረጃዎችን መያዝ እንዲሁም እነዚህን መዛግብትና መረጃዎች ለተዋዋይ ባለስልጣን ምርመራ ክፍት ማድረግ አለበት::

፪/ የፕሮጀክት ኩባንያው ኦዲት የተደረገውን የሂሳብ መዝገብ እና ተዋዋይ ባለስልጣን በሚያቀርበው ጥያቄ ሌላ ማንኛውንም መረጃ፣ የፋይናንስ አመቱ ባለቀ በስድስት ወር ውስጥ ማቅረብ አለበት።

52. Security Interests

1/ Subject to any restriction that may be contained in the Project Agreements, the Private Party has the right to create security interests over any of its assets, rights or interests, including those relating to the infrastructure project, as required to secure any financing needed for the project, including, in particular, the following:

a) Security over movable or immovable property owned by the Private Party or its interests in project assets;

b) A pledge of the proceeds of, and receivables owed to the Private Party for, the use of the facility or the service it provides.

2/ The shareholders of the Private Party shall have the right to pledge or create any other security interest in their shares in the Private Party.

3/ No security under Sub-Article (1) of this Article may be created over public property or other property, assets or rights needed for the provision of a Public Service Activity where the creation of such security is prohibited by the law.

53. Financial reporting and audit

1/ A Project Company shall keep proper books of accounts and records in relation to the Project and shall be open for scrutiny by the Contracting Authority.

2/ A Project Company shall submit the duly audited financial accounts and any other information as may reasonably be required by the Contracting Authority within six months after the end of each financial year.

፶፬. <u>የፕሮጀክቱን ስምምነት ስለማስተላለፍ</u>

፩/ በዚህ አዋጅ በአንቀጽ (፶፪) እና (፶፯) ከተገለፀው በስተቀር የግል ባለሀብቱ ከፕሮጀክቱ ስምምነት የሚመነጩ መብቶችንም ሆነ ግዴታውን ከተዋዋይ ባለስልጣን ፈቃድ ውጭ ለሌላ ሦስተኛ ወገን ማስተላለፍ አይችልም።

፪/ የፕሮጀክቱ ስምምነት ተዋዋዩ ባለስልጣን ፈቃዱን እንዲሰጥ የሚያስፈልጉ ቅድመ ሁኔታዎችን መያዝ አለበት። መብትና ግዴታው የሚተላለፍለት አዲሱ የግል ባለሀብት መብቱን አስተላላፊው ባለሀብት የገባቸውን ግዴታዎች መቀበሉን እና ለፕሮጀክቱ አስፈላጊ የሆነው የቴክኒክና የፋይናንስ ብቃትን ጨምሮ ሁሉንም መሥፈርቶች የሚያሟላ መሆን አለበት።

፶፭. <u>በሕግ ለውጥ ምክንያት ስለሚከፈል ካሳ</u>

መሠረተ ልማቱን ወይም አገልግሎቱን የሚያስተዳድሩ ሕጎች በመቀየራቸው ምክንያት የግል ባለሀብቱ ግዴታውን ለመወጣት የሚያወጣው ወጪ በከፍተኛ ሁኔታ በመጨመሩ ወይም በመቀነሱ ወይም የግል ባለሀብቱ ከሥራው ጋር ተያይዞ ያገኘው ገቢ ከተገመተው ገቢ ጋር ሲነፃፀር በከፍተኛ ሁኔታ በመጨመሩ ወይም በመቀነሱ ምክንያት የግል ባለሀብቱ ወይም ተዋዋዩ ባለስልጣን ካሣ የሚያገኙበት ሁኔታ በፕሮጀክት ስምምነቱ ውስጥ በግልጽ መመልከት አለበት።

፶፮. <u>የመሠረተ-ልማት ፕሮጀክትን ስለመረከብ</u>

፩/ በመንግሥትና በግል አጋርነት ስምምነት በተመለከተው መሰረት የግል ባለሀብቱ ግዴታውን በአግባቡ መወጣት ሳይችል ሲቀር ወይም በተወሰነ ጊዜ ውስጥ ችግሮቹን ማስተካከል ሳይችል ሲቀር ፕሮጀክቱ ውጤታማ እንዲሆንና

54. <u>Assignment of the Project Agreements</u>

1/ Except as otherwise provided in Article (52) and (57) of this Proclamation, the rights and obligations of the Private Party under the project agreements may not be assigned to third parties without written consent of the Contracting Authority.

2/ The Project Agreement shall set forth the conditions under which the Contracting Authority shall give its consent to an assignment of the rights and obligations of the Private Party under the Project Agreement, including the acceptance by the new Private Party of all obligations there under and evidence of the new Private Party's technical and financial capability as necessary for providing the service.

55. <u>Compensation for Specific Changes in Legislation</u>

The Project Agreements may set forth the extent to which the Private Party or the Contracting Authority is entitled to compensation in the event that the costs of the Private Party's performance of its obligations under the Project Agreements has substantially increased or decreased, or that the value that the Private Party receives for such performance has substantially diminished or increased, as compared with the costs and the value of performance originally foreseen, as a result of changes in legislation or regulations specifically applicable to the Infrastructure Facility or the services it provides.

56. <u>Takeover of an Infrastructure Project by the Contracting Authority</u>

1/ Under the circumstances set forth in the Public Private Partnership Agreement, the Contracting Authority has the right to temporarily take over the operation of the facility for the purpose of ensuring the

ያልተቋረጠ አገልግሎት እንዲሰጥ ለማድረግ ሲባል ተዋዋዩ ባለስልጣን ፕሮጀክቱን ለተወሰነ ጊዜ ተረክቦ ሊያስተዳድር ይችላል።

፪/ ተዋዋዩ ባለስልጣን በዚህ አንቀጽ ንዑስ አንቀጽ (፩)መሠረት ፕሮጀክቱን ለመረከብ በመረጠ ጊዜ፦

ሀ) ፕሮጀክቱ የገባቸውን ግዴታዎች መወጣት እንዲችል በመያዣነት የተሰጡ ማንኛውንም ገቢዎች መሰብሰብና ለተገቡ ግዴታዎች ክፍያ እንዲውል ማድረግ፤

ለ) የፕሮጀክቱን የልማት ስራዎች ማስቀጠል፣ የአገልግሎት ክፍያዎችን መጣል እንዲሁም ቀደም ሲል የነበሩ የአገልግሎት ስምምነቶችን መወጣት፣

ሐ) ለፕሮጀክቱ ግንባታ፣ ጥገና ወይም አስተዳደር የመወዳደሪያ ሀሳቦችን መጠየቅ፣ ይችላል።

፶፯. የግል ባለሀብቱን ስለመተካት

የግል ባለሀብቱ የፕሮጀክት ስምምነቱን ከጣሰ ወይም ስምምነቱን ማቋረጥ የሚያስችሉ ሌሎች ሁኔታዎች ሲያጋጥሙ ወይም ተመሳሳይ ሁኔታዎች ሲከሰቱ ተዋዋዩ ባለስልጣን ለፕሮጀክቱ መሰረተ-ልማት እና ለግል ባለሀብቱ ብድር ከሰጡ የፋይናንስ ተቋማት ጋር የግል ባለሃብቱን በሌላ አዲስ ድርጅት ወይም ሰው ለመተካት ሊስማማ ይችላል።

፶፰. የፕሮጀክት ስምምነት ስለማቋረጥ

በዚህ አዋጅ መሠረት የሚወጣው መመሪያ የፕሮጀክቱ ስምምነት ሊቋረጥ የሚችልባቸውን ምክንያቶች ሊዘረዝር ይችላል።

effective and uninterrupted delivery of the service in the event of serious failure by the Private Party to perform its obligations and to rectify the breach within a reasonable period of time after having been given notice by the Contracting Authority to do so.

2/ In the event that a Contracting Authority elects to take over a project under Sub-Article (1) of this Article, the Contracting Authority:

a) shall collect and pay any revenues that are subject to lien to satisfy any obligation;

b) may develop and operate the project, impose user levies and comply with any service contract existing; and

c) may solicit proposals, as appropriate, for the construction, maintenance or operation of the project.

57. Substitution of the Private Party

The Contracting Authority may agree with the entities extending financing for an infrastructure project and the Private Party to provide for the substitution of the Private Party by a new entity or Person appointed to perform under the existing Project Agreements upon serious breach by the Private Party or other events that could otherwise justify the termination of the Project Agreements or similar circumstances.

58. Termination of the Project Agreements

The directive may prescribe events that may cause the termination of the Project Agreement.

፶፱. ፕሮጀክቱ ስምምነት በተቋረጠ ጊዜ ስለሚከፈል ካሣ

የፕሮጀክቱ ስምምነት በሚቋረጥበት ጊዜ ካሣ ሊከፈለው የሚገባ ወገን በምን መልኩ መካስ እንዳለበት የፕሮጀክት ስምምነቱ ማመልከት አለበት፣ አስፈላጊ በሆነ ጊዜም ስምምነቱ በፕሮጀክት ስምምነቱ መሰረት ለተሰሩ ስራዎች ፍትሀዊ የሆነ ዋጋ፣ ያልተከፈለ ቀሪ ሰዳ፣ በተዋዋይ ወገኖች ለወጡ ወጪዎች ወይም ከሁለቱ በአንዱ ወገን ላይ ለደረሱ ኪሣራዎች፣ እንደአስፈላጊነቱም የታጣ ትርፍ በካሣ መልክ የሚከፈልበትን ሁኔታ ለማካተት ይችላል፡፡

፷. ገዥ ሕግ

የፕሮጀክቱ ስምምነት በኢትዮጵያ ፌደራላዊ ዲሞክራሲያዊ ሪፐብሊክ ሕግ ይገዛል፡፡

፷፩. በተዋዋዩ ባለስልጣን እና በግል ባለሀብቱ መካከል ስለሚፈጠር አለመግባባት

፩/ ማንኛውም በተዋዋይ ባለስልጣንና በግል ባለሀብቱ መካከል የሚነሳ ያለመግባባት፣ ተዋዋዮቹ በፕሮጀክቱ ስምምነት ላይ በተስማሙበት የግጭት መፍቻ ዘዴ መሰረት መፍትሔ ማግኘት አለበት፡፡

፪/ በስራ ላይ ባሉ ሕጎች በተቃራኒ የተደነገገ ቢኖርም የፕሮጀክቱ ስምምነት አለመግባባቶች በግልግል ወይም በአማራጭ የግጭት መፍቻ ዘዴ እንዲፈቱ ሊወሰን ይችላል፡፡

፷፪. ደንበኞችን ወይም የመሠረተ-ልማት ተጠቃሚዎችን የሚመለከት አለመግባባት

የግል ባለሀብቱ ለኅብረተሰቡ አገልግሎት የሚሰጥ ወይም ለሕዝብ የሚቀርቡ የመሠረተ-ልማት አውታሮችን የሚያስተዳድር ከሆነ፣ ተዋዋዩ ባለስልጣን የግል ባለሀብቱ ከደንበኞች ለሚቀርቡ ቅሬታዎች ቀላልና ውጤታማ የሆነ የቅሬታ ማስተናገጃ ዘዴ እንዲተገብር ሊጠይቅ ይችላል፡፡

59. Compensation upon Termination of the Project Agreements

The Project Agreement shall stipulate how compensation due to either party is calculated in the event of termination of the Project Agreement, and where appropriate, for compensation for the fair value of works performed under the Project Agreement, costs incurred or losses sustained by either party, including, as appropriate, lost profits.

60. Governing Law

The Project Agreements are governed by the laws of the Federal Democratic Republic of Ethiopia.

61. Disputes between the Contracting Authority and the Private Party

1/ Any disputes between the Contracting Authority and the Private Party shall be settled through the dispute settlement mechanism agreed by the parties in the Project Agreement.

2/ Notwithstanding any contrary provision in any existing law, the Project Agreements may provide for settlement of disputes through arbitration or any other forms of alternative dispute resolution mechanism.

62. Disputes involving Customers or Users of the Infrastructure

Where the Private Party provide services to the public or operates Infrastructure Facilities accessible to the public, the Contracting Authority may require the Private Party to establish simplified and efficient mechanism for handling claims submitted by its customers or users of the Infrastructure Facility.

፷፫. **ሌሎች አለመግባባቶች**

፩/ የግል ባለሀብቱ እና ባለአክሲዮኖች በመካከላቸው ለሚነሳው አለመግባባት ማንኛውንም የግልግት መፍቻ ዘዴ መምረጥ ይችላሉ።

፪/ የግል ባለሀብቱ ከአበዳሪዎቹ፣ ከሥራ ተቋራጮቹ፣ ከአቅራቢዎቹ እና ከሌሎች የሥራ አጋሮቹ ጋር የሚያጋጥሙትን አለመግባባቶች ለመፍታት የፈለገውን የግልግት መፍቻ ዘዴ የመምረጥ ነፃነት አለው።

ምዕራፍ አስራአንድ
ልዩ ልዩ ድንጋጌዎች

፷፬. **አቤቱታዎች**

በኢትዮጵያ ፌደራል መንግሥት የግዥና የንብረት አስተዳደር አዋጅ የተደነገገው የአቤቱታ ማቅረቢያ እና የውሣኔ አሰጣጥ ዘዴ እንደሁኔታው ማስተካከያ ታክሎበት ለዚህ አዋጅ ተፈፃሚ ይሆናል። በዚህ አዋጅ መሰረት የሚወጣው መመሪያ እነዚህን ድንጋጌዎች ለመንግሥትና ለግል አጋርነት ተስማሚ በሚሆኑበት አኳኋን ማጣጣም ይችላል።

፷፭. **ደንብና መመሪያ የማውጣት ሥልጣን**

፩/ የሚኒስትሮች ምክር ቤት ይህን አዋጅ ለማስፈጸም ደንብ ሊያወጣ ይችላል።

፪/ ሚኒስቴሩ የዚህን አዋጅ ዓላማዎች ከግብ ለማድረስ እና በዚህ አንቀጽ ንዑስ አንቀጽ (፩) መሠረት የወጡትን ደንቦች ለማስፈፀም የሚረዱ መመሪያዎችን ያወጣል።

፷፮. **የተሻሩና ተፈፃሚ የማይሆኑ ሕጎች**

፩/ በዚህ አዋጅ ከተመለከቱት ውስን ድንጋጌዎች በስተቀር፣ የኢትዮጵያ ፌደራል መንግሥት የግዥና የንብረት አስተዳደር አዋጅ ድንጋጌዎች በመንግሥትና በግል አጋርነት ላይ ተፈፃሚነት የላቸውም።

63. **Other Disputes**

1/ The Private Party and its shareholders shall be free to choose the appropriate mechanism for settling disputes among themselves.

2/ The Private Party shall be free to agree on the appropriate mechanism for settling disputes between itself and its lenders, contractors, suppliers and other business partners.

CHAPTER ELEVEN
MISCELLANEOUS PROVISIONS

64. **Complaints**

The complaints and review mechanism provided under the Ethiopian Federal Government Procurement and Property Administration Proclamation, as they may be amended or replaced, shall be applicable mutatis mutandis to this Proclamation. Directives issued in accordance with this Proclamation may provide for an adaptation of these articles as to apply to Public Private Partnership projects.

65. **Power to issue Regulation and Directives**

1/ The Council of Ministers may issue regulation for the implementation of this Proclamation.

2/The Ministry shall issue directives enabling the realization of the objectives of this Proclamation and for the proper implementation of the directives issued in accordance with sub article 1 of this Article.

66. **Repealed and Non-Applicable Laws**

1/ Except for specific provisions referred to in this Proclamation, the provisions of the Ethiopian Federal Government Procurement and Property Administrat ion Proclamation, as may be amended, are inapplicable to Public Private Partnership projects.

፪/ ይህን አዋጅ የሚቃረን ማንኛውም ሕግ፣ ደንብ፣ መመሪያ ወይም የአሠራር ልምድ በዚህ አዋጅ በተሸፈኑ ጉዳዮች ላይ ተፈፃሚነት አይኖረውም፡፡

፷፯. **የመሸጋገሪያ ድንጋጌዎች**

ይህ አዋጅ ከመፅናቱ በፊት በተፈረሙ ወይም በድርድር ላይ ባሉ የመንግሥትና የግል አጋርነት ስምምነቶች ላይ የዚህ አዋጅ ድንጋጌዎች ተፈፃሚነት አይኖራቸውም፡፡

፷፰. **አዋጁ የሚፀናበት ጊዜ**

ይህ አዋጅ በፌደራል ነጋሪት ጋዜጣ ታትሞ ከወጣበት ቀን ጀምሮ የፀና ይሆናል፡፡

አዲስ አበባ የካቲት ፲፭ ቀን ፪ሺ፲ ዓ.ም.

ዶ/ር ሙላቱ ተሾመ

የኢትዮጵያ ፌደራላዊ ዲሞክራሲያዊ ሪፐብሊክ ፕሬዚዳንት

2/ No law, regulation, directive or practices inconsistent with this Proclamation shall have effect with respect to matters provided for in this Proclamation.

67. **Transitory Provisions**

This Proclamation shall not apply to existing public private partnership projects where contracts have already been concluded or are under negotiation.

68. **Effective Date**

This Proclamation shall enter into force on the date of its publication in the Federal Negarit Gazette.

Done at Addis Ababa, this 22nd day of February,2018.

MULATU TESHOME (DR.)

PRESIDENT OF THE FEDERAL DEMOCRATIC REPUBLIC OF ETHIOPIA

ብርሃንና ሰላም ማተሚያ ድርጅት
