# Supplementary material for: Range of hurdles and opportunities for developing PPPs in diagnostics: a contextual analysis, Ethiopia
Source: BMC Health Serv Res. 2026 Mar 28;26:651. doi: 10.1186/s12913-026-14354-z (PMC13151300; doi:10.1186/s12913-026-14354-z)
Supplement: Supplementary file 2 — Supplementary Material 2 [file 12913_2026_14354_MOESM2_ESM.pdf]

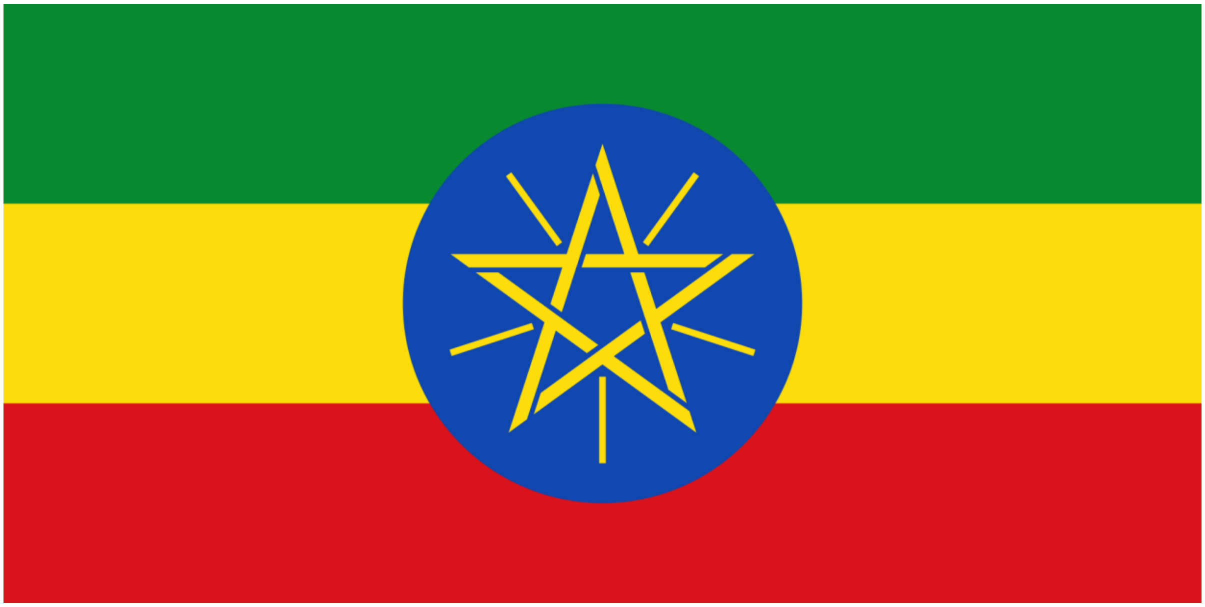

Policy for the Use and Implementation of Public-Private Partnerships

Supporting Economic Development in Ethiopia

Federal Democratic Republic of Ethiopia

August, 2017

FDRE Ministry of Finance and Economic Cooperation

Table of Contents

1. OVERVIEW 1
2. POLICY CONTEXT 3
GTP II and Vision 2025 3
Infrastructure Gap and Investment Need 3
Investment Policy 4
Economic Stability 4
3. OBJECTIVES 5
Objectives for the Use of PPPs 5
Objectives for the PPP Framework 6
4. GOVERNMENT COMMITMENT 7
5. SCOPE AND APPLICATION 10
Definition of Public-Private Partnership 10
Coverage of the Policy 10
Criteria for PPPs 11
6. GOVERNANCE 14
Roles and Responsibilities 14
Government Participation and Support 16
Process 17

1 OVERVIEW

The Government of Ethiopia ("Government") has targeted the achievement of middle income status by 2025 through the application of development policies and strategies that enable rapid, broad-based, sustainable and equitable economic growth. Considerable success has been shown to date through the double digit economic growth that has been over the last decade. This is achieved due to the fact that several development projects planned under the first phase of the GTP have been completed successfully through government financing. Similarly, the government paid the same attention for the execution of the projects and programs under the second phase of the GTP ("GTP2") by allocating a huge amount resource. However the GTP2 implied that government financing alone cannot meet the growing demand for public services sustainably.

The government understands the need for mobilizing resources from different sources other than the traditional government financing in order to meet the growing demand for public services. Particularly, involving the private sector in partnership with the government has been used by governments to fill the gap in infrastructure financing. Public Private Partnership (PPP) is used as one of the tools to improve infrastructure services and if appropriately utilized gives the opportunity to use the private sector investment potential as required.

The Ministry of Finance and Economic Cooperation has taken the initiative to design the Public Private Partnership program to realize the government's partnership with the private sector. This policy is about the utilization of the partnership and is implementation policy where preparation of the legal framework is taken as the priority activity. The policy apart from being a document by which a direction is given to the way PPP projects are implemented in the country, it helps on the implementation of the program in a transparent way.

The PPP framework is about documents and administrative institutions that facilitate the public private partnership. Proclamations and regulations that for instance explain the scope and utilization, identification and preparation of projects to be financed under PPPs, and identify key stakeholders and institutions that are established to follow the program in a special focus are included under this framework. Moreover, guidelines on the implementation of PPP projects and manuals and regular bid documents are part of the framework.

It is oblivious that taking PPPs as an option to ensure infrastructure provisions is new for Ethiopia. And hence it is important that a focus is given to few infrastructure projects carefully chosen as test at the first few years. Starting with infrastructure projects identified as priority in the second phase of the GTP will be advantageous. The criterion

for selecting the test projects should be the potential in enhancing the capacity of the government and their potential that lessons are drawn for future when PPP projects are implemented in a larger scale. The PPP framework apart from taking into account the important exhibitions of international experiences of PPP, it is expected that it will be developed in a way that it captures the lessons in the life of the program from time to time. This aims at drawing lessons for the program from the experience of other countries and to put good governance and a transparent PPP program in place starting from the beginning. Hence Ethiopia’s decision to embark on PPP program to support its overall development vision emanates from the understanding of the use of the program and the strong position the government has followed the need for developing a legal framework to utilize the benefits of the program.

2 POLICY CONTEXT

GTP II and Vision 2025

Rapid, broad-based, sustainable and equitable economic growth is a key objective of the Government as it supports the country's vision of reaching middle-income status by 2025. During the past decade, Ethiopia has achieved such growth, averaging approximately 11 percent per annum. Strategic planning has been useful in both sustaining and directing this growth to achieve targets in specific areas.

In 2010, Ethiopia embarked on a five-year Growth and Transformation Plan ("GTP I"), which included seven strategic pillars highlighting the key areas of focus to drive desired economic growth. A number of pillars focused on strengthening areas such as social development, good governance, and promoting gender and youth equity, as well as making improvements in areas of existing capacity, such as agriculture. A focus on infrastructure development was also included specifically to address weaknesses in Ethiopia's existing infrastructure stock. The implementation of GTP I proved to be a success with the Government achieving many of the economic, infrastructure, and social targets over the five-year plan period.

Hence, several projects that have the basis from the pillars of the GTP II are designed. Since the investment, technology and skill requirments of the projects are high, the government has envisaged to involve the private sector in a large scale and Public Private Partnership is one of the modes by which the plan is realized.

Infrastructure Gap and Investment Need

Ethiopia has acknowledged in her policies and strategies the well-established link between access to infrastructure and economic growth and development. Access to infrastructure underpins economic growth through increasing an economy's competitiveness and enabling higher private sector productivity. Investment in physical infrastructure reduces the cost of the production and transportation of goods and services and facilitates the mobility of people, while investment in social infrastructure and other public services improves education, health and other development outcomes. This investment is even more important in light of infrastructure access and coverage indicators in Ethiopia that highlight critical needs for infrastructure development.

GTP II recognizes the role infrastructure plays as a catalyst for economic growth and development by including infrastructure development as strategic pillar. Specific

infrastructure development targets include the expansion of transport infrastructure (roads, rail) and logistic services; the further development of the Addis Ababa airport; an expansion in energy production, transmission and distribution networks; and, extension in water supply service coverage and irrigation infrastructure. In addition, a number of goals in other strategic pillars will also be directly supported by the provision of improved infrastructure service. These infrastructural building activities cannot be implemented solely from the government sources. And hence it will wise to involve the private sector in infrastructure services.

Investment Policy

A second five-year plan, GTP II, was approved in December 2015. Although GTP II continues the broad policy focus of GTP I, it also includes an increased emphasis on the transformation of the current, predominantly agrarian, economy to a more modern, industrialized economy. As a result, it includes strategic pillars associated with manufacturing sector development, infrastructure development, and capacitation of the local private sector, among others. GTP II envisions investments of $150 billion over the next 5 years. Similar investment requirements are expected to continue, beyond the GTP II planning horizon, in order to reach the target of achieving middle-income status. Although much of this investment is expected to come from domestic sources, GTP II also seeks to leverage increasing foreign direct investment (“FDI”) in addition to borrowing expectations, an approach that will continue to be relevant over the longer-term.

Hence, the use of PPPs may serve as a tool to assist in attracting the necessary FDI to support the implementation of our development plans.

Economic Stability

Achieving economic growth is one of the goals of the development and implementation of infrastructure projects highlighted in GTP2. However, the implementation of these projects alone is not sufficient to ensure that economic stability accompanies desired economic growth. As a result, the Government is also focused on the prudent management of public debt. PPPs are seen as a tool that can support the Government in managing the level and timing of government expenditure.

3 OBJECTIVES

The Government has a number of overarching goals associated with the implementation of its PPP Policy. These include:

- Expanding public service activities
- Reduction in project delays and cost overruns;
- Increased utilization of resources from the private sector on public services

Objectives for the Use of PPPs

The objectives for the use of PPPs to support the implementation of individual projects are two-fold:

- To increase the financial resources available for the development and delivery of infrastructure services in Ethiopia through leveraging private sector investment and management; and,

- To capture the benefits of private sector involvement in infrastructure development through the alignment of public and private sector incentives and the use of appropriate risk transfer, where such benefits exist. These benefits may include, but are not limited to, the following:
  - Innovations in design or service delivery approach;
  - Reduction in project delays and cost overruns;
  - Implementation of a life-cycle approach to infrastructure service costing;
  - Improved quality or efficiency;
  - Knowledge transfer to local private sector; and,
  - Increased asset utilization.

The Government has designed a number of specific objectives to support the achievement of these overarching goals.

Objectives for the PPP Framework

The Government's interest in the use of PPPs as an infrastructure delivery mechanism goes beyond facilitating initial pilot projects, and is expected to extend to a broader range of public services that could benefit from private sector experience and investment. In order to benefit from this broader use of PPPs, the Government intends to take a programmatic approach to their use, both over-time and across sectors. This approach to the development and implementation of PPPs would be captured in the development of the PPP Framework. Such PPP Frameworks that define policy, establish enabling legislation and set out detailed procedures that will be followed in the development and implementation of projects have been found to be an important factor in the success of PPP Programs worldwide.

The objectives of implementing such an approach are as follows:

- To promote an enabling environment to facilitate enhanced and systematic private investment in public infrastructure;
- To capture and share knowledge on the implementation of PPP projects, from both international and domestic experience, in order to support the development of successful PPP models in Ethiopia;
- To provide clarity and consistency for both public and private sector parties with respect to the roles and responsibilities of all parties;
- To facilitate the project development process through providing clear guidance on the process for the development, implementation and monitoring of PPPs, including the selection of private sector partners; and,
- To provide for objective evaluation of the use of PPPs in Ethiopia to support the evolution of the PPP Framework and the implementation of PPP projects.

4 GOVERNMENT COMMITMENT

The Government is committed to meeting the infrastructure and public service development needs of the country, and in doing so, to utilize approaches to infrastructure delivery that best leverage available resources, including private investment. The choice of delivery approach must therefore center on how to best meet development needs given existing constraints and will necessarily involve the consideration of many factors. Therefore, the Government sets out below its commitment with respect to the principles that will underpin its decisions associated with the development of the PPP Framework and individual PPP projects.

Protection of Public Interest

PPP projects provide infrastructure services that enable the public to carry out their day to day responsibilities and lead their lives. These services therefore directly affect the welfare of end-users of the service. Beyond end-users, the development of infrastructure projects tends to affect the welfare of the general public in a variety of ways. Notably, these effects may include impacts on the environment that need to be mitigated and managed appropriately, but may also address issues of health and safety, privacy, public access and equity that are associated with the development of infrastructure necessary to provide a service or the provision of service itself. Given this, the Government commits to consider the public interest associated with the implementation of any PPP.

Private Sector Participation

In enabling the use of PPPs, the Government is explicitly recognizing that private sector participation in the delivery of infrastructure service can, in the right circumstances, deliver value beyond public sector service delivery alone. As a result, it is the Government's intention to promote the participation of the private sector in infrastructure service delivery wherever such value can be realized. The Government further recognizes that the added value of private sector participation in infrastructure service delivery can be achieved in a number of different ways. Value addition is most likely to be achieved through improved service or reduced life-cycle costs associated with individual projects. However, the Government may also consider value addition to the broader PPP program or infrastructure development agenda, as a part of its programmatic approach. Examples of this could include a project that a) reduces barriers for the implementation of other similar projects or b) frees up resources to speed the delivery of other PPP or non-PPP projects.

Project Selection

Although the use of PPPs may deliver benefits beyond the traditional provision of infrastructure services by the public sector, these benefits can only be captured when PPPs are appropriately designed based on infrastructure needs as well as local circumstances and constraints. `Ensuring that projects selected for delivery though PPPs can be structured appropriately is therefore of considerable importance. This is even more relevant in the Ethiopian context, where there is little experience with PPPs to date. As a result, the Government expects that its use of PPPs will initially be selective and will focus on projects where the Government's objectives for the use of PPPs can be most clearly met. As public sector capacity to implement PPPs grows, a wider range of projects may be considered for implementation as PPPs.

In determining which projects may be undertaken as PPPs, the Government commits that its decisions will be based on a clear evaluation process that involves a rigorous assessment of the various costs and benefits of individual projects under different delivery approaches. Such evaluation will necessarily include an assessment of the risks retained by the Government and the financial and human capital implications of managing these risks over the life of the project.

Governance

PPPs involve the transfer of certain roles and responsibilities typically held by the public sector to a private sector partner. However, it is important to note that, regardless of the nature of the project, the Government shall retain ultimate accountability to its constituents with respect to its obligation to develop and deliver infrastructure service. PPPs are a tool the Government may leverage to meet its obligations more efficiently, but accountability for service delivery outcomes cannot be delegated. As a result, the public sector will maintain a central role in the definition of the function of the private sector in a particular project and the ongoing monitoring of performance of the private sector partner. Beyond accountability for individual projects, the Government is also accountable for its use of resources in the development of PPPs. The Government, therefore, commits to assess the use of PPPs by considering the overall benefits to the country of various PPP projects against the overall Government resources consumed to deliver these projects.

Selection of Private Sector Partner

Due to the far reaching impacts of the implementation of a long-term contract for the delivery of critical public infrastructure service, the selection of the private sector partner is fundamental in driving the value of private sector involvement for the Government. While different approaches to selection may be appropriate across the various types of projects considered as PPPs, the Government commits to both articulate and implement a clear, consistent and transparent system for the selection of a private sector partner

that encourages competition. The PPP Framework is expected to capture the legal requirements for procurement of PPP and provide considerable guidance to support the selection of qualified private sector partners.

5 SCOPE AND APPLICATION

The PPP Framework is expected to apply to a broad range of projects, as well as a number of public entities. This section describes the Government institutions expected to be bound by the PPP Framework, the general categories of projects eligible to be considered under the PPP Framework and the criteria that such projects must meet to be designated as PPPs.

Definition of Public-Private Partnership

PPPs are defined as long-term agreements between a Contracting Authority and a third party where:

- the third party provides or contributes to the provision of a Public Service Activity;
- the third party receives a revenue stream for providing such service from end-user charges, Government budget, or a combination of the two; and,
- this revenue stream must be based on the availability and quality of the contracted service, thereby transferring significant risk associated with the provision of the Public Service to the third party.

Public Service Activities are defined as those which the Government has decided to perform for the reason that it has deemed such services to be in the general interest and considered that private initiative was inadequate for ensuring the appropriate level and quality of service.

Coverage of the Policy

Contracting Authorities who are subject to the law include:

- Public Bodies which include any organ of the Federal Government which is partly or wholly financed by Government allocated budget;
- Publicly Enterprises which include any organization where the Federal Government has a significant ownership interest and is undertaking a project that meets the definition of a PPP; and,

Based on this policy the Regional Sates may issue their own implementation framework to implement PPP projects.

Criteria for PPPs

Projects undertaken by Contracting Authorities that meet the general definition of PPP must still be evaluated to ensure that a PPP delivery approach is the most appropriate. This approach recognizes that a large number of services could be delivered as PPPs, but that the ability to deliver a project as a PPP does not indicate that a PPP delivery approach is the most economically advantageous to Ethiopia. This may be particularly true in the early years of PPP program development, when capacity within the Government to deliver PPP projects is limited. As a result, projects to be delivered using a PPP approach will need to be studied in sufficient detail to address the extent to which they meet certain criteria. An overview of key criteria for consideration is provided below. Guidance on how to undertake such studies is expected to be provided in the PPP Framework.

Value for Money

As highlighted in the Government Commitment section, private sector participation in the delivery of infrastructure service can deliver value beyond public sector service delivery alone. However, the involvement of the private sector alone does not guarantee that such value will be generated, or that this value is sufficient to justify the associated costs to government. Projects where private sector involvement is expected to generate benefits sufficient to justify their implementation as PPPs are deemed to deliver Value for Money ("VfM") and demonstration of this expectation is a requirement for projects to use the PPP delivery approach.

VfM is generally derived by comparing expected cost to government for the implementation of the project under a PPP delivery approach to the expected cost for delivering the same project under a traditional public procurement approach. A wide variety of estimated benefits and costs may be considered in determining VfM and further guidance on how to evaluate VfM will be laid out in the PPP Framework. However, it is important to recognize that the costs to government associated with individual projects may consider both financial and economic costs and benefits.

It is also important to note the importance of the impact of a project's risk allocation on VfM. In order to maximize VfM, risks should be allocated to the party best able to manage them. This reduces the overall cost to government associated with the delivery of the project. To calculate VfM, therefore, risks associated with a project's implementation must be considered. These include both risks the Government has transferred, for example the value of avoiding responsibility for a particular cost overrun, as well as risks the Government has fully or partially assumed, for example the provision of a payment guarantee which may or may not be called.

Given the expected costs of PPP project development, projects that have contract values of greater than USD [50] million are generally those that are expected to be most likely to generate VfM. However, projects below this threshold may also be suitable for PPP delivery if the project exhibits sufficient potential to deliver VfM.

Affordability

PPP agreements frequently represent a long-term commitment of government resources or the resources of end-users who pay for the service, or some combination of the two. Regardless of the source of revenue to the private party, such revenue stream must be evaluated to ensure that it is within the means of the government and end-users to meet any payment obligation. As a result, studies investigating the delivery of projects through a PPP approach should include appropriate projections of expected tariff and/or expected government budget allocation. These projects should be compared against existing constraints such as end-user ability and willingness to pay and the expected availability of government funds. If end-user ability or willingness to pay is insufficient, government budget may be used to reduce end-user tariffs where appropriate.

Public Interest

Consistent with the Government's commitment to ensure protection of the public interest, individual projects should be studied to understand the ways in which issues of public interest may be impacted over the life of the project. This may include an evaluation of the environmental, health and safety impacts of the development or delivery of a particular public service, along with other issues that may have a bearing on the welfare of the general public or end-users of the service. These impacts should be clearly presented so that the Government can make an informed determination with respect to the protections that may need to be put into place in order to ensure the safe and equitable implementation of the project in accordance with Ethiopian law.

Sustainability

Given the long-term nature of PPP agreements, PPP projects will need to weather many changes in circumstance, both foreseen and unforeseen. To the extent possible, PPP agreements are written to enable flexibility to allow parties to adapt to changing situations. However, the best assurance of sustainability is a project which has the potential to deliver benefits to both parties over its life. When both parties are truly benefitting from the deal, each will strive to fulfill the spirit of the agreement despite future changes. For the Government, this means not only considering whether the quality and price of an infrastructure service is acceptable, but also ensuring that the private party will have an opportunity to earn an appropriate profit given the risk it has assumed. A government's commitment to structuring win-win agreements will also serve

to increase private sector interest in the project, thereby driving a competitive selection process and improving VfM. For both reasons, the assessment of the attractiveness of the project to private sector parties is an important step in determining whether a project can be successfully implemented through a PPP approach.

Institutional Capacity

PPP agreements are both complex and time consuming to prepare and also impose long-term commitments on the Government. These commitments fall into two broad categories, which are:

- fulfilling government obligations under the agreements, which may include among others: providing land, connecting infrastructure, making timely payments, or adjusting tariffs over time; and
- monitoring the performance of the private sector's obligations under the agreement.

Achieving VfM is extremely dependent on both how well prepared a project is and how well the Government meets its ongoing commitments. Therefore, it is important to clearly understand the institutional capacity that will be required during the project preparation stage and throughout the life of the project. A realistic plan demonstrating how these issues will be managed by the Contracting Authority should form an integral part of the Government's evaluation of whether a PPP delivery approach is appropriate.

6 GOVERNANCE

In order to successfully design, evaluate, implement, and monitor PPP projects, the Government will need to engage in a number of coordinated actions. The PPP Framework is intended to create consistency and clarity around the roles and responsibilities of various parties, as well as the process through which various actors will need to be engaged. This section highlights the key responsibilities associated with the development and implementation of PPPs. The PPP Framework will provide more detailed guidance.

Roles and Responsibilities

PPP Board

A PPP Board shall be formed to grant approvals at key milestones in the project development process. Specifically, the PPP Board shall have the responsibility of approval of PPP pipeline projects, approving a PPP project prior to tender and prior to award. In such cases, approval indicates that the proposed project structure, including the risks allocated to the public and private sectors and any proposed government support or participation, is considered to be economically advantageous to Ethiopia. Prior to tender, in cases where government support requires additional approval beyond that of the PPP Board, the PPP Board shall recommend the project to the appropriate authority for review. The authorities that may be required to approve government support include the National Bank, the Investment Board and, in cases where federal budget is committed to a project, the Parliament.

Further approval of the PPP Board or other authorities in the case of government support would also be necessary upon any material change to the project structure or government support. In addition, the Board shall have the power to instruct a Public Body or Public Enterprise to use a PPP approach for the delivery of a specific projects.

The PPP Board shall be chaired by the Minister of Finance and Economic Cooperation and shall comprise other relevant Ministers and public officials as standing members, as well as members from the private sector. The Contracting Authority and other parties relevant for individual projects will be invited to join the Board on an ad hoc, non-voting basis. The Board is intended to both represent the interests of the Contracting Authority, but also the broader Government. The Board shall be supported in its duties by the PPP Unit, who shall serve as its secretariat.

Ministry of Finance and Economic Cooperation

MoFEC has several key roles associated with the development and implementation of PPP Projects. As the owner of the PPP Framework, MoFEC, as supported by the PPP Unit, is responsible for its management and development and serves as the key driver of Government policy with respect to the use of PPPs.

With respect to individual projects, MoFEC has several key responsibilities which include:

- the designation of a project as a PPP – this enables MoFEC to provide guidance to Contracting Authorities on the Government's policy for pursuing PPPs prior to undertaking significant studies associated with the PPP development of a project. This will be done in consultation with the National Planning Commission;
- the approval of any proposed Government Support or Participation within its jurisdiction, particularly as it relates to any request to provide a guarantee for payment obligations of the Contracting Authority; and,
- Chairmanship of the PPP Board.

PPP Unit

A PPP Unit will be established in MoFEC where it will complement other functions central to the development and implementation of PPPs, such as procurement and project budgeting. This unit will have the overarching goal of supporting good practice and consistency across the development and delivery of PPP projects in Ethiopia. The PPP Unit's responsibilities will include:

- Supporting MoFEC in the management and development of the PPP Framework
- Supporting the development of a PPP program and individual PPP projects through by providing assistance:
    - in the identification and analysis of projects likely to deliver VfM
    - to Contracting Authorities in the development, implementation and monitoring of PPPs
    - to the PPP Board in evaluating and approving PPP projects.
- Collecting and disseminating information on the PPP program in Ethiopia and on PPP experience and best practice more generally
- Promoting the participation of the private sector in the delivery of infrastructure services.

In order to fulfill its responsibilities, the PPP Unit will require staff with a range of technical, legal and financial skills. Given the importance of building strong institutional capacity to the overall success of PPP projects and the PPP program, attracting and retaining staff of an appropriate caliber will be critical. To this end, the Government will ensure that it has the means to employ appropriate staff and may also rely on external advisors for both delivering some of the services of the PPP Unit and capacitating domestic PPP Unit staff.

Contracting Authority

The Contracting Authority is the entity that is legally empowered to provide the service that is being considered for private sector participation. The Contracting Authority has ownership of the project and is responsible for its development and implementation. Also, as is consistent with current practice in Ethiopia, the Contracting Authority shall have the responsibility for implementing a procurement process in accordance with law.

In order to fulfill these responsibilities, the Contracting Authority is expected to form a Project Team to manage the development and implementation of any PPP project. The head of the Contracting Authority is responsible for appointing the Project Team; however, a PPP Unit member is to be included on the Project Team in order to facilitate the sharing of capacity related to PPP project development and implementation. The Contracting Authority may also retain any consultants or advisors as necessary to support the Project Team in fulfilling its duties.

Government Participation and Support

Beyond the critical roles of development and regulation of PPP projects, governments also play other roles in PPP projects, notably providing various types of support or participating more directly in project implementation. The Government wants to enable a variety of forms of government participation and support in order to ensure that it will have all available tools at its disposal in the future; however, the use of some forms of government participation or support will require careful evaluation on the part of the government. The determination of whether these types of government involvement are merited is best made on a case by case basis. In making such determination, the Government will take into account:

- what type of involvement is required for successful project implementation;
- whether the project's expected benefits merit this involvement; and,
- any constraints which may exist to the desired approach.

The PPP Framework will provide additional guidance on the circumstances which might merit various types of government participation and support; however, in general these methods may include:

- **Project facilitation:** PPP projects interact with many different Government bodies across their life-cycle. The Contracting Authority or broader Government may agree to facilitate, where possible, these interactions in order to reduce time and cost.
- **In-kind support:** the provision of non-cash support to facilitate the implementation of the project. This may include making land available or providing easements needed to implement the project or providing incentives like income tax or duty exemptions.

- **Project investment:** the provision of either debt or equity to support the development of the project.
- **Capital support:** the provision of funding, generally early in the development of a project, in order to reduce private financing requirements, possibly with the goal of reducing ongoing payments or tariffs.
- **Contracted payments:** the provision of payments over the life of the contract, either to pay for service or as otherwise specified in the contract.
- **Credit enhancement:** the use of a variety of mechanisms to provide comfort that the Contracting Authority will meet its contractual financial obligations. This may include the use of sovereign guarantees to support these contractual obligations.

Process

Identification

Project identification is in general expected to be undertaken by Contracting Authorities as a part of their normal infrastructure planning process. Ideally, PPP projects would be selected from those projects already defined and identified as priorities, but where the development and implementation of that project can be improved through the involvement of a private sector partner. In some cases, the PPP Unit may also recommend that a particular project be implemented as a PPP project, subject to agreement by the Contracting Authority, where it believes significant value can be gained through the use of a PPP delivery approach. In all cases, a project should be formally designated as a PPP, thereby indicating that the procedures and policies applicable to PPP projects will apply. Such a designation should be approved by the Minister of Finance, who may delegate such decision to the PPP Unit head given certain project characteristics. To the extent that funding is available to support the development of PPP feasibility and structuring work, the designation of a project as a PPP would enable it to access such support.

Feasibility and Structuring

The Project Team formed by the Contracting Authority shall carry out all necessary project due diligence, which will, at a minimum, include thorough technical, legal and financial analysis. In general, this analysis will include projections of project capital and operating costs under various scenarios and will be used to inform the development of a project structure that will also capture:

- proposed responsibilities of the public and private sector parties;
- risks that each party is expected to assume and how risk will be transferred to the private sector; and,
- proposed government participation and support, if any.

Following the completion of this stage, the Contracting Authority should be able to demonstrate to what extent the project addresses the Criteria for PPPs highlighted in Section 3.

Approval of Structure

Based on the proposed project structure, the PPP Board will conduct its review, supported by the PPP Unit. This review shall be focused on the extent to which the project has met the Criteria for PPPs and the ability and willingness of the Government to accept any payment or other obligations or risks that are imposed by the Project. The approval of the PPP Board, combined with any other approvals required for government support, indicates the willingness of the Government to proceed with the project on the basis of the project structure and its expected financial impact including any government support or participation. Effectively, such approval indicates that the proposed allocation of risks and responsibilities is deemed to be economically advantageous to Ethiopia. Once approval has been given, the Project Team may begin the selection of of the private sector partner. The PPP Board may delegate such decision to the PPP Unit head given certain project characteristics.

Selection of Private Partner

The Contracting Authority has the responsibility of selecting a private sector partner in accordance with the provisions of the PPP law relevant to procurement. In general, this is expected to be through a transparent, competitive tender process conducted to identify the proposal which presents the most economically advantageous option for Ethiopia. While the nature of the tender process may vary depending on the project in question, PPP procurement processes are typically expected to involve a pre-qualification phase, followed by a bid submission phase where only prequalified bidders may participate. During this bidding phase, draft PPP project agreements are generally made available for review and comment by all prequalified bidders and a final draft is submitted to bidders prior to bid submission with the goal of obtaining readily comparable bids. It is important to note that if project agreements are modified materially in terms of risk allocation or expected cash flow to or from the Government, further approval of the PPP Board may be required.

In the event that the Government receives unsolicited bids, a competitive process should be pursued except where otherwise prescribed by law. Further guidance on options to introduce competition in situations of unsolicited bids, as well as on PPP procurement options in general, will be provided as a part of the PPP Framework.

Award and Signing

Following the selection of the winning bidder, the Contracting Authority will seek approval of the evaluation and final agreements from the PPP Board. If there has been

any material change to proposed government support, any authorities that provided approvals prior to tender will also need to be reviewed and approved. Following approval, the Contracting Authority will formally give notice of the award and accordingly prepare for the signing of the PPP Contract. Although negotiation after award should be minimized, some changes to the project agreements may be necessary, particularly with respect to lender participation. Should the project risk allocation or expected cash flows change materially from what was previously approved by the PPP Board, further approval may be necessary. After obtaining any further PPP Board approval, if necessary, the Contracting Authority and any other Government signatories are then authorized to sign the PPP agreements in order to begin the implementation stage of the project, including taking any steps necessary to support the private partner in reaching financial close.

Implementation and Monitoring

The Contracting Authority is responsible for the implementation and monitoring of the project with respect to the performance of the private sector party and the overall extent to which the project is satisfactorily meeting service delivery needs. To facilitate this process, the Project Team who developed the project will prepare guidelines for management of the PPP agreements which highlight ongoing Government responsibilities under the project agreements, both in terms of financial obligations as well as performance monitoring requirements. These contract management guidelines will then be used by Project Team members, and their successors, to oversee the contract over the life of the PPP project.
