# Supplementary material for: Range of hurdles and opportunities for developing PPPs in diagnostics: a contextual analysis, Ethiopia
Source: BMC Health Serv Res. 2026 Mar 28;26:651. doi: 10.1186/s12913-026-14354-z (PMC13151300; doi:10.1186/s12913-026-14354-z)
Supplement: Supplementary file 3 — Supplementary Material 3 [file 12913_2026_14354_MOESM3_ESM.pdf]

ገንዘብ ሚኒስቴር
MINISTRY OF FINANCE

Preparation of General and Sector Specific Implementation Guidelines for Public Private Partnerships

PPP GUIDELINES

Acknowledgements

The CPCS Team acknowledges and is thankful for the input and guidance provided by the PPP Directorate General (PPPDG), the World Bank Group, and other stakeholders consulted in the preparation of these Guidelines.

Table of Contents

1 PPP PRIMER AND PHASES IN PPP DEVELOPMENT 5

1.1 DEFINITION OF PPPs 5

1.1.1 WHAT IS A PPP? 5

1.1.2 DEFINITIONS 6

1.1.3 WHY USE PPPS 8

1.1.4 WHEN SHOULD A PPP BE USED? 10

1.1.5 PPPS IN ETHIOPIA 12

1.2 RISK: A CRITICAL FOCUS OF PPP DESIGN 12

1.2.1 MAJOR RISKS IN INFRASTRUCTURE PPPS 12

1.3 OVERVIEW OF PPP MODEL VARIANTS 15

1.3.1 PROJECT CHARACTERISTICS THAT AFFECT THE CHOICE OF PPP MODEL 15

1.3.2 FORMS OF PPP 17

1.3.3 RISK ALLOCATION UNDER DIFFERENT PPP MODES 22

1.4 OVERVIEW OF PPP DEVELOPMENT PROCESS 24

1.4.1 PPP DEVELOPMENT LIFE CYCLE 24

1.4.2 PPP PROCESS MAP 25

2 ROLES AND RESPONSIBILITIES 26

2.1 PARTIES INVOLVED IN THE PPP PROCESS 26

3 PHASE I: PPP PROJECT IDENTIFICATION AND SUITABILITY ANALYSIS 32

3.1 PROJECT IDENTIFICATION 36

3.1.1 PPP DIRECTORATE GENERAL 36

3.1.2 PUBLIC ENTITIES 36

3.1.3 UNSOLICITED PROPOSALS FROM THE PRIVATE SECTOR 36

3.2 PPP SUITABILITY AND PRELIMINARY APPROVAL PROCESS 40

3.2.1 PPP SUITABILITY EVALUATION 41

3.2.2 PRE-FEASIBILITY STUDY 44

3.2.3 APPROVAL TO PROCEED TO FEASIBILITY STUDY BY THE PPP BOARD 46

3.3 ACTIONS UPON APPROVAL BY THE PPP BOARD TO PROCEED TO FEASIBILITY STUDY (NEXT STEPS) 46

3.3.1 UPDATE PPP PIPELINE 46

3.3.2 APPOINTING THE PROJECT MANAGEMENT TEAM 47

3.3.3 ALLOCATION OF FUNDS FOR THE DETAILED STUDY OF THE PPP PROJECT 51

3.3.4 PROCUREMENT OF THE TRANSACTION ADVISOR 51

4 PHASE II: PPP PROJECT APPRAISAL AND PREPARATION (FEASIBILITY STUDY) 55

4.1 FEASIBILITY STUDY AND OUTCOMES 58

4.1.1 PROJECT HISTORY BACKGROUND 58
4.1.2 DEVELOPING THE SERVICE OUTCOME LEVELS FOR THE PPP PROJECT 59
4.1.3 DESIGNING THE STAKEHOLDERS CONSULTATION PLAN 60
4.1.4 PROJECT AFFORDABILITY ANALYSIS 62
4.1.5 PROJECT DEMAND ANALYSIS 68
4.1.6 PROJECT TECHNICAL FEASIBILITY ANALYSIS 71
4.1.7 PROJECT FINANCIAL FEASIBILITY ANALYSIS 73
4.1.8 PROJECT ECONOMIC FEASIBILITY ANALYSIS 79
4.1.9 LEGAL AND INSTITUTIONAL FEASIBILITY ANALYSIS 81
4.1.10 PROJECT ENVIRONMENTAL AND SOCIAL IMPACT ASSESSMENT 84
4.1.11 PROJECT RISK IDENTIFICATION 87
4.1.12 PROJECT RISK ANALYSIS AND MITIGATION 91
4.1.13 RECOMMENDED PPP RISK ALLOCATION STRUCTURE 94
4.1.14 VALUE FOR MONEY ANALYSES/BENEFITS 99
4.1.15 MARKET TESTING 103
4.1.16 PPP PROCUREMENT STRATEGY 104
4.2 CONSOLIDATING THE PPP FEASIBILITY PROCEDURES 107
4.3 DISCLOSURE OF INFORMATION AT PROJECT STRUCTURING PHASE 109
5 PHASE III: STRUCTURING AND DRAFTING THE TENDER AND CONTRACT DOCUMENTS. 111
5.1 PROJECT STRUCTURING 114
5.1.1 PROJECT SPECIFICATIONS, COSTING AND FINANCIAL STRUCTURE 114
5.1.2 RISK ALLOCATION AND STRUCTURING 116
5.1.3 PAYMENT MECHANISMS AND INSTRUMENTS 116
5.2 SELECTION AND APPROVAL OF TYPE OF PROCUREMENT PROCESS 116
5.2.1 THE PPP AGREEMENT 118
5.3 MANAGEMENT OF TENDER DOCUMENT PREPARATION 118
5.3.1 PREPARATION OF EARLY MARKET ENGAGEMENT DOCUMENTS (OPTIONAL) ...119
5.3.2 PREPARATION OF THE REQUEST FOR QUALIFICATION (RFQ) 121
5.3.3 PREPARATION OF THE RFQ EVALUATION CRITERIA, GUIDELINES AND SCORING SHEET 122
5.3.4 PREPARATION OF THE REQUEST FOR PROPOSAL 124
5.3.5 BID BOND REQUIREMENT 130
5.3.6 CONTRACT GUIDANCE 132
5.3.7 SIGNIFICANT STANDARD AND SPECIFIC CLAUSES 135
5.3.8 AGREEING ON LANGUAGE FOR GENERAL CLAUSES 135
5.3.9 PPP SECURITY PACKAGES 135

5.3.10 DRAFTING THE PPP AGREEMENT 137

5.4 BOARD APPROVAL OF FEASIBILITY REPORT AND TENDER DOCUMENTS..................138

5.4.1 GUIDANCE ON SUBMISSION AND REVIEW OF APPRAISAL DOCUMENTS 138

5.4.2 GUIDANCE ON PPP DG OPINION 139

5.4.3 GUIDANCE ON BOARD DISCUSSION AND APPROVAL PROCESS..........................140

6 PHASE IV: TENDER & AWARD (IMPLEMENTING PPP TENDER) 142

6.1 PRE-TENDERING PREPARATIONS AND RECONFIRMATION OF PROCUREMENT PLAN & TENDER RESPONSIBILITIES 145

6.1.1 FINALIZE TENDER DOCUMENTS TO INCORPORATE COMMENTS OF PPP BOARD 145

6.1.2 CONFIRM SCOPE OF WORK FOR PPP TRANSACTION ADVISORS.........................145

6.1.3 PREPARE AND IMPLEMENT ACTION PLAN 146

6.2 ESTABLISHMENT OF PROJECT EVALUATION TEAM/PROCEDURES............................147

6.2.1 ROLE AND COMPOSITION OF THE PROJECT EVALUATION TEAM 147

6.2.2 SUPPORT FROM TRANSACTION ADVISOR IN ESTABLISHING THE PROJECT EVALUATION TEAM...148

6.3 LAUNCH OF TENDER PROCESS 149

6.3.1 PUBLICIZE THE GENERAL PROCUREMENT NOTICE AND THE EXPRESSION OF INTEREST (AS RELEVANT)...150

6.4 MANAGEMENT OF PREQUALIFICATION PROCESS...151

6.4.1 ISSUING THE RFQ AND RECEIVING PREQUALIFICATION SUBMISSIONS.............151

6.4.2 EVALUATING THE SUBMITTED RESPONSES FOR THE RFQ 152

6.5 MANAGEMENT OF REQUEST FOR PROPOSAL PROCESS...155

6.5.1 SETTING UP THE PHYSICAL/VIRTUAL DATA ROOM 156

6.5.2 ISSUE BID DOCUMENTATION TO APPROVED SHORTLISTED ENTITIES................157

6.5.3 MANAGE REQUESTS FOR CLARIFICATION 157

6.5.4 ARRANGE SITE VISIT(S) 158

6.5.5 HOST A BIDDERS' CONFERENCE 158

6.5.6 MANAGE ANY REVISIONS TO THE BID DOCUMENTS WITH PPPDG/CONTRACTING AUTHORITY...159

6.5.7 RECEIPT AND REGISTRATION OF SUBMISSIONS...159

6.5.8 BOND COMPLIANCE 160

6.5.9 PROPOSAL EVALUATION AND APPROVAL...160

6.5.10 HANDLING OF COMPLAINTS...166

6.6 ACTIONS ON APPROVAL OF EVALUATION REPORT BY THE PPP BOARD....................168

6.6.1 INITIAL NOTIFICATION OF PREFERRED BIDDER...168

6.6.2 RE-EVALUATING THE GOVERNMENT'S FISCAL COMMITMENT 169

6.6.3 BOARD APPROVAL FOR PROJECT AWARD...170

6.6.4 PROJECT AWARD 171
6.6.5 ESTABLISHMENT OF PROJECT COMPANY AND PPP AGREEMENT SIGNING 172
6.7 OTHER PRIVATE PARTY SELECTION METHODS 175
6.7.1 TWO STAGE BIDDING 177
6.7.2 COMPETITIVE DIALOGUE 178
6.7.3 DIRECT NEGOTIATIONS 180
6.8 FINANCIAL CLOSE 182
6.8.1 PROCESS OVERVIEW 182
6.8.2 ACCOUNTING/BUDGETING 182
6.8.3 ROLE OF LENDERS, POTENTIAL NEED FOR DIRECT AGREEMENTS 184
6.8.4 FOREIGN EXCHANGE 185
6.8.5 MANAGEMENT OF GOVERNMENT CONDITION PRECEDENTS 187
6.8.6 MONITORING OF PRIVATE PARTY CONDITION PRECEDENTS 187
6.9 NEXT STEPS 188
6.9.1 STRENGTHEN THE CONTRACTING AUTHORITY'S PPP PROJECT MANAGEMENT TEAM 188
6.9.2 CLOSE-OUT REPORT OR CASE STUDY 190
6.10 DISCLOSURE OF INFORMATION AT PROJECT TENDER AND AWARD PHASE 190
7 PHASE V: PPP CONTRACT MANAGEMENT & PERFORMANCE REPORTING 192
7.1 OVERVIEW OF CONTRACT MANAGEMENT 195
7.1.1 PROJECT MANAGEMENT TEAM 195
7.1.2 CONTRACT ADMINISTRATION PROCESS 196
7.1.3 RELATIONSHIP MANAGEMENT 197
7.1.4 PPP PROJECT PERFORMANCE MEASUREMENT AND MONITORING 198
7.2 PPP CONTRACT MANAGEMENT 200
7.2.1 PROJECT MANAGEMENT CHECKLIST 200
7.3 PERIODIC INTERNAL REVIEWS 202
7.4 DISCLOSURE OF INFORMATION AT CONTRACT MANAGEMENT PHASE 203
7.5 GUIDANCE ON MANAGING KEY ISSUES 204
7.5.1 REPORTING AND PERFORMANCE MONITORING 204
7.5.2 ANTICIPATED CHANGES AND TRANSITIONS 207
7.5.3 UNANTICIPATED CHANGES AND RENEGOTIATION 208
7.5.4 DISPUTES AND RELATIONSHIP MANAGEMENT 212
7.5.5 CONTRACT TERMINATION 214
7.5.6 END OF PPP CONTRACT 216

List of Figures

Figure 1-1: PPP Contracting Eco-System 8
Figure 1-2: Forms of PPPs 17
Figure 1-3: Forms of Public Private Partnership Schemes 18
Figure 1-4: Examples of PPP Contract Types 19
Figure 1-5: Contractual forms of PPP and Risk Levels 22
Figure 2-1: Parties Involved in PPP Process 27
Figure 3-1: Overview of Tasks under Phase I 33
Figure 3-2: Phase I Process and Key Stakeholders 34
Figure 3-3: Phase I Process and Key Stakeholders – Unsolicited Proposals 35
Figure 3-4: PPP Suitability 40
Figure 3-5: Separate Advisor vs. Single Transaction Advisor 53
Figure 4-1: Overview of Tasks under Phase II 56
Figure 4-2: Phase II Process and Key Stakeholders 57
Figure 4-3: Example of content of the PPP Project Affordability Report (detailed by year of the PPP Project) 68
Figure 4-4: Range of options of Government Support 71
Figure 4-5: Indicative steps involved in designing a PPP Financial Feasibility model 74
Figure 4-6: Value for Money in PPP projects 100
Figure 5-1: Overview of Tasks under Phase III 112
Figure 5-2: Phase III Process and Key Stakeholders 113
Figure 5-3: Selection and Approval of Type of Procurement Process 117
Figure 5-4: Procurement Stages and Documentation 118
Figure 5-5: Bid process – Bid Bond Management 131
Figure 5-6: Basic Scheme of a PPP Contract Structure 137
Figure 6-1: Overview of Tasks under Phase IV 143
Figure 6-2: Phase IV Process and Key Stakeholders 144
Figure 6-3: Importance of the Project Company in the PPP Process 174
Figure 6-4:Closing the PPP Transaction 188
Figure 7-1: Overview of Tasks under Phase V 193
Figure 7-2: Phase V Process and Key Stakeholders 194

List of Tables

Table 1-1: Comparison of Traditional Procurement and PPP 9
Table 1-2: Project characteristics creating possible conditions for PPPs..................................11
Table 1-3: Typical Risks in Road PPP projects...14
Table 2-1: Core Relevant Function and Composition..28
Table 2-2: Key Responsibilities throughout the PPP Project Development Lifecycle as per the Regulatory Framework in Ethiopia 30
Table 3-1: Summary of PPP Projects Prioritization Matrix...43
Table 4-1: Procedures for Developing Feasibility Study..58
Table 4-2: PPP Concerns and Interests by Stakeholder Group 61
Table 4-3: PPP Contract Management & Regulatory Issues 83
Table 4-4: PPP Risk Identification Matrix 98
Table 4-5: Key Questions on Procurement Strategy 105
Table 5-1: Prequalification Minimum Criteria 123
Table 5-2: PPP Agreement Heads of Terms...134
Table 5-3: Indicative List of Agreements 136
Table 6-1: Actions to take by the Contracting Authority and the PPPDG to facilitate the process of projects financing agreements negotiation 184
Table 7-1: Main Reasons for Renegotiation 210
Table 7-2: Assessment of the Need for renegotiation 211

INTRODUCTION

PURPOSE OF THE PPP GUIDELINES

In 2017, the Government of Ethiopia commenced the development a Public Private Partnership (PPP) framework in Ethiopia, in order to create an enabling environment for PPPs at the federal level.

The overarching objectives of the PPP framework are to facilitate the implementation of privately-financed infrastructure projects which promote Ethiopia's economic growth; to establish a fair and transparent legislative framework to guide this process; and to openly publish the specific operational and strategic procedures for the procurement and award of infrastructure projects involving PPPs.

The Policy for the Use and Implementation of PPPs (the "PPP Policy"), authored by the Ministry of Finance (MOF), provided the first mechanisms to develop and implement PPPs in the country in 2017. The policy was followed by a proclamation ratified by the Federal House of Representatives in January 2018 and a specific PPP directive issued by the Ministry of Finance in July 2019. To date, the actions undertaken by the Government of Ethiopia towards developing this architecture of PPPs include the following:

i. **The adoption of a Policy for the Use and Implementation of PPPs (the "PPP Policy").** *The PPP Policy gives an overall direction to the way PPP projects are implemented in the Ethiopia.*

ii. **The ratification by Parliament of a *Public Private Partnership Proclamation (No. 1076/2018) (the "PPP Proclamation").*** *The objective of the PPP Proclamation is (i) to establish a favourable framework facilitating privately financed infrastructure projects and (ii) to develop the general principles of transparency, efficiency and fairness in the award of contracts by public entities. The PPP Proclamation defines (i) what PPPs are, (ii) the role of the different stakeholder involved, (iii) the procedures to follow at each phase of a PPP process.*

iii. **The development of a Directive issued to implement Public Private Partnership (No. 55/2010/2018) ("the "PPP Directive").** *The Directive is a secondary legislation issued to further detail how PPPs will be initiated, developed and implemented by the PPPDG, Ministry or Contracting Authorities.*

iv. **The establishment of a PPP Directorate General (PPPDG) within the Ministry of Finance (MOF) in 2018.** *The PPP Proclamation establishes the PPPDG and details its objectives, duties and responsibilities throughout the PPP project cycle.*

Figure 1-: PPP Framework Chronology

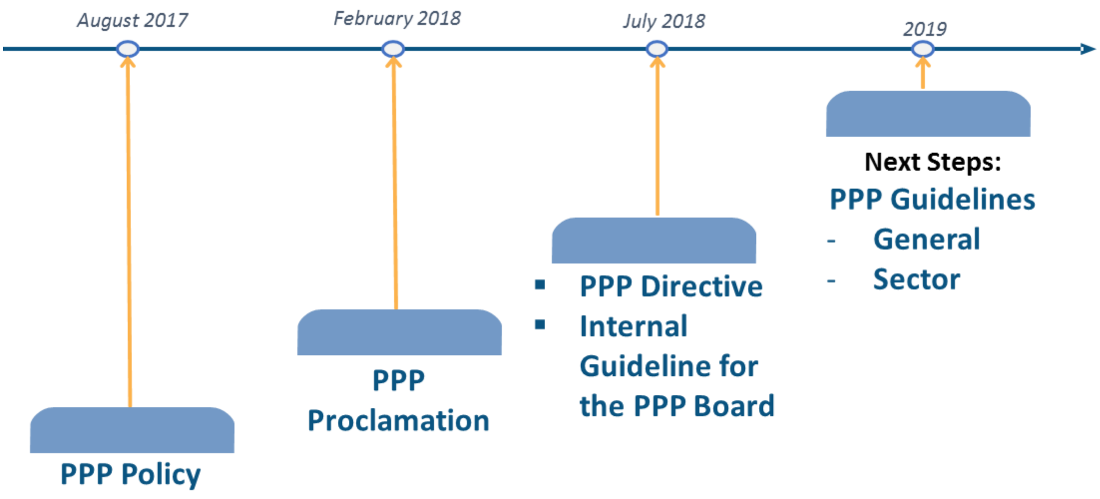

The PPP Guidelines represent the next step in the development of the PPP Framework. Article 12 (4) of the Proclamation and article 8 of the PPP Directive[1] required the PPPDG to issue guidelines designed to foster the development of PPPs.

The General PPP Guidelines herein take users through the PPP lifecycle for all federal-level PPP projects – from project identification and selection, to project development, procurement and contract management – with the sector-specific guidelines for the six priority sectors - energy, transport, water, health, industrial parks and public housing - provided separately under **Appendix F** (together referred to as, "the PPP Guidelines").

The purpose of these PPP Guidelines is to:

- Provide consistency and clarity to all parties involved in PPP projects, both private and public sectors alike regarding how a PPP project will be identified, assessed, tendered and managed to meet the Federal government's objectives for PPPs[2];
- Provide detailed guidance to line ministries, departments and agencies during the development of PPP projects over the full PPP project development life cycle;
- Serve as notice to the private sector of the governments' intent to systematically develop PPP projects; to ensure quality and consistency across projects; and to competitively procure private sector parties – thereby instilling confidence in prospective partners;

[1]PPP Directive No. 55/2010/2018 Article 8: 1/ The PPP Directorate General shall issue guidelines designed to foster the development of Public Private Partnerships covering topics deemed appropriate by the PPP Directorate General including at least: a. Identification and selection of potential PPP projects; b. Project Feasibility screening and appraisal manual c. Value for Money; d. Requirements for the request and use of Government Support; e. Model Project Agreements; f. PPP contract management practices;

[2]PPP Proclamation Article 3: 3. Objective [This Public Private Partnership Scheme shall have the following objectives: 1/ to create a favorable framework for promoting and facilitating the implementation of privately financed projects to support Ethiopian economic growth; 2/ to enhance transparency, fairness, Value for Money, efficiency and long-term sustainability; 3/ to improve quality of Public Service Activity; and 4/ to maintain macroeconomic stability by reducing growth in public debt.]

- Identify the key parties involved in the development of PPP projects at each stage; and
- Allow for regular updating and improvement of PPP procedures and practices, with the objective of ensuring consistency and synergy with the broad regulatory framework.

ORGANIZATION OF THE GUIDELINES

The PPP Guidelines are organized based on the PPP lifecycle. PPPs have a lifecycle from project identification to handback[3]; it is helpful to break up this process into phases to help focus on the distinct lifecycle elements, and in order to facilitate effective management and delivery. These phases are as follows:

i. PPP Project identification & screening (selection & review)

ii. PPP Appraisal & preparation (feasibility study)

iii. Structuring, draft contract & tender documents

iv. Tender and award (selection and contracting of the private partner)

v. PPP Contract management and performance review

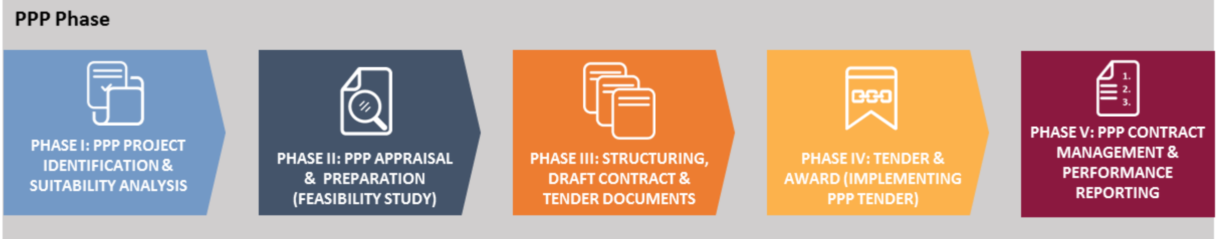

The Sector Guidelines (**Appendix F – Priority Sector Guidance**) provide additional guidance and information to users on the six specific sectors. They are designed to be used alongside the General Guidelines, highlighting particular characteristics and issues relating to the particular sector that will need to be taken into account in designing, delivering and successful monitoring performance on PPP projects. This can take the form of particular PPP models, structures, payment mechanisms, etc. and pointers from international experience and best practice.

It is important to provide context regarding PPPs for the users of the guidelines with respect to the key concepts behind PPPs together with a description of the Institutional Framework for delivering PPPs in Ethiopia.

The PPP Guidelines therefore include two introductory chapters which present (i) key concepts and (ii) the institutional framework, with the remaining Chapters covering the five stages of PPP development as follows:

**Chapter 1**	Key Concepts: PPP Primer and Phases in PPP Development
**Chapter 2**	Institutional Framework: Roles and Responsibilities

[3] This is at the end of the PPP Term where the Private Party hands back the rights to manage the Public Service Activity on behalf of the Contracting Authority.

Chapter 3 ▪ Phase I: PPP Project Identification and Screening (Selection and Review)

Chapter 4 ▪ Phase II: PPP Project Appraisal and Preparation (Feasibility Study)

Chapter 5 ▪ Phase III: Structuring and Drafting the Tender and Contract Documents

Chapter 6 ▪ Phase IV: Tender & Award (Implementing PPP Tender)

Chapter 7 ▪ Phase V: PPP Contract Management & Performance Reporting

Appendix A ▪ **PPP Glossary and Key Concepts**

Appendix B ▪ **Background references, Forms and Templates for Phase 1 PPP Project Identification & Suitability Analysis**

Appendix C ▪ **Background references, Forms and Templates for Phase 2 PPP Appraisal and Preparation**

Appendix D ▪ **Background references, Forms and Templates for Phase 3 Structuring, Draft Contract &Tender**

Appendix E ▪ **Background references, Forms and Templates for Phase 4 Tender and Award**

Appendix F ▪ **Priority Sector Guidance:**

- *Part 1. Energy Sector Guidelines*
- *Part 2. Transport Sector Guidelines*
- *Part 3. Water Sector Guidelines*
- *Part 4. Industrial Parks Sector Guidelines*
- *Part 5. Public Housing Sector Guidelines*
- *Part 6. Health Sector Guidelines*

1 PPP PRIMER AND PHASES IN PPP DEVELOPMENT

1.1 DEFINITION OF PPPs

1.1.1 WHAT IS A PPP?

Physical infrastructure, such as roads, water and sanitation networks, and transportation systems, involve large investments that are traditionally provided by the public sector. The demand for infrastructure can put severe strain on the government's capacity for both finance and funding; this pressure is especially great for economies undergoing rapid development and urbanisation, and for countries with a significant need for expanded infrastructure. Ethiopia notably has a substantial infrastructure need and an associated infrastructure funding gap.

Public-private partnerships (PPPs) are increasingly being used by governments and public sector authorities throughout the world as a way of increasing access to infrastructure services for their citizens and economies at a reduced cost.

Through the involvement of the private sector, the amounts invested in infrastructure can increase as use is made of new sources of finance (private sector financing) achieving a larger portfolio of projects than would be undertaken by the government alone. The government is also able to transfer risks to the private sector (e.g. benefits from on budget and on time performance) and gain overall improvements in efficiency through the benefits of private sector management, experience and innovation.

PPP projects often involve the private sector arranging and providing finance. This frees the public sector in the short-term from the need to meet financing requirements from its own revenues (taxes) or through borrowing. This is an advantage where the public sector is facing limits on how much capital it can raise in the short term, as in Ethiopia. By deferring payments, PPPs can enable more pressing investment in infrastructure and increased access to infrastructure services.

A PPP typically has the following characteristics:

- The private sector is responsible for carrying out or operating the project and takes on a substantial portion of the associated project risks;
- During the operational life of the project, the public sector's role is to monitor the performance of the private partner and enforce the terms of the contract;
- The private sector's costs may be recovered in whole or in part from charges related to the use of the services provided by the project, and may be recovered through payments from the public sector;
- Public sector payments are based on performance standards set out in the contract;

- Often the private sector will finance upfront the majority of the project's capital costs, although this is not always the case.

It will often be necessary to build, rehabilitate, or add to existing assets in order to meet the infrastructure needs of the economy and users. However, an important part of the infrastructure PPP concept is that a PPP is focused on outputs, and the outputs of the PPP are infrastructure services, not infrastructure assets.

The reason for the focus on outputs and services rather than assets is to encourage efficient use of public resources and improved infrastructure quality. The private partner is not constrained by input specifications and can design the project to achieve for example the best whole-of-life costs benefits.

A PPP brings the public and private sectors together as partners in a contractual agreement, for a pre-defined period (e.g. 20-30 years) matched to the life of the infrastructure assets used to provide the services. The private partners (investors, contractors and operators) provide specified infrastructure services and, in return, the public sector either pays for those services or grants the private partner the right to generate revenue from the project. For example, the private partner may be allowed to charge user fees or receive revenue from other aspects of the project.

1.1.2 DEFINITIONS

In Ethiopia, the PPP Proclamation[4], defines a Public Private Partnership (PPP) as "*a long-term agreement between a **Contracting Authority** and a **Private Party** under which a Private Party:*

a) undertakes to perform a Public Service Activity that would otherwise be carried out by the Contracting Authority;

b) receives a benefit by way of:
(1) compensation by or on behalf of the Contracting Authority;
(2) tariffs or fees collected by the Private Party from users or consumers of a service;
(3) a combination of such compensation and such charges or fees; or

c) is generally liable for risks arising from the performance of the activity or use of the state property in accordance with the terms of the Project Agreements."

A **Public Service Activity**, as per the PPP Proclamation[5], means "*any activity the government has decided to perform for the reason that it has deemed it to be necessary in the general interest of the public and considered that private initiative was inadequate for carrying it out*." These services are provided by the Government in the public interest, unless otherwise decided.

The Private Party to enter into a Public Private Partnership with the Contracting Authority is required under the legal framework to establish a "***Project Company***"[6], incorporated under the

[4] Public Private Partnership Proclamation (No. 1076/2018) Article 2(12)
[5] Public Private Partnership Proclamation (No. 1076/2018) Article 2(14)
[6] Public Private Partnership Proclamation (No. 1076/2018) Ch. 10 Article 44

laws of the Federal Democratic Republic of Ethiopia, in order to execute and implement the obligations in the PPP Agreement – and as applicable, any other Project Agreements.

A **Contracting Authority**, as per the PPP Proclamation[7], means *"a Public Body or Public Enterprise which intends to enter into a Public Private Partnership Agreement with a Private Party."* The Public Entity means either a Public Body (*"any organ of the Federal Government which is wholly financed by the Federal Government budget")* or a Public Enterprise *("an enterprise fully owned by the Federal Government and defined under the relevant laws of the Federal Democratic Republic of Ethiopia"*). For example, under this definition, the Ministry of Transport is a Public Body whereas the Ethiopian Railways Corporation is a Public Enterprise. A Public Entity takes on the characterization of the Contracting Authority upon the decision to procure a project as a candidate PPP, and the "Public Entity" is confirmed by the PPP Board (if necessary[8]) as the counterparty to sign the PPP Agreement with the Private Party.

The **Project Company** to be established by the Private Party is a special purpose vehicle established for the purpose of entering the PPP Agreement.

Aside from the PPP Agreement, the Project Company may also enter into several other ancillary agreements. A typical contractual eco-system between a Contracting Authority and the Private Party Project Company is illustrated below.

Private parties in PPPs are generally compensated via two forms, or a hybrid of the two:

- **User pay PPP,** whereby the private partner is mostly or completely paid by the users;
- **Public payment PPP,** whereby it is the public entity (i.e., the Contracting Authority) that ensures the payment of the private partner's compensation and therefore bears the revenue risks associated with the operation of the infrastructure asset and the related services; or
- **Hybrid**, a combination of the two sources of funding.

Careful and appropria*te* **risk allocation** between the public and private partners is also critical focus of PPP design. If private partners do not bear the risks that are under their control, their incentives for efficiency will be weakened and PPP benefits may be reduced. Discussion on risk sharing modalities can be found in sections 1.2 and 1.3.3.

---

[7] Public Private Partnership Proclamation (No. 1076/2018) Article 2(2)

[8] Article 6(1) of the PPP Proclamation states that the Contracting Authority for a PPP shall be the Public Entity legally mandated to be responsible for the infrastructure. Article 6(2) states, in the event that a PPP project involves infrastructure/services the responsibility of more than one Public Entity, the PPP Board shall select the appropriate Contracting Authority.

Figure 1-1: PPP Contracting Eco-System

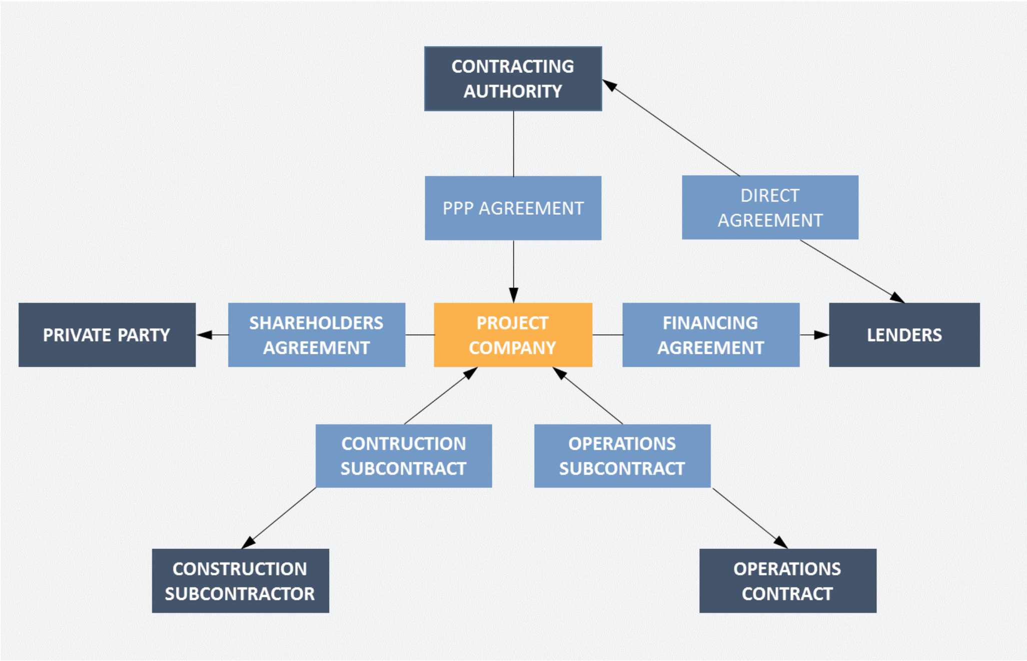

In some PPPs, the Contracting Authority may also be a shareholder in the Project Company. This is often the case where the Government, represented by the Public Entity that is Contracting Authority, has committed to investments in the project or makes some other intangible contribution to project equity. In such instances, the shareholders in the Project Company would include the Contracting Authority.

1.1.3 WHY USE PPPS

The advantages of PPP include:

- Access to private sector finance
- Efficiency advantages from using private sector skills and from transferring risk to the private sector
- Potentially increased transparency
- Enlargement of focus from only creating an asset to delivery of a service, including maintenance of the infrastructure asset during its operating lifetime (Focus on outputs)
- Risks are allocated to the party best able to manage the risk
- This broadened focus creates incentives to reduce the full life-cycle costs (i.e., construction costs and operating costs). Better value for money over the lifetime of the project. Value for Money means that the undertaking of the Public Service Activity by a Private Party under a PPP results in a net benefit accruing to that Contracting Authority or consumer defined in terms of cost, price, quality, quantity, timeliness of implementation and other factors which influence the determination of the best economic value compared to other options of delivering this Public Service Activity or use of government property.

All of these provide strong reasons in favour of using PPPs for infrastructure delivery. However, PPPs should only be used when it is the most appropriate procurement option (See section1.1.4).

Some risks are better managed by the private sector. The transfer of those risks and responsibilities to the private party is expected to yield Value for Money (VfM). The PPP Proclamation[9] states, "the incorporation of private sector participation is contributing to a net benefit for the Contracting Authority or the consumer, in terms of cost, price, quality, quantity, timeliness of implementation and other factors, compared to public service delivery."

VfM tends to be driven by:

- Transferring risks to the party best able to manage the risks;
- The long term nature of contracts;
- Competition;
- Output-based specifications;
- Performance measurement and incentives; and
- Private sector management skills.

Further detail on 'Value for Money" and how it is assessed is presented in Chapter 4.

The table below compares traditional public sector procurement and PPP.

Table 1-1: Comparison of Traditional Procurement and PPP

	TRADITIONAL PROCUREMENT	PPP
**RISK**	Public sector bears the majority of risk of undertaking project	Risks distributed between public sector and private sector
**PROJECT MANAGEMENT**	Public sector is responsible for all project management and interfaces	Private sector managed overall project and interfaces
**PERFORMANCE MANAGEMENT**	Private sector may undertake elements of the design, construction, operation and management or combine them on turnkey basis. Public sector is accountable for performance and maintenance.	Private sector assumes responsibility for design, construction, operation & Maintenance and even management and is paid based on performance.
**FUNDING**	All project related costs (capital and operational) paid by public sector collected through taxes or user fees	Users pay for services or public sector agency pays for service on predefined performance criteria
**FINANCING**	Public sector responsible for securing finance	Depending on the PPP model, the private sector may be responsible for financing through arrangement of equity and debt

Source: CPCS

[9] Article 2(18) of the PPP Proclamation

1.1.4 WHEN SHOULD A PPP BE USED?

PPPs are different to the traditional public sector route and these differences require adaptation of approach and capabilities in the public sector. There are also some new costs associated with PPPs.

A PPP is not a panacea for all the public sector's financing and infrastructure problems and PPPs are not always the most appropriate procurement option. PPPs can be complex as the procurement process can be lengthy and involve high transaction costs. Access to private sector finance tends to involve borrowing from banks and other investors on a 'project finance' basis where monies are secured on the project itself not the parties involved. Project due diligence is an important part of the process and can involve considerable time and costs lengthening the procurement process. Concepts of bankability and affordability are important and discussed at length later in the guidelines. The projects tend to be of a long duration (20-30 years) and challenging to predict in the long term. This can make it difficult to enforce contracts over time leading often to renegotiations. Therefore, not all projects are suitable in terms of demonstrating value for money for the government.

The use of PPP for infrastructure projects should only be considered when:

- *The public sector environment is suited to supporting PPPs* – a PPP is a complex arrangement that requires support from the public sector during development and operation. The likelihood of PPP success will be increased when the public sector supporting environment is strong.
- *The project is suitable to being carried out as a PPP* – certain characteristics make a project well suited to being a PPP, while others imply that the PPP approach will be difficult or inappropriate.
- *The potential barriers to successful project implementation have been identified and can be overcome* – many of the common obstacles to successful PPP implementation can be identified in advance. If these are insurmountable then the project should not proceed as a PPP. If they can be overcome, as will often be the case, then this needs to be factored into the PPP development and thoroughly planned.
- *Given that these conditions are satisfied, the project must be commercially viable for the private sector and offer value for money (VFM) for the public sector* —the choice of PPP should allow the project to be undertaken at lower cost on a lifetime basis, while delivering the same or better quality services than could be achieved through implementation by the public sector or private sector on their own. It must also be commercially viable in order to be attractive to private investors.

These important conditions should be checked early for every project. This will improve the quality and likely success of projects entering the PPP development pipeline (Chapters 3 & 4).

Table 1-2: Project characteristics creating possible conditions for PPPs

Characteristic	Description
**Opportunity for risk transfer**	– A defining characteristic of a PPP arrangement is the ability to allocate project responsibility and risk to the party best positioned to manage it. Achieving value for money on a PPP project requires a well-managed scope to allocate appropriate risks to the private sector with the aim of optimizing risk allocation in the partnership.
**Whole-of-life cost certainty**	– Improved efficiencies can be realized where a project can allow for the full integration of planning, design, construction and ongoing service delivery (including operational, lifecycle maintenance and refurbishment) to be the responsibility of a single party.
**Long term**	– PPP arrangements tend to be more suitable for projects where Government is willing to contract to the private sector for the long term (generally between 20 and 50 years). This enables the benefits of efficient risk transfer and a whole life of cost approach to be delivered.
**Increased scope for core services**	– Traditionally, PPP models often include a requirement for a range of non-core and support activities to be delivered that were otherwise utilizing public sector capabilities and resources. Increasingly, Governments have also begun incorporating core service requirements in PPP contracts. This has been well received and responded to by the market.
**Clear and measurable outputs**	– Projects where the output requirements are clearly understood by the Government and can be readily measured are likely to be viable under a PPP model as these measurable outputs can be translated into performance contracts. This also allows for payment mechanisms to be structured on the basis of these outputs and the alignment of financial incentives to key performance indicators.
**Innovation**	– Larger projects tend to be sufficiently complex and often require more innovative approaches to achieve value for money (in terms of asset and service design, technology and/or delivery).
**Market Appetite**	– The project needs to create certainty and a genuine commercial opportunity to attract an appropriate number of private sector parties and allow for effective and competitive tender process.
**Additional commercial opportunities**	– The private sector may favor additional commercial opportunities (e.g. retail) as a result of the project that could allow them to realize revenue streams earlier in the project. For example, certain sectors like airports can offer additional commercial opportunities like duty-free stores, where the private party can sell additional services and goods to end users. However, additional commercial development, which may be non-core, can add to the complexity of the PPP arrangement.
**Bundling of contracts**	– A PPP arrangement can be an effective delivery model where the Government is required to provide a service or capability that depends on a number of separate contracts. PPPs can combine related services and an asset into a single long-term contract. This can add to complexity, particularly as an increased number of client stakeholders may be involved.

Source: Guidelines for Public Private Partnerships. The Partnership Framework. Australian Capital Territory, Canberra. Second Edition: May 2016 https://apps.treasury.act.gov.au/__data/assets/pdf_file/0010/869941/Guidelines-for-PPPs-Second-Edition.pdf

1.1.5 PPPS IN ETHIOPIA

The PPP framework in Ethiopia is prepared in such a way to be both broad enough to capture all types of PPPs and flexible enough to address different issues that may arise. This framework includes a threshold for PPPs to be considered within the Federal process and excludes a number of sectors from the framework as follows:

1.1.5.1 Threshold for PPPs in Ethiopia

The PPP Policy adopted in August 2017, anticipates that given the cost of PPP project development, projects with contract values of more than USD50 million are generally those that would generate Value for Money. However, the policy[10] does not rule out the possibility that projects with a contract value lower than USD50 million can generate Value for Money and thus be eligible for procurement as PPPs. Each candidate PPP project would need to be assessed on merit.

1.1.5.2 Sectors where PPPs are not permissible

Article 4 of the PPP Proclamation indicates that the activities in the four sectors below as well as divestiture transaction are excluded from its scope of the PPP Proclamation:

- Oil;
- Mines;
- Minerals;
- Rights of air space[11]; and
- Privatization or **divestiture** of public infrastructure or public enterprises

Privatization/Divestiture are not considered PPP under the Ethiopian PPP framework. Privatizations presents a model whereby the government makes a permanent transfer to the private sector of a previously publicly-owned asset and the responsibility for delivering a service to the end user. Usually, the role of the private sector remains limited to regulate the delivery of the services. A PPP however necessarily involves a **continuing role for the public sector as a "partner" in an ongoing relationship with the private sector**[12].

1.2 RISK: A CRITICAL FOCUS OF PPP DESIGN

1.2.1 MAJOR RISKS IN INFRASTRUCTURE PPPS

The requirements for effective risk transfer and the ability to harness private sector efficiencies means PPPs are best suited to projects for which:

---

[10] The USD50 million requirement is introduced in the PPP Policy and not the PPP Proclamation which has a binding effect. It can therefore be adjusted going forward as needed by the Government.

[11] This does not include air right, which is the right to control, occupy, or use the vertical space above a property, subject to the regulation in place.

[12] World Bank -Farquharson, Torres de Mästle, and Yescombe, with Encinas 20112011

- It is possible to clearly specify the requirements in terms of service outputs – the idea is to capture as much of the private sector efficiencies as possible by allowing scope for bidders to introduce efficiencies through innovations proposed in their bids
- The requirements can be specified so as to enable monitoring of performance against measurable standards and enforcement of penalties where standards are not met
- The requirements of the public sector sponsor are likely to be stable throughout the life of the PPP – the aim is to avoid the need to renegotiate the contract at a later date due to changes to project scope or requirements

"*Risk is the chance of an event occurring which would cause actual project circumstances to differ from those assumed when forecasting project benefits and costs"*[13] Allocating risk to achieve added efficiency is what makes PPP a potentially powerful way of reducing project-related costs and achieving improved Value for Money for the public sector. The level of risk can be changed by allocating responsibility for individual risks to those who are best able to manage them.

The public and private sectors are different in the types of influence and information that they have. This means that they can control risks in different ways from each other and they are better at controlling some risks and not as good at controlling others. For example, the public sector has certain powers and advantages in the process of land acquisition that mean it is sometimes better suited to this task and undertaking the associated risks. By contrast, the private sector is exposed to competitive pressures that force it to establish improved management practices. It is also often the technology leader. This means it may be better suited to managing the design and construction risks.

On their own, both the public and private sectors are weaker in their ability to control certain risks. One of the goals of a well-designed PPP is to pick out the strengths and combine them. The result should be that a partnership of public and private parties is stronger and more efficient than either party by itself.

Not all projects will have the same set of risks and the risks that are common will vary in importance from one project to another. However, it is possible to identify a set of risks that generally apply to projects in the sector.

For example, the typical risks for a project in the roads sector are shown and described in the table below, grouped according to important project stages and 'other' risks. Further discussion of risk can be found in Chapters 4 and 5.

---

[13] Risk Management & Contractual Issues, Partnerships Victoria Guidance Material June 2001 S3.1

Table 1-3: Typical Risks in Road PPP projects

RISK TYPE	DESCRIPTION
**Pre-operative task risks**	
Delays in land acquisition	– Refers to the risk that the project site will be unavailable or unable to be used within the required time, or in the manner or the cost anticipated or the site will generate unanticipated liabilities due to existing encumbrances and native claims being made on the site.
External linkages	– Refers to the risk that adequate and timely connectivity to the project site is not available, which may impact the commencement of construction and overall pace of development of the project.
Financing risks	– Refers to the risk that sufficient finance will not be available for the project at reasonable cost (e.g., because of changes in market conditions or credit availability) resulting in delays in the financial closure for a project.
Planning risks	– Refers to the risk that the pre-development studies (technical, legal, financial and others) conducted are inadequate or not robust enough resulting in possible deviations from the planned or expected outcomes in the PPP project development.
Approvals risk	– Refers to the risk that necessary permits, authorisations and approvals required prior to the start of construction are not obtained in a timely fashion, resulting in delays to construction and the project as a whole.
**Construction phase risks**	
Design and technological risk	– Refers to the risk that the technology used will be unexpectedly superseded during the term of the project and will not be able to satisfy the requirements in the output specifications. It would result in increased costs of a replacement technology.
Construction risk	– Refers to the risk that the construction of the assets required for the project will not be completed on time, budget or to specification. It may lead to additional raw materials and labour costs, increase in the cost of maintaining existing infrastructure or providing a temporary alternative solution due to a delay in the provision of the service.
Approvals risk	– Refers to the risk that delays in approvals to be obtained during the construction phase will result in a delay in the construction of the assets as per the construction schedule. Such delays in obtaining approvals may lead to cost overruns.
**Operation phase risks**	
Operations and maintenance risk	– Refers to the risks associated with the need for increased maintenance of the assets over the term of the project to meet performance requirements.
Volume risk	– Refers to the risk that demand for a service will vary from that initially projected, such that the total revenue derived from the project over the project term will vary from initial expectations. There is no risk in annuity contracts.
Payment risk	– Refers to the risk that tolls are not collected in full or are not set at a level that allows recovery of costs. This is a risk for the public sector under shadow tolls and for the private sector under user tolls. There is no risk in annuity contracts.
Financial risk	– Refers to the risk that the private sector over stresses a project by inappropriate financial structuring. It can result in additional funding costs for increased margins or unexpected refinancing costs.
**Handover risks**	
Handover risk	– Refers to the risk that the concessionaire will default in the handover of the asset at the end of the project term or will deviate from the minimum quality / value of the asset that needs to be handed back to the public entity.

RISK TYPE	DESCRIPTION
Terminal value risk	– Refers to the risk relating to differences from the expected realisable value of the underlying assets at the end of the project.
**Other risks**	
Change in law	– Refers to the risk that the current legal / regulatory regime will change, having a material adverse impact on the project.
Force Majeure	– Refers to the risk that events beyond the control of either entity may occur, resulting in a material adverse impact on either party's ability to perform its obligations under the PPP contract.
Sponsor risk	– Refers to the risk that sponsors will prove to be inappropriate or unsuitable for delivery of the project, for example due to failure of their company.
Concessionaire event of default	– Refers to the risk that the private entity will not fulfil its contractual obligations and that the government will be unable to either enforce those obligations against the sponsors, or recover some form of compensation or remedy from the sponsors for any loss sustained by it as a result of the breach or the sponsors will prove to be inappropriate or unsuitable for delivery of the project.
Government event of default	– Refers to the risk that the government will not fulfil its contractual obligations and that the private entity will be unable to either enforce those obligations against the government, or recover some form of compensation or remedy from the government for any loss sustained by it as a result of the breach.

Source: India PPP Toolkit – Road Sector https://www.pppinindia.gov.in/toolkit/highways/module1-racfopd-mriip.php?links=risk1a

1.3 OVERVIEW OF PPP MODEL VARIANTS

1.3.1 PROJECT CHARACTERISTICS THAT AFFECT THE CHOICE OF PPP MODEL

There are a number of different PPP modes and variants of them and they will be appropriate to different types of projects. This will depend in particular on the nature of the service or output required, which in turn depends on the sector and sub-sector, and the political and economic climate in which the PPP will be carried out.

The key aspects that define the PPP model are:

- Does the PPP involve building new assets to provide the service (capital expenditure project), or are the required services for operations and management only?
- Which roles will the private sector carry out? For example, who will provide finance? Who will design and construct?
- Who will have ownership of the assets during the PPP and when the PPP ends?
- What will be the duration of the contract?
- How are the various project risks allocated between the private and public partners?
- What will be the major revenue source for the project? For example, will it be from charges to users (e.g. ticket sales, direct tolls, user charges, etc.), or payment from Government (e.g. availability payment, annuity, subsidy, etc.)?
- Is demand for the infrastructure service expected to be stable over the period of the contract?

New (“Greenfield”) or existing assets – Greenfield developments, which include major capital expenditure to build new infrastructure, have different requirements to the rehabilitation or management of existing assets in Brownfield developments.

The scope of potential private sector roles is broader in Greenfield projects. The chosen PPP model will reflect whether the private sector will be responsible for the design, finance and construction of the project (e.g. DBO agreement or a variation) or only some of these roles.

Ownership flexibility – There may be legal restrictions or political sensitivities on public ownership.

Restrictions on ownership rule out PPP models that specifically contain ownership aspects, such as Build-own-operate (BOO) and its variants (e.g. BOOT). In this case other options such as lease management contracts, BOT, BTL, could be considered.

Lifetime of the asset and scale of capital costs – Infrastructure assets that involve large upfront capital costs, such as roads, require long timeframes for cost recovery. Such assets tend to be suited to more long-term contracts (e.g. BOT, BLT etc.).

However, long timeframes also bring greater risk of future unknowns. The public sector may be required to take on some of these risks by providing some guarantee to cost recovery in order to attract private sector project finance. For example, for a road project where future traffic volumes are uncertain the PPP might be structured with annuity payments rather than being toll-based, to reduce the revenue risk to the private operator. Alternatively, if long-tenor finance from the private sector is not available public sector financing may need to step into the gap.

The willingness or ability of the public sector partner to meet these risks is a further factor to be considered in determining the length of contract. For example, if facilities to support long-tenor debt are not available shorter term contracts with renewal clauses may be appropriate.

The nature of the service to be provided and the supporting infrastructure assets – More broadly, the nature of the end-user service itself will tend to favour a type of contracting structure. This is related to the capital cost structure (scale and timing) and the nature of the assets (physically fixed to their location or transportable).

Large capital-intensive network infrastructure assets tend to be natural monopolies and require some form of institutional price and quality regulation, either within the terms of contract or by a dedicated regulatory agency.

By contrast, some services such as those that are provided on the network (e.g. municipal buses, electric energy) or solid waste collection, can be subject to market competition. A different contracting structure is possible in this case, including greater opportunity for shorter contracts and periodic competitive re-bidding to maintain pressure on costs.

Cost recovery options – Whether the revenue from the PPP will be from a user-charge or a management fee or annuity paid by the public sector has important implications for the nature of the risk sharing.

Stability of demand for the services required – Long-term PPP contracts are best suited to the provision of infrastructure services which are not expected to change much through time. These

projects have lower risk of unforeseeable outcomes compared with projects whose services are subject to change, for example in sectors that are subject to rapid technological change.

In some cases, it may be necessary to provide the project with some protections from competition in order to reduce volume and revenue risk. For example, a roads project might have a guarantee from the public sector that an alternative route won't be allowed nearby within a set number of years or until traffic has reached a specified level.

1.3.2 FORMS OF PPP

The PPP proclamation[14] provides the following concerning the forms of Public Private Partnerships:

1. Public private partnerships may involve any of the following activities or any combination thereof:

   a) The design, construction, financing, maintenance or operation of new Infrastructure Facilities.

   b) The rehabilitation, modernization, financing, expansion, maintenance or operation of existing Infrastructure Facilities; and/or

   c) The administration, management, operation or maintenance pertaining to new or existing Infrastructure Facilities.

2. The Contracting Authority shall select the form of contract which reflects the desired allocation of risks and responsibilities for each agreement.

   Different PPP modes can be compared on a spectrum ranging between low and high levels of private participation and involvement.

Figure 1-2: Forms of PPPs

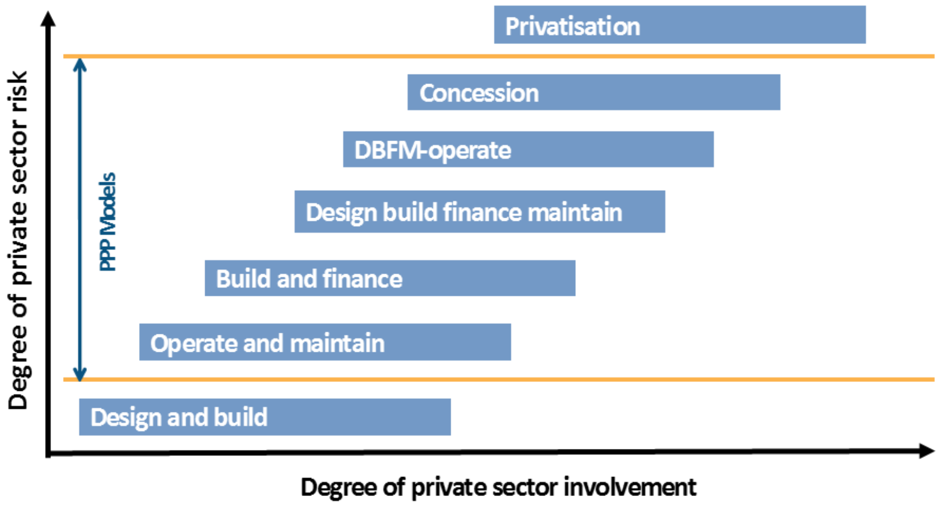

Source: Adapted from Canadian Council PPP, 2009

[14] Article 5 of the PPP Proclamation

As discussed in section 1.1.2, the different modes of compensation of the private partner is reflected in the definition of PPP under Article 2(12) of the PPP Proclamation. Private parties in PPPs are generally compensated via two forms, or a hybrid of the two: User pay PPP, Public payment PPP, and a Hybrid one which is a combination of the two sources of funding.

- .

PPP models specify the responsibility (and sequencing) for design, construction, ownership, operation and status of the project at the end of the contract period (whether it remains in private hands or is it transferred to the private sector). There are separate terms depending on whether the facility is built or rehabilitated and whether it is owned, concessioned or leased. The main difference between various types of PPP is the level of risk allocation, as illustrated below.

PPP projects involve a combination of the following components which can be attributed to the private party:

- Design
- Build (Construction)/Rehabilitation
- Finance
- Maintain
- Operate
- Transfer asset back to public authority

Figure 1-3: Forms of Public Private Partnership Schemes

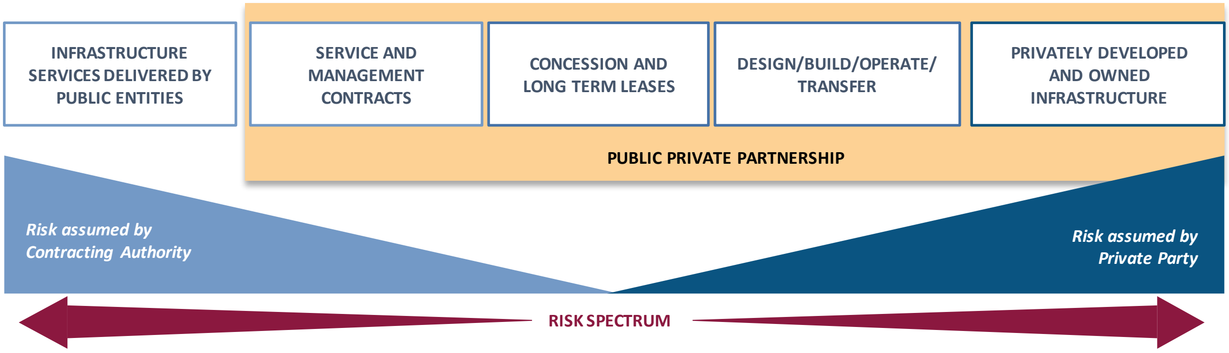

Source: CPCS

The above figure illustrates, in the top row, the possible forms of ownership and management of infrastructure facilities. In the bottom half of the figure, the relative share of risks assumed by the public and private sector is shown. At one extreme (at the left-hand side) is the traditional archetype, whereby infrastructure is owned by the public sector and operations are managed by a public enterprise. At the other end of the spectrum is the full private option, where both infrastructure and services are privately-developed and owned.

Under the traditional structure, the public enterprise manages asset delivery (usually through turnkey contracts) and revenue collection (with public enterprise personnel). When public enterprises start contracting out some functions, then the relationship starts moving towards the right side of **Figure 1-3** . For example, a public enterprise may decide to contract out revenue management to a private party with the objective of improving revenue collection and also training the public enterprise personnel. The contract could be structured such that the private party takes over the revenue collection platform infrastructure (e.g. locations and technology) of the contracting authority. Under the simplest form of such a contract, the private party will not take the risk of the contracting authority’s asset (i.e. not be responsible for renewing or

maintaining the infrastructure). The contract could also be structured so the private party manages contracting authority personnel to carry out the work, thus the private party need not introduce its own personnel. The private party could be paid a fixed fee or in some cases take a percentage of revenue collected, accepting a minimal level of payment risk as a performance measure. This contract form, has elements of a PPP, and most resembles a management contract, to the right of the traditional archetype. As the contract form assigns more and more responsibility (investment, management, etc.) and risk to the private sector on long term basis, the PPP forms moves rightwards in **Figure 1-3**.

In Ethiopia, the legal framework is explicit that privatizations or divestitures of public infrastructure or public enterprises are not covered by the PPP Proclamation. Fully privately developed infrastructure, such as power plants, which still have contracting relationships with public enterprises, for instance, where the public enterprise is an off-taker, under a long term agreement, are not excluded based on the PPP legal framework in Ethiopia. The structure for any PPP project in Ethiopia is defined by the Feasibility Study which is to be carried out by the Contracting Authority. Globally, there are certain features of PPPs which describe more common PPP contracts. Figure 1-3 has been revised to include some of the more common PPP contract types seen globally and which are permissible under the Ethiopian PPP legal framework.

Figure 1-4: Examples of PPP Contract Types[15]

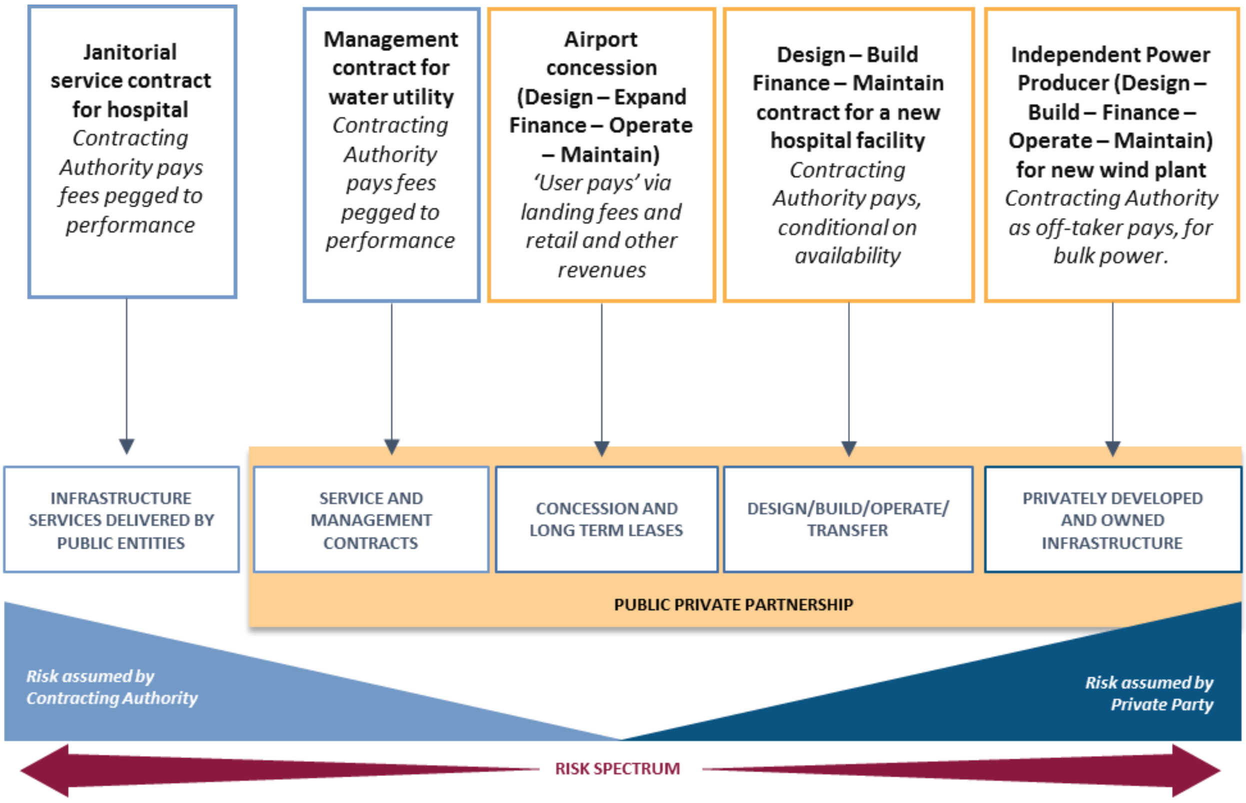

[15] Source: Public-Private Partnerships Reference Guide Version 2.0, International Bank for Reconstruction and Development / The World Bank, Asian Development Bank, and Inter-American Development Bank, 2014

Source: CPCS

- **MANAGEMENT CONTRACTS**

Management contracts or "performance-based management contracts" are typically employed by governments to acquire technical expertise to assist in managing public sector organizations (whether as traditional parastatals or commercialized enterprises). Under management contracts, contracting authorities may sign Operations and Maintenance (O&M) contracts under which for a designated period, usually short term (3 – 5 years) – technical experts supervise the management of an infrastructure facility, usually with the objective of transferring technical and operational expertise to the managers of the host organization. Under these arrangements, the private party typically bears no commercial or revenue risk, i.e. risk that public demand for the service will be insufficient and so result in low revenues accruing to the private party.

Typically, under a management contract, the private party is paid a predetermined rate for labour and other anticipated operating costs. To ensure that the quality of work performed by the private party meets the minimum expected performance levels required by the contracting authority, there are often minimum key performance indicators (KPIs) in the management contract agreement, assigned to the private party against which pre-specified penalties or incentives may be tied to performance.

- **LEASE AGREEMENTS**

Lease agreements represent an alternative approach to service and management contracts, which pushes for more responsibility and incentives toward the private party. Under such arrangements, the contracting authority cedes greater authority to the private party for managerial decisions, as opposed to advisory services.

The contractual framework consists of: clearly defined operational and/or service performance standards and outputs, thereby holding the private party to account; provisions that specify the legal rights and responsibilities of the parties under the agreement, thereby protecting the private party against arbitrary interference in management decisions.

Instead of receiving a fee for services rendered, the private party obtains the commercial revenue generated by the defined business and, in return, pays a lease fee for the use of the assets. Ownership of the assets remains with the contracting authority. Typically, the agreement will specify responsibility for investment over the course of the agreement. Investments to expand the asset base and/or rehabilitation of the existing asset base are usually but not invariably the responsibility of the contracting authority. The private party will in most instances be responsible for day to day maintenance of the assets whereas the Contracting Authority will be in charge of more structural and fundamental maintenance.

- **CONCESSIONS**

Concessions are essentially an extension of the lease concept. The contracting authority concedes the right to ownership and management of the domain of the concession for a specified duration, with ownership to be returned to the concession authority at the end of the concession period. The duration of the concession depends upon a number of factors, the most important being the time required to recover initial investments made by the private party (the

concessionaire). There is a grey area between leases and concessions, depending on issues such as the extent to which the private party is responsible for financing investments. Leases are more commonly used for project contracts where the government remains responsible for investments, and the private partner is only responsible for ordinary maintenance and operation, whereas in concessions the private party is also usually responsible for capital expenditure.

A concession is therefore an arrangement under which a contracting authority, owner of the infrastructure, delegates to a private party the responsibility for providing and maintaining a specified level of service to the users in exchange for the right to collect revenue from those users.

- **AFFERMAGE**

Affermage is a type of leasing arrangement (similar to a concession) under which an operator takes over and runs public infrastructure and collects revenue from customers, but does not undertake and finance new investment (hybrid approach). Investments, particularly in fixed infrastructure, remain the responsibility of the public partner. The operator either makes a specified lease payment to the contracting authority (under a simple lease), or shares revenues according to a predetermined formula (under an affermage).

- **BUILD-OPERATE-TRANSFER (BOT)**

This is the simple and conventional PPP model where the private partner is responsible to design, build, operate (during the contracted period) and transfer back the facility to the public sector. Role of the private sector partner is to bring the finance for the project and take the responsibility to construct and maintain it. In return, the public sector will allow it to collect revenue from the users. BOT schemes are favoured in developing new standalone assets.

In some countries the term BOT tends to be used as a generic term for all at risk PPPs whereas others list many model variants depending on the specific aspects of the project included in the PPP contract (level of investment, ownership control, risk sharing, technical collaboration, duration of the project, financing mode, etc.). Different jurisdictions use different nomenclature to describe similar projects. We focus on a small number related to large infrastructure construction types of projects.

- **DESIGN-BUILD-FINANCE-MAINTAIN-OPERATE (DBFMO)**

Design-Build-Maintain-Operate (DBMO), Design-Build-Finance-Maintain-Operate (DBFMO) and other Public Finance Initiatives (PFI) schemes as the names imply requirement elements of private and public sector financing. Under DBMO schemes, the contracting authority owns and finance the infrastructure construction. The private party is paid for the turnkey construction and is thereafter paid an operating fee over the years that the private party operate the infrastructure. Under DBMO contracts, the private party designs, builds the asset to meet agreed specifications and outputs. The private party assumes minimal financing risk for the infrastructure, but will be required to operate the infrastructure to outputs derived from the infrastructure specifications.

In DBFMO contracts, the private party assumes the infrastructure financing risk and is entitled to recoup its cost from operating the asset (i.e. from end user tariffs) and in some cases with support payments from the contracting authority, while meeting agreed upon performance indicators.

1.3.3 RISK ALLOCATION UNDER DIFFERENT PPP MODES

The following table provides a high level illustration of the main risks allocated over different PPP modes.[16]

Figure 1-5: Contractual forms of PPP and Risk Levels

PPP Model / Risks	Management Contract	Operations Concession	DBFM and PFI	DBFMO BOT
Residual Asset Value	● (Public)	● (Public)	● (Public)	● (Public)
Construction	● (Public)	● (Public)	● (Private)	● (Private)
Demand	● (Public)	● (Private)	● (Public)	● (Private)
Performance	● (Private)	● (Private)	● (Private)	● (Private)

● Public
● Private

[16] DBFM Design Build Finance and Maintain, PFI Private Finance Initiative, DBFMO Design Build Finance Maintain and Operate, BOT Build Operate and Transfer

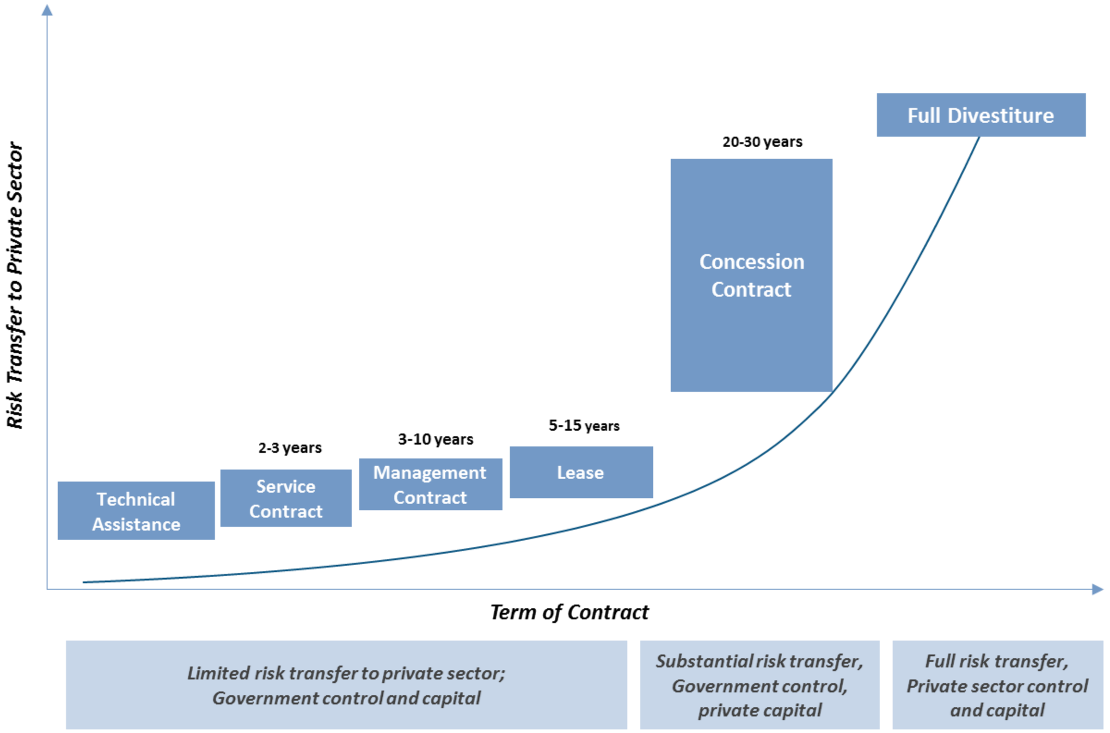
Risk Transfer to Private Sector
Full Divestiture
20-30 years
Concession Contract
2-3 years
3-10 years
5-15 years
Technical Assistance
Service Contract
Management Contract
Lease
Term of Contract
Limited risk transfer to private sector; Government control and capital
Substantial risk transfer, Government control, private capital
Full risk transfer, Private sector control and capital

1.4 OVERVIEW OF PPP DEVELOPMENT PROCESS

1.4.1 PPP DEVELOPMENT LIFE CYCLE

Identifying, developing, implementing and monitoring a project as a PPP involves a series of steps and should be undertaken following a clear process. This guideline organises the PPP process into a sequence of five phases based on the PPP lifecycle from identification to project end; it is helpful to break up this process into phases to help focus on the distinct life cycle elements, and in order to facilitate effective management and delivery. These phases are as follows:

i. PPP Project identification & suitability analysis

ii. PPP Appraisal & preparation (feasibility study)

iii. Structuring, draft contract & tender documents

iv. Tender and award (selection and contracting of the private partner)

Chapters 3-7 provide detailed guidance through these phases of development.

v. PPP Contract management and performance review

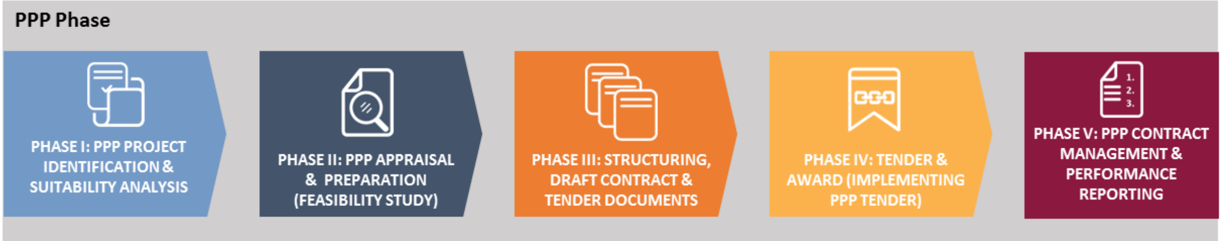

List of Documents Provided in Appendix A - PPP Glossary and Key Concepts
1. Part 1: Glossary
2. Part 2: PPP Toolkits and Reference Documents

1.4.2 PPP PROCESS MAP

PPP Development Phases: Overview of Tasks

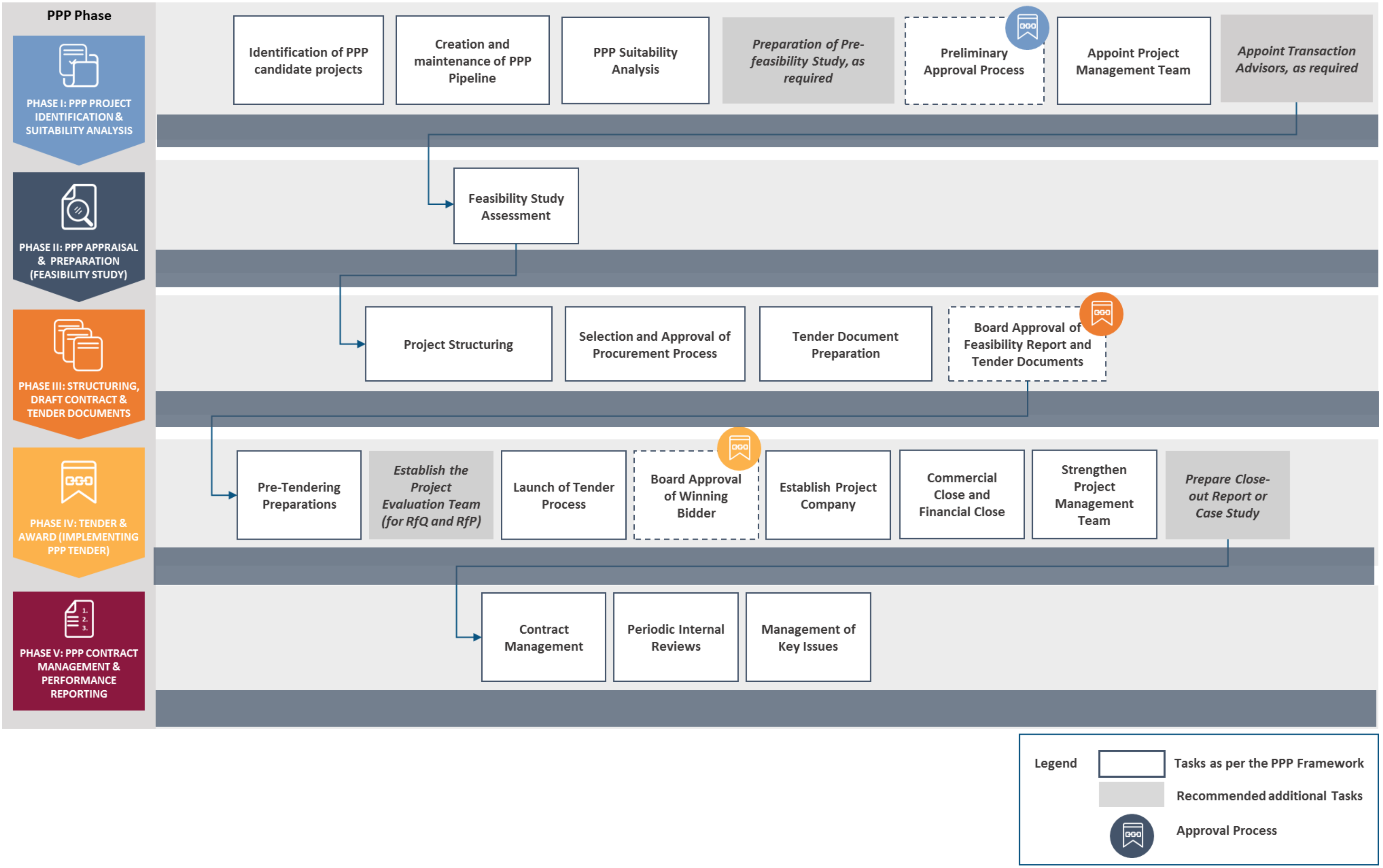

2 ROLES AND RESPONSIBILITIES

This Chapter identifies the main stakeholders involved in the PPP process and details their roles and responsibility in the PPP cycle.

2.1 PARTIES INVOLVED IN THE PPP PROCESS

A typical PPP would involve the PPP Board; the PPP DG; the Contracting Authority and other agencies that would be involved in planning, delivery or regulation of the Public Service Activity.

The institutional framework for PPPs identifies the following bodies to be involved in the conceptualizing, structuring and procuring PPPs: (i) PPP Board, (ii) Ministry of Finance (MOF) which is also the chairperson of the PPP Board, (iii) PPP Directorate General (PPPDG) and (iv) Contracting Authority.

Contracting Authorities are a Public Entity (Public Bodies or Public Enterprises[17]) intending to enter into a PPPP Agreement with a Private Party[18] and legally mandated to be responsible for the infrastructure service to be delivered by way of PPP[19].This will require that prior to entering any PPP contract, the particular Contracting Authority must have by law the authority over the infrastructure to be developed by PPP.The legal framework establishes that a Public Entity legally mandated to be responsible for a Public Service Activity[20] shall also be the Contracting Authority. Where the Public Service Activity is under the responsibility of more than one Public Entity, then the PPP Board shall select the appropriate Contracting Authority for the specific PPP project[21].

Additional bodies, which also have important constitutional roles in delivery Public Services Activities and thus relevant for PPPs, include: (i) Planning and Development Commission (PDC), (ii) National Bank of Ethiopia (NBE), (iii) Environment, Forest and Climate Change Commission (EFCCC), (iv) Ethiopian Investment Commission (EIC), and (v) Line Ministries.

---

[17] Public Bodies are organs of the federal government wholly financed by the Federal Government and Public Enterprises are enterprises fully owned by the Federal Government.

[18] Article 2(2) of the PPP Proclamation

[19] Article 6 (1) of the PPP Proclamation

[20] Article 2 (12-a) and Article 6(1) of the PPP Proclamation

[21] Article 6(20) of the PPP Proclamation

Figure 2-1: Parties Involved in PPP Process

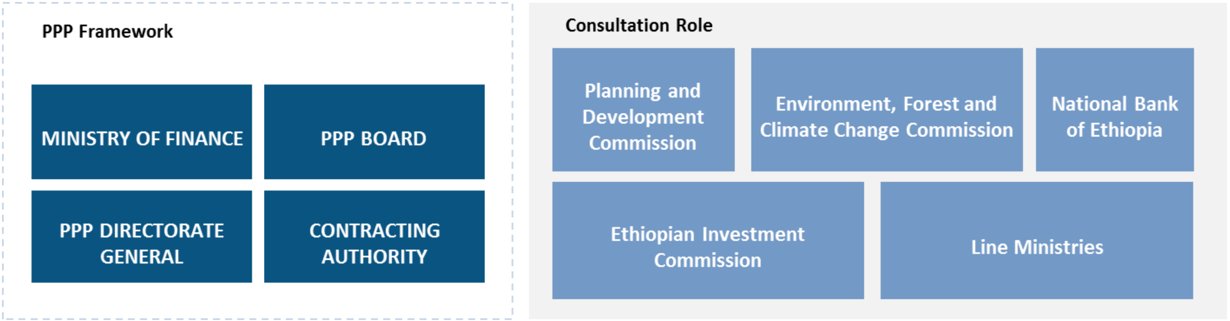

The composition and responsibilities of each stakeholder group are presented below.

Table 2-1: Core Relevant Function and Composition

Key Stakeholders:	PPP Board	PPP Directorate General (PPPDG)	Contracting Authority (CA)	Project Management Team (PMT)	Ministry of Finance (MOF)
**Core Relevant Function**	**Function**<br>▪ Responsible for granting approvals at key milestones in the project development process including approval to carry out feasibility study, approval to proceed to tender and approval to award the project.<br>**Enabling Instrument**<br>→ PPP Proclamation (article 9)	**Function**<br>▪ Act as a Secretariat to the PPP Board<br>▪ Develop and Manage Ethiopia's PPP Framework<br>▪ Develop Capacity within the Public Sector to Deliver and Manage PPP Projects<br>▪ Participate in the Development and Implementation of PPP Projects:<br>→ Identify potential PPP projects and participate in selection of projects to be undertaken as PPPs<br>→ Support in the development of PPP projects, in coordination with contracting authorities, including the identification, feasibility and structuring phases,<br>→ Lead the procurement and award of PPP projects, in coordination with contracting authorities<br>→ Provide assistance to contracting authorities through active participation in the PPP Project Team established for each project at all stages of the project lifecycle<br>→ Monitor and evaluate the implementation of PPP projects, in coordination with the CA and PMT.[22];<br>▪ Serve as a Center of Information and Expertise on PPPs in Ethiopia<br>**Enabling Instrument**<br>→ PPP Proclamation (article 12) and PPP Directive (article 7)	**Function**<br>▪ Responsible for the initiation of PPP projects, supporting the PPP DG in the procurement process; signing of agreements and implementation of PPP projects it administers.<br>▪ In order to fulfill its mandate, the CA forms a Project Management Team.<br>▪ CA is responsible for ensuring that the Project Management Team fulfills its responsibilities[23]<br>**Enabling Instrument**<br>→ PPP Proclamation (article 13) and PPP Directive (article 9)	**Function**<br>▪ On behalf of the CA, the PMT is responsible for the development of PPP projects and the monitoring of the implementation of PPP agreements.<br>**Enabling Instrument**<br>→ PPP Proclamation (article 14) and PPP Directive (article 10)	**Function**<br>▪ PPPDG is established within MOF.<br>▪ MOF is responsible for adequately staffing PPPDG<br>▪ and is the chair of the PPP Board<br>▪ Responsible for pre-approval of the PPP Board<br>**Enabling instrument**<br>→ PPP Proclamation (article 16)<br>→ PPP Proclamation (article 10)<br>→ PPP Proclamation (article 10)<br>→ PPP Proclamation (article 8(2))
**Composition**	**Composition**<br>▪ Ministry of Finance (MOF)<br>▪ National Bank of Ethiopia<br>▪ Ministry of Water, Irrigation and Electricity<br>▪ Ministry of Transport<br>▪ Ministry of Public Enterprises<br>▪ Planning and Development Commission<br>▪ Ministry of Peace<br>▪ Two members from the private sector appointed by the MOF<br>**Enabling Instrument**<br>→ PPP Proclamation (article 8.1)	**Composition**<br>▪ Established within the Ministry of Finance<br>▪ Three Departments:<br>1. **PPP Framework Management and Contract Support Directorate**. Main responsibilities include: (i) supervision of legal due diligence work and providing reviews of draft PPP contracts and tender documentation, (ii) review and drafting of relevant policy and legislative documents, the provision of advice on the interpretation and application of the PPP Framework, as well as the review of contracts on behalf of the PPP DG, such as contracts with external advisors or transactions advisors.<br>2. **PPP Capacity Building, Knowledge Management and Communications Directorate**. Main responsibilities include: (i) monitoring and communicating information to the various stakeholder communities associated with PPPs, (ii) coordinating the organization of Capacity Building events such as training courses, seminars, workshops, etc., (iii) Knowledge Management.		**Composition**<br>The PMT is composed of:<br>▪ Team leader from the CA<br>▪ Representative from the PPPDG<br>▪ Personnel from the CA with financial, legal and technical skills<br>▪ Representatives of other key stakeholders, as appropriate<br>▪ Additional information on the composition of the PMT is provided in section 3.3.2<br>**Enabling Instrument**<br>→ PPP Directive (article 9)	▪ Debt Management Directorate: consulted to assess contingent liabilities and to confirm government commitments/support.<br>▪ Legal Department: review of the PPP draft agreement and consultation throughout the PPP process as relevant<br>▪<br>**Enabling Instrument**<br>→ PPP Proclamation (articles 18(2) and 47) and PPP Directive (articles 13(3) and 15).

[22] Source: PPPDG's Capacity Building Plan, July 2018
[23] Article 9(3) of the PPP Directive

Key Stakeholders:	PPP Board	PPP Directorate General (PPPDG)	Contracting Authority (CA)	Project Management Team (PMT)	Ministry of Finance (MOF)
	→ Functioning of the Board detailed in the PPP Directive (article 6)<br>→ PPP Board Guidelines	3. **PPP Project Development and Monitoring Directorate**. (i) Reviewing initial PPP Suitability Analysis, (ii) review feasibility study; (iii) lead procurement process; (iv) monitor and evaluate the progress of implementation of PPPs, (v) review the overall PPP program to determine whether the program as a whole is delivering the expected benefits to the Ethiopian economy and to build on past experience to improve future project implementation.<br>**Enabling Instrument**<br>→ Office of PPPDG - Capacity Building Plan Document (June 2018)			

Table 2-2: Key Responsibilities throughout the PPP Project Development Lifecycle as per the Regulatory Framework in Ethiopia

	PPP Board	PPPDG	Contracting Authority	Project Management Team	MoF [24]	Planning And Development Commission	Ethiopian Investment Commission	National Bank of Ethiopia	Environment, Forest and Climate Change Commission
**PHASE I: PPP PROJECT IDENTIFICATION AND SUITABILITY ANALYSIS**									
i. Identifying potential PPP projects		✓	✓						
ii. Carrying out suitability analysis of candidate projects		✓	✓						
iii. Consultation during the suitability analysis process of candidate projects, as relevant					✓	✓			
iv. Carrying out Pre-Feasibility study, as relevant[25]			✓						
v. When required, PPP Board identifies appropriate Contracting Authority (Public Body or Public Enterprise) and instruct it to carry out a project as a PPP	✓								
vi. Granting preliminary approval for a project to be implemented as a PPP					✓				
vii. Granting approval for a project to be implemented as a PPP	✓								
**PHASE II: PPP APPRAISAL & PREPARATION (FEASIBILITY STUDY)**									
viii. Hiring of external advisors to support the PMT in carrying out its duties		✓	✓		✓				
ix. Carrying out feasibility study			✓	✓					
x. Granting environmental clearance after the ESIA study is carried out for relevant sectors[26]			✓						✓
xi. Reviewing feasibility study		✓							
xii. Analyzing potential constraints associated with any financial commitments		✓			✓				
xiii. Reviewing the project's impact in terms of foreign currency requirement		✓			✓			✓	
**PHASE III: STRUCTURING, DRAFT CONTRACT & TENDER DOCUMENTS**									
xiv. Preparing draft tender documents and PPP contract				✓					
xv. Reviewing draft tender documents and PPP contract		✓			✓				
xvi. Granting approval for Government Support	✓				✓				
xvii. Recommending specific tax or other incentives for a particular project to relevant authority	✓				✓				
xviii. Approving the procurement process to implement	✓								
xix. Granting approval for the PPPDG to proceed to tender or to Direct Negotiation	✓								
**PHASE IV: TENDER & AWARD (IMPLEMENTING PPP TENDER)**									
xx. Implementing PPP tender process with PPPDG's lead and support from CA and PMT		✓	✓	✓					
xxi. Negotiating with successful bidder with PPPDG's lead and support from CA and PMT		✓	✓	✓					
xxii. Consultation during negotiation regarding potential project structure changes					✓				
xxiii. Granting approval if there is significant change (i) to the project structure previously approved by the Board prior to the tender (ii) and to Project Agreement.	✓								
xxiv. Reviewing the Project Agreement prior to approval by PPP Board[27]		✓			✓				
xxv. Granting approval to proceed to contract award	✓								
xxvi. Signing Project Agreement with Private Party			✓		✓[28]				
xxvii. Facilitating issuance of commercial registration and/or investment permits required		✓	✓	✓					
xxviii. Granting issuance of commercial registration and/or investment permits required							✓	✓	✓

[24] MoF's Debt Management Directorate consulted during (i) the suitability analysis process of candidate projects, as relevant (Phase I), (ii) analysis of potential constraints associated with any financial commitments (Phase II), (iii) negotiation regarding potential project structure changes (Phase IV), (iv) to provide economic support and guarantees to ensure sustainability, implementation and/or financial viability of the PPP project (Phase V). MoF's Legal department consulted during (i) review of draft tender documents and PPP contract (Phase III), (ii) review the Project Agreement prior to approval by PPP Board (Phase IV)

[25] This is based on CPCS's recommendation

[26] This is based on CPCS's recommendation

[27] This is based on CPCS's recommendation

[28] The MoF will sign the implementation agreement

	PPP Board	PPPDG	Contracting Authority	Project Management Team	MoF [24]	Planning And Development Commission	Ethiopian Investment Commission	National Bank of Ethiopia	Environment, Forest and Climate Change Commission
**PHASE V: PPP CONTRACT MANAGEMENT & PERFORMANCE REPORTING**									
xxix. Managing disputes (as per PPP Agreement)		✓							
xxx. Managing complaints	✓	✓			✓				
xxxi. Renegotiations with PPPDG's lead and support from CA and PMT		✓	✓	✓					
xxxii. Assisting private party in obtaining land rights related to the project site			✓						
xxxiii. Providing economic support and guarantees to ensure sustainability, implementation and/or financial viability of the PPP project					✓				
xxxiv. Monitoring Key Performance Indicators (KPI)		✓	✓	✓	✓				
xxxv. Submitting periodic reports to the PPPDG and the Board				✓					
xxxvi. Providing information to the public on the implementation/performance of PPP projects		✓			✓				
xxxvii. Ensuring that the transfer of assets at the expiry or early termination of a PPP Agreement is consistent with the terms and conditions of such agreement		✓	✓	✓					

3 PHASE I: PPP PROJECT IDENTIFICATION AND SUITABILITY ANALYSIS

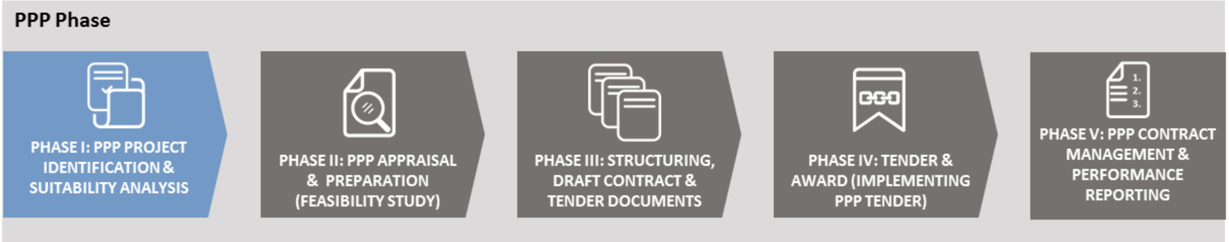

This chapter summarizes the processes and tasks involved for selecting candidate PPP projects, conducting the Pre-Feasibility study, and deciding which projects should be approved for full PPP feasibility studies. This early assessment of a potential PPP project evaluates whether the project is worth pursuing and incurring additional costs in developing into a PPP.

Figure 3-1: Overview of Tasks under Phase I

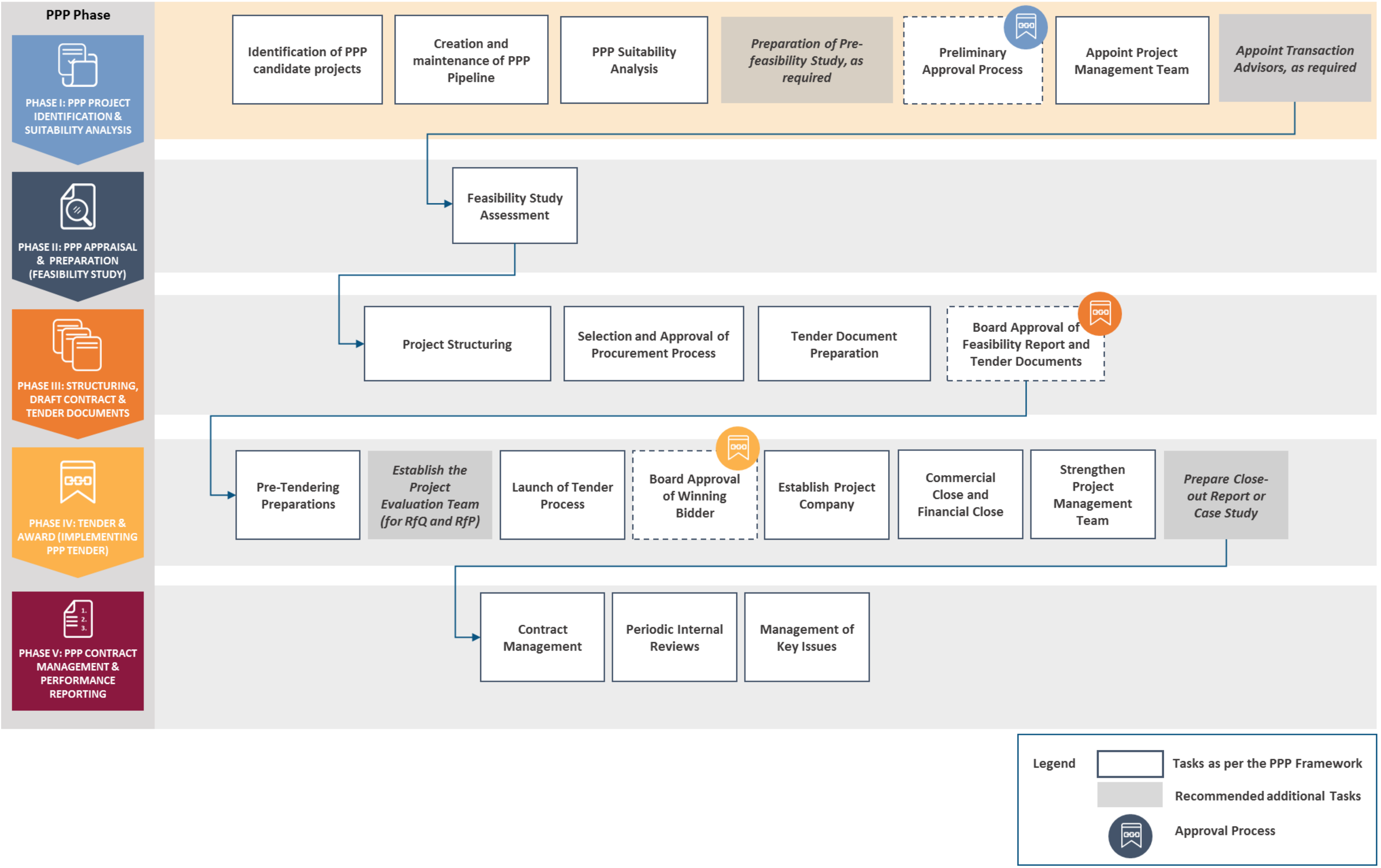

Figure 3-2: Phase I Process and Key Stakeholders

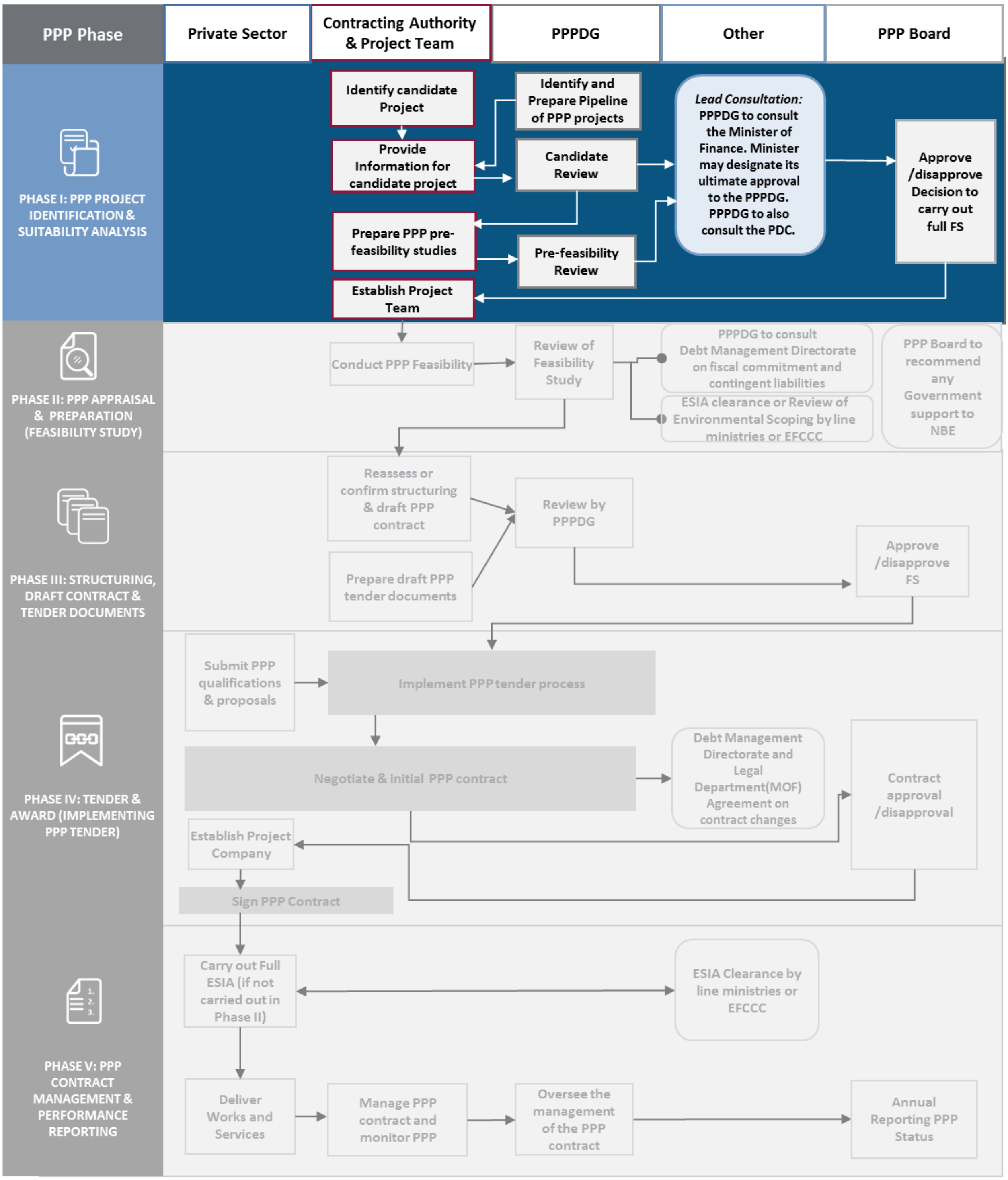

Figure 3-3: Phase I Process and Key Stakeholders – Unsolicited Proposals

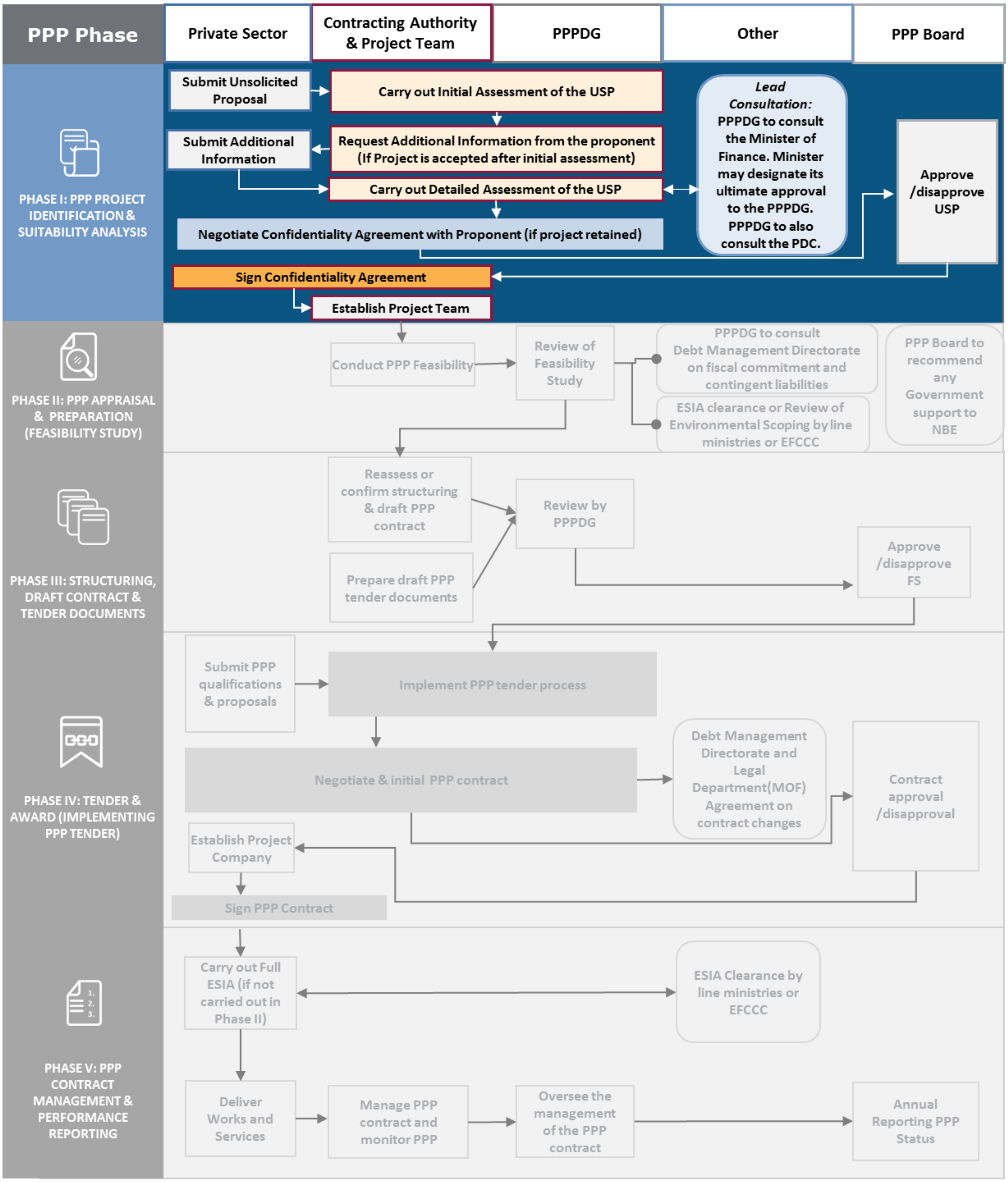

3.1 PROJECT IDENTIFICATION

The proclamation allows for three routes for project identification: (i) through the PPPDG project pipeline; (ii) projects identified by public entities; and (iii) unsolicited proposals submitted by the private sector.

3.1.1 PPP DIRECTORATE GENERAL

Articles 12 and 15 of the PPP Proclamation and article 11 of the PPP Directive indicates that the PPPDG can identify PPP projects to be approved by the PPP Board. Once the PPPDG has identified a potential PPP project, it submits it to the relevant Contracting Authority to prepare the PPP Suitability Application and if required the Pre-Feasibility study.

BEST PRACTICE
**Decision-making at Phase I**<br><br>Users of this Guideline should note that the procedures of Phase I generally require making decisions about potential projects based only on initial, available and limited information about potential projects.<br><br>At this early stage, project information is often limited and qualitative in nature. The goal of this first Phase is therefore to determine if a given project initially *appears* to be a promising candidate that could be later made into a viable and attractive PPP project, and whether or not additional human and financial resources should be spent on conducting a detailed feasibility analysis in the subsequent Phase II.

3.1.2 PUBLIC ENTITIES

Potential PPP projects can also be identified by Public Entities[29] which can be Public Bodies or Public Enterprises. The PPP Policy establishes that Public Entities, as part of their normal infrastructure planning process, would identify projects that are candidate PPP projects. Such projects would have already been defined and prioritized for implementation by the Public Entities. One of these Public Entities is the Ministry of Finance.

The candidate PPP project would then be submitted to the PPPDG for inclusion on the PPP project pipeline.

3.1.3 UNSOLICITED PROPOSALS FROM THE PRIVATE SECTOR

The PPP Proclamation[30] allows for Unsolicited Proposals (USP) to be submitted by the Private Party. USPs are submissions from private parties to implement as PPPs specific Public Service Activities that have not been previously identified as candidate PPP projects. USPs could potentially (i) benefit the government from the knowledge and innovative solutions of the private sector to address infrastructure gaps, (ii) provide information of where market interest exists, (iii) reduce financial and technical bottlenecks at an early stage of the PPP process by providing preliminary feasibility studies. However, USPs might not be aligned with Ethiopia's

[29] Article 15 of the PPP Proclamation
[30] Chapter 9 of the PPP Proclamation

national strategy, be in the public interest and could divert government attention from a planned and structure approach to infrastructure development.

USPs have to be addressed to the PPPDG which will carry out a review in consultation with the relevant Public Entity responsible for the provision of the Public Service Activity. Following receipt and preliminary examination of the USP, the PPPDG shall make use reasonable efforts to inform the proponent whether or not the project is considered for further evaluation. During the preliminary evaluation, the PPPDG will examine if the project (i) is aligned with Ethiopia's national strategy, (ii) provides a public service activity in a priority sector, (iii) potentially provides innovative solutions infrastructure gaps, and (iv) is considered to be potentially in the public interest. Following this preliminary assessment, the PPPDG will invite the proponent of the project to be of potential interest to submit as much information on the proposed project as is feasible.

A USP submission will include the following documents[31] and information in order for the PPPDG to evaluate the suitability of the project:

- Description of the Proposed Project, including a high-level design, sketches, or alignment maps;
- Preliminary assessment of the public need for the project, including a description of the benefits to society and alignment with the Ethiopia's Growth and Transformation Plan;
- Description of the environmental and social features of the project;
- Preliminary economic feasibility assessment or cost benefit analysis;
- Preliminary technical description of the project;
- Preliminary financial feasibility assessment and a preliminary funding and financing plan;
- Preliminary operating plan for the project;
- Preliminary assessment of project risks;
- Description of the type and range of Government support expected to be required.

The USP will also need to provide to the PPPDG, evidence of experience and qualifications in similar project implementation.

USPs may be reviewed and accepted, in so far as it is not related to a project which has received the PPP Board approval for study or implementation as a PPP project.

USPs must follow the same process as candidate PPP projects identified by the PPPDG or a Public Entity. **Once the PPPDG has received all the relevant information from the proponent, it evaluates the suitability of the USP to be carried out as a PPP using the PPP suitability process defined in section 3.2.1, to which is added the evaluation of the proponent's qualifications.** During this evaluation process, the PPPDG will consult with the Ministry of Finance and the

[31] Best practice recommended in the Policy Guidelines for Managing Unsolicited Proposals in Infrastructure Projects – Volume II World Bank Group

Planning and Development Commission. The PPPDG may contact the proponent with requests for clarification or additional information. The information provided by the proponent should be sufficient enough for the PPPDG to take its decision on the PPP suitability and must not necessitate a Pre-Feasibility study. Once the evaluation is carried out, the PPPDG will inform the proponent of its decision. If the project is found to be suitable for a PPP, and prior to submission and approval by the PPP Board, a confidentiality agreement will be signed between the Contracting Authority and the proponent[32]. The confidentiality agreement will at least cover:

- Confidentiality obligations for both parties relating to the content of the studies prepared by the proponent
- The terms and conditions for the transfer of all studies and rights over the project to the Contracting Authority or another Person should the proponent not be awarded the project
- The allocation of bonus points during the tender process or the allocation of a financial compensation for studies undertaken by the proponent should the proponent not be awarded the project in accordance;
- The next steps for the development of the project and if appropriate the allocation of responsibilities to carry out these steps.

Once the PPPDG has evaluated the suitability of the USP and a draft confidentiality agreement between the Contracting Authority and the proponent has been prepared, the project will be submitted to the PPP Board for approval.

Following approval by the Board, the confidentiality agreement is signed by the Contracting Authority and the proponent. A feasibility study is then carried out by the Contracting Authority. In the case where the proponent has provided a detailed feasibility study, the Contracting Authority will carry out a verification of the study.

The project then goes through a competitive procurement process: single stage open bidding, two stage open bidding and competitive dialogue. The identity of the proponent, the detailed allocation of bonus points or the amount of the potential compensation to the proponent will be indicated in the competitive selection procedure documents.

The allocation of bonus points to the proponent by the PPPDG which can apply to the technical and/or financial score will be negotiated with the proponent as part of the confidentiality agreement. Similarly the financial compensation the proponent will receive should it not be awarded the project as well as the maximum budget available for compensation will also be negotiated and figure in the confidentiality agreement. The bonus points awarded and financial compensation will be based on the scale, size and complexity of the project. It is recommended that the bonus awarded remains small, in order not to discourage potential bidders and encourage equal bidding conditions. The financial compensation will cover the cost of preparing the proposal and studies undertaken by the proponent. This will be paid by the successful bidder as a condition precedent to financial close and commencement of the works and/or services[33].

---

[32] Article 24 of the PPP Directive

[33] Article 25 of the PPP Directive

The bonus and financial compensation mechanisms will be included in the RfP when the project goes to tendering.

The project can also be awarded through direct negotiation if it meets the conditions[34] for use of Direct Negotiation as prescribed in the PPP Proclamation:

- Where there is an urgent need in the provision of the service;
- Where the project is of short duration and the anticipated initial investment value does not exceed the amount to be specified by directive;
- Where the project involves infrastructure projects related to national defense or national security;
- Where there is only one source capable of providing the required service;
- When an invitation to the prequalification proceeding or a request for proposals has been issued but no applications for qualification or proposals were submitted or all proposals failed to meet the evaluation criteria set forth in the request for proposals;
- Where a PPP project, of limited value and length is ancillary and the necessary complement to a mining, industrial or other type of investment and must be carried out by the private sponsor of such mining, industrial or other type of investment.

The type of procurement to be used for the project will be decided during the structuring of the project, once the feasibility study is carried out by the Contracting Authority.

Given the substantial challenges related to USPs presented in this sub-section, this process is not recommended unless exceptional circumstances.

[34] Article 39 of the PPP Proclamation

Figure 3-4: PPP Suitability

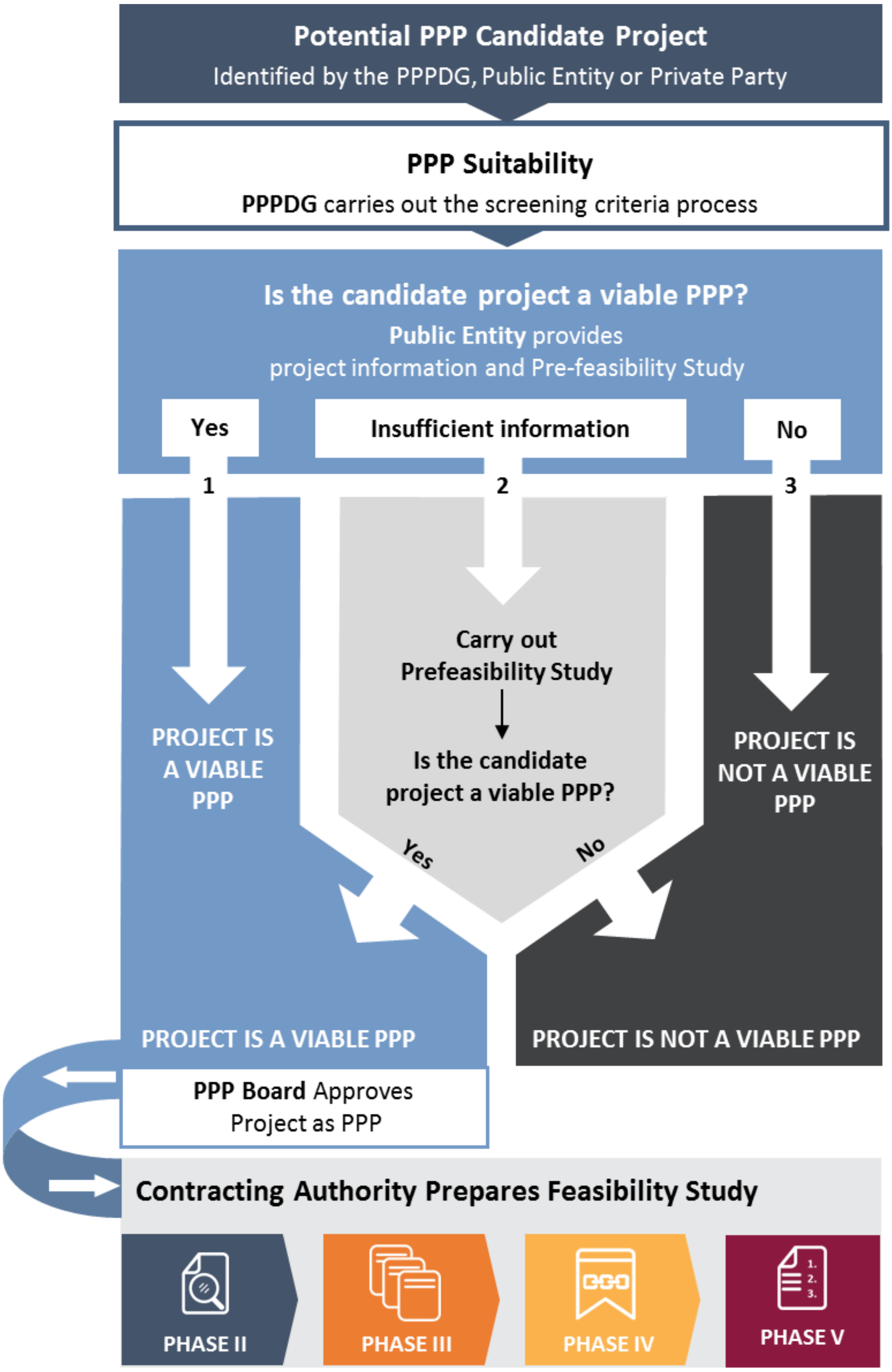

3.2 PPP SUITABILITY AND PRELIMINARY APPROVAL PROCESS

3.2.1 PPP SUITABILITY EVALUATION

PPP candidate projects identified by the PPPDG, a Public Entity or a Private Party (Unsolicited Proposal) will first go through a PPP Suitability Evaluation.

The PPP Suitability Evaluation has two components: (i) a preliminary screening and (ii) a prioritization matrix. The PPP Suitability Evaluation methodology used is based on the one developed in the PPP Projects Screening Report[35].

The first step in the PPP Suitability Evaluation is for the PPPDG to provide the appropriate Public Entity a PPP Suitability Application to complete (Refer to **Appendix B – Part 1: PPP Suitability Application).** This application has two components (i) a preliminary screening and (ii) a prioritization matrix. For candidate projects identified by the PPPDG or USPs, the PPP Board identifies the appropriate Public Entity to complete the application form. For candidate projects identified by a Public Entity, the same Public Entity completes the application form.

3.2.1.1 PPP Preliminary Screening

The first component of the PPP Suitability Evaluation is the Preliminary Screening.

The Preliminary Screening is designed to address the PPP pre-approval criteria, defined in the PPP Directive[36], as follows:

- Whether the project is consistent with Ethiopia's development objectives and national plans;
- Whether there is sufficient demand for the project outputs;
- Whether the project will give substantial benefit to the public, if private sector is preferred to provide the service and if the project is expected to be able to transfer meaningful and appropriate risks to a private sector partner;
- Whether a project is likely to deliver Value for Money;
- Whether sufficient financial resources, including those required for expected government support, are expected to be available for the project; and
- Whether the Contracting Authority has sufficient capacity and resources to appropriately prepare and implement the project.

The Preliminary Screening will also provide an answer to the following questions:

- Is the output or services from the project affordable to users/ customers?
- Is the project large enough to justify transaction costs, i.e. above USD 50 million, and of a "bankable" size?
- Are the environmentally and social impacts of the project acceptable and can they be mitigated using Ethiopian and international standards?

[35] PPP Project Pipeline Screening and Initial Feasibility Assessment of Potential Infrastructure PPPs Report, Ministry of Finance (August 2018)
[36] Article 12(1) of the PPP Directive

- Is the project replicable or scalable?
- Can appropriate and relevant project risks be allocated to the private sector?
- Is the business and revenue model adequate to attract private investment?

The Preliminary Screening is provided in **Appendix B (**Refer to **Part 1. PPP Suitability Application [Preliminary Screening]**).

At this initial stage, for projects identified by the PPPDG or the Public Entity, concise information should be provided by the Public Entity, to roughly estimate whether a potential project could be carried out as a PPP. For USPs, the documentation and information provided by the proponent must be comprehensive and exhaustive enough for the Public Entity to fill out the preliminary screening form with the support of the PPPDG. The questions can be answered a priori, without having detailed data that would be obtained at the feasibility stage. **All questions must be answered positively for a project to go the next step which is the prioritization phase. Otherwise, a PPP Pre-Feasibility study is required.**

3.2.1.2 PPP Prioritization

The second phase of The PPP Suitability Evaluation is the PPP Prioritization, where the candidate project is scored according to a specific framework. Candidate PPP projects that have passed the Preliminary Screening are subjected to the prioritization framework.

This prioritization is carried out using the form provided in **Appendix B** (Refer to **Part 1. PPP Suitability Application [Prioritization]**). The objective of the prioritization exercise is to identify the potential projects that will be submitted to the PPP Board for approval to be procured as a PPP. The Public Entity is responsible for filling in the application and providing the necessary information with the support of the PPPDG for the prioritization scoring. For USP, the Public Entity, with the support of the PPPDG, fill out the application based on documentation provided by the proponent. Once all the questions in the prioritization application are answered, the PPPDG carries out the scoring.

The two primary criteria used to prioritize projects are: (i) the Level and Forms of Support and (ii) the Fundamental Feasibility of the project (see table below). The sub-criteria of these two categories are listed in the table below. The form provided in **Appendix B** (Refer to **Part 1. PPP Suitability Application [Prioritization]**) defines each of these sub-criteria.

Table 3-1: Summary of PPP Projects Prioritization Matrix

PPP Prioritization		Scoring (1-4)
**Primary Criteria I - Level and Forms of Support**		
1	Government / Political Support	
2	Market Support	
3	Legal, Regulatory, Institutional Support	
4	Data and Information Availability	
5	Social and Environmental Sustainability	
**Sub-total 1**		/20
**Primary Criteria II – Fundamental Feasibility**		
1	Technical Feasibility	
2	Financial Feasibility / Private Sector Attractiveness	
3	Replicability	
4	Fiscal affordability	
5	Ease of Implementation	
**Sub-total 2**		/20
**TOTAL**		**/40**

The PPPDG prioritizes each potential PPP Project using a two-step approach. Each sub criteria is scored out of 4, with 4 representing the highest quality score and 0 the lowest. Explanation on how to score each criteria is also provided in **Appendix B** (Refer to **Part 1. PPP Suitability Application [Prioritization]**).

The total score of each project will be out of 40.

During this prioritization process, the PPPDG will consult with the Ministry of Finance and the Planning and Development Commission. The Ministry of Finance is consulted to discuss on the availability of funds and the availability of budget room to accommodate any government support required. The Planning and Development Commission is also consulted to establish if the project (i) fits with the country's national plan and (ii) is within the overall development projects/investment plans of the Federal Government.

The prioritization methodology establishes a minimum score of 28 for projects to be considered candidate PPP projects for further consideration by the Ministry of Finance and the Planning and Development Commission. The PPPDG has two alternatives for projects scoring below 28, (i) the project scored below 28 because of lack of data in which case a Pre-Feasibility study is required or (ii) the project scored below 28 because it is not suitable to be a PPP in which case it is not considered as a PPP. The PPPDG should assess the reason for the score below 28 and instruct the Public Entity accordingly.

BEST PRACTICE
**Initial PPP Suitability Evaluation**<br>**Avoid the temptation to Require Perfect Results:** Public managers should remember that this is only the first step in selecting potential candidate projects for PPP. This procedure does not promise that a given project will be feasible as a PPP, or commit the Contracting Authority to enter into a contract.<br><br>It is common in conducting a project selection procedure like this to face large variations in the quantity and the specificity of information that is available about each of a Contracting Authority's planned projects. Some may be well-advanced in their analysis and already have completed technical and economic feasibility studies, preliminary designs and environmental impact assessments. Others may only consist of a title and a single-page description of the need and general concept for a new project. If a project is a priority for the Contracting Authority, but very little information is available on it, it can still be determined as an appropriate candidate for PPP after a Pre-Feasibility Study is carried out.<br><br>Of all of the PPP project selection criteria of this procedure, the most important is that they should be clear priorities for the Contracting Authority. Public authorities should avoid the traditional assumption that priority projects should be reserved for conventional public procurement and public financing, while the lower priority or less-attractive projects should be offered to the private sector as PPPs. This has sometimes been referred to as offering-up the "worst first" for PPPs, and has not been successful.

Estimated Time Required: The amount of time required to complete this procedure depends considerably on the quality of the information available and also on the level of PPP knowledge of the public managers tasked with completing this procedure. It should not take more than 1 to 2 weeks for the Contracting Authority to complete this procedure.

3.2.2 PRE-FEASIBILITY STUDY

A project will require a Pre-Feasibility study, to be carried out by the Public Entity, when there is a lack of information to carry out the initial suitability evaluation or when the project is sizeable and complex with too many unknown parameters.

For each project, the prefeasibility study will provide responses and assessments for the **PPP Pre-Feasibility Checklist** provided in **Appendix B** (Refer to **Part 2. PPP Pre-Feasibility Checklist**).

The Public Entity will carry out a PPP Pre-Feasibility Study Report which consists of:

- A one-to-two-page summary description of the project's concept, including the basic information about the name, sector, location, investment size or capacity, key functions, and the basic rationale for the project, etc.
- The completed PPP Pre-Feasibility checklist with all questions answered based upon the information that is available

Following completion of the Pre-Feasibility, the Public Entity will submit this report and an updated PPP Suitability Application to the PPPDG. The PPPDG will then review the Pre-Feasibility

study and re-do the scoring of the project. The PPPDG will consult with the Ministry of Finance and the Planning and Development Commission during its review.

BEST PRACTICE

PPP Pre-Feasibility Study

In practice, this procedure is an excellent opportunity to identify the key challenges that face a PPP candidate project, and which should be the focus of subsequent analyses and project structuring decisions. Often times the result of this procedure is not a conclusion that "this project is clearly feasible across all indicators."

Rather the conclusion is more likely one of, "this is a priority project that is generally suitable as a PPP, but more detailed subsequent analysis should focus on determining what the affordability limit is, if the output standards can be expressed more clearly, whether demand risk should be shared between the public sector and the service operator, etc." This will give better focus to the planning and implementation of subsequent procedures, such as in Phase II.

Of all of the criteria included in this suitability checklist, the most important one is if the project can be described in terms of clear output levels of service. Experienced PPP practitioners of PPP in Europe and Australia have noted that if a given PPP project encounters problems later on during its tendering or financing phases, it is usually because of uncertainties about how the project's output standards have been defined and worded.

Therefore, it is strongly recommended that time and efforts be invested in drafting (and reviewing and revising) very clear output standards during this Phase I. This will be very helpful in preventing costly delays and problems during the later Phases.

Reviewing PPP Pre-Feasibility Study

In deciding whether or not to recommend a project, the PPP Unit should look beyond the technical details and estimates about project costs, etc. and consider the broader strategic and the key public policy issues involved. The overall strategic decision to proceed with a PPP is ultimately a political and a strategic one and requires clear and long-term political commitments to overcome the constraints to PPPs and to make sure that PPPs deliver clear and visible benefits to the public.

The PPPDG's reviewers should understand that this procedure is only a recommendation on whether or not to conduct a full PPP feasibility study. It is not a final or irrevocable decision to sign a binding, long-term PPP contract. As the key oversight body, the PPPDG will have other important opportunities during the preparation of the PPP project to review the latest analysis, progress, and costs & benefits of the PPP and to either approve its advancement to the next Phase, or to reject it.

For very large, strategic, or politically-sensitive PPP projects, the PPPDG should review this early PPP summary information and to make a recommendation on whether or not the given project should proceed to Phase II's PPP Feasibility Analysis. Doing this can avoid problems later on where a large PPP project could suddenly get stopped at the tendering or even the contract signing stage (after months or years of costly project preparation), because the PPPDG is

reviewing the project for the first time and deciding whether to approve or to deny its implementation.

Estimated Time Required: The amount of time required to complete this procedure depends clearly on the availability of information about the project (technical, economic, budgeting, environmental, etc.) either from existing project studies or from the line Ministry's or Contracting Authority's own planning, technical, and budgeting specialists. If this project information is already available, it may require about 2 weeks for a public authority to complete this procedure for a single project. However, if project information and estimates must be gathered from different design, construction, operational, budgeting, and planning specialists, it may take 3 to 4 weeks to complete this procedure for a given project.

3.2.3 APPROVAL TO PROCEED TO FEASIBILITY STUDY BY THE PPP BOARD

If the project scores above 28, then it could potentially become a PPP. The PPPDG submits the potential project along with its recommendation to proceed to procurement as a PPP first for pre-approval by the Ministry of Finance and then for approval by the PPP Board. Based on the recommendation of the PPPDG the PPP Board grants or denies approval to proceed to full feasibility study.

If the project is approved for procurement as a PPP, the PPP Board shall confirm or nominate, as relevant, the appropriate Contracting Authority.

Estimated Time Required: The amount of time required to complete the review and approval should take approximately 2 to 4 weeks, depending upon the size and complexity of the given candidate project.

3.3 ACTIONS UPON APPROVAL BY THE PPP BOARD TO PROCEED TO FEASIBILITY STUDY (NEXT STEPS)

3.3.1 UPDATE PPP PIPELINE

The PPPDG is mandated to maintain a PPP Project Pipeline[37], which will track projects at each phase of the PPP cycle. The template for the PPP Project pipeline is presented in **Appendix B. Part 3 - PPP Project Pipeline Form.** This PPP Project Pipeline is part of the proactive information disclosure management which is best practice for encouraging PPPs. The PPPDG will publicize its PPP pipeline online and using relevant print media as part of its General Procurement Notices.

The PPPDG has to update this PPP Pipeline as candidate projects move through the various stages of the PPP cycle. Candidate projects approved for procurement as PPPs should be indicated on the pipeline list. Similarly, potential PPP projects that have been denied *preliminary approval* or *approval to tender* will be moved to the "Projects not implemented as PPP"

[37] Article 11 of the PPP Directive

category. The PPPDG will report on the status of the projects in the pipeline to the PPP Board during its quarterly meetings.

On a regular basis, the PPPDG shall publish in a widely circulated national newspaper the entire list of the PPP Pipeline with a brief description of the stage of the PPP project. Where PPP projects are past commercial close, the announcement shall point readers to where additional information on project performance can be obtained.

At this early stage of the project life cycle before detailed feasibility, any information about the commercial parameters of the project, i.e. project infrastructure costs, anticipated end-user tariffs, government support required, shall be considered preliminary and thus not suitable for public circulation. If such information is circulated and later conflicted by feasibility conclusions, it could cause unnecessary confusion with some stakeholders (those without sophisticated understanding of Pre-Feasibility and feasibility details).

Information on anticipated demand for service and potential coverage area can be circulated if available. Such information tends to be drawn from existing infrastructure and sector master plans, which have already been through some rigorous analysis.

If no progress has been made with a project for around 18 months after approval by the Board to proceed to the feasibility stage, an investigation as to the status of the project will be carried out by the PPPDG with the support of the Contracting Authority. If the project is found to be no longer relevant and deemed to be no longer developed as a PPP, the PPPDG will update the PPP Board in order to have its approval to remove it from the PPP Project Pipeline.

3.3.2 APPOINTING THE PROJECT MANAGEMENT TEAM

Following preliminary approval of a candidate project by the PPP Board, the head of the Contracting Authority shall appoint a Project Management Team[38]. The Contracting Authority establishes a Project Management Team to, among other tasks, act on the Contracting Authority's[39] behalf in:

- Carrying out the PPP project appraisal;
- Preparing tender documentation and Project Agreements;
- Supporting the PPPDG implementing the PPP tender process;
- Preparing the Project Management Checklist;
- Monitoring the implementation of the PPP Agreement; and
- Liaising with all key stakeholders during the PPP project cycle

To provide effective guidance to the Project Management Team in carrying out this task, the Contracting Authority shall prepare a Terms of Reference for the Project Management Team. The Terms of Reference will include (i) the membership of the Project Management Team and their qualifications, (ii) the precise role and responsibilities of the Project Management Team on the PPP transaction, (iii) the working procedures of the Project Management Team and (iv) the expected deliverables from the Project Management Team.

[38] PPP Policy, page 16
[39] Article 14(1) of the PPP Proclamation

3.3.2.1 Membership of the Project Management Team

The legal framework[40] outlines four broad categories of members for the Project Management Team:

1. A Team Leader from the Contracting Authority who will be the main focal point for leading the work of the Project Management Team. The Contracting Authority should appoint a high ranking civil servant from within its personnel, possible the most senior ranking civil servant (who may delegate this responsibility to another named senior official). The Team Leader, is empowered by the PPP Directive[41], to take project development decisions on behalf of the Contracting Authority
2. Other members from within the Contracting Authority with the requisite financial, technical and legal skills it considers necessary to handle the management and implementation of the PPP project.
3. A representative from the PPPDG
4. If required, other representatives with the financial, technical and legal skills necessary in order to properly supervise the transaction advisor

The Contracting Authority shall chair the Project Management Team

3.3.2.2 Roles and Responsibilities of the Project Management Team

The Project Management Team's Terms of Reference will elaborate on the entire life cycle of responsibilities of the Project Management Team from PPP project preparation to contract management, as outlined in the legal framework[42] (further elaborated in Section 2.1). The responsibility of each team member, for each stage of the PPP project life cycle should be articulated and written down in the Terms of Reference.

The table below highlights the responsibilities of the Project Management Team, as outlined in the legal framework, along with indicative assignments for members.

Project Management Team Tasks as per legal framework	Team members indicative assignments
a. Prepare and appraise the Public Private Partnership project to ensure its, legal, regulatory, social, financial, economic and commercial viability;	Legal, regulatory, social, financial and economic specialists within team to ensure that scope of work defined for feasibility advisors to address PPP project structure requirements. Team leader to review and approve final scope of work
b. Prepare the project in accordance with guidelines and standard documents issued by the PPPDG	Team Leader to appoint Secretary for the Project Management Team. Secretary responsible for ensuring Project Management Team processes are consistent with guidelines and directives issued by PPPDG.
c. Ensure that the Private Party comply with the provisions of this Proclamation;	Team Leader to provide support to PPPDG in overseeing PPP procurement process

[40] Article 9 of the PPP Directive
[41] Article 9(1)c
[42] Article 14(2) of the PPP Proclamation

Project Management Team Tasks as per legal framework	Team members indicative assignments
d. Monitor the implementation of the Public Private Partnership Agreement entered into with the Contracting Authority;	Team Leader with support from his team to develop Project Management Checklist for monitoring Private Party performance<br>Monitoring will be required after commercial close, during construction and then operations, requiring different expertise on the Project Management Team.
e. Liaise with all key stakeholders during the project cycle	Team Leader with support from his team to ensure feasibility advisor's scope of work includes extensive stakeholder consultations
f. Oversee the management of a project in accordance with the Project Agreements entered into by the Contracting Authority	Legal specialist to ensure all parties to PPP agreement are duly executing obligations
g. Submit to the PPP Directorate General, annual or such other period reports requested by the PPP Directorate General on the Project Agreements entered into by the Contracting Authority	Team Leader with support from his team to appoint Project Records Manager to be responsible for developing and maintaining library of Project reports and agreements for sharing with PPPDG
h. Maintain a record of all documentation and agreements entered into by the Contracting Authority relating to the Public Private Partnership project	Project Records Manager to be responsible for developing and maintaining library of Project reports and agreements for sharing with relevant stakeholders
i. Ensure that the transfer of assets at the expiry or early termination of a Public Private Partnership Agreement is consistent with the terms and conditions of such agreement, where this agreement involves a transfer of assets	Team Leader and Technical Expert (engineer) to participate in pre-handover and pre-handback inspections.
j. Carry out such other functions as may be assigned to it by the Contracting Authority	Team leader to assign roles to members as required

3.3.2.3 Working Procedure of the Project Management Team

Following the setup of the Project Management Team, the Team Leader will supervise the team in preparing a work plan and operational procedures for the Project Management Team. The Team Leader may nominate the Project Management Team Secretary to lead the preparation of the operational procedures. The procedures will cover:

- Schedule and frequency of meetings for the Project Management Team meetings;
- Task assignments and responsibilities for each member, including if required specific sub-teams or committees for specific tasks (e.g. specific sub-Team with expertise for Contract Monitoring);
- Structure for each meeting and protocols for managing meetings (e.g. seniority in leading meetings in the absence of the Team Leader or his/her delegate, process for reviewing reports and adopting decisions, communication mechanisms with internal and external stakeholders);

- Framework for managing advisors; and
- Protocols for communication with the PPPDG and Contracting Authority

BEST PRACTICE

Establishing the Project Management Team

Arguably the largest single constraint to the successful and timely implementation of PPPs is the ability of Governments to make clear and prompt decisions to approve specific PPP structures and decisions.

In many countries, PPPs can take years to be implemented due to delays in decision-making, reviews, and approvals by Government bodies and committees – which often have limited understanding of the requirements of PPPs or who are asked to approve complex, limited guarantee mechanisms for PPPs for the very first time. Therefore, when establishing a new PPP Project Management Team, the Contracting Authority should pay particularly close attention to providing it with clear authority and resources to be able to either make clear and timely PPP recommendations to the Contracting Authority to act on, or to make clear and timely decisions itself on behalf of the Contracting Authority.

Composition of the Project Management Team

The Project Management Team should include the following representatives:

- An engineer familiar with engineering activities among Public Entities responsible for the sector where the Public Private Partnership is being implemented;
- A lawyer familiar with the enabling legislation for the Contracting Authority and the legal environment in the sector where the Public Private Partnership is being implemented;
- An economist familiar with demand and supply for public infrastructure and services in the sector where the Public Private Partnership is being implemented;
- A social and environmental safeguards specialist familiar regulations and best practices in the sector where the Public Private Partnership is being implemented,
- A representative from the PPPDG; and
- The most senior civil servant in the parent Ministry responsible for the sector where the Public Private Partnership is being implemented.
- The Project Management Team should be chaired by the senior civil servant, or a designate.

3.3.2.4 Expected Deliverables

The Project Management Team's Terms of Reference will outline the expected deliverables from the PMT. These deliverables will include:

- Participation in the preparation of the feasibility study
- Participation in the preparation of tender and contractual documents

- Preparation of a Project Management Checklist
- Preparation of a Project Close-out Report
- Preparation of an annual or such other period report regarding project implementation
- Record of all documentation and agreements entered into by the Contracting Authority relating to the Public Private Partnership project

Estimated Time Required: For the first pilot PPP projects that a Contracting Authority prepares, it may take up to 4 weeks to establish the Project Management Team. For subsequent PPP projects by Contracting Authorities already experienced with PPP transactions, it may take 2-3 weeks to complete this procedure.

3.3.3 ALLOCATION OF FUNDS FOR THE DETAILED STUDY OF THE PPP PROJECT

As part of the preliminary approval, the PPPDG will confirm from the Ministry of Finance that sufficient funds will be made available to support the detailed analysis of the PPP project. The funding required from the government to finance the studies and advisory services for detailed analysis (i.e. the feasibility and ultimately the PPP transaction) shall be estimated by the Contracting Authority and have been provided as part of the PPP candidate project review.

Following the preliminary approval, the Contracting Authority would need to (as part of its budgeting process) allocate funding for the cost of Transaction Advisors. Since PPPs are complex projects, there is a need for skilled professionals particularly at the early stage of development to assist the Contracting Authority and the PPPDG in the process. Transaction Advisors[43] are consultants hired (i) to conduct the full feasibility study, (ii) to prepare the tender documents and the draft PPP Agreement, (iii) to support the PPPDG during the procurement phase. This budgeting process shall be the responsibility of the unit that prepares annual budgeting within the Contracting Authority. This budget would then be appropriated as part of the federal budgeting and appropriation process by the legislature.

The Contracting Authority needs to ensure funding is appropriate and available for engaging feasibility study Transaction Advisors prior to proceeding with the tender procedure. Once the funding is available, the implementation of **Phase II** can begin.

3.3.4 PROCUREMENT OF THE TRANSACTION ADVISOR

When the Contracting Authority is allocated funding to procure advisors for the in-depth study of the PPP project, feasibility advisors can be engaged. The legal framework[44] mandates the PPPDG to provide Technical Assistance to the Contracting Authority in procuring for a Transaction Advisor to carry out the Feasibility Study. The PPPDG shall exercise this obligation by retaining, in conjunction with the Contracting Authority, an Advisor to carry out the Feasibility Study.

[43] Articles 7(1), 9(1), 10(3) and 10(4) of the PPP Directive allow for a Transaction Advisor to be hired by the Contracting Authority and PPPDG

[44] Article 7 (1-a) of the PPP Directive

The legal framework[45] specifies that the PPPDG has an obligation to ensure the Transaction Advisor reports both to the Contracting Authority and the PPPDG.

The Contracting Authority and the PPPDG have the option to hire (i) one advisor to carry out both the feasibility study/tender and contract document preparation and the transaction advisory at the procurement phase, or (ii) two separate advisors, one to carry out the project feasibility study and another one to handle the procurement phase. The choice of hiring one or two advisors depends on the size, scale and complexity of the project. If a project is more complex and requires different specific sets of skills to carry out the feasibility study and the transaction advisory, two separate advisors will be hired. Under this approach, the Contracting Authority will engage an advisor for the feasibility study once the project has been approved to be develop as a PPP by the Board. The PPPDG will then hire a transaction advisor for the procurement phase. In the case of two advisors, there will need to be an interface mechanism between the parties to ensure smooth transition from one to the other once the project has been approved by the PPP Board to proceed to tender?.

For less complex projects that do not require specialized and different skills for the feasibility study and the transaction advisory, a single advisor carries out the in-depth feasibility analysis on behalf of the Contracting Authority, and if the feasibility conclusion is a recommendation to proceed with a PPP transaction, then the advisor supports the PPPDG in procuring the PPP transaction. As relevant, different firms (engineering, financial, technical, management consulting firms, etc.) will team-up together in consortia to provide all of these services.

The relationship among the various technical advisors and their counterparts in the Government side should evolve as the Government's experts become more knowledgeable and experienced. Indeed, as they develop experience and expertise, there should be less reliance on outside advisors.

[45] Article 7 (1-e) of the PPP Directive

Figure 3-5: Separate Advisor vs. Single Transaction Advisor

Two Advisors: Separate Advisors for Feasibility and Procurement Stages	One Transaction Advisor
**ADVANTAGES**	**ADVANTAGES**
Separate advisory allows the recruitment of specialised firms for feasibility and transaction advisory	Avoids second procurement process for Transaction Advisor following feasibility approval
Feasibility advisor not conflicted on transaction recommendations	Allows speedy procurement of PPP following feasibility approval
	Transaction Advisor incentivised to develop implementable PPP structure
	May lead to lower overall transaction costs; potential cost and time savings (synergy)
**DISADVANTAGES**	**DISADVANTAGES**
Would need to launch second procurement for Transaction Advisor following feasibility approval	Smaller pool of advisors with both feasibility and transaction advisory experience
Feasibility results may be stale (changed circumstances) due to lengthy procurements	Potential conflict of interest on transaction recommendation
Potential conflict in methodology/approch between Advisors	Advisor more likely to be conflicted and recommending transaction proceeding under less than robust conditions

3.3.4.1 Preparation of Terms of Reference for Project Preparation

A. TERMS OF REFERENCE FOR TRANSACTION ADVISORS

This is the procedure to be followed by Contracting Authority in preparing the scope of work for Transaction Advisors that will support the procurement of the feasibility and PPP transaction.

Based on the size, sector, and complexity of the project the Contracting Authority will first decide to hire individual or a firm as a transaction advisor.

The Contracting Authority will then prepare a Terms of Reference for the Transaction Advisors, describing the work, deliverable reports, and outputs expected for the feasibility report and the tender documents. The PPP legal framework[46] establishes nine critical expectations from the feasibility study:

1. Project capital and operating costs
2. Proposed responsibilities and risks assumed by the Contracting Authority and the Private Party
3. Proposed government participation and support, if any

[46] Article 17(2) and article 17(3) of the PPP Proclamation

4. The technical and legal requirements of the project
5. The social, economic and environmental impact of the project
6. The sustainability of the project for both parties
7. Value for Money of the project compared to other procurement methods
8. Affordability of the project by the Contracting Authority and/or end-users
9. The institutional capacity of the Contracting Authority to prepare, tender, implement and monitor the project

These nine requirements inform the requirements from the advisor, and therefore the make-up of the Transaction Advisor's team.

A sample Terms of Reference for a Transaction Advisor is presented in **Appendix B (Part 4:– Indicative TOR for the External Advisor)**

Estimated Time Required: The amount of time required to complete this procedure depends on the size, the nature and complexity of the given PPP project. Preparing of the Terms of Reference for PPP Consultants should require between 2-3 full days to complete. However, the tendering, selection, and contracting of external advisors may require 2-3 months or more to complete, depending on the number of qualified consulting firms & teams that have already been short-listed.

List of Documents Provided in Appendix B - Phase 1 PPP Project Identification & Suitability Analysis
1. Part 1: PPP Suitability Application (Preliminary Screening Form and Prioritization)<br>***2. Part 2: PPP Pre-feasibility Checklist***<br>***3. Part 3: PPP Project Pipeline Form***<br>***4. Part 4: Indicative Terms of Reference (ToR) for the External Advisor(s)***

4 PHASE II: PPP PROJECT APPRAISAL AND PREPARATION (FEASIBILITY STUDY)

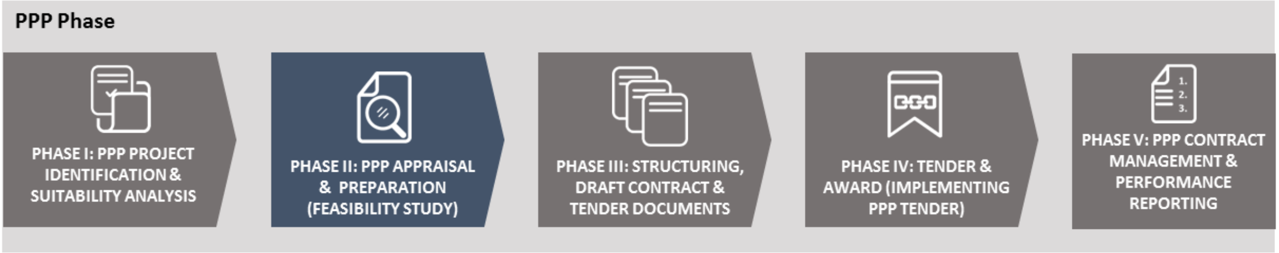

The purpose of the project appraisal and preparation phase is to determine if the PPP approach is the most economically advantageous to the Contracting Authority (*Article 17 of PPP Proclamation*). In the PPP framework, this economic advantage is demonstrated when a project is analysed and determined to be ***technically and financially viable*** and results in ***Value for Money*** for the Government of Ethiopia.

A well-developed feasibility study will not only illustrate the economic advantage of the project to the Contracting Authority, but it will also indicate of the project will present an attractive investment proposition to the Private Party.

KEY CONCEPTS AND DEFINITIONS

'Project Viability'

A Public Service Activity is deemed to be ***technical viable*** if its proposed plan of implementation is deliverable with current available technology, skills and resources.

The Public Service Activity is deemed to be ***financial viable*** if proceeds from its operations are sufficient to cover operating costs sustainably and return of capital.

The Public Service Activity is deemed to be ***economically viable*** if its implementation generates net positive impacts on the society and the environment and mitigates the wasteful use of resources.

This section summarizes the processes and tasks involved in developing a potential PPP to a stage ready for procurement. A promising PPP candidate project is subjected to a full and detailed analysis of its feasibility as a PPP, the proposed structure is designed and draft tender documents including a draft of the contract detailing the proposed risk-allocation structure are prepared.

Figure 4-1: Overview of Tasks under Phase II

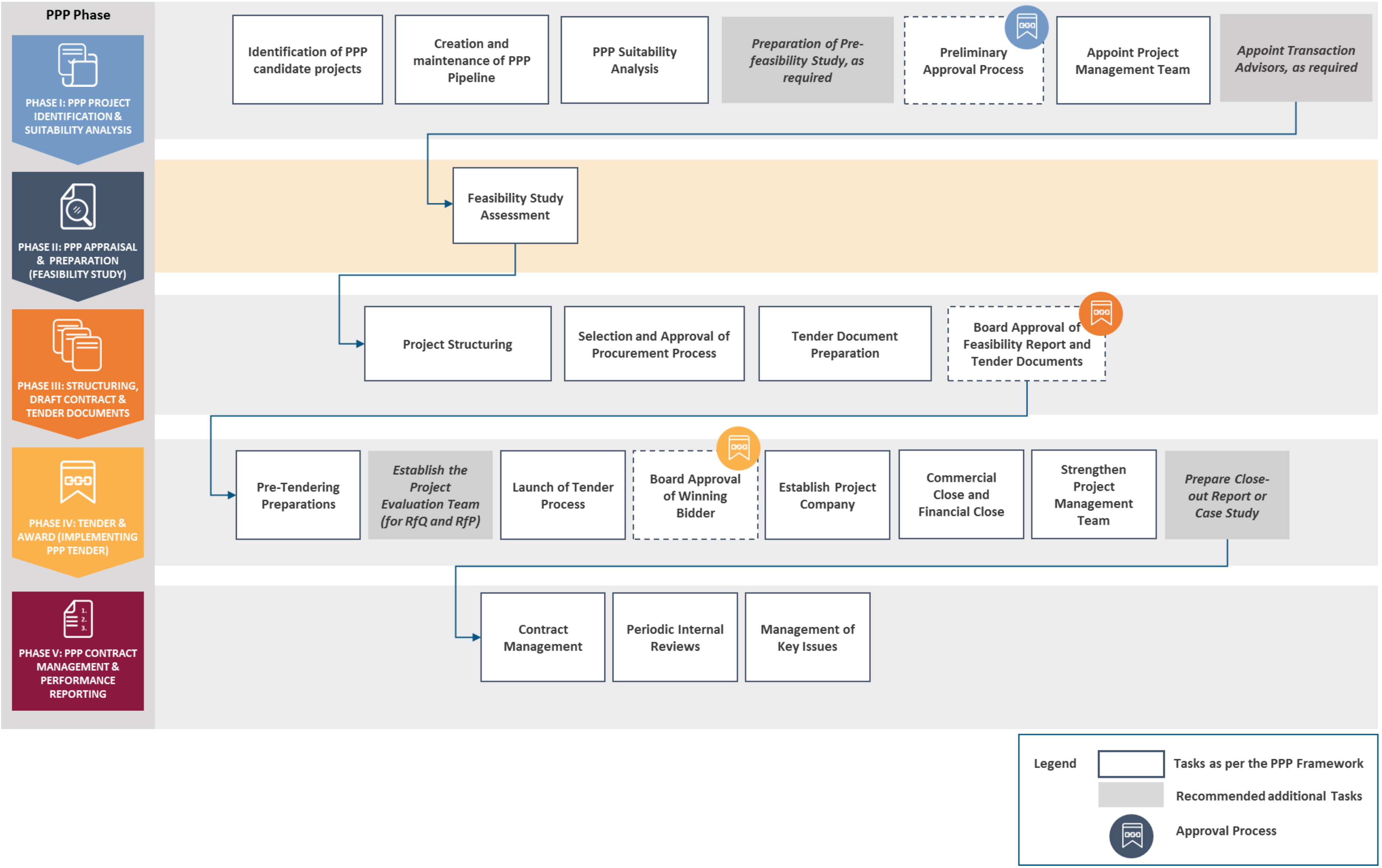

Figure 4-2: Phase II Process and Key Stakeholders

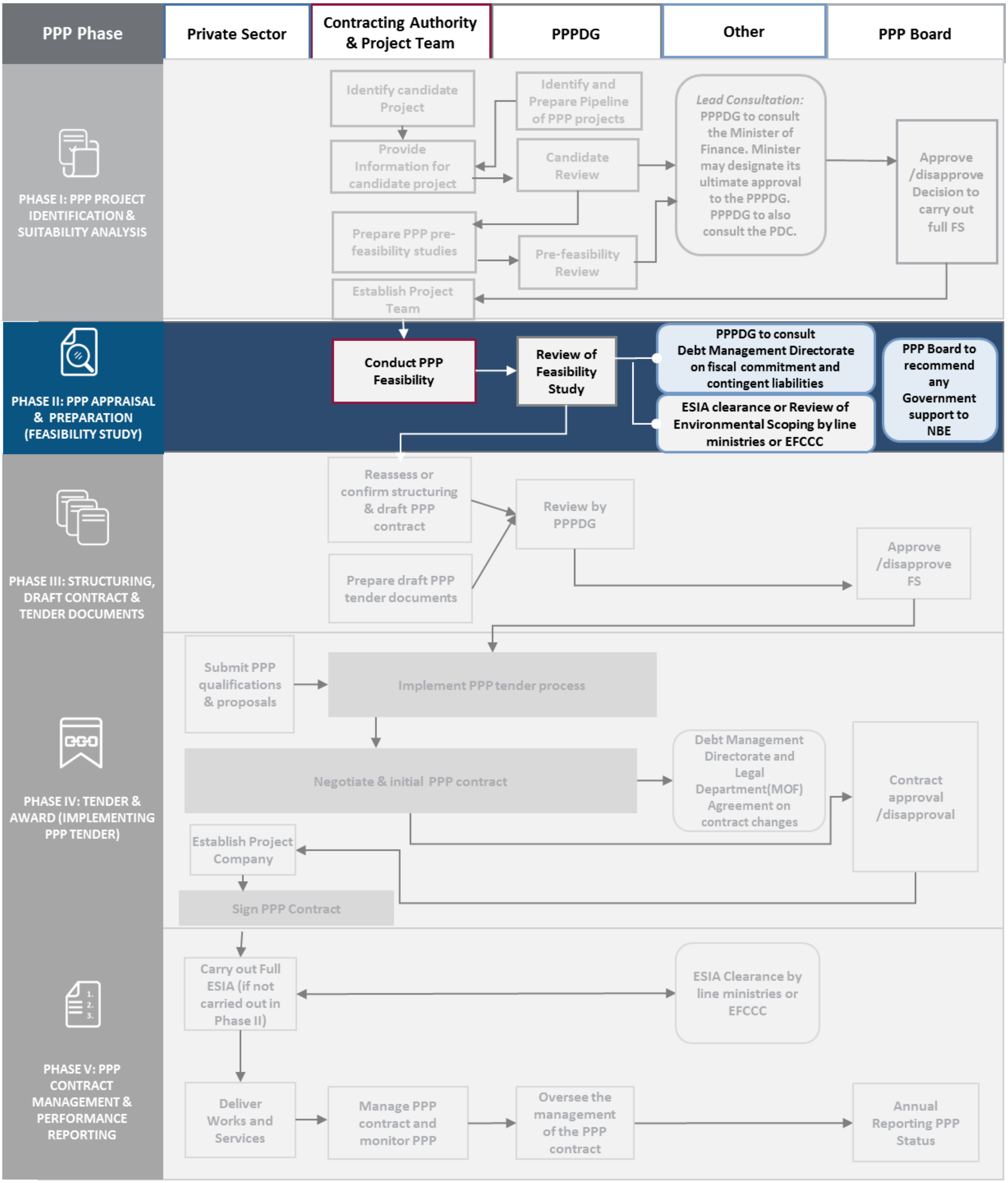

4.1 FEASIBILITY STUDY AND OUTCOMES

This feasibility study will involve a rigorous process of identifying and gathering complete qualitative and quantitative information about the proposed PPP project. This information must be synthesized and analyzed to determine if the proposed PPP project is technically, economically and financial feasible and generates Value for Money for the Contracting Authority.

The legal framework is not explicit on a process for preparing the feasibility study, but mandates the PPPDG to prescribe guidelines for this activity. The legal framework, however, is precise on the expected outcomes for the feasibility study, outlined in the Terms of Reference for the feasibility advisors in Section 3.3.4.1. This task is led by the Contracting Authority with the support of its PMT.

Very generally, there are a series of steps in preparing a PPP Feasibility study, listed below.

Table 4-1: Procedures for Developing Feasibility Study

1	Establish the Project Management Team ***(End of Phase I)***
2	Contracting Authority to engage feasibility advisor ***(End of Phase I)***
3	Project history background
4	Developing service output levels for PPP project
5	Designing stakeholders consultation plan
6	Project affordability analysis
7	Project demand analysis
8	Project technical feasibility analysis
9	Project financial feasibility analysis
10	Project economic feasibility analysis
11	Legal and institutional feasibility analysis
12	Project environmental impact assessment
13	Project risk identification
14	Project risk analysis and mitigation
15	Recommended PPP risk allocation structure
16	PPP Value for Money analysis/benefits
17	Market Testing
18	Designing and drafting Draft PPP Agreement
19	Procurement Plan

4.1.1 PROJECT HISTORY BACKGROUND

This introductory section of the feasibility study will briefly describe the project and its history background.

The project description will mainly cover the following points:

- Project Description
- Scope of Project
- Justification of Project
- Contracting Authority

As part of the project's history background this section will discuss if the project is aligned with the country's national strategy mainly the GTP or a sector Master Plan. If previous studies have been carried out on the project, their main conclusions will also be highlighted here.

4.1.2 DEVELOPING THE SERVICE OUTCOME LEVELS FOR THE PPP PROJECT

4.1.2.1 Definition

The Project Management Team is responsible for preparing the PPP project specifications and the service outcomes.

In contrast to traditional public sector procurement, which is input-based (e.g. a Public Entity providing the design specifications for an EPC Contractor to construct an office building on a turnkey basis), PPP projects require that Contracting Authorities specify the project outcomes for delivery by the Private Partner. The Private Partner then has to determine the most economical means to deliver those outcomes. Examples of PPP services outcomes are presented include:

- Number of passengers per day for urban transit systems; the technology for an urban transit system; the corridor for an urban transit system;
- MW capacity of proposed new power plant; technology for the power plant;
- Number of households to be provided a water and sewerage service; capacity of water and waste treatment plant;
- Length (km) or capacity (kg, metric tonne, cubic meter) of infrastructure to be developed

These specifications and outcomes will be informed by the Contracting Authority's requirements or legal obligations for the delivery of Public Service Activity. Proposed outcomes must be clear, measurable, and also provide a solid foundation for the viability analysis, preparation and risk-structuring of the Public Service Activity as a PPP.

4.1.2.2 Implementation Process

1. Refer to **Appendix C (Part I: Background References - Phase II – Outcomes from the PPP Feasibility Study)** for some key questions that should be asked by the Project Management Team as part of the PPP feasibility study to enable the determination of service outcomes for PPP projects. For many project outcomes, the analysis to be carried out by advisors would span more than one specialisation (e.g. demand, technical and legal). The general process for defining service level outcomes is as follows: Identify the major technical components, and associated outputs, for the PPP project. Technical components of many typical PPP projects would include, among others:

    a. Serviced facilities/core project facilities

    b. Any alterations required to existing facilities

    c. Buildings, installations and asset maintenance requirements of the project

    d. Energy management services

2. In each of the technical components identified in (1) above, draft outputs standards for the overall performance of the project.

    a. Specify the **geographic scope** or describe the area of the demand that the project must serve. For example, this output specification could require that project serve the needs for a single Contracting Authority, for an entire municipality, or for other multi-jurisdictional area.

    b. Specify the number of **users or the volume demand** for services that the project would need to meet. For example, for most roadway PPPs, output levels of service require the maximum daily number of vehicles or the minimum traffic speeds that the new road project must be able to provide

    c. Estimate the **minimum time requirements** the project must meet. For example, a PPP project might require that the service provider must deliver its services to end users within a specific number of minutes, of hours, or of days from the official submission of requests

    d. What is the minimum level of **operational reliability** that the PPP project should function? For example, for some key public services, it is not uncommon to require that PPPs provide 99% levels of guaranteed reliability

    e. **Technical Quality Standards**. Each sector of infrastructure and Public Service Activity will feature its own sector-specific measures of quality of service. Industry standards for each sector should be researched, assessed and identified.

    f. Clear **Maintenance Standards**, provide a guide to the required standards for the PPP project's facilities and equipment maintenance frequency and level.

    g. **Appearance Standards.** Specifying clear and measurable standards for the appearance of a public facility and its cleanliness is difficult to prescribe in practice. In general, such issues can be addressed through requiring standards such as, "ensure that the facility is clean enough, when in use, to provide a safe, hygienic, environment and a positive image."

4.1.2.3 Outputs from the Process

The outputs from this process is a clearer and refined scope for the PPP contract. In some projects, this refining of the scope of the PPP contract may result in different PPP models being used to develop, manage and operate a Public Service Activity.

The main output from this procedure is the **PPP Project Outcomes Standards Report**.

4.1.3 DESIGNING THE STAKEHOLDERS CONSULTATION PLAN

This is the process for the Project Management Team to communicate activities about the PPP project in an open and transparent manner by including the important participation of key stakeholders from the end user, labour, private sector, public sector, Non-Governmental, and other relevant sectors. This activity should be carried out in close coordination with the PPPDG and in particular, based on the disclosure policy adopted by the PPPDG.

The Ethiopian PPP legal framework provides for periodic announcements, approved by the PPP Board, communicating information about the implementation of PPP projects.

The purpose of designing and implementing a stakeholders' consultation plan is to determine if there are significant public concerns, issues, or even opposition to a potential project that must be effectively resolved before the project can be considered viable as a PPP. International experience has shown that PPP projects have the best chance of being sustainable when they can incorporate the important views of end-users and other affected stakeholder groups and to effectively address or resolve them within the project's structure.

4.1.3.1 Implementation Process

1. Identify the relevant stakeholder groups that would likely be affected by and interested in this project. It is also useful at this point to estimate each group's probable concerns about and interests in the proposed PPP project. An indicative list of stakeholder groups and their likely PPP concerns and interests is illustrated below:

Table 4-2: PPP Concerns and Interests by Stakeholder Group

	Stakeholder Group name	Probable Interest in PPP Project	Probable Concern in PPP Project
1	End Users & Consumer Associations	Interest in being provided with improved, more accessible and reliable levels of service	Concern about tariffs and prices increasing or becoming unaffordable
2	Commercial Users & Commercial Consumer Associations	Interest in being provided improved, more accessible and reliable levels of service	Concerns about tariffs and prices increasing
3	Industrial/ High-Volume Users	Interest in being provided improved, more reliable levels of service	Concerns about tariffs & prices increasing
4	Land-owners	Interest in seeing the value of adjoining lands increase	Concerns about being forced to sell land and relocate
5	Current providers of services within the sector	Interest in possible new business opportunities with the new PPP, such as providing services or supplies to the new PPP company	Concerns about no longer being a viable, competitive service provider due to new PPP

2. Design a PPP Stakeholder Consultation Plan: Based upon the completion of the above matrix, design a plan for communicating with and consulting each of relevant stakeholder groups:

   a. Identify leaders or representatives of each relevant stakeholder group.

   b. Identify relevant reports and summaries about the project that should be developed to provide relevant information on the project status to the stakeholders on key issues.

   c. Identify relevant stakeholder consultation mechanisms

3. Develop a PPP Project Stakeholder Consultation Plan, for each Phase of the PPP project cycle, how and when each stakeholder group will be consulted on key options for structuring the project and on its current status. An indicative project management

scheduling template provided below, can be used as the basis for identifying the consultation mechanism planned for each stakeholder group during the relevant phases of the project.

	Feasibility Study and Tender Documents	PPP Tender Implementation	PPP Contract Management and Monitoring
Timeline (illustrative)	1 – 3 months	4 – 10 months	11 – 260 months
Stakeholder Group 1			
Stakeholder Group 2			
Stakeholder Group 3			

4. Develop a Stakeholders' Feedback Tracker Report. For each phase of the PPP project, this report will list and categorize comments from stakeholders and indicate how their feedback were incorporated in the preparation of the project.

4.1.4 PROJECT AFFORDABILITY ANALYSIS

Affordability analysis is the procedure for determining the maximum amount that users of the Public Service Activity (a public Contracting Authority, end-users, or a combination of both), can afford to pay for the PPP project's services over the entire life of the contract. Three kinds of analyses are involved:

1. Fiscal Affordability Analysis refers to the analysis to determine if the public Contracting Authority can afford a PPP project

2. End User Affordability Analysis examines Public Service Activity consumers' ability to afford PPP services

3. A Hybrid Affordability Analysis examines if a PPP project is affordable based on a combination of support and payments by a Contracting Authority and End Users.

In all instances, the affordability analysis procedure is completed by setting an "affordability limit" that service users (Contracting Authority, end users or both) can be expected to pay, and PPP prices or tariffs that are above this are considered unaffordable and therefore cannot be approved by the PPP Committee unless subsidies are incorporated.

4.1.4.1 Fiscal Affordability Analysis

The Contracting Authority's cost in developing a PPP project, includes investments in fixed infrastructure or moveable assets, recurring payments (such as annual management contract fees, annuities, etc.) and any contingent obligations that are part of the public sector's liabilities for PPP. For a PPP project to be affordable for the Contracting Authority, the present and future values of these costs must, in simple terms, be capable of being accommodated in the Contracting Authority's budget.

In contrast to traditional public sector procurement, where the entire cost of the Public Service Activity is allocated to the Contracting Authority current budget, on a PPP project, payments

are deferred over time and the cost must be reflected in multi-year budgets and they require a clear mechanism for reporting by the Contracting Authority.

The PPP Proclamation and PPP Directives require the PPPDG to consult with the Ministry of Finance at the Project Identification Phase to ensure that sufficient budgetary provision has been made to implement a Public Service Activity as a PPP. At the Project Identification Phase, the requirement is ensuring that sufficient budget is allocated to fund the feasibility study and advisors, if needed.

Part of the output of the Feasibility Study, will be the commitments required of the Government of Ethiopia (including the Contracting Authority and other agencies such as Ministry of Finance, if guarantees are required). The PPP Directives requires the PPPDG, in coordination with the Ministry of Finance and the National Bank of Ethiopia, to outline these financial firm and contingent commitments and identify any potential constraints in the Government of Ethiopia's budget to funding these commitments over the PPP project horizon.

For each PPP project, a "Fiscal Affordability Assessment" should be prepared by the relevant authority. This would be in two stages: (i) prior to entering procurement as part of the feasibility and structuring process, and (ii) prior to signing the PPP contract. The Fiscal Affordability Assessment evaluates the expected budgetary requirements of direct and contingent public sector liabilities over the duration of the proposed PPP contract. Two sample forms have been included for use in consultation with the MOF Debt Management Directorate and to obtain agreement regarding fiscal affordability in **Appendix C – Part 3 and Appendix E- Part 4.**

The expected fiscal impact during the PPP contract will be modelled as part of the feasibility financial analysis, to assess its affordability to the government. This includes determining the financial flows that the government will be expected to receive from the private party (concession fees), and/or is required to pay to the private party (availability payments, Viability Gap Funding, subsidies, etc.). It also includes examining "contingent liabilities" that may occur under stressed scenarios, such as higher or lower growth rate of demand, the occurrence of risk events, delayed implementation of the project, etc.

Contingent liabilities are payment obligations whose timing and amount are contingent on the occurrence of a particular discrete/uncertain future event or series of future events. Examples include loan guarantees, and minimum revenue guarantees in PPP contracts. May also refer to 'implicit contingent liabilities' where there is no formal obligation but likely pressure for government to provide fiscal support e.g. following a disaster.

Budgeting for contingent liabilities is a challenge, as the need for, timing, and amount of payments are often not known until the liability is realized and the financial model will include various scenarios and sensitivities.

Type	Description	Example
**Direct Liabilities (always explicit)**	▪ Predictable government obligations that are certain and quantifiable<br>▪ Typically fixed contractual payments to be made by the government to a private party	▪ Availability payments<br>▪ Milestone payments<br>▪ Output based payments<br>▪ Viability gap payments
**Contingent Liabilities (can be explicit or implicit)**	▪ Unpredictable government obligations that may be incurred when an uncertain risk event occurs	▪ Government guarantees on risk variables including inflation risk or exchange rate risk<br>▪ Government guarantees on demand<br>▪ Force Majeure<br>▪ Termination payments<br>▪ Credit guarantees<br>▪ Compensation payments by the government if the PPP project defaults

Source: Caribbean PPP toolkit 2017 International Bank for Reconstruction and Development / The World Bank

The outcome of this modelling exercise is a projection of the impact of the PPP project on government liabilities, non-financial assets, net lending/borrowing and the resulting cash balance to government. The project impacts can then be compared to national forecasts of the same fiscal variables to evaluate the fiscal affordability of the project. This assessment must be done not only for the PPP project under consideration, but for all existing PPP projects (or the fiscal impact of existing PPP projects must be included in the forecasts of the national fiscal variables).

There are tools available to help with this analysis and reference is made to the PPP Fiscal Risk Assessment Model (PFRAM) developed by IMF and the World Bank.

PPP Fiscal Risk Assessment Model (P-FRAM)

The P-FRAM is an analytical tool developed by the IMF and the World Bank to assess the potential fiscal costs and risks arising from Public Private Partnerships (PPP) projects. In many countries, investment projects have been procured as PPPs not for efficiency reasons, but to circumvent budget constraints and postpone recording the fiscal costs of providing infrastructure services. Hence, some governments ended up procuring projects that either could not be funded within their budgetary envelope, or that exposed public finances to excessive fiscal risks. To address these concerns, P-FRAM was developed as an analytical tool to quantify the macro-fiscal implications of PPP projects. It is designed to be used mostly by PPP units in ministries of finance. P-FRAM provides a structured process for gathering information for a PPP project in a simple, user-friendly, Excel-based platform, following a four steps decision-tree:

Who initiates the project? The impact of main fiscal indicators (i.e., deficit and debt) varies depending on the public entity ultimately responsible for the project (e.g. central, local governments, state-owned enterprises, etc.).

Who controls the asset? Simple standardized questions assist the user in making an informed decision about the government’s ability to control the PPP-related asset—either through ownership, beneficial entitlement, or otherwise.

Who ultimately pays for the asset? The funding structure of the project is what determines its implication on main fiscal aggregates. P-FRAM allows for three funding alternatives: (i) the government pays for the asset using public funds; (b) the government allows the private sector to collect fees directly from users of the asset (e.g., tolls); and (c) a combination of the two.

Does the government provide additional support to the private partner? Governments can not only fund PPP projects directly but they can also support to the private partner in a variety of ways, including providing guarantees, equity injections, or tax amnesties.

Once project-specific and macroeconomic data are introduced, P-FRAM automatically generates standardized outcomes. The latter include: (i) project cash flows; (ii) fiscal tables and charts both on a cash and accrual basis.

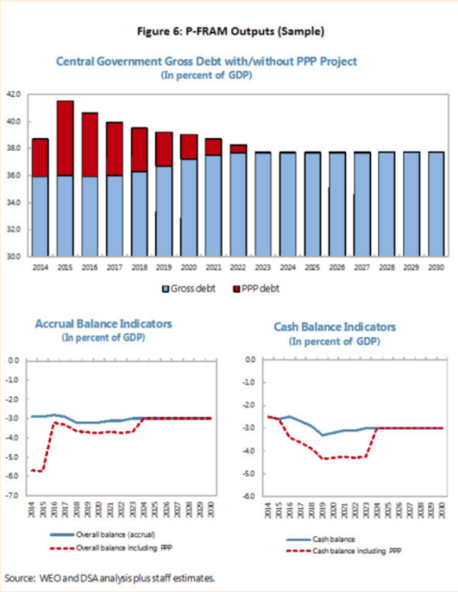

Source: https://www.imf.org/external/np/fad/publicinvestment/

4.1.4.2 End User Affordability Analysis

The end user affordability analysis takes the form of a Willingness-to-Pay (WTP) or Ability-to-Pay (ATP) survey carried out as part of the Project Demand Analysis.

The methodology for ATP involves identifying the expenditure profile of the project end users of the Public Service Activity, and ascertaining how much those users spend on the Public Service Activity. That benchmark is then compared to the PPP prices or tariffs of the Public Service Activity. If the proposed PPP prices or tariffs are higher than current expenditures and make up a large share of end user's income, then prospective end users may not have the Ability-to-Pay.

WTP involves utilizing stated preference survey techniques determine how much prospective end users of a Public Service Activity are willing to pay for that service. As with most surveys, there is a tendency for respondents' bias (in this case, understating their WTP). Therefore, the ATP survey is a natural complement to the WTP survey to illustrate the revealed preferences of prospective end users.

4.1.4.3 Hybrid Affordability Analysis

For PPP projects where the Contracting Authority and end users contribute to PPP tariffs or prices, the affordability analysis will be a combination of the methodologies for fiscal and end user affordability. The fiscal affordability analysis is to be carried out in any scenario since there will always be contingent liabilities.

4.1.4.4 Overview

The affordability limit is later compared to what end users will have to pay for the Public Service Activity if provision was delivered by a Public Body. This analysis is part of the analysis called the Public Sector Comparator and is examined in greater detail in the Value for Money analysis.

One of the requirements of the feasibility study outcome under the Ethiopian PPP legal framework is to determine the sustainability of the PPP. This procedure ensures that a proposed PPP scheme can be funded by sustained payments from service consumers (i.e. the Contracting Authority, end-users, or both) over the entire term of the PPP contract. This requires analyzing Contracting Authority's medium term budget plans, under its budget framework; or analyzing end-users ability to pay for services. Without this procedure, a Contracting Authority risks entering into PPP contracts (especially ones that offer the attraction of not requiring the Government to borrow funds to build infrastructure) which are not affordable over the medium to long term. In such instances, the Ministry of Finance would have to step-in and assume the burden of making PPP payments – at the expense of other competing needs for key public services; or else the Contracting Authority may be forced to terminate a PPP contract it can no longer afford and be required to bear considerable close-out and takeover costs.

4.1.4.5 Implementation Process (Contracting Authority Inputs)

4.1.4.5.1 Fiscal Affordability

The emphasis during this assessment is to determine if sufficient budgetary allocation has been made for (i) Public sector project costs or liabilities (ii) Project contingent liabilities

1. Check if a budget has already been established for the specific PPP Project by the Contracting Authority or by the Government's budget office. This may be broken up between:

    a. Project preparation and development (fees for transaction advisors)

    b. A capital budget set for the design, construction and equipment costs for the project.

    c. An operating budget for the project's operating and maintenance costs.

2. If these budget figures are available, summarize these capital and operating budget limits over the project years.

3. It is important to compare any budgets with the estimate of the project's whole-life capital and operating costs determined during the PPP financial and technical feasibility assessment. This would include potential contingent liabilities.

4. If the available estimates of the project's costs are above the budget limit that has been set by the Contracting Authority, then either the PPP project's scope and output specifications may need to be reduced (to make the project less costly), or the project may need to be referred to the MOF budget office for a new, higher budget. Clearly all parties involved do not want to spend valuable resources developing a PPP project that is beyond the ability of the Contracting Authority, Government, or its end-users', capacity to pay.

4.1.4.5.2 End User Affordability Analysis Procedures

The emphasis during these procedures is to establish end users' ability and willingness to pay PPP tariffs or prices for the Public Service Activity.

1. Design an instrument to survey prospective end users of the Public Service Activity. The instrument will solicit end users usage habits (frequency, payments) and experience around the Public Service Activity.

2. The survey will also collect socio-economic information, such as age, gender, employment status, incomes, and expenditure habits. The survey can be complemented with information from the national statistics office to validate socio-economic and other profile information

3. The survey will present a description of the proposed PPP project and request from respondent how much they would be willing to pay for the service as a PPP

4. The survey results will be analysed to present, at the minimum, the following for prospective end users; (i) Average and median income (ii) Share of income spent on basic goods and service (iii) Share of income spent on Public Service Activity of prospective (iv) WTP for the Public Service Activity (v) ATP for the Public Service Activity

5. Prospective end users WTP and ATP will be compared to the PPP prices or tariffs to determine if the proposed PPP project is affordable for end users

4.1.4.6 Outputs

The main outputs from this analysis is the **PPP Project Affordability Report**[47] which provides the MOF Debt Management Directorate with sufficient information about the impacts of the PPP project (outline above) to enable them to assess whether or not the project is an affordable proposition for the government over the duration of the complete project. The MOF Debt Management Directorate will be responsible for assessing and monitoring the impact of PPPs on the governments overall budgetary framework (at both individual project and PPP portfolio levels). The MOF Debt Management Directorate will assess the long-term fiscal risks and impact of the PPP Project, whether direct or contingent, explicit or implicit, and determine whether they are acceptable and affordable given other ongoing Government priorities.

Figure 4-3: Example of content of the PPP Project Affordability Report (detailed by year of the PPP Project)

Revenue	
**PPP fees**	Concession fee - Upfront
	Concession fee – fixed annual
	Concession fee - Variable
	Revenue sharing
	Other
	Concession fee - Upfront
**Total**	
**Expenditure**	
**Government commitments:**	
**Capital**	Investment grants
	Repayments/refunds
	Other
**Total**	
**Annual payments:**	Availability payments
	PSO/subsidy payments
	Other
**Total**	
**Contingent Liabilities**	Guarantees – specific risk
	Guarantees - other
	Termination - Government default
	Termination - Private Party default
	Other
**Total**	

4.1.5 PROJECT DEMAND ANALYSIS

Demand analysis is the procedure that forecasts the volume of service that the Contracting Authority or end users will need from the project over its life. This demand forecasts gives a very important indication of how large the project must be in terms of its design capacity, to adequately meet projected demand. This demand forecast will also indicate whether, when, and what size additional expansions to project's capacity should be made during the life of the project in order to adequately meet future demand.

Demand forecasting for PPP projects are needed to confirm what size or design capacity of the project should have in order to be economically sustainable. Moreover, because this demand forecast determines the size of the project that is needed, it is critically important in determining

[47] This is the report prepared from the feasibility study examination of the project. A further report will be made prior to contract signature if changes during the tender and award process significantly alter the affordability profile of the project.

the subsequent PPP procedures' estimations of project costs (financial costs, economic costs, environmental impacts, and potential size of any public sector supports for a project).

4.1.5.1 Implementation Process

Infrastructure demand forecasting is usually based upon two different strategies:

- Regression analysis for a countrywide, region, or municipal area; and
- Case-study method for a portion of an administrative jurisdiction

Regression analysis models for projecting demand levels link past demand growth rates for the given service (as the dependent variable) to factors such as past GDP growth rates, population rates, etc. (as independent variables). This enables planners to make new projections of future demand for the given service, based upon other, well-established and generally-available projections of medium-to-long term population growth rates, or short-to-medium term projections of GDP growth.

For projects requiring regression analysis:

a. Gather the population or GDP data projections. GDP data and projections from the Ministry of Public Administration's Statistical Office of Country or from the relevant infrastructure line ministry.

b. When using simple linear regression analysis, the formula for projecting demand is often written based upon the general model:

$$Y_{t+1} = BS_t * (1 + GDP_{t+1})$$

Where
- "Y" is the level of demand forecasted
- "BS" is the baseline for the variable being forecast
- "GDP" is the assumed growth rate.

c. Calculate the standard deviation and the degree of correlation between these variables.

d. Identify specific risks to demand levels and projection: A defining characteristic of infrastructure investments, compared to other commercial and industrial sectors of the economy, is their relatively stable levels of demand. While other commercial sectors often experience wide boom and bust cycles, the demand for services like roads, public transport, solid waste management, water and even energy remains more or less constant. However, infrastructure projects that serve a single, large end-user or off-taker often face a higher level of demand risk. Demand forecasting shall therefore include estimates of the magnitude and probability of a single large customer suddenly reducing its demand for the project.

Case study method is generally used for projects whose demand cannot be projected linearly. For example, in practice often a significant portion of the demand for new roads transportation facilities are not demand driven but instead "supply driven." The road is built first, and then the population growth and demand for the road is "induced" to come later on. A portion of the demand for the new project would come from simply diverting current travelers in the transport corridor from the existing routes to use this new road, and some of this demand could be

estimated through regression analysis. However, an important portion of its future demand will come from new passengers attracted into the corridor due to the new additional economic benefits (time savings and better access to economic activity) that the new project offers. This portion of demand requires the case method, and data would need to be gathered, for example, on projected growth rates in new business and housing that would be assumed to locate along within the current transport corridor.

This procedure's report on the project's demand will clearly address the following:

	Demand Analysis Questions	Project Management Team's Answer
1	What general method is being used to estimate the demand for the PPP project's services? (Regression, Case Method, or Other). Describe and explain why?	
2	Present the projected level of demand using to at least 3 general scenarios:<br>▪ Base Case<br>▪ Optimistic Case (higher than planned demand)<br>▪ Pessimistic Case (lower than planned demand)<br>Describe the assumptions used for each scenario and briefly explain the reasons for selecting the assumptions of each scenario	
3	List and describe any relevant and significant specific risks to demand for the project, such as single-industry risks, etc.	
4	At this initial stage, who would likely be able to best manage the project's demand risks - the Government or the private service provider? Briefly explain why.	

4.1.5.2 Outputs

The main outputs from these tasks shall include the following:

1. Submit a PPP Project Demand Analysis Report using the above matrix as a general outline.
2. It is important for public authorities to understand how the output of this procedure is a critical input for several of the subsequent PPP Feasibility Study procedures, which will ultimately determine the size, the possible structure, and the viability of the whole project as a PPP. This forecast of demand determines:
    a. The size of the project and therefore the capital costs of its construction and start-up
    b. The size of the operating & maintenance, and periodic renewal costs needed to sustain the project over its entire economic life
    c. The size of the economic and social benefits the project can provide to the local economy
    d. The size of any public sector supports or risk-sharing in the PPP project by the Government in order to realize these net economic benefits from the project

e. Whether or not the specific project's demand risks can be best managed by the private service provider, by the Government, or should be shared between the two.

4.1.6 PROJECT TECHNICAL FEASIBILITY ANALYSIS

A PPP project's technical feasibility analysis identifies the key functional components of a project, the applicability of existing technologies to meet the planned output specifications, as well as the size and probability of key risks to the technical performance of the project and its component parts. PPP contracts need to be output-based and to assign the risks of selecting the project's inputs (like selecting the technology, design, materials, etc.) to the private partner. However, this procedure does not seek to prescribe what technical inputs the private partner must use. Rather this procedure confirms that a viable technical solution to the project does exist, it analyzes key risks to the technical performance of the project and, provides inputs for subsequent procedures that estimate the project's capital and operating costs.

This procedure is needed to clarify that a feasible technical solution does exist to meeting the established demand requirements over the entire life of the project. This procedure is also needed to identify the key technical components of the project, which may need to be provided by other parties. For example, a new bus terminal project may require that the Contracting Authority fund and construct new approach roads, new water and electricity interconnections, etc. It is also needed to allow a meaningful estimate of the cost to construct, operate, maintain is likely going to cost to construct, operate, maintain, and periodically renew and rehabilitate the appropriate technical solution to the project. These costs ultimately determine if the project is financially viable and whether it may need Government support and forms of public sector risk-sharing to be deemed feasible as a PPP.

Figure 4-4: Range of options of Government Support

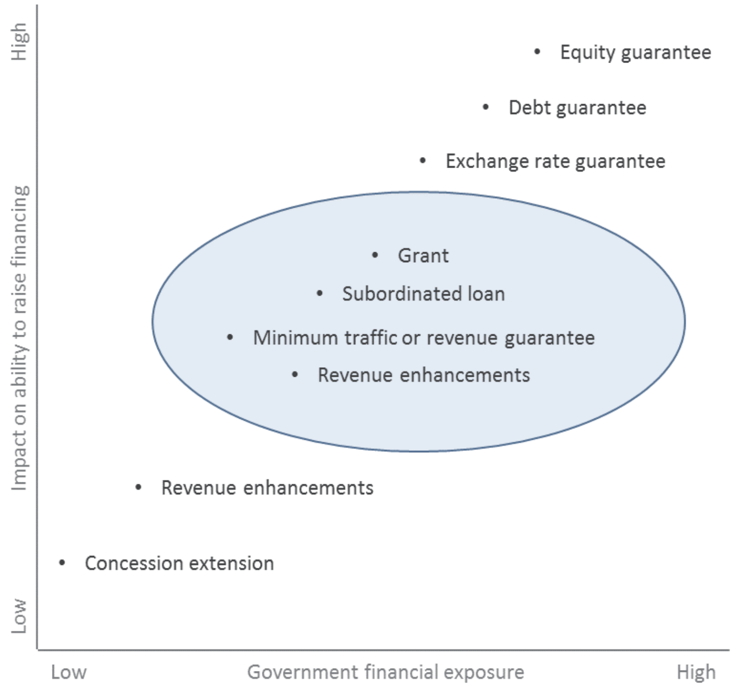

Source: Private Financing of Toll Roads Gregory Fisher & Suman Babbar RMC Discussion Paper Series 117

Technical feasibility investigations have been conducted by experienced engineers in Public Entities in governments around the world for many years. The experience and expertise to supervise advisors can be found with Public Entities and should be introduced into this procedure.

4.1.6.1 Implementation Procedure

For each relevant technical solution for the project, identify and describe the main technical components required, estimate the capital and operating costs for the overall technical solution (or for each component if available), including the need for any additional renewal of these components during the overall life of the project.

Describe and analyze any key technical risks facing each component of the project. Lastly, estimate the total time likely required for the construction, completion, and commissioning of the given technical solution. The matrix below can be used to organize and present the output of this task.

	Technical Component Description	Estimated Cost	Asset Life	Technical Risks for this project
1	Land acquisition			Location's appropriateness
2	Site preparation and civil works			▪ Antiquities<br>▪ Environmental Remediation/ clean-up requirements<br>▪ Sub-soil testing
3	Provision of existing public assets & facilities			▪ Unknown condition of existing assets or non-performance
4	New Building & Facility Design & Construction (by components)			▪ Non-performance of the design & technology
5	Equipment Supply & Installation			▪ Non-performance of the equipment/ technology
6	Ancillary Facilities (App roach Roads, parking facilities, on-site housing, etc.)			▪ Risk of completing ancillary facilities on time
7	Interconnections (electricity, water, communications, other bulk supplies & disposals)			▪ Risk of completing connections on time
8	Storage Facilities (reservoirs, fuel storage, chemical storage, etc.)			▪ Risk of inadequate supply of inputs
9	Input/Fuel Supplies (fuel, raw water, bulk wastewater, etc.)			▪ Risk of insufficient supply of fuel inputs
10	Transmission & distribution assets (bulk water mains, electricity transmission lines & distribution pipes, etc.)			▪ Risk that transmission and distribution assets may be inadequate to meet output requirements
11	Safety & Security Facilities			▪ Risks to the security and safety of project's assets
12	Waste disposal facilities & systems			
13	Operation & maintenance systems			
14	Other technical components required			
15	Total Estimated Costs for Technical Solution			

	Technical Component Description	Estimated Cost	Asset Life	Technical Risks for this project
16	Total Estimated Construction Period and Economic Life for the Technical Solution			

4.1.6.2 Outputs

This technical feasibility analysis may conclude that a given technology should not be considered for the project due to problems of safety, incompatibility with existing technologies in the sector, lack of proven track-record of successful performance, unacceptable environmental impacts, etc. If this is the case, the explanation shall be clearly stated as a conclusion of this procedure and shall also be shared with potential bidders during the tender process.

4.1.7 PROJECT FINANCIAL FEASIBILITY ANALYSIS

Financial feasibility analysis for a PPP estimates the total costs of the project and then identifies the likely range of tariffs, fees or per-unit prices that will be required to recover all of these costs for the project to be financially viable as a PPP. These cost estimates must include a realistic, expected return on the Private Party' equity. Financial feasibility analysis differs from economic feasibility analysis, which estimates and includes the benefits and costs of the project for the economy (i.e. reduced traffic congestion, shorter commuting times, reduced pollution levels, etc.).

Financial feasibility analysis is required to estimate the size of the end-user tariffs, tolls, fare or per-unit prices that the Private Party would need to charge to recover the full costs of the project. These include operating and maintenance costs, regular debt service payment, and adequate returns on the investor's capital. This analysis is also needed to indicate opportunities for any changes in PPP prices over the life of the project. For example, a number of long-term BOT contracts plan for reductions in tariffs or payments after year 15 when the underlying long-term debt would be paid off. Without this analysis, public authorities often proceeding to tender and expecting an unrealistically low prices from bidders. This analysis will also indicate when a project is not financially viable at a given per-unit price. Therefore, the Contracting Authority may need to consider either changing the scope of the proposed project or offering some additional public supports and credit enhancements.

4.1.7.1 Implementation Procedures

1. Designing the PPP Financial Feasibility model to be easily understood, as well as to respond to the numerous "what if" questions, the basic design of PPP financial feasibility spreadsheet models shall have three general components:

    a. Inputs of data

    b. Manipulations and displays of data

    c. Analytical results

Figure 4-5: Indicative steps involved in designing a PPP Financial Feasibility model

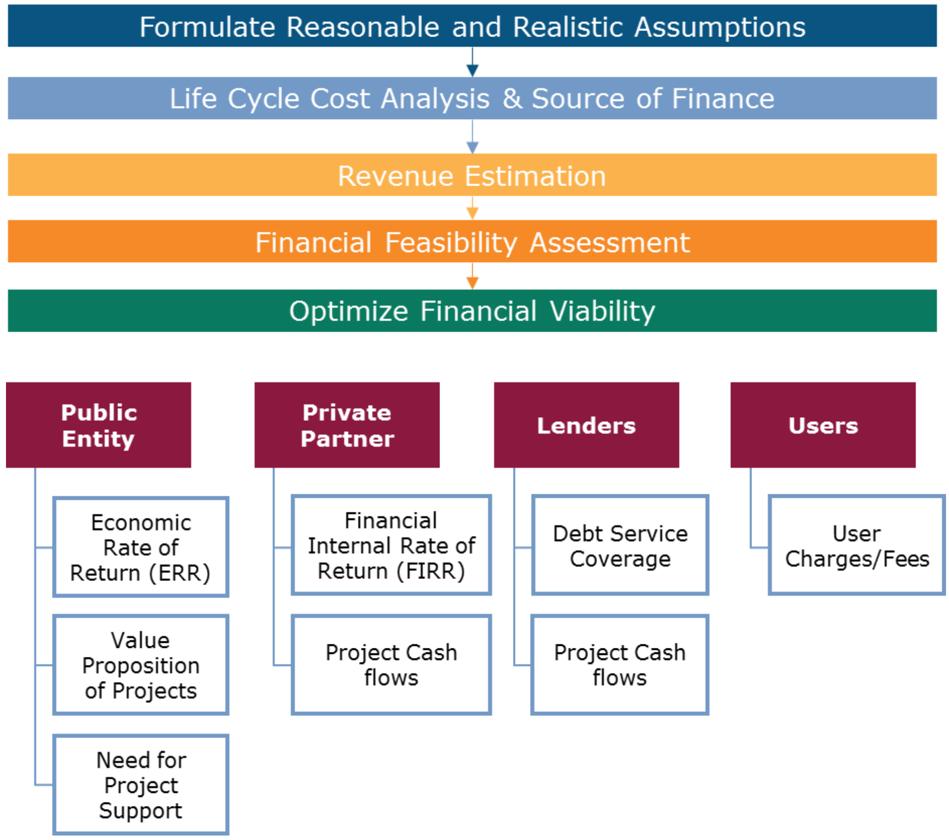

Source: https://www.pppinindia.gov.in/documents/20181/33749/PPP+Guide+for+Practitioners/

While each individual PPP project is unique, all PPP financings have a few components in common. These components include:

a. Key Inputs and Results: When designing any model is advisable to place the key input variable cells as well as to display the key analytical results. This makes it much easier to understand the sensitivity of the project's key results to given changes in the inputs.

b. Capital Expenditures (Capex): This includes lists of construction costs, equipment purchases, working capital, testing and commissioning, and other start-up costs.

c. Financing Structure: This shows how the total capital expenditure and start-up costs will be met, by equity, by senior debt, by subordinated debts, public sector grants and other sources. Either within this component, or as a sub-component, debt repayment schedules shall be shown for each loan.

d. Income Statement: This shall show the project's revenues, operating costs, depreciation, and finally its net income. For projects whose revenues are subject to varying levels of demand, a separate sub-component dealing with demand levels may be needed.

e. Cash Flow Analysis: This converts the accounting results of the income statement into cash flows. The key results from this component include the investor's return on equity as well as the key coverage ratios important to lenders.

f. Balance Sheet: This tracks the total assets and liabilities of the project company through the life of the project.

In practice almost all analysts are asked to present their models and to demonstrate to the other stakeholders involved in the project how the model was designed, how it functions, and to interpret the meaning of the key results.

2. Listing the Key Input Data to Gather. The next step is to list out the key data that will needed as inputs for the model. This will serve two important functions:

   a. It is a guide to key input cells that need to be included in each of the model's components

   b. It is a guide for interviewing and requesting data from technical specialists, engineers, operators, and other consultants or stakeholders working on the project

   Some common checklists of data to gather for inputs in project finance cash flow models include:

   a. Project Technical Data

      i. Minimum required performance standards of the new project/facility, expressed in terms of outputs

      ii. Background data on demand and growth projections from existing official master plans for the sector (20-30 years). Identify classes of users (i.e. retail, institutional, etc.)

      iii. Background data on existing network: size and age of existing facilities & assets

      iv. Proposed or possible location for the facility

   b. Capital and Operating Cost Data

      i. Preliminary Capital Expenditure estimates of project construction and start-up. Sub-categories of these costs could include:

         - Any Civil works or major preparations

      ii. Installation, broken down by sub-project and project phase

         - Equipment, broken down by category
         - Interconnection costs
         - Other capital expenditures
         - Periodic rehabilitation and renewal costs

      For cash flow models at the Feasibility Analysis Phase, these costs are almost always estimates based upon construction cost estimating

guidelines and tables. More detailed data on these has to wait until the proposal or design stage of the project. If possible, modellers should try to include the formulas behind these estimates in the model so that any proposed changes in the size or capacity of the project can more easily show reasonable estimates of changes in capital expenditures.

iii. Operating & maintenance cost estimates for major cost categories including:

- Wages
- Overheads
- Utilities, electricity, water, communications & other services
- Any consumables required (fuel, chemicals, lubricants, etc.)
- Other operating & maintenance costs

iv. Because O&M cost data at the Feasibility Analysis Phase is based upon estimating guidelines, such as percentage of plant size, etc. it is important to try to include these formulas, when available in the model.

v. Depreciation schedules for assets

c. Financing Data

i. Debt

- List potential commercial lending sources
- Estimates of Debt/Equity leverage acceptable
- Lending periods available
- Currencies and estimated ranges of interest rates and spreads
- Terms of any grace periods available
- Terms for any subordinated debt available
- Minimum coverage ratios required: interest & debt service

ii. Equity

- Estimates of Debt/Equity leverage acceptable
- Estimates of expected return on equity by likely investors

The recommended outputs that an effective PPP financial model will produce include:

	PPP Financial Model Outputs	Explanatory Notes/Comments	Description of How these Outputs Apply to this Proposed PPP Project
1	Overall cash flow	This will show not just accounting measures of revenues, expenses, and net income, but show actual cash flow, including cash flow available to pay debt. Revenue projections are not a certainty and depend on many future variables.	
2	Cash flow available to equity participants	This is net cash flow, available after cash expenses, debt service, and taxes	
3	Profitability/ Viability	The Financial Internal Rate of Return (FIRR) for the whole project, and the Internal Rate of Return on the equity investment or "ROE"	
4	Cost Recovery (expressed as a Payback Period)	The number of years to pay back the equity investment. The norm is 5-7 for commercial investments, but for long-term infrastructure investments, 10-15 years can be common	
5	Debt Service Coverage Ratio (DSCR)	The cash flow available to repay debt each year (after operating cash expenses have been paid) should be at least 1.5x – 1.3x the debt payment that is due that year. The minimum DSCR varies from sector-to-sector, but often lenders look for an initial minimum of 1.5x for projects facing demand risks, which can be reduced if the project's creditworthiness is strengthened.	
6	Estimated Financial Net Present Value (FNPV)	As a project's investment costs occur during the first 2-3 years and then its profits/returns occur later on in the future (years 4-20) is it difficult to compare them over time. The FNPV uses a common "discounting" technique to express all costs and returns in today's monetary terms, which allows an investor to determine whether total returns are greater than total costs, and by how much.	

The key results of the PPP financial feasibility analysis model that will be produced include:

	PPP Financial Model Outputs	Explanatory Notes/Comments	Description of How these Outputs Apply to this Proposed PPP Project
1	Length of the PPP contract	The length of a PPP contract is usually determined by the length of the useful life of the new assets created by the PPP + approximately 20 to 25%. Debt raised by the PPP should have a term roughly equal to the useful life of its key assets. Good practices recommend having an additional contract term margin, or cushion, of 20-25% in case the PPP ever needs to be financial restructured and the debt terms extended by a sufficient amount.	
2	Financial impact of different types of debt and equity	This looks at whether there may be a role in the project for subordinated debt, or secondary equity.	
3	Optimum debt & equity ratios	For most large PPPs the D/E is around 70/30, (depending on the sector). Higher risk sectors & projects generally require more equity (30-40+ %), while lower risk PPPs need less equity (15-25%). The lower the equity, the	

	PPP Financial Model Outputs	Explanatory Notes/Comments	Description of How these Outputs Apply to this Proposed PPP Project
		lower the PPP's cost of financing, up to a point, as high leverage raises credit risks.	
4	Losses in early years (if applicable) that need to be met by PPP service provider (and/or by Government Support)	A new project facing market demand risk, like a toll road may need many months (or even years) before traffic levels grow to the point where they cover the project's full O&M and debt service costs. In the meantime the project still needs to pay its O&M expenses and also to make its required, regular principal + interest payments to lenders.	
5	Government support that may be needed	If the PPP financial model indicates that based upon current tariff levels, the project would not be able to recover all of its costs then additional public sector supports or risk-sharing mechanisms may be required. The PPP financial model can usually provide estimates for the levels of public sector supports that would be needed for the project to be financially viable. Such options could include the public sector contributing additional equity to an institutional PPP or providing a guaranteed minimum level of demand for a PPP, especially during the project's early years. These new options will require updating and reconfirming the PPP's affordability analysis procedure.	
6	Corporate tax revenue to GOE (when profits are made)	As financially viable projects, PPPs should generate positive net income and therefore to have to pay corporate taxes on that income. The financial model should accurately show the taxes the project will need to pay, to make sure that tariffs are set to recover these costs, and to be able to calculate the net level of Govt. support needed. For example, if a project requires $10 million of public support at the start of the project, but later on it pays $10 million of taxes (on a net present value basis) during the life of the contract. The net impact of the Govt. support for this PPP would be zero.	
7	Impact of changing key variables such as tariffs, project costs, etc.	This result requires conducting sensitivity analyses on the project's key variables. What analysts want to know is, "If one input variable such as the tariff level (or revenues, construction costs, op. costs, interest rates, forex rates, etc.) falls by 10%, by how much do the key output results of the project (ROE, DSCR, Payback Period, etc.) change?" When a project is no longer financially viable due to a relatively small change in an input variable, we say that the project is very sensitive to that variable. This may indicate an area where Government support may need to help share the risk.	

When analyzing the outputs of a PPP financial model the results listed and described in the matrix above will be areas the Project Management Team will focus. Financial analysts responsible for implementing this procedure shall provide analyses and descriptions of these specific results for the proposed PPP project whose feasibility is being analyzed.

4.1.7.2 Outputs

The main outputs from these tasks shall include the following:

- A PPP Project Financial Feasibility Analysis Report covering:
    - Description of the design of the financial feasibility model
- Explanation for the sources of key input data that has been gathered, such as estimates of construction costs, operating costs, lending terms, tenors, and coverage ratios, expected returns on equity, etc.
- Descriptions of the need for any public sector supports required by the project.

4.1.8 PROJECT ECONOMIC FEASIBILITY ANALYSIS

A PPP project's economic costs include both the direct and tangible, cash-based costs as well as the indirect and intangible costs, such as costs for mitigating negative environmental impacts, resettlement costs, etc. The project's economic benefits, which are often a major source of justifying a long-term infrastructure project's potentially substantial investment requirements, will include direct as well as indirect benefits such as reduced congestion on roads, improved public health levels, improved education levels, etc. As economic feasibility analysis makes adjustments to a project's financial feasibility analysis, to carry out this activity, it is essential that the project financial costs be determined. These financial costs are then converted to economic costs using an appropriate conversion factor, such as the Social Discount Rate used to put a present value on costs and benefits that will occur at a later date. Financial costs may not reflect an appropriate cost for society. In this instance, it is necessary to make some adjustments to evaluate the economic cost using a conversion factor. In many cases, for simplicity, the procedure for the Project Economic Feasibility Analysis will be carried out after the PPP Project Financial Feasibility Analysis has been completed.

Infrastructure projects, particularly in the social and environmental sectors, which tend to have negative financial returns, may justified on their net economic benefits.

The net economic benefit is obtained by calculating the Net Present Value (NPV) of the expected benefits less the economic costs using an appropriate discount rate. The discount rate typically used by development partners such as the World Bank or the African Development Bank is 12% reflecting the high economic growth potential of developing countries such as Ethiopia. In other words, if the project does not generate an economic return of more than 12%, the project would not be deemed to add sufficient value to the economic growth potential.

Such projects often require large financial costs to develop and construct, but they can only collect a relatively small amount in cash-based fees from end users. Such PPPs will only be financially viable if there is support from the Government in terms of important availability payments. However, the Government should only make these payments if the project can deliver its planned economic benefits such as a better-educated populace, improved public health, a safer environment, reduced time lost to congestion on roads, etc. For such projects, it is important for the government to carry out an Economic Feasibility Analysis to determine if the project has a positive economic NPV and an economic rate of return higher than the social cost of capital. Therefore, economic feasibility analysis is critically important in determining whether a proposed PPP project warrants public support and risk-sharing, such as availability payments, so that it becomes "bankable," or not.

4.1.8.1 Implementation Procedures

Identify the economic cost items of the proposed PPP projects. Most of these will be similar with the financial project cost items, except the project taxes and government subsidies (if any), which will be excluded from the economic cost items:

1. Calculate the value of project's economic costs by multiplying the economic cost items - which have been selected from financial cost items - with relevant conversion factors appropriate for Ethiopia. This economic cost is the actual cost that will be borne by Country's economy as-a-whole, excluding government interventions and market distortions.

Identify the net economic benefits of the proposed PPP project. Unlike economic costs, the economic benefits for infrastructure projects are quite different with the financial benefits. For example, a new road development could generate economic benefits in the form of journey time savings, fuel savings and reduced car maintenance requirements. Such benefits can be quantified and a monetary value applied to them to enable the measurement of the economic benefit.

The procedure involves quantifying and assessing the monetary value of the individual economic benefits of the proposed PPP project. For example, the journey time savings that result from the construction of a new road is reported in minutes. Academic study and research, both nationally and internationally, have established the monetary value per unit of time for different road commuters (e.g. drivers, car passengers, drivers in the urban core, drivers in suburbs, rail passengers, cyclists, etc.). A hypothetical estimation of the journey time savings from the new road constructed is illustrated below.

1. Annual journey time savings (in minutes) for all passengers utilizing a new road is estimated over the PPP project horizon. The total minutes per trip saved per annum is identified.

2. The value of time (per category of traveler) and per unit of time (Ethiopian birr per minute/hour) is determined from literature or academic research carried out in Ethiopia.

3. In the absence of academic literature, a proxy involving the hourly rate of wage labour (Realized Income method) as a measure of the value of time. Using this method, the annual income of road users is determined and divided by the hours of work. The resulting wage per hour/minute can be used as a proxy for the value of their time.

4. The total annual time savings is then multiplied by *the per minute* value of time to determine the total annual economic benefit in time saving.

The methodology can be extended to other economic benefit factors such as fuel savings (cost of litres of fuel saved by using the new road), reduced car maintenance costs (avoided maintenance cost due to less km travelled, etc.), employment income from new jobs caused by the project.

The graphic below presents an illustrative analysis of the net economic benefit of an infrastructure project.

			Year 1	Year 2	Year 3	Year 4	Year 5	Year 6	Year 7	Year 8	Year 9	Year 10
**Economic CAPEX**												
Roadway		USD 000	-5,398	0	0	0	0	0	0	0	0	0
Tolling and communication		USD 000	0	-2,586	0	0	0	0	0	0	0	0
**Economic OPEX**												
Infrastructure maintenance		USD 000	0	-352	-346	-339	-332	-326	-178	-175	-171	-168
Staffing		USD 000	0	-271	-266	-260	-255	-250	-245	-241	-236	-231
**Economic Benefits**												
Journey Time Savings		USD 000	0	1,054	1,354	1,653	1,953	2,102	2,102	2,102	2,102	2,102
Employment Benefits		USD 000	0	432	432	432	432	432	432	432	432	432
Increase in Rental Income		USD 000	0	0	0	0	0	0	0	0	0	0
Increase in Land Value		USD 000	0	565	565	565	565	565	565	565	565	565
**Net Benefits**			**-5,398**	**-1,159**	**1,739**	**2,051**	**2,362**	**2,523**	**2,675**	**2,684**	**2,692**	**2,700**
SDR	12%											
**NPV**	**£8,236.50**											
**IRR**	**28.15%**											

Since many of the parameters used in the Economic Analysis are based on assumptions, a sensitivity analysis will be carried out to test the robustness of the Economic Analysis. For example, the assumptions to be modified may be the cost projections and overruns, income generated, the discount rate used in NPV calculations.

4.1.8.2 Outputs

The outputs of this procedure are as follows:

1. Prepare a PPP Project Economic Feasibility Analysis Report, which establishes a likely range of required economic costs, net economic benefits, and Economic Internal Rates of Return for the PPP Project.

4.1.9 LEGAL AND INSTITUTIONAL FEASIBILITY ANALYSIS

This procedure assesses the existing laws and regulations that are relevant to the specific project and determines whether or not these are adequate to make the project viable. This procedure also analyzes whether the existing public sector institutions have the capacity and resources to enforce these laws and regulations and to sustain all of the public sector’s roles and obligations throughout the term of the PPP contract.

While the PPP framework may clarify the overall legality of PPP contracts for the provision of Public Activity Services, there may be specific legal and regulatory uncertainties within a proposed project that could still lead to costly delays or even cancellations. Additionally, nearly all PPP contracts require Contracting Authorities to fulfill new institutional functions which they may have never performed previously, such as monitoring the performance being delivered by the project and ensuring contractual compliance.

4.1.9.1 Implementation Procedure

1. Legal and Regulatory Feasibility Analysis. This task consists of a systematic review of all existing, relevant laws and regulations within the given infrastructure sector in question. The legal specialist(s), tasked with carrying-out this review and with experience in the structuring and regulation of PPP projects, shall be familiar with both the Public Service Activity sector in question and with the consequences of risks and risk allocation options for PPPs. This review could include:

	Legal and Regulatory Feasibility Question	Explanatory Notes/Comments	Description of How these Outputs Apply to this Proposed PPP Project
1	Do the existing laws and regulations clearly allow a private contractor or PPP approach to be used in offering the proposed public service?	In many cases existing laws do not explicitly allow for Government agencies to "transfer" this right to provide public services to outside private contractors, but they also do not explicitly prohibit it.	
2	Are there Ministerial regulations or implementing rules in the sector which prescribe additional specific steps that must be followed in a PPP?	Some sector regulations may require their own specific processes that must be followed in areas of PPPs like land acquisition, resettlement, labour impacts, tendering, stakeholder consultation, etc.	
3	Do laws allow for private companies to actually collect payments or fees directly from end users?	Some sector laws may only allow a public or statutory corporation to actually collect end user fees.	
4	Do existing laws allow a private service provider to legally own the infrastructure asset it either constructs or manages? Can a private service provider borrow against (i.e. mortgage) project assets?	Some sector laws may not allow private ownership of the underlying infrastructure assets. In such cases the Government may retain legal title or "ownership" rights but instead transfer specific "Usufructuary[48]" or operating rights to the service provider.	
5	Are more than one Government entity required to award the PPP or to contribute assets needed?	Some PPPs require both national and local Government assets, such as land, buildings, & other assets be provided to the project and are delayed by legal uncertainty over existing asset ownership assets.	
6	Are the legal procedures for acquiring the land needed for the project clear and manageable?		
7	What are all of the permits, licenses and approvals that the project needs from different Government bodies? How long will it take to obtain each one?		
8	Would a PPP contract be clearly enforceable? What factors might affect the ability to enforce the contract by either the Government or service provider?		
9	Are there key risks of legal liability in the proposed PPP?	In sectors like roads, the legal liability from traffic accidents that may occur on the road can be considerable. In many cases, such legal liability for accidents are better managed by the Government than by the service provider.	
10	Can the private service provider obtain legal access to all the inputs and outputs required by the project?	In some cases, such as waste disposal, laws require that ownership of actual wastes, and any products produced from	

[48] One having the legal right of using and enjoying the fruits or profits of something belonging to another.

	Legal and Regulatory Feasibility Question	Explanatory Notes/Comments	Description of How these Outputs Apply to this Proposed PPP Project
		waste is legally Government property. Such cases may require legal amendments.	
11	Will the PPP likely require partial guarantees from the Government, such as ensuring the timely availability payments from a non-creditworthy Public Entity? What is the process for obtaining & approving such guarantees, and how long would it require?	If a financial performance guarantee or "comfort letter" is required for the PPP, the process for preparing, approving, and providing such a guarantee shall be confirmed.	

2. Institutionally, does the public sector have the resources and the skills needed to oversee and to monitor the performance of the PPP contractor throughout the life of the contract? This includes the public sector having the resources to enforce compliance with the terms of the contract by end users, by other Government agencies, and especially by the private contractor.

    a. Plans for establishing within the client Contracting Authority a Contract Management Team.

    b. Are new laws, decrees, or regulations needed to establish and create a budget for a new organization or to give it legal authority to enforce the PPP contract?

    c. Will the Contracting Authority have the technical skills, resources (e.g. information technology systems, monitoring equipment) and capacity to carry-out these monitoring functions (technological skills, financial skills, legal skills, quality-of-service monitoring skills, etc.)?

3. An important element of contract monitoring is deciding the appropriate regulatory framework for contract monitoring. Broadly speaking, many jurisdictions regulate PPP contracts using a (i) Regulation by Contract approach, (ii) Regulation by Commission approach, or (iii) A hybrid of the two

4. The table below illustrates the two broad approaches.

Table 4-3: PPP Contract Management & Regulatory Issues

Regulatory Issues	Regulation by Contract	Regulation by Commission
**Market structure**	Monopsony (single public sector buyer of PPP services)	Monopoly (single private provider and many individual users)
**Risk of inaccurate information about existing system network and assets**	Relatively low	High risk of unknowns
**Private party's certainty on new investments required**	Higher degree of certainty	Lower degree of certainty
**Collection risk faced by private party**	Low (collection from single public client whose payment can be guaranteed by Ministry of Finance	Higher (collection from thousands of individual retail users)

Regulatory Issues	Regulation by Contract	Regulation by Commission
**Price adjustment procedures**	Determined by specific formula within the contract. Little discretion give, such as to a regulator, to make tariff adjustments	Prescribed by price adjustment procedures within the contract, but usually verified and approved by the discretionary judgment of a qualified commission of regulators

5. The PPPDG must accurately diagnose, for each Public Service Activity, the nature of the market, the risk allocation, mechanisms for price adjustments as some of the factors when deciding which regulatory approach to adopt.

6. For example, if the market exhibits characteristics of a monopsony, then regulation by contract is appropriate.

7. In practice however, many jurisdictions adopt several layers and regimes of regulations on PPP services. This is because PPPs are not one-dimensional, i.e. a Public Service Activity could be in a monopsony and still be subject to price adjustments being approved by a commission of regulators.

8. For instance, a hydro IPP will be bound by it Power Purchase Agreement as well as a water rights license issued by a Ministry of Water Resources and then also an independent electricity regulator if the sector has one.

9. In such instances, the PPPDG must ensure that the package of PPP Agreements are back-to-back with other legal instruments within the regulatory framework. This will avoid complications in the post-award contract management phase.

4.1.9.2 Outputs

The main outputs of this procedure include the following:

1. PPP Legal and Institutional Feasibility Analysis Report

2. PPP Contract Monitoring Framework

3. Make sure the findings of this legal feasibility report are available as inputs for the subsequent procedure on recommending PPP Risk Allocation Framework

4.1.10 PROJECT ENVIRONMENTAL AND SOCIAL IMPACT ASSESSMENT

This procedure provides an overview of the environmental and social analysis needed for a proposed PPP project, which environmental permits will likely be required, how to determine the project's overall environmental feasibility, and which additional environmental and social impact mitigation measures the project would require.

Project proponents will need to comply with the specific requirements contained in the Environmental Impact Assessment Proclamation No. 299/2002 and applicable regulations, which this procedure does not intend to replace or modify.

A key characteristic shared by almost all infrastructure projects, including PPPs, is the size and nature of their impacts on the surrounding environment and populations. Such projects, therefore, often have significant impacts on the surrounding communities, natural resources including air quality, water quality, land as well as the flora and fauna. In order for a PPP, or any

other infrastructure project to remain viable throughout its lengthy operating life, its environmental and social impact must be both accurately projected as well as carefully managed and monitored. Such environmental and social impact assessments are not only required under law, they are also needed to inform private service providers of the level of environmental and social risk a PPP project is likely exposed to and the requirements to properly manage and minimize these risks. Often potential impacts can be reduced or mitigated through alternative designs and technologies, which usually add to the cost of the project. It is important, therefore, to confirm that a proposed PPP project is still financially viable after the new costs and requirements of environmental and social mitigation measures are added to the project.

4.1.10.1 Implementation Procedure

1. Selecting the Appropriate level of Environmental and Social Impact Assessment & Permitting:

    a. The first step in this procedure is to decide what level of environmental and social feasibility analysis is required for the proposed PPP project.

    b. Ethiopia's legal framework on environmental and social analysis and approvals will guide the task to be carried out. The legal framework for ESIA includes the Environmental Impact Assessment Proclamation No. 299/2002, the Directive No.2/ 2008 issued to determine the categories of projects subject to the Environmental Impact Assessment pursuant to Proclamation No. 299/ 2002, the Environmental Policy of Ethiopia (1997), and existing ESIA Procedural Guidelines.

2. Conducting the Environmental and Social Impact Assessment. The PPP project environmental and social impact analysis will include the following components:

    a. Provide accurate baseline information about the environmental and social conditions of the project;

    b. Provide reliable information on the potential environmental and social impacts of the project, including the nature of these impacts, their magnitude, their range and distribution, their duration, and which stakeholder groups would be affected;

    c. Identify appropriate and effective mitigation measures for these impacts, including estimating the costs and other requirements;

    d. Assess and recommend the best alternative for properly mitigating and minimizing these environmental and social impacts taking into consideration the economic, financial, social, and environmental costs and benefits of these options;

    e. Provide the basis for developing an effective environmental and social management plan for the design, construction, and operation of the entire project.

3. These components are only indicative. Project proponents must consult the laws and regulations of Ethiopia and comply with its national ESIA requirements, which list in more detail the required contents of the environmental and social impact assessment.

4.1.10.2 Outputs

The key steps in developing the Terms of Reference for the Environmental and Social Impact Assessment will include the following:

	Key Environmental and Social Impact Assessment Tasks	Proposed PPP Project Answer and Explanation
1	The condition of the project's environmental and social context and resources	
2	Identify all of the likely environmental and social impacts and other environmental concerns	
3	Identify the specific, most-important environmental and social impacts that required detailed analysis	
4	Identify alternative measures and strategies that can be taken to mitigate these impacts, including their economic, financial, social and environmental requirements and benefits	
5	Identify all of the affected interests and stakeholders who should be consulted in carrying-out the ESIA study	
6	Recommend the option or strategy that provides the best overall (economic, financial, social, and& other) solution to for the project's environmental and social viability.	
7	Identify those key outputs of the ESIA study that should be managed and monitored as part of the project's environmental management and sustainability plan	

The key contents and requirements that an Environmental and Social Impact Assessment will address include the following:

	Outline of Key and Social Environmental Impact Assessment Requirement	Proposed PPP Project Answer and Explanation
1	Introduction & Background:<br>▪ Description of the approach to the ESIA<br>▪ Description of the policy, legal & administrative framework within which the ESIA is being prepared<br>▪ Description of project, its spatial needs & areas to be affected by the project<br>▪ Description of projects socio-economic benefits & impacts<br>▪ Description of other project impacts including resources consumed, wastes produced, etc.<br>▪ Summary of any relevant environmental and social studies completed	
2	Existing Environmental Conditions, Analysis of Alternatives and Preferred Design	
3	Direct & Indirect Ecological Impacts, including impacts on Valued Ecosystem Components (VECs) watersheds, key species, water supply, nature reserves, landscapes, housing, areas of socio-economic activity, & indigenous groups:<br>▪ Energy, waste & resource impacts<br>▪ Conservation & reparation potential<br>▪ Preservation of urban, historic, & cultural qualities	
4	Resettlement and Land Acquisition Needs, including a resettlement plan that includes:<br>▪ Numbers of people affected<br>▪ Their livelihoods	

	Outline of Key and Social Environmental Impact Assessment Requirement	Proposed PPP Project Answer and Explanation
	▪ Assets and other socio-economic activities<br>▪ Identification of land values	
5	Other Socio-Economic Impacts & Needs for Analysis, including:<br>▪ Assessments of poverty, poverty alleviation and benefits distribution<br>▪ Impacts on indigenous peoples and genders	
6	Methods for Mitigating Environmental Impacts & Required Costs	
7	An Environmental Management Plan, including:<br>▪ Mitigation<br>▪ Monitoring<br>▪ Environmental Construction Guidelines<br>▪ Resettlement & Rehabilitation Action Plan	
8	Details of Stakeholder Consultations Undertaken	

The relevant line Ministry or the EFCCC will review the ESIA study and provide their feedback. If at this stage the study carried out is full blown ESIA, it is also required that the relevant authority provides an ESIA clearance, in accordance with the 2002 EIA Proclamation.

4.1.11 PROJECT RISK IDENTIFICATION

Risk identification is the process of determining which specific possible risk events (such as increases in the costs of land to be acquired, higher than planned construction costs, higher than planned operating costs, less than projected levels of demand for the project, etc.) are the most important in determining whether the project will be viable as a PPP or not.

By this point, the project's technical, financial, economic, legal, institutional, and environmental feasibility analyses have all indicated some of the major risks, or unplanned changes, that could threaten the viability of the project. In PPP projects in particular, the end result of a given risk is that the project would become financially unviable. This procedure of identifying the project's most important risks is necessary in order to come up with an effective and acceptable risk allocation structure that ensures the project is viable. If for example, key risks are identified which show that there is a significant possibility the project may become financially unviable, then it is likely that additional credit enhancements, including risk-sharing by the Government or other forms of public sector supports may be needed in the project's final risk allocation structure.

4.1.11.1 Implementation Procedure

Using the PPP Risk Identification Matrix below as a tool, identify and describe all relevant, material risks for the project as a PPP:

PPP RISK IDENTIFICATION MATRIX		
	Risk Name and Description	How the Risk Impacts the PPP Project
1	Land Availability and Acquisition. Land needed for the project is not available or has not been acquired. There uncertainty over how much it would cost to acquire needed land and its timing	▪ Land is owned by the Government of Ethiopia. Although the Government (Regional/local) will allocate land for infrastructure projects it is understood that it is the private sector's responsibility to handle compensation with existing users. In other countries this tends to be handled by the government and may be an area that presents the private party with a risk of unacceptable costs and delays particularly in respect of large road/rail projects bordering densely populated areas.

PPP RISK IDENTIFICATION MATRIX		
	Risk Name and Description	How the Risk Impacts the PPP Project
		▪ This could increase the construction cost of the project beyond what is<br>▪ planned<br>▪ This could significantly delay construction, adding interest costs during<br>▪ construction and delaying when the project can earn its first revenues
2	Land Unsuitability. Unanticipated adverse ground conditions are discovered	▪ This could increase construction costs as new lands need to be identified and purchased<br>▪ This could increase construction costs as more expensive pilings or foundation strengthening must be built.<br>▪ This could significantly delay construction, adding interest costs during construction and delaying when the project can earn its first revenues
3	Environmental. The project causes major environmental impacts on its surrounding natural resources	▪ Neighboring residents dependent upon these natural resources could lose their livelihoods, or lives.<br>▪ The project could have to pay significant new, large sums to mitigate or correct these environmental damages<br>▪ The project could have to pay significant new, large sums in fines, penalties, or punitive damages<br>▪ The project may have to cease operations altogether or until it has successfully mitigated the damages
4	Climate. Climate change and climate related natural hazards (e.g. floods, droughts, landslides, cyclones, extreme heat, wildfires)	▪ Exposure and vulnerability to risks associated with climate change and climate related natural hazards could negatively affect project implementation and its longevity.<br>▪ The project could have to conduct in-depth climate risk impact assessment to calculate the incremental cost required for addressing climate risks.<br>▪ The project may have to cease operations altogether or until it has successfully incorporated sufficient adaptation and/or mitigation measures.
5	Health, safety, permits/licenses. Regulations and standards on health, safety, permitting, licenses, etc. are not complied with	▪ Workers or neighboring residents could suffer poor health, injuries, or other safety consequences.<br>▪ The project could have to pay significant new, large sums to mitigate or correct these health/safety damages<br>▪ The project could have to pay significant new, large sums in fines, penalties, or punitive damages<br>▪ The project may have to cease operations altogether or until it has successfully corrected these violations
6	Currency availability and transferability. Foreign currency is not available to transfer funds from local to hard currency Profits earned by the PPP project inside the country cannot be repatriated to its owners outside the country	▪ Convertibility: Where a PPP company is receiving payments in local currency but has to incur costs in a foreign currency to service foreign debt, purchase materials that are only available abroad or pay expatriate workers in foreign currency, it will be concerned to ensure that exchange-control laws of the host country will not prevent the company from meeting its foreign currency liabilities. Where foreign investors are involved, they will also be concerned to ensure that they can repatriate profits off-shore.<br>▪ Exchange rate fluctuation risk: Currency fluctuation risks will depend on asset type, project costs and the project revenues available. As an example, if project revenues are available in foreign currencies and debt finance is available in that same foreign currency, this provides a natural

PPP RISK IDENTIFICATION MATRIX		
	Risk Name and Description	How the Risk Impacts the PPP Project
		hedge against the currency exchange rate and convertibility risks depending on the volatility of the foreign currency revenue. However, where project revenues are only available in a local currency and the only debt finance available is in a foreign currency, this currency mismatch creates an exchange rate risk.<br>▪ If this risk is present during the tendering phase, then international bidders will not bid on the project, and the tender may fail<br>▪ If this risk is present during the operating phase, investors will not be able to earn their projected financial returns, and may seek disputes, termination or damages.
7	Operating costs. The costs of operating the project are higher than they were expected to be	▪ This would reduce the profitability of the projects for its owners, and the creditworthiness or coverage ratio for its lenders<br>▪ Lenders may require more investments, such as reserve accounts, from owners<br>▪ Investors may try to request price or tariff increases from the Government or its contracting agency.
8	Interest Rates. Interest rates on the loans used to construct the project increase	▪ This would reduce the profitability of the projects for its owners, and the creditworthiness or coverage ratio for its lenders<br>▪ Lenders may require more investments, such as reserve accounts, from owners<br>▪ Investors may try to request price or tariff increases from the Government or its contracting agency.
9	Exchange Rate. The local currency depreciates in value relative to the hard currencies in which the PPP project's loans and equity investments are denominated	▪ This would reduce the profitability of the projects for its owners, and the creditworthiness or coverage ratio for its lenders<br>▪ Lenders may require more investments, such as reserve accounts, from owners<br>▪ Investors may require price or tariff increases from the Government or its contracting agency to be able to pay these higher debt service return on equity costs.
10	Market. The actual quantity of outputs or services demanded by users or the off-taker is less anticipated The project's tariffs or prices are not adjusted according to the escalation formula agreed upon	▪ This would reduce revenue and therefore also the profitability of the project for its owners, and the creditworthiness or coverage ratio for its lenders<br>▪ Lenders may require more investments, such as reserve accounts, from owners<br>▪ Investors may view this a contract violation and thus seek disputes, termination or damages.
11	Responsibility of Design. The Government has provided a faulty or inappropriate design	▪ This could increase construction costs as new, more expensive designs would have to be completed and built<br>▪ This could increase operating costs more than anticipated as a result of having to follow a faulty or inappropriate design.<br>▪ This could significantly delay construction, adding interest costs during construction and delaying when the project can earn its first revenues<br>▪ Investors may require price or tariff increases from the Government or its contracting agency to be able to pay these higher construction and operating costs.
12	Detailed Design, Specifications and Standards. The project's performance	▪ This could increase construction costs as new designs may have to be completed and built

PPP RISK IDENTIFICATION MATRIX		
	**Risk Name and Description**	**How the Risk Impacts the PPP Project**
	standards and design specifications are inappropriate for the project's needs	▪ This could increase operating costs more than anticipated as a result of having to follow an inappropriate design.<br>▪ This could significantly delay construction, adding interest costs during construction and delaying when the project can earn its first revenues<br>▪ The private developer may have to pay penalties to the contracting agency for not be able to the project's minimum, contracted performance standards
13	Design Date. Wrong or inaccurate data was used during the project's construction	▪ This could increase operating costs more than anticipated as a result of having to follow an inappropriate design.<br>▪ This could significantly delay construction, adding interest costs during construction and delaying when the project can earn its first revenues<br>▪ The private developer may have to pay penalties to the contracting agency if it is not able to the project's minimum, contracted performance standards
14	Procurement and construction. Completion of the project construction was delayed	▪ This could increase construction costs through higher interest-during construction costs<br>▪ This could delay when the project can earn its first revenues<br>▪ The private developer may have to pay penalties to the contracting agency for not be able to start-up operations by the contracted deadline
15	Construction Cost. Total construction cost was more than anticipated	▪ This would reduce the profitability of the projects for its owners, and the creditworthiness or coverage ratio for its lenders<br>▪ Lenders may require more investments, such as reserve accounts, from owners<br>▪ Investors may try to request price or tariff increases from the Government or its contracting agency.
16	Program. The completion of the project is delayed or there is a cost over-run due to faulty work scheduling	▪ This could increase construction costs through higher interest-during construction costs<br>▪ This could delay when the project can earn its first revenues<br>▪ The private developer may have to pay penalties to the contracting agency for not be able to start-up operations by the contracted deadline
17	Operation. The project is not able to function and operate as fully as had been Anticipated	▪ This could reduce the project's revenues, if it its outputs are lower than anticipated<br>▪ This could increase operating costs if more needs to be spent on operating costs to achieve higher levels of output.<br>▪ This could significantly delay construction if a new, corrected design must be completed and built.<br>▪ The private developer may have to pay penalties to the contracting agency if it is not able to the project's minimum, contracted output or performance standards
18	Maintenance. The project and its assets are not properly maintained	▪ The project could face unscheduled outages, reducing its revenues, it creditworthiness and its profits<br>▪ The project may face higher and sooner than anticipated asset rehabilitation, renewal, and replacement costs on its un-maintained assets

PPP RISK IDENTIFICATION MATRIX		
	**Risk Name and Description**	**How the Risk Impacts the PPP Project**
		▪ If the project is not able to meet its minimum, contracted availability standards, it may need pay penalties to the contracting agency
19	Ancillary features. Ancillary infrastructure services that the project needs, such as approach roads, interconnection facilities, etc. are not provided and completed on Time	▪ This could delay the date when the project can earn its first revenues, reducing its creditworthiness for lenders and profitability for investors<br>▪ The developer may seek either price increases or damages from the contracting agency or Government body responsible for completing and delivering the ancillary services on time.
20	Transfer. The condition of the project's assets at the end of the contract term when they are transferred back to Government, is not in compliance with the PPP contract's maintenance and performance standards	▪ This could increase the costs to the Government and its contracting agency of renewing and rehabilitating the transferred assets in order to keep them operational
21	Regulatory. The terms and conditions of the PPP contract about the private operator's ability to collect revenues and to seek reasonable tariff increase in accordance with the contract's price escalation formula are not fulfilled; or New laws or regulations are passed which increase the costs or reduce the revenue of the PPP contractor without fair compensation	▪ This would reduce the profitability of the projects for its owners, and the creditworthiness, or coverage ratios, for its lenders<br>▪ ·· Lenders may require more investments, such as reserve accounts, from owners<br>▪ ·· The developer may claim a dispute or seek compensation damages from the contracting agencies for the lost revenue, increased costs, or lost<br>▪ profits from the regulatory action
22	Political/Sovereign. The Government nationalizes the project	▪ The private developer may seek damages for breach of contract<br>▪ The Government may compensate the private developer at a level that is below its costs, making it unable to repay the balances of the loans is owes to lenders, and expected profits to owners<br>▪ If there is political risk insurance (such as from MIGA) or a Partial Risk Guarantee (such as from the WB or EBRD) then these
23	The project is unable to perform due to terrorism, riots, war, or natural catastrophes (earthquakes, flooding, etc.)	▪ The project may be terminated if damage is fatal or complete<br>▪ If the damage is partial, the project may have a time limit within which to return to operation.<br>▪ If the project has applicable insurance, it may file a claim

4.1.11.2 Outputs

The main output from this procedure is a Risk Identification Report that features the completed risk identification matrix above. This report will also feature additional explanations of the impacts of key risks and why they are considered important for this given project.

4.1.12 PROJECT RISK ANALYSIS AND MITIGATION

This is the process of estimating the size of the impact of each major risk to the PPP project as well as estimating the probability that this specific risk event will occur to estimate the total cost of the risk, in quantitative terms, to the proposed PPP project.

The purpose of this procedure is to provide a more transparent and measurable valuation of the costs of each relevant risk for an infrastructure project. PPPs seek to break out of the patterns of decades of conventionally procured infrastructure projects, where large expensive projects have been planned and approved, but then fail to perform because risks are not well-managed or properly "priced" in the public sector. The key to breaking out of this vicious cycle is to consistently and transparently estimate the cost of these risks as a combination of both the size of their impacts and the probability of the risk event occurring. Once risks are systematically and consistently analyzed in this procedure, the next procedure, PPP Risk Allocation, will recommend who should be made responsible for each risk, based upon the analysis that results from this procedure.

4.1.12.1 Implementation Procedures

1. Assemble a workshop(s) of these experienced Contracting Authority and public sector management specialists to draw on both their decades of professional experience and on actual historical data.

2. Using the risks identified through the previous procedure's Risk Identification Matrix for each risk, estimate the most likely cost of each risk event happening as well as the most likely probability of the risk event actually occurring.

3. To assist the completion of this process, a risk analysis template can be used that proposes several scenarios for each risk event, such as:

    a. Catastrophic Scenario. The maximum possible cost from the risk event occurring;

    b. Critical Scenario. The costs from a large impact from the risk event occurring;

    c. Moderate Scenario: The costs from a medium-sized impact from the risk event occurring;

    d. No Change Scenario: No risk event occurs, and the costs of the project are what they were predicted to be;

    e. Optimistic Scenario: The lower than planned costs due to better-than-planned management of the given risk.

PPP Risk Size/Impact Cost Estimation					
	**Catastrophic Scenario**	**Critical Scenario**	**Moderate Scenario**	**No Change Scenario**	**Optimistic Scenario**
**Project Risk 1**	Cost of catastrophic risk event for Risk 1	Cost of critical risk event for Risk 1	Cost of moderate risk event for Risk 1	No cost (risk event #1 does not occur)	Lower cost (in risk event #1 area
**Project Risk 2**	Cost of catastrophic risk event for Risk 2	Cost of critical risk event for Risk 2	Cost of moderate risk event for Risk 2	No cost (risk event #2 does not occur)	Lower cost (in risk event #2 area
**Project Risk 3**	Cost of catastrophic risk event for Risk 3	Cost of critical risk event for Risk 3	Cost of moderate risk event for Risk 3	No cost (risk event #3 does not occur)	Lower cost (in risk event #3 area

4. Estimate the probability, expressed as a percentage, of the risk scenario identified by the above matrix occurring. To assist the completion of this process, a risk probability template can be used that proposes several scenarios for each risk event, such as:

    a. Catastrophic Scenario: The probability of a catastrophic version of the risk event occurring;

    b. Critical Scenario: The probability of a critical version of the risk event occurring;

    c. Serious Scenario: The probability of a serious version of the risk event occurring;

    d. No Change Scenario: The probability that the costs of the project are what they were predicted to be;

    e. Optimistic Scenario: The probability that project costs are lower than planned costs due to better-than-planned management of the given risk.

PPP Risk Probability Estimation Matrix						
	Catastrophic Scenario	Critical Scenario	Moderate Scenario	No Change Scenario	Optimistic Scenario	Sum of Probabilities
**Project Risk 1**	Probability (%) of a catastrophic risk event for Risk 1	Probability (%) of a critical risk event for Risk 1	Probability (%) of a moderate risk event for Risk 1	Probability (%) of a no risk event #1	Probability (%) of optimistic management of risk event #1	=100%
**Project Risk 2**	Probability (%) of a catastrophic risk event for Risk 2	Probability (%) of a critical risk event for Risk 2	Probability (%) of a moderate risk event for Risk 2	Probability (%) of a no risk event #2	Probability (%) of optimistic management of risk event #2	=100%
**Project Risk 3**	Probability (%) of a catastrophic risk event for Risk 3	Probability (%) of a critical risk event for Risk 3	Probability (%) of a moderate risk event for Risk 3	Probability (%) of a no risk event #3	Probability (%) of optimistic management of risk event #3	=100%

5. Estimate the Probability-Weighted Cost of Each PPP Project Risk. For each scenario, multiply the size of the estimated impact (expressed as a cost), by the estimated probability (expressed as a percentage) of that sized risk scenario occurring. Then add up the cost of all of these scenarios for that risk. This total is the probability-weighted cost of each risk.

4.1.12.2 Outputs

The main outputs from these tasks will include the following:

- The Project Management Team will focus on the estimated size of each risk's probability-weighted cost to gain an understanding of how sensitive the project's overall viability may be to a given a risk. Risks that have high probability-weighted costs will need to be carefully allocated in the following procedure and if they are unusually large, they may need to be shared or may require additional contributions or credit enhancements from the public sector.

PPP Risk Probability-Weighted Cost Estimation						
	**Catastrophic Scenario**	**Critical Scenario**	**Moderate Scenario**	**No Change Scenario**	**Optimistic Scenario**	**Sum of Probabilities**
**Project Risk 1**	Probability (%) of a catastrophic risk event for Risk 1 **X** Cost of catastrophic risk event for Risk 1	Probability (%) of a critical risk event for Risk 1 **X** Cost of critical risk event for Risk 1	Probability (%) of a moderate risk event for Risk 1 **X** Cost of moderate risk event for Risk 1	Probability (%) of a no risk event #1 **X** No cost (risk event #1 does not occur)	Probability (%) of optimistic management of risk event #1 **X** Lower cost (in risk event #1 area	=Sum (Total Probability – Weighted Cost of Project Risk #1)
**Project Risk 2**	Probability (%) of a catastrophic risk event for Risk 2 **X** Cost of catastrophic risk event for Risk 2	Probability (%) of a critical risk event for Risk 2 **X** Cost of critical risk event for Risk 2	Probability (%) of a moderate risk event for Risk 2 **X** Cost of moderate risk event for Risk 2	Probability (%) of a no risk event #2 **X** No cost (risk event #2 does not occur)	Probability (%) of optimistic management of risk event #2 **X** Lower cost (in risk event #2 area	=Sum (Total Probability – Weighted Cost of Project Risk #2)
**Project Risk 3**	Probability (%) of a catastrophic risk event for Risk 3 **X** Cost of catastrophic risk event for Risk 3	Probability (%) of a critical risk event for Risk 3 **X** Cost of critical risk event for Risk 3	Probability (%) of a moderate risk event for Risk 3 **X** Cost of moderate risk event for Risk 3	Probability (%) of a no risk event #3 **X** No cost (risk event #3 does not occur)	Probability (%) of optimistic management of risk event #3 **X** Lower cost (in risk event #3 area	=Sum (Total Probability – Weighted Cost of Project Risk #3)

- A PPP Project Risk Analysis Report summarizing both the process used to estimate risk costs, risk probabilities, and probability-weighted risk costs as well as the end results.

4.1.13 RECOMMENDED PPP RISK ALLOCATION STRUCTURE

Risk allocation is the process of determining which parties to make responsible for bearing the impacts of and for managing and controlling each project risks. These risks, which were identified and analyzed by the previous procedures will each be clearly allocated between the different parties to the project, including: the private service provider, the Government/Contracting Authority, or shared between the two.

The purpose of this procedure is to complete the process that was begun by the previous procedures and to propose the most effective strategy for allocating all of the relevant project risks between the parties. Each risk will be assigned to and made the responsibility of the party that is best able to

manage, to control, and ultimately to minimize that risk. If this were not done then a party would be exposed to a risk that it would have no control over. For example, when it comes to operating risk (the risk that the project's operating costs will be higher than anticipated) this is a risk that the private service provider is best able to control. To assign this risk to the Contracting Authority would expose it to having to pay for rising O&M costs, over which it would have little ability to control, as daily operational management would be in the hands of the private partner. Similarly, political and regulatory risks are ones that the private partner has no legal ability to control. Some risks, however, such as market and demand risks in road projects, usually neither side can fully control, and therefore risk allocation structures must often include mechanisms for sharing these between the parties.

4.1.13.1 Implementation Procedure

1. PPP Risk Mitigation Matrix. Use the matrix on PPP risk mitigation techniques to assess how the project's key risks can be best minimized, managed, and mitigated, and by which party.

PPP RISK IDENTIFICATION MATRIX			
	**Risk Name and Description**	**Common Risk Mitigation Techniques**	**Project Management Team Mitigation Recommendation**
1	Land Availability and Acquisition. Land needed for the project is not available or has not been acquired. There uncertainty over how much it would cost to acquire needed land and its timing	▪ Government should make available the project land at the point of tender.	
2	Land Unsuitability. Unanticipated adverse ground conditions are discovered	▪ The private sector should conduct proper due diligence on the site suitability	
3	Environmental. The project causes major environmental impacts on its surrounding natural resources	▪ The private sector should conduct proper due diligence to assess any major environmental impacts and associated risk mitigation costs.	
4	Climate. Climate change and climate related natural hazards (e.g. floods, droughts, landslides, cyclones, extreme heat, wildfires)	▪ The Government should screen all projects using a standardized climate and disaster risk screening methodology<br>▪ For projects deemed moderate to high risk by the screening methodology, the private sector should conduct proper due diligence to assess potential impact of climate risk on the project and the cost associated with addressing this risk, including the incremental cost of introducing the appropriate adaptation and mitigation measures	
5	Health, safety, permits/licenses. Regulations and standards on health, safety, permitting, licenses, etc. are not complied with		

PPP RISK IDENTIFICATION MATRIX			
	Risk Name and Description	Common Risk Mitigation Techniques	Project Management Team Mitigation Recommendation
6	Currency availability and transferability. Foreign currency is not available to transfer funds from local to hard currency. Profits earned by the PPP project inside the country cannot be repatriated to its owners outside the country	▪ Government provides a guarantee on currency availability and ability to repatriate profits<br>▪ Minimizing foreign content and promoting local production, as relevant.	
7	Operating costs. The costs of operating the project are higher than they were expected to be	▪ The private sector could mitigate by outsourcing operations and maintenance to a third party, such as a specialized O&M contractor	
8	Interest Rates. Interest rates on the loans used to construct the project increase	▪ The private sector could finance the project with a fixed-rate loan or use a hedge on interest rates (if available)	
9	Exchange Rate. The local currency depreciates in value relative to the hard currencies in which the PPP project's loans and equity investments are denominated	▪ The private sector could use currency swaps (if available)	
10	Market. The actual quantity of outputs or services demanded by users or the off-taker is less anticipated The project's tariffs or prices are not adjusted according to the escalation formula agreed upon	▪ Government could provide a minimum revenue guarantee; the private sector could arrange for a standby facility in the project financing<br>▪ Government may need to compensate private investor if the non-adjustment is in breach of contract	
11	Responsibility of Design. The Government has provided a faulty or inappropriate design	▪ Government should appoint experienced and reputable consultants responsible for providing and reviewing the Government's design	
12	Detailed Design, Specifications and Standards. The project's performance standards and design specifications are inappropriate for the project's needs	▪ The private sector could mitigate this risk to a third party through a fixed price turnkey EPC contract.<br>▪ Private sector should appoint experienced and reputable design and construction consultants	
13	Design Date. Wrong or inaccurate data was used during the project's construction	▪ The private sector could shift this risk to a third party through a fixed price turnkey EPC contract.	
14	Procurement and construction. Completion of the project construction was delayed	▪ The private sector could shift this risk to a third party through a fixed price turnkey EPC contract.	
15	Construction Cost. Total construction cost was more than anticipated	▪ The private sector could shift this risk to a third party through a fixed price turnkey EPC contract.	

PPP RISK IDENTIFICATION MATRIX			
	Risk Name and Description	Common Risk Mitigation Techniques	Project Management Team Mitigation Recommendation
16	Program. The completion of the project is delayed or there is a cost over-run due to faulty work scheduling	▪ The private sector could shift this risk to a third party through a fixed price turnkey EPC contract.	
17	Operation. The project is not able to function and operate as fully as had been anticipated	▪ The private sector could outsource<br>▪ operation & maintenance to a specialized third-party contractor	
18	Maintenance. The project and its assets are not properly maintained	▪ The private sector could outsource<br>▪ maintenance to a specialized third-party contractor	
19	Ancillary features. Ancillary infrastructure services that the project needs, such as approach roads, interconnection facilities, etc. are not provided and completed on Time	▪ The private sector could insist that these non-provisions constitute a breach of contract, or warrants compensatory damages from the Government.	
20	Transfer. The condition of the project's assets at the end of the contract term when they are transferred back to Government, is not in compliance with the PPP contract's maintenance and performance standards	▪ The private sector could mitigate by<br>▪ ensuring there is proper maintenance, such as through outsourcing to a maintenance contractor	
21	Regulatory. The terms and conditions of the PPP contract about the private operator's ability to collect revenues and to seek reasonable tariff increase in accordance with the contract's price escalation formula are not fulfilled; or New laws or regulations are passed which increase the costs or reduce the revenue of the PPP contractor without fair compensation	▪ The private sector could mitigate through agreed compensatory changes to the contract	
22	Political/Sovereign. The Government nationalizes the project	▪ The private sector could mitigate through some forms of political risk insurance, e.g. from MIGA, OPIC or partial risk guarantees (PRGs) from WB	
23	The project is unable to perform due to terrorism, riots, war, or natural catastrophes (earthquakes, flooding, etc.)	▪ The Government and private sector could jointly mitigate against man-made events through some form of political insurance, e.g. MIGA and OPIC	

2. PPP Risk Allocation Matrix. Use the second matrix as a guideline to analyze each identified, relevant project risk and to decide which party to allocate each risk: Government, Private, or Shared. While the matrix below suggested risk allocations for typical PPPs, each project will develop its own unique risk allocation structure that best

suits the individual needs of that specific project. Provide an explanation (as provided by the last column) for why each risk should be allocated to each party as shown.

Table 4-4: PPP Risk Identification Matrix

	Risk Name and Description	Risk Allocation			Explanation
		Shared	Government	Private	
1	Land Availability and Acquisition. Land needed for the project is not available or has not been acquired. There uncertainty over how much it would cost to acquire needed land and its timing				
2	Land Unsuitability. Unanticipated adverse ground conditions are discovered				
3	Environmental. The project causes major environmental impacts on its surrounding natural resources				
4	Health, safety, permits/licenses. Regulations and standards on health, safety, permitting, licenses, etc. are not complied with				
5	Currency availability and transferability. Foreign currency is not available to transfer funds from local to hard currency Profits earned by the PPP project inside the country cannot be repatriated to its owners outside the country				
6	Operating costs. The costs of operating the project are higher than they were expected to be				
7	Interest Rates. Interest rates on the loans used to construct the project increase				
8	Exchange Rate. The local currency depreciates in value relative to the hard currencies in which the PPP project's loans and equity investments are denominated				
9	Market. The actual quantity of outputs or services demanded by users or the off-taker is less anticipated The project's tariffs or prices are not adjusted according to the escalation formula agreed upon				
10	Responsibility of Design. The Government has provided a faulty or inappropriate design				
11	Detailed Design, Specifications and Standards. The project's performance standards and design specifications are inappropriate for the project's needs				
12	Design Date. Wrong or inaccurate data was used during the project's construction				
13	Procurement and construction. Completion of the project construction was delayed				
14	Construction Cost. Total construction cost was more than anticipated				
15	Program. The completion of the project is delayed or there is a cost over-run due to faulty work scheduling				
16	Operation. The project is not able to function and operate as fully as had been anticipated				
17	Maintenance. The project and its assets are not properly maintained				
18	Ancillary features. Ancillary infrastructure services that the project needs, such as approach roads, interconnection facilities, etc. are not provided and completed on time				

	Risk Name and Description	Risk Allocation			Explanation
19	Transfer. The condition of the project's assets at the end of the contract term when they are transferred back to Government, is not in compliance with the PPP contract's maintenance and performance standards				
20	Regulatory. The terms and conditions of the PPP contract about the private operator's ability to collect revenues and to seek reasonable tariff increase in accordance with the contract's price escalation formula are not fulfilled; or<br>New laws or regulations are passed which increase the costs or reduce the revenue of the PPP contractor without fair compensation				
21	Political/Sovereign. The Government nationalizes the project				
22	The project is unable to perform due to terrorism, riots, war, or natural catastrophes (earthquakes, flooding, etc.)				

4.1.13.2 Outputs

The main outputs from these tasks will include the following:

1. Prepare a PPP Risk Allocation Report that features the completed risk identification matrix above. This report will also feature relevant explanations for why each risk should be allocated in the manner recommended.

- This risk allocation structure will become the basis for the design and drafting of the actual PPP Contract, is explained below.

4.1.14 VALUE FOR MONEY ANALYSES/BENEFITS

This is the procedure to be followed to develop a PPP Value for Money (VfM) assessment. This assessment is carried out to determine which procurement option (i.e. public sector procurement or PPP) for the delivery of a Public Service will result in higher net economic benefit for the government.

Figure 4-6: Value for Money in PPP projects

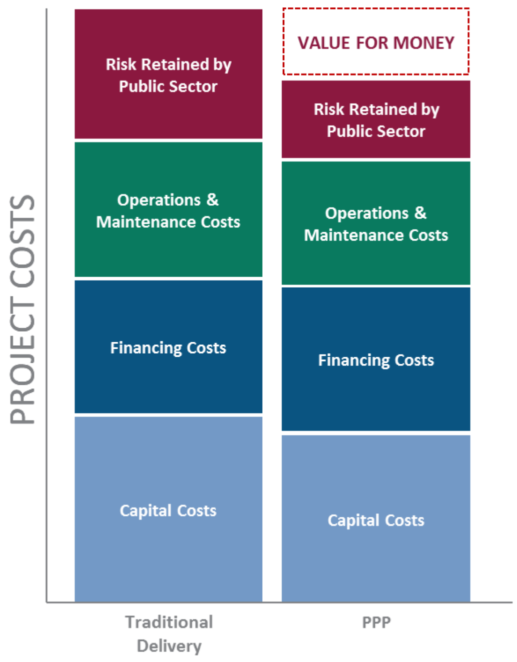

VfM is the test of whether the PPP procurement option is better than the traditional procurement mechanism.

On the one hand, PPPs have higher financing and transaction costs than traditional procurement. On the other hand, the main value drivers of PPP in comparison to traditional procurement are:

- the long term nature of a PPP contract - leading to construction cost savings and life-cycle cost efficiencies (on time and on budget), and
- the allocation of risks to the private contractor – leading to less construction cost overruns and delays and better quality of services.

The VfM analysis is forward looking and is based on assumptions of what it would cost the Private Party or a Public Entity to deliver the Public Service Activity. Due to this speculative element, the VfM assessment shall be repeated following negotiation with the Private Party to confirm that the project still yields Value for Money for the government.

There are two common approaches to developing the VfM assessment, although on most PPP projects both methods are implemented to allow a robust and comprehensive discussion of all the project benefits. The methods are:

1. Quantitative VfM; and
2. Qualitative VfM.

4.1.14.1 Implementation Procedure

4.1.14.1.1 Quantitative VfM

The approach to quantitative VfM analysis relies on comparing the life cycle costs of the project as delivered through traditional procurement versus with a PPP approach (i.e. the Public Sector Comparator [PSC]). This approach assumes that under either procurement approach, the project outcomes would be the same. While this assumption may not be entirely accurate, the purpose of the VfM assessment is to determine the cost for achieving a specific outcome. Thus, project outcomes are harmonized, under both the public and private delivery, for the purpose of this analysis.

1. Estimate the Public Entity's Operating Expenses (OPEX), which, for simplicity can be estimated as a percentage of CAPEX if the information is not available (e.g. Greenfield projects) or drawn from the base case costs in the financial model. OPEX includes line items such as staff wages, tools and materials, consumables, etc.

2. Estimate Capital Expenditures including, design, land, constructions, plant and equipment costs.

3. Estimate the cost of asset maintenance and renewals over the lifetime of the project to obtain the whole life cycle cost of the project.

4. If cost items were drawn from the financial model, any assumptions on operational efficiency as a result of private delivery will be adjusted out of the estimates. For instance, if the private delivery model assumes that labour productivity of staff would be approximately 80 percent, then the public delivery model shall reflect the true estimate of labour productivity in the public enterprise.

5. Estimate project revenues (including contributions from end users). If it is assumed that private party would be able to generate additional sources of revenue from end-users that would not be possible with government operations (due to legal reasons, for instance), then such revenues shall be excluded from the project revenues.

6. The project costs shall then be adjusted to account for risks that Public Entities assume (risks that would have been transferred under a PPP, e.g. construction risks) under the traditional project delivery.

    a. Risks like construction delays, cost overruns, etc. all impose a cost for public sector delivery.

    b. These costs will be estimated (drawing on historical records on average project delays on public sector procurement, average cost overruns on similar sized public sector contracts).

    These costs are then added to the whole life cycle project costs to yield the Public Sector Comparator.

7. Estimate the PPP project costs, which will be drawn from the financial feasibility analysis and model. Any non-fiscal revenues (payments from end-users) will be excluded from

this estimation. Any tax payments will be added back to this cost as it reflects a transfer to the public sector.

8. Estimate the cost of developing and implementing the PPP transaction (cost of advisors, cost of time for PPPDG, Contracting Authority and Project Management Team members) and all other costs associated with developing the PPP.

9. Following the estimation of whole life cycle PSC and PPP costs, the results have to be discounted to present values to enable comparison in today's costs.

    a. The choice of a discount rate to use in this analysis is significant as it impacts the results of the VfM analysis.

    b. Some approaches prefer to use the government risk-free rate, as it reflects the opportunity cost of government funds

    c. Other approaches prefer to use the Social Discount Rate as a true measure of the society's value of time

    d. The approach to use would depend on government policy, as the choice of discount rate would need to be consistent for the assessment of all PPP projects in order to obtain an objective assessment of PPP VfM

10. Once the PSC and PPP costs have been discounted to present value, the two costs are compared. If the PSC costs are higher than the PPP costs, then it implies the delivery of the project results in Value for Money for society.

11. The figure below presents an illustration of the analytical steps described above using a hypothetical PPP project.

PPP Project		Year 1	Year 2	Year 3	Year 4	Year 5	Year 6	Year 7	Year 8	Year 9	Year 10	Year 11
PPP Dev. & Impl. Costs	$ 000	(250.0)	-	-	-	-	-	-	-	-	-	-
(-) PPP Management Costs	$ 000	-	(163.5)	(174.9)	(185.4)	(196.6)	(208.4)	(220.9)	(234.1)	(248.2)	(263.1)	(278.8)
(-) Foregone Operating Profit	$ 000	-	(44.6)	(185.3)	(342.2)	(455.5)	(482.8)	(511.8)	(542.5)	(575.0)	(609.5)	(646.1)
(+) Taxes Collected from Concessionaire	$ 000	-	-	24.2	74.4	107.9	119.4	132.1	145.4	159.7	174.9	191.3
**Total Costs of PPP Project**	**$ 000**	**(250.0)**	**(208.1)**	**(336.0)**	**(453.2)**	**(544.1)**	**(571.8)**	**(600.5)**	**(631.2)**	**(663.5)**	**(697.6)**	**(733.6)**

Public Sector Comparator (PSC)		Year 1	Year 2	Year 3	Year 4	Year 5	Year 6	Year 7	Year 8	Year 9	Year 10	Year 11
Revenues	$ 000	-	284.5	608.9	968.1	1,231.5	1,305.3	1,383.7	1,466.7	1,554.7	1,648.0	1,746.8
(-) Capital Costs	$ 000	(550.0)	-	-	-	-	-	-	-	-	-	-
(-) Operating Costs	$ 000	-	(239.9)	(423.6)	(625.9)	(776.0)	(822.5)	(871.9)	(924.2)	(979.7)	(1,038.4)	(1,100.8)
(-) Risk Adjustments:												
Capital Cost Overruns	$ 000	(222.74)	-	-	-	-	-	-	-	-	-	-
Operational Inefficiencies	$ 000	-	(251.71)	(495.58)	(765.14)	(963.57)	(1,021.39)	(1,082.67)	(1,147.63)	(1,216.49)	(1,289.48)	(1,366.84)
**Risk Adjusted PSC**	**$ 000**	**(772.7)**	**(207.1)**	**(310.3)**	**(422.9)**	**(508.1)**	**(538.6)**	**(570.9)**	**(605.2)**	**(641.5)**	**(680.0)**	**(720.8)**
**NPV of PPP Project**	**$000**	**(3,401.5)**										
**NPV of Risk Adj. PSC**	**$000**	**(3,740.4)**										
**Value for Money (VfM)**	**$000**	**338.9**										

12. The above presents a simple methodological approach to carrying out a quantitative VFM analysis. As the concept evolves and matures in its application to PPPs, different costs and benefits are added into the estimation. It is essential that for each cost or benefit added and adjusted out of the project life cycle costs, the PPPDG have a robust justification for the inclusion or exclusion.

4.1.14.1.2 Qualitative VfM

The qualitative VfM analysis presents a narrative as to whether a project fits the PPP concept and what general socio-economic benefits (improvement in public service delivery, expanded infrastructure, etc.) are delivered by PPPs. non-financial benefits.

A qualitative VfM analysis would also discuss the potential challenges involved in delivering public projects using PPPs. This includes loss of an important public sector tool to implement social programs and challenges in overseeing private sector delivery of public services.

VfM analysis should involve a combination of qualitative and quantitative approaches which will complement each other. Since the quantitative approach is only as good as the available data and other assumptions made, there is a risk of depending too heavily on quantitative analysis49. It is now considered best practice, tends to balance the quantitative approach with qualitative analysis.

4.1.14.2 Outputs

The main outputs of this procedure are:

1. Detailed estimates of public sector costs: The output from this task will be the cash flow for capital and operating expenditures.
2. Detailed development of risk impacts.
3. Detailed proposal for allocation of project risks (transferred, shared, or retained). The output from this task will be:
    a. Risk allocation worksheet
    b. PSC adjusted to risk cash flow
4. Estimation of the Private Party's costs (based upon risks transferred): The output from this task will be the Private Party's financial model demonstrating the projected cash flow of the Public Sector for a PPP structure.
5. Analysis of Value for Money benefits. The output of this task will be the comparison of the PSC adjusted risk cash flow to the PPP cash flow (from the Service Provider's model).
6. Affordability analysis: comparing PSC and Private Party's costs estimate to updated affordability limit to reconfirm project affordability. The output of this task will be a worksheet that calculates the NPV of the CAPEX, OPEX and Risks.

4.1.15 MARKET TESTING

This is the process of determining if both the general nature of the given PPP project, and the proposed risk allocation structure is adequate to attract qualified private sector investors and financiers.

---

[49] Some countries have however, like the United Kingdom to focus on qualitative factors alone.

The purpose of this procedure is to make sure that the project would receive sufficient private sector interest to attract serious, good-quality bids if the project were approved to advance to Phase 3's PPP Tendering Implementation. While international experience with PPPs has already shown strong interest by both local and international bidders in nearly all infrastructure sectors – there may be country-specific and project specific risks that shall be addressed before such bidders would participate in a specific PPP project in a specific country. Without this procedure, public authorities might risk tendering a given project, only to find out that both local and international firms have serious concerns about the project's proposed structure that prevent them from submitting any bids at all or end up with limited competition.

4.1.15.1 Implementation Procedure

Use the following Matrix as a Guideline to determine if there is likely to be serious private sector interest in the project as a PPP:

Private Sector Market Interest		
	Private Sector Market Interest Questions	Response
1	Are there previous examples of PPPs in the same sector, or for the same specific service in country?	
2	Are there previous examples of PPPs in the same sector, or for the same specific service in other countries?	
3	Do local contractors, investors, and lenders have the financial and technical resources and capacity required to undertake the PPP project on their own? Are outside or international resources required?	
5	Have local or international service providers expressed interest in the project during the initial, early stages of the project? Do they still express interest based upon the proposed risk allocation structure?	
6	What are the risks that the private sector can only accept at an unaffordable PPP tariff or price, and should additional enhancements risk mitigation instruments be considered in order to ensure better private sector interest in the projects, such more sharing of risks by the Government, partial guarantees, and other credit enhancements?	

4.1.15.2 Outputs

1. The main outputs from these procedures will include the following:

    a. Based upon the responses to the key market interest questions above, carefully evaluate if any changes should be considered to the project's risk analysis and allocation structure to ensure a stronger interest by serious private sector bidders.

    b. Prepare a Private Sector Market Interest Assessment Report.

4.1.16 PPP PROCUREMENT STRATEGY

This procedure elaborates the process for going to tender and selecting the PPP Partner. The objective of the process is to ensure that the selection procedure does not result in an outcome that erodes the Value for Money in the PPP approach. This means a selection scheme that targets the appropriate prospective private partners with the right mix of technical, managerial

skill, financial capacity and risk appetite to enter into a PPP relationship with the Contracting Authority.

Most infrastructure PPPs are likely to involve the single stage procurement system. Two stage and competitive dialogue tend to be used for very complex projects often involving new technology or innovative design. They involve much longer processes, tying up resources and incurring higher transaction costs.

The procurement strategy centers around two key tasks (i) Prequalification (ii) Request for Proposal. The matrix below presents some key questions that must be addressed by the PMT and the Contracting Authority in designing the procurement strategy. This list is not exhaustive and for each project, the unique project circumstances will illuminate other critical questions for the design of the procurement strategy. The Contracting Authority's advisor will present such considerations in the Feasibility Report in presenting the rationale for the selected procurement strategy.

Table 4-5: Key Questions on Procurement Strategy

	Prequalification
1	Whether and when to launch the prequalification process?
2	Should the prequalification process be open or restrictive?
3	What should be the evaluation criteria for the prequalification and how should the scoring be designed?
4	How many candidates should be shortlisted to compete at the Request for Proposal stage
5	Should restrictions/conditions be placed on the participation of international or local firms?
**Request for Proposal**	
1	The timing for the issue of the RFP and length of period allowed for bidders to respond
2	Whether or not site visits and other forms of reconnaissance on the proposed project are permitted
3	Whether the RFP and bid documents should be purchased or provided without charge to bidders
4	The value and duration of validity of any bid bond
5	The submission requirements and evaluation criteria for the Request for Proposal

4.1.16.1.1 Implementation Procedure

1. The Feasibility Study Due Diligence will provide the initial guidance for the design of the procurement strategy. Elements like whether to have an open competitive process or restricted shortlist, shortlisting criteria, the duration of the bid process and other factors are all determined by the conclusions from the feasibility due diligence investigations.

    a. The Technical Due Diligence will elaborate on the technically feasibility solutions for the proposed PPP activity. If the feasible solution involves a technology and skillset widely dispersed and available from multiple sources, then the PPPDG may choose to have an open competitive process to enable different solutions providers to propose the most cost effective option. A widely available solution may also mean the PPPDG may want to prequalify and shortlist a group of solution providers that best meet the needs of the proposed PPP.

b. The Legal Due Diligence will highlight what procurement methods and tactics are permissible within specific sectors or overall. For instance, a PPP involving the use of a public resource may require that the prospective private party also be prequalified for access to that resource (e.g. water rights license for hydro IPPs). This knowledge will influence the design of the prequalification criteria to ensure that shortlisting criteria for prospective candidates also take into account ancillary but critical requirements.

c. The Market Sounding activity will allow the PPPDG assess the market appetite for the PPP and determine the categories of prospective private parties that would be interested in the transaction. This information will not only inform bid process marketing activities such as road shows and investor conferences, but as well help the PPPDG to develop the Project Information Memorandum (PIM) and other bid documents to address the questions and comments that interested parties have on the project.

2. The PPP legal framework will also dictate what procurement activities are required for PPP transactions. In Ethiopia, the PPP framework mandates the prequalification activity for all PPP bid processes, except for Direct Negotiation where prequalification is run when deemed appropriate by the PPPDG.[50]

3. The PPP legal framework also identifies the permitted bid process model described in detail in Section 6:

    a. Single-stage bidding

    b. Two-stage bidding

    c. Competitive Dialogue

    d. Direct Negotiations

    e. Unsolicited Proposals

The choice of bid process is constrained by the methods permissible under the PPP legal framework in Ethiopia.

4.1.16.2 Output

1. The main output of this process will be a Procurement Strategy Document, identifying:

    a. The preferred bid process model based on the availability and type of solution proposed for the Public Service Activity under the PPP;

    b. The propose bid process duration considering factors like approval timelines within the public service, availability of funding;

[50] The PPP Proclamation requires the PPPDG to initiate a prequalification for all the bidding methods, even including Direct Negotiation transactions.

c. The structure and/or templates for the prequalification, Request for Proposal and accompanying evaluation criteria with rationale for the selection of specific criteria.

4.2 CONSOLIDATING THE PPP FEASIBILITY PROCEDURES

The PPP legal framework does not require the Contracting Authority to engage advisors for the basic PPP project selection and Pre-Feasibility analysis. When Contracting Authorities build capacity in PPPs, such analysis can be carried out internally. For the more rigorous Feasibility Study analysis, the legal framework states that the PPPDG will engage Transaction Advisors to work with the Contracting Authorities. It is however, important that Contracting Authorities through the Project Management Team, carefully oversee the progress and the key deliverables from the Transaction Advisors, to ensure that project deliverables meet this PPP Guidelines Manual's required procedures, which it is complete, and it provides a clear basis for subsequent decision-making and management of the PPP project.

The Project Feasibility Report shall include at a minimum the following sections.

1. **Introduction and Purpose of the Feasibility:** Outlining the Transaction Advisor's understanding of the Terms of Reference
2. **Needs Analysis:** Describing market dynamics for the public service being considered for PPP including detailed feedback from stakeholders and users in the sector on their experiences and expectations from the service provider
3. **Technical Due Diligence:** Describing the infrastructure solution for the deploying the public service as a PPP
4. **Legal Due Diligence:** Describing the legal and institutional framework governing the provision of the public service
5. **Social and Environmental Assessment:** Describing the needed safeguards to ensure vulnerable groups and the environment and protected in deploying a PPP service
6. **Financial Appraisal:** Presenting the simulated cash flows from the operation of the public service as a PPP
7. **Economic Impact Assessment:** Presents the cost-benefit analysis (CBA) for implementing the project (with and without project scenario)
8. **Risk Identification and Allocation:** Identifies risks associated with deploying PPPs and recommends actions to be taken by specific parties to mitigate the risk
9. **PPP Options Analysis:** Describes different PPP options for providing the public service and their unique structure
10. **Fiscal Commitments and Affordability**: Presents the direct project costs and potential contingent liabilities following from the proposed PPP structure.

11. **End-user Affordability:** Examines Public Service Activity consumers' ability to afford PPP services

12. **Value for Money Assessment:** Presents an objective assessment of whether the public service is best delivered via a PPP or by the public sector

13. **PPP Procurement Strategy:** Presents a strategy, work plan and timetable for procuring the public service via a PPP

The feasibility study sanitized from any commercially sensitive data will be disclosed to the public on the Contracting Authorities website.

A sample PPP Feasibility Study Table of Contents is provided in **Appendix C – Part 2**

RECOMMENDED PROCESS

Holding Investor Roadshows at the Earliest Stages of the Project

While all project details may not yet be finalised, engaging the market with the broad framework as early as possible allows the potential private sector investors/partners to investigate the project from the early stage and be prepared to develop qualified bids when the procurement process commences.

Additionally, early engagement of the private sector allows the PMT and Transaction Advisors to identify, at the early stage of the PPP process, key issues concerning the project from the private sector perspective, which otherwise may come up later in the transaction and drag the process. These issues can then be considered in the early development of PPP options.

The Contracting Authority to a PPP outlines specific outcomes against which the private party is expected to perform. The Project Feasibility Study shall present in clear and unambiguous terms the required outcome levels (service or infrastructure and service) expected from the private sector.

Outcome specifications differ for different PPPs. Omitting or inaccurately specifying outcome specifications could create problems in the PPP relationship. For instance, if a PPP project is procured or the provision of a shuttle service at an airport terminal and only specifies the frequency of service, but not the capacity of shuttles. The PPP operator may procure shuttles that do not provide sufficient capacity for current or anticipated passenger traffic, thereby creating delays in airport transfers for users. In this instance, the Contract Authority may judge the PPP operator for failing to perform, whereas the PPP operator can claim it is performing exactly to the specifications it proposed in its proposal.

The Contracting Authority shall be able to confidently define the precise outcomes required for the performance of public services. This clarity of definition will improve the offers received in response to any procurement requests for PPP partners.

The table on the outcomes from the PPP Feasibility Study, in **Appendix C** (Part I: Background References - Phase II), presents a list of structural questions the PPP Feasibility Study shall address and indicates the section of the feasibility report which shall address that question. In

the second column on the table, the outcomes that shall be prescribed in the PPP tender document or agreement are indicated.

The Contracting Authority and its PMT shall also ensure that the conclusions from the Feasibility Report allow it to make a recommendation on the proposed structure of the PPP project.

This indicative project structure will highlight:

- A definition of the project scope (services to be performed, consumers of the service, Key Performance Indicators, the duration of performance);
- An explanation of the business model indicating how the service provider earns revenue and manages its commercial obligations with other parties;
- Presentation of any segmentation or market categorizations imposed by the Contracting Authority (e.g. tender lots) for the project;
- A definition of the PPP model being adopted for the project;
- Any fiscal or contingent liability for the government; and
- The risk management framework that will government the PPP relationship and agreement.

This preliminary project structure must be approved by the PPP Board as part of the process of the approval of the Feasibility Report. The Contracting Authority with the support of the PMT shall submit to the PPPDG an Executive Memo (a sample template for such a memo is presented in **Appendix D: Part 7** summarizing the key elements of the feasibility report. The PPP Board will make its approval or rejection decision of the Feasibility Study based on the Executive Memo. The PPP Board is empowered by the PPP framework to make changes to the proposed PPP structure identified in the Feasibility Report.

If the Feasibility Report is approved, the PPP Project proceeds to the Project Structuring Phase.

Estimated Time Required: The amount of time required to complete the Feasibility Study will depend on the technical nature, the size, the impacts, and the complexity of the individual PPP project. It can take 3 to 10 months to carry out this procedure.

4.3 DISCLOSURE OF INFORMATION AT PROJECT STRUCTURING PHASE

During this phase of the PPP life cycle, the PPPDG must balance between keeping the general public informed and maintaining an element of confidentiality of the project findings and conclusions. For instance, if a feasibility study determines that ongoing public subsidies would be required for a new proposed transport system, it would be useful to inform the general public about plans for the ongoing feasibility study, however, the requirement for subsidy shall be withheld until the PPPDG has confirmed that there is the political support and capacity (budget) to commit to ongoing subsidy payments.

The criteria for deciding which information to disclose and which ones to withhold is whether or not the disclosed information has the potential of being contradicted. The

PPPDG must be seen to be consistent and precise in its announcements. This is useful for building credibility as a new PPP organization.

The PPPDG may also undertake an Early Market Engagement during this phase to help it gather more information about a prospective PPP project, investor appetite for PPPs or other critical information that will feed into the feasibility analysis. The PPPDG must have a clear process for this Early Market Engagement, and it involves ensuring non-discriminatory access to the event for all interested parties (Section 6.3.1 elaborates on this procedure).

List of Documents Provided in Appendix C - Phase 2 PPP Appraisal and Preparation
1. Part 1: Background References – Phase II: Outcomes from the Feasibility Study<br>***2. Part 2: Sample PPP Feasibility Study Table of Contents***<br>***3. Part 3: Fiscal Affordability Forms***

5 PHASE III: STRUCTURING AND DRAFTING THE TENDER AND CONTRACT DOCUMENTS

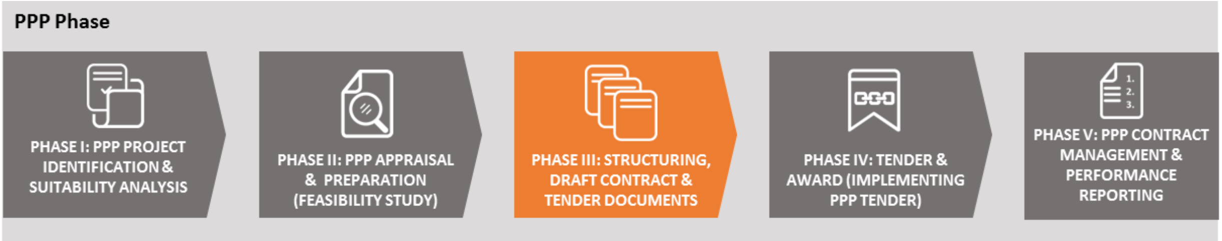

Figure 5-1: Overview of Tasks under Phase III

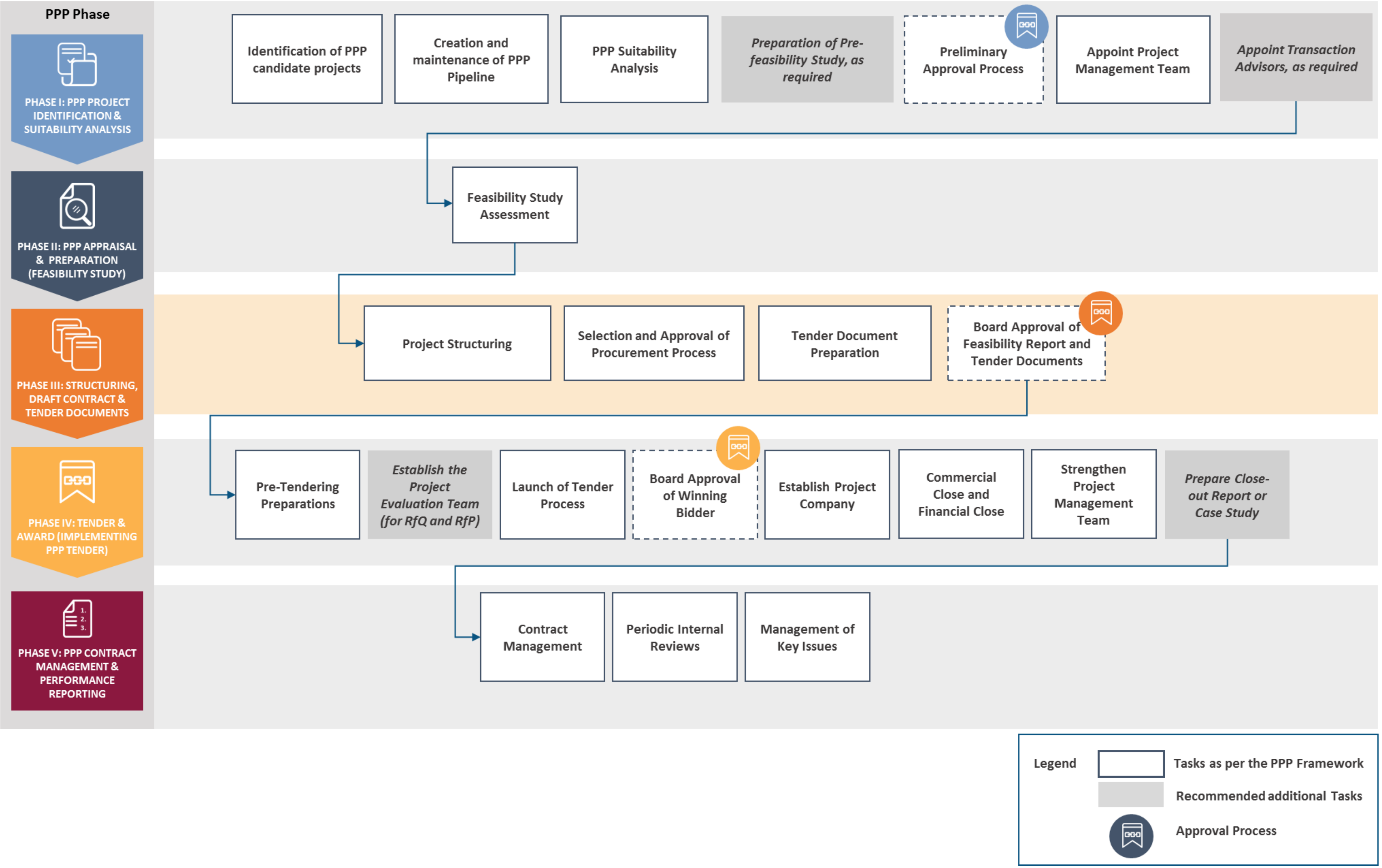

Figure 5-2: Phase III Process and Key Stakeholders

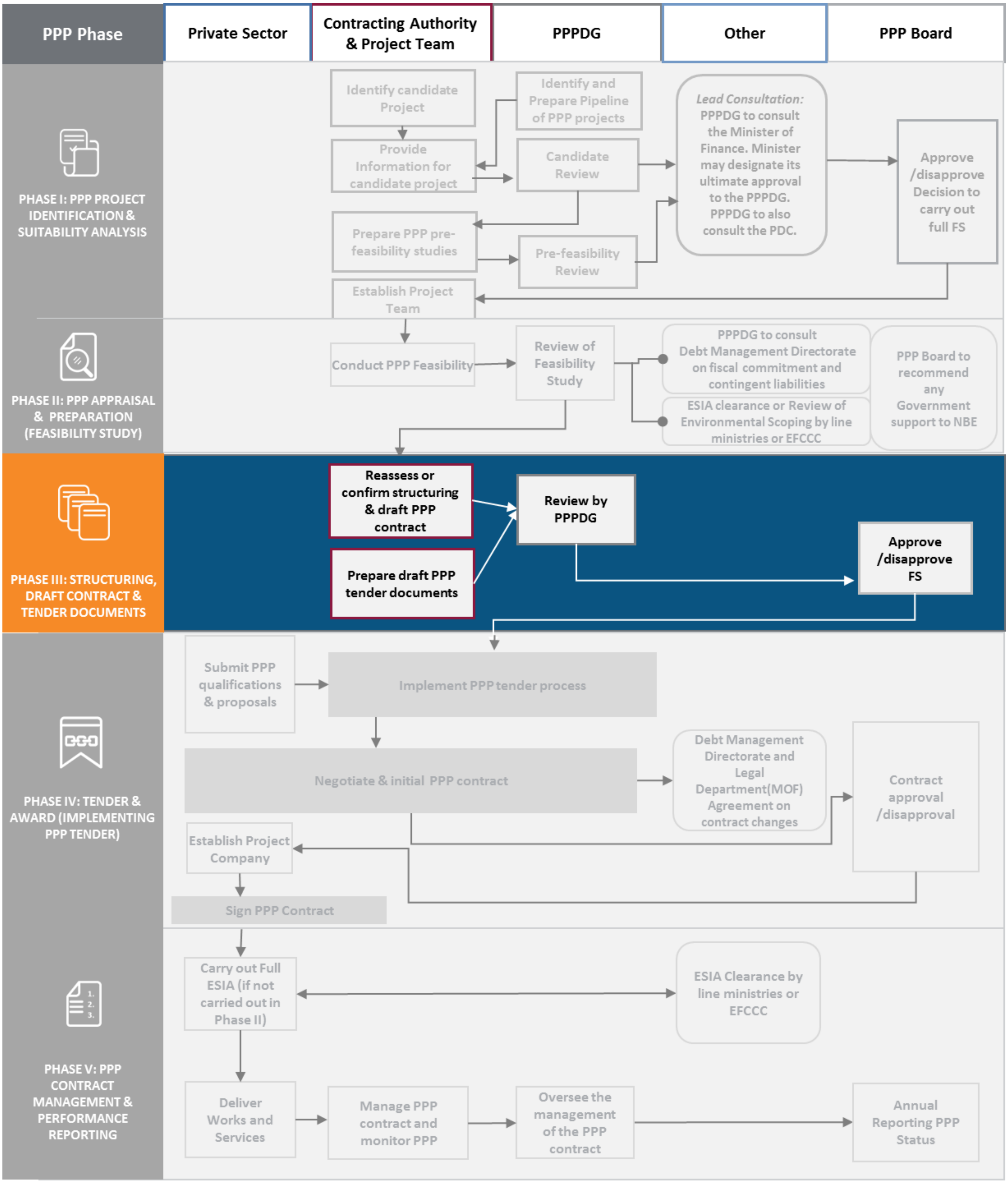

5.1 PROJECT STRUCTURING

Overview of Project Structuring In this phase of the PPP life cycle, the PPP project structure identified at the feasibility stage is stressed-tested to confirm final risk allocation and incorporate that allocation into the tender documents. This is a procedure that includes selecting the procurement model, designing the qualification and evaluation criteria and all the components of the tender process to suit the unique PPP project structure. While the project feasibility study demonstrates that a particular Public Service Activity can be feasibly procured as a PPP, the project structuring phase details the precise mechanics (policy decisions, project marketing and promotion, contract documents, selection process, payment mechanisms, guarantees and other risk mitigation mechanisms) that binds the feasibility outcomes into cohesive commercial relationships.

The primary output of the project structuring phase is the PPP Agreement and tender documents. The procedures under this phase are aimed at reconciling the assumptions and other building blocks of the project feasibility with specific PPP contract clauses. The procedures are concluded with a design of the procurement process, selection of tender process, design of the tender documents and schedule to efficiently procure the PPP in a manner that preserves Value for Money.

The Project Management Team is responsible for leading the project structuring activities. The Project Management Team may continue utilizing the services of the Transaction Advisor, if the advisor’s scope were defined to include support with project structuring following feasibility. The Key elements of the feasibility conclusions that are stressed-tested during the structuring phase include:

→ Project Specifications, Costing and Financial Structure

→ Payment Mechanisms and Instruments

→ Risk Allocation and Mitigation

5.1.1 PROJECT SPECIFICATIONS, COSTING AND FINANCIAL STRUCTURE

The Project Management Team must establish precisely the outcomes for the PPP project. During the feasibility stage, PPP project outcome can be varied to arrive at an optimal level that balances cost and affordability. During the structuring phase, the specific outcomes that will be associated with the PPP Agreement shall be identified so that other project parameters, technical solution, design and cost, service tariff and costs, any user fees and government contributions are determined and integrated into the PPP Agreement. Specific components of the feasibility that should be revisited are:

5.1.1.1 Technical Requirement

The parameters prescribe specifically the technical solution for the delivery of the Public Service Activity. For example, on an energy project, the technical solution could be defined as a 50 MW Solar IPP. The two primary reasons to have precision in the prescription of the technical solution are:

- To enable a direct comparison between the offers of two or more solutions providers

- To create an incentive for innovation and efficiency, as competitors strive to produce the same technical standard at the most efficient cost, thus generating Value for Money

5.1.1.2 Project Cost and Financial Structure

Following the definition of the technical solution, the efficient project cost shall be estimated. This cost is estimated by reviewing indicative costs prepared during the feasibility stage and validate those estimates using benchmarks and case studies to arrive at cost figures with a higher level of confidence. The Project Management Team's confidence level in the cost estimates shall influence the risk allocation in the PPP Agreement and as well the procurement method type selected for the process. For instance, if the project cost cannot be estimated with a high degree of confidence, and historical precedents or benchmarks show a wide range of estimates for the same solution, then the Project Management Team may want to recommend to the PPPDG that procurement for the technical solution may have to be carried out via a method such as Two-Stage Bidding, where Bidders can propose different solutions and costs allowing the PPPDG to select an efficient solution that best matches its needs.

The estimated project cost will impact the designed financial structuring of the project in the following ways:

- The amount of public sector contribution of support required. Public sector contribution would have to be approved by the Ministry of Finance and National Bank of Ethiopia and the precise amount appropriated by the legislature. Once the estimated project cost is estimated, this figure will need to be submitted to the PPP Board for approval. The PPP Board will consult with the Ministry of Finance and the National Bank of Ethiopia. Any specific government instruments (e.g. guarantees) that were approved as part of the Project Feasibility to support the project costs would need to be approved as well by the PPP Board.

- The PPP project duration may need to be revised to accommodate the final project cost.

Any changes to the project cost, PPP duration will necessarily have an impact on the service costs and an associated user fees. Thus, the project financial model will have to be updated to take into account the revised variables. If any of these variables or outputs from the financial model were subsequently tied to elements of the PPP structure, e.g. government payments subsidising user-fees, then those factors need to be updated.

The Project Management Team must reflect all these elements into the PPP Agreement. In making these changes, the Project Management Team must be mindful that the project Value for Money would have been impact with the changed project parameters. The Project Management Team must update the Value for Money to ensure that the PPP project as specified during the structuring phase still generates Value for Money.

5.1.1.3 Performance Requirements and Management

With the establishment of the project outcomes, the Project Management Team shall then define the minimum performance requirements along with the Key Performance Indicators to evaluate performance. This activity will presage the development of other performance related metrics in the PPP Agreement, including penalties for non-performance, events of default,

performance monitoring provisions, renegotiation conditions, early termination as well as other PPP Agreement parameters.

Establishing the technical parameters of the PPP project, will allow the Project Management Team to establish the final risk allocation for the proposed PPP.

5.1.2 RISK ALLOCATION AND STRUCTURING

The risk matrix developed as part of the feasibility study will have to be finalized and incorporated into the PPP Agreement as part of the project structure. Following the finalization of the project outcome specifications, technical solution and costing, the final risk allocation can be determined. If the risk allocation proposal is significantly different that the matrix identified during the feasibility and market tested, then the Project Management Team has to carry out another round of market sounding to gauge the perspective of investors and operators of the revised allocation.

The Project Management Team shall review the Risk Allocation prepared as part of the feasibility. Any new risks that has risen as a result of the final project specifications will be included into the matrix and assigned to the party best suited to manage that risk. In carrying out that task, the Project Management Team will also ensure that the PPP Agreement recognizes the assignment of that risk to a particular party.

5.1.3 PAYMENT MECHANISMS AND INSTRUMENTS

When the public sector is contributing to project costs or is responsible for making payments to the Private Party as part of the PPP, the PPP Agreement must specify the mechanism and structure of such payments. Decisions on this will depend on government policy and budgeting process. The Project Management Team must have a discussion with the Ministry of Finance with regards to how public sector contribution to a PPP will be structured (e.g. payment instrument [grants, loans, credits] and frequency of payments [milestone-based or time-based]). Agreements around these parameters shall be included in the PPP Agreement.

5.2 SELECTION AND APPROVAL OF TYPE OF PROCUREMENT PROCESS

For each PPP process, the Contracting Authority needs to select a procurement process to be adopted. Section 4.1.16 describes the procedures for deciding the optimal procurement strategy and bid process for a PPP. This procurement process – with associated steps and timelines – must be communicated in the Request for Prequalification and Request for Proposal. The PPP Proclamation[51] identifies four types of procurement processes.

Each type of procurement process is further elaborated under **Appendix D, Part 1: Background References – Phase III.**

[51] Chapters 5 to 9 of the PPP Proclamation

Figure 5-3: Selection and Approval of Type of Procurement Process

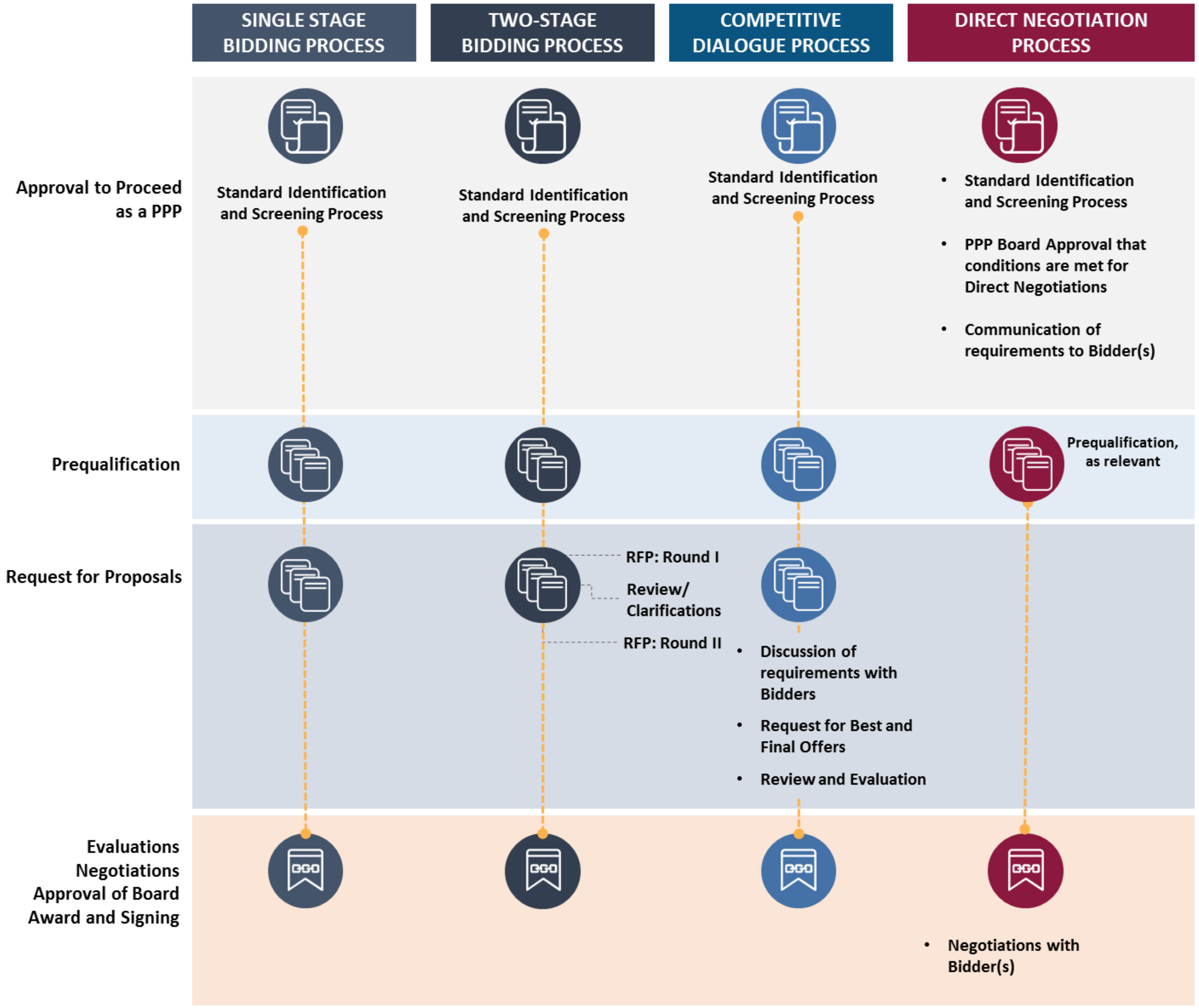

5.2.1 THE PPP AGREEMENT

The PPP Agreement will include clauses that are standard to many commercial agreements and others that will be specific to the PPP Project. In preparing the PPP Agreement, the Project Management Team, will be presenting the PPP relationships in legally binding commercial terms. This means for each risk assignment, responsibility and compensation mechanism is drafted into the agreement with clear accountability and measurement metrics and associated penalty mechanisms or other recourse for non-performance.

The PPP Agreement structure provides for specific sections for including standard clauses, specific clauses, schedules, annexes and forms extracted from Bidder's proposal submissions. On some PPPs, it may also be necessary to incorporate third party agreements and instruments, for instance a guarantee agreement with the government, as part of the suite of PPP Agreements. In such cases, the suite of agreements, the PPP Agreement Package, comprises the complete agreement for the PPP.

5.3 MANAGEMENT OF TENDER DOCUMENT PREPARATION

The PPP Proclamation authorizes the PPPDG to carry out the procurement of PPP projects.

Best practice recommends that Contracting Authorities, who will ultimately be the owners of a PPP Project must be involved in the project preparation. This is to ensure buy-in for the project concept and active contribution during the project appraisal and preparation phase.

The basic procurement stages shall be organized as: the Prequalification stage and Request for Proposal Stage. For complex PPP projects, where the PPPDG requires additional market consultations to establish private sector appetite, the PPPDG shall conduct Early Market Engagements prior to the prequalification to enable a more accurate targeting of the tender process.

For each stage, there is a specific set of tender documentation which must be prepared, alongside the corresponding Evaluation Criteria, Evaluation Guidelines and Scoring Sheets.

Figure 5-4: Procurement Stages and Documentation

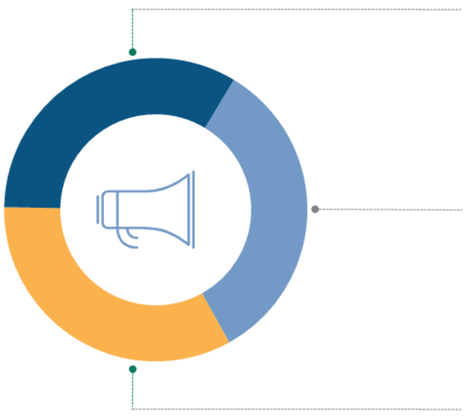

1 **EARLY MARKET ENGAGEMENT (Optional)**

- Draft General Procurement Notice for publication
- Draft a Request for an Expression of Interest (targeted market sounding questions to inform key project parameters/structuring)

2 **PREQUALIFICATION STAGE**

- Draft RFQ Notice for publication
- RFQ Evaluation Criteria, Evaluation Guidelines and Scoring Sheet
- Draft Evaluation Matrix and Evaluation Form

3 **REQUEST FOR PROPOSAL STAGE**

- Draft Request for Proposal (RFP)
- Prepare Technical and Financial Forms
- RFP Evaluation Criteria, Evaluation Guidelines and Scoring Sheet

5.3.1 PREPARATION OF EARLY MARKET ENGAGEMENT DOCUMENTS (OPTIONAL)

The early market engagement stage represents an optional step that public authorities may undertake prior to issuing a more detailed notice at the Prequalification Stage; it does not bind private firms to then submit actual PPP bids, nor can it be used by the PPPDG to disqualify an interested firm from participating in subsequent steps in the tendering process.

The General Procurement Notice is the PPP Project Pipeline. The PPPDG can decide to publicize it, and more specifically the list of projects approved by the PPP Board to proceed to feasibility study and to tender, in order to inform stakeholders of potential project that will be procured by the PPPDG.

Additionally the PPPDG can decide to prepare a Request for Expression of Interest (REOI). The REOI typically provides an early notification regarding a specific project, and includes basic project information such as: the Project Title, Project Sponsors, Project Background, and Procurement Method. The EOI also includes instructions on how to *register interest* to receive more information about the project/to receive the Request for Qualification (RFQ) notice upon the launch of the prequalification stage. Standard practice is to provide the name and contact information (email address) for prospective bidders to register their interest.

For large and complex PPPs, it may be beneficial to engage with prospective bidders. Requesting feedback from prospective bidders at this early stage can provide several benefits, among which are the following:

- Help the PPPDG refine the project structure arising from the Feasibility Study
- Help the PPPDG identify project or technology solutions for delivering the Public Service Activity as a PPP
- The Early Market Engagement is also an excellent opportunity for the PPPDG (i) to learn about important concerns that private bidders may have about the PPP project, (ii) identify potential sources for innovation and technologies available, and (iii) inform the key project parameters/project structuring of the PPP:
  - This may be carried out by launching a Request for Expression of Interest (REOI); an RFEOI provides a specific set of questions for prospective bidders to include in their response, within a specified timeframe.
  - Early market engagement activities may alternatively take the form of key stakeholder consultations with prospective service providers/operators, investors, etc.

To avoid the perception of bias or unfairly favouring a group of stakeholders, consultations during the Early Market Engagement shall be held publicly with free access to all willing participations. The PPPDG shall carry out the following for Early Market Engagement consultations:

1. Publish in a widely disseminated medium, the announcement of the PPPDG's intent to hold a public roundtable on the proposed PPP project

2. In the announcement the PPPDG will briefly describe the PPP project, focusing on the anticipated demand and the proposed risk allocation structure, without elaborating on any commercial parameters
3. In the publication, the PPPDG shall invite the general public, along with prospective investor, operators to participate in the roundtable discussion
4. The PPPDG shall emphasize in the announcement that participation in the roundtable will not disqualify prospective any candidate from participating in the Bid Process
5. An Aide Memoire of discussions during the roundtable will be made available on the website of the PPPDG (until the transaction reaches close), and will be part of the Data Room materials during the Bid Process

KEY CONCEPTS AND DEFINITIONS

The purpose of the Expression of Interest is to:

- Generate and promote interest in the PPP project amongst private service providers (as well as potential private suppliers, sub-contractors, and lenders);
- Allow the Contracting Authority to determine if there is sufficient interest amongst private bidders to proceed with the rest of the PPP tendering process. Without this procedure, the Contracting Authority could risk issuing an RFQ only to discover that there is too little interest from private bidders to proceed with the PPP tender.
- Alert serious private bidders that they should begin planning to make their best PPP analysts, designers, and other bid development specialists available to spend several weeks in the near future to prepare for the bid.
- Alert bidders, particularly for larger PPPs, to form their consortia of firms to bid on the opportunity.

5.3.2 PREPARATION OF THE REQUEST FOR QUALIFICATION (RFQ)

The Request for Qualification (RFQ) advert will be targeted to potential bidders for the PPP scheme. The advert will include a brief description of the project, an introduction to the project sponsors, technical requirements for submitting a bid, the bid submission address and date.

Generally, the information requested in an RFQ shall not require respondents to incur significant expenses in the preparation of their responses but shall be sufficient to allow informed evaluation by the PPPDG.

The objective of a qualification process is to set a minimum bar of capability for the bidder company or group of companies (bidding consortium) entering into the PPP contract. Setting qualification criteria will reduce the risk of project failure caused by a lack of capabilities and capacity. However, if the criteria are too restrictive this may limit competition too much, but too lax criteria may also discourage highly qualified bidders as they perceive they have a very low chance of winning. The aim is to achieve an appropriate balance, bearing in mind the project's specific needs.

> **KEY CONCEPTS AND DEFINITIONS**
>
> **Optional TOR and Information Memorandum (IM)**
>
> If available, the RFQ may also be accompanied by an 'Optional Terms of Reference,' to provide prospective bidders with more information on the scope of work. An Information Memorandum (IM) or abridged IM may also be prepared as a hook to attract more participants in the process. The IM may provide an overview of the key market data and relevant statistics, an overview of the transaction structure and process, and any commitments from the public sector.

Best practice requires that RFQ notices include information about how RFQ will be evaluated. Thus any invitation for prequalification circulated by the PPPDG shall generally contain the following information so as to be consistent with the PPP Proclamation and obtain valid results:

Basic Project Information

i. Name and brief description of the Contracting Authority

ii. Background with respect to the present state of the relevant service

iii. Objectives and overview of the project, drawing upon the Project Initiation, Screening

iv. Basic risk-allocation structure of PPP being considered (i.e., Lease, BOT, PFI, concession, etc.), including any public sector supports or risk-sharing mechanisms available;

v. Services that the private sector is expected to deliver (i.e. design, construction, technology, equipment installation, project management & oversight, maintenance, renewal & replacement of assets, etc.)

Qualification Criteria

vi. Required information that interested private bidders should submit, and any technical input and/or comments on the project;

▪ Description of the Technical Expertise being sought from the private sector	Explicit requirement

▪ Description of the Financial Capacity being sought from the private sector	in PPP Proclamation[52]

vii. Level of experience in the sector and region;

viii. Whether joint expressions of interest from consortia of private bidders is allowed or encouraged, and whether there are any local participation requirements (e.g. requiring all international bidders to team up with local firms);

ix. Required information about the private entity including the name, address, nature of the organization, relevant expertise and experience, technical input and comments on the project.

Indicative Schedule for Procurement

x. The Contracting Authority's planned future steps and communications with the interested private respondents (i.e. when the RFP are expected to be released);

A Sample **RFQ** is included in **Appendix D: Part 3.**

5.3.3 PREPARATION OF THE RFQ EVALUATION CRITERIA, GUIDELINES AND SCORING SHEET

The PPPDG shall prepare the RFQ evaluation criteria including thresholds for basic compliance, technical, financial and operational capacity – at RFQ stage the evaluation tends to be on a pass/fail basis – minimum requirements for qualification - but for more complex projects an appropriate scoring matrix can be used.

As mentioned above the aim is to produce a balanced response to the RFQ, too many bids can prove onerous at the RFP stage and act as a deterrent to some of the more serious bidders. A scoring matrix can be used to determine a ranking of responses and a specific shortlist of say the top 8 bidders for qualification. With a pass/fail process all bidders fulfilling the criteria would be qualified.

The pre-qualification evaluation criteria shall only shortlist candidates which demonstrate that they can manage and sustain the entire PPP process. This includes, at a minimum, the following:

- *Sufficient financial capacity to undertake the project, through evidence of net worth or other appropriate measure; and*
- *Sufficient technical capacity to undertake the project, through evidence previous appropriate experience.*

As per the Directive[53], the prequalification criteria shall include, at the minimum, the following:

[52] Article 21 of the PPP Proclamation
[53] Article 17(1) of the PPP Directive

Table 5-1: Prequalification Minimum Criteria

Minimum Criteria	Supporting Evidence
**Relevant experience and expertise:**<br>▪ Adequate professional and technical qualifications as necessary to carry out all the phases of the project<br><br>**Managerial and organizational capacity:**<br>▪ Appropriate managerial and organizational capability, reliability and experience, including previous experience in operating similar infrastructure facilities	▪ Experience of the proposer and record in delivering similar projects, including:<br>→ Minimum number of projects of more than a specified capacity developed/ constructed / operated<br>→ Minimum number of projects of more than a specified project value developed/ constructed/ operated<br>→ Minimum number of O&M contracts of similar facility<br>→ Any other criteria suitable for the need of the PPP project<br>▪ Capacity to deliver PPP in addition to existing commitments<br>▪ Proposed team of experts<br>▪ Experience of working in local conditions (critical if the local conditions have a material impact on the technical solution or operation of the proposed facility or service)<br>▪ Other mechanisms as are deemed appropriate by the PPPDG
**Financial capacity, net worth, net turnover, or other financial criteria deemed appropriate:**<br>▪ Sufficient ability to manage the financial aspects of the project and capability to sustain its financing requirements	▪ Net worth of the proposer<br>▪ Average annual turnover derived from audited financial statements<br>▪ Average net cash accrual derived from audited financial statements<br>▪ Capability of the proposer to invest the capital that would be needed for the PPP project<br>▪ Ability of the proposer to secure adequate funds for the PPP project<br>▪ Ability of the proposer to support the contractual arrangements over the contract term

As per the PPP Proclamation[54], bidders may only participate directly or indirectly in one consortium at a time. It shall therefore be clearly stated in the prequalification notice that if

[54] Article 22(2) of the PPP Proclamation

bidders are found to be involved directly/indirectly in one or more bids, they will be excluded from the procurement process. In order for the Project Evaluation Team to monitor this, the Lead Firm shall provide details of the proposed consortia and cross reference members to relevant submissions, including experience and financial capacity, and provide supporting **Letters of Association (LOA)** where the bidding entity is a consortia of firms; in the case of a joint venture, the management structure should be clearly defined. Letters of Association shall be required for both the prequalification and proposal stages.

Allowing flexibility on changes to consortia, provided that the lead members do not change, can be beneficial in projects in which the complete mix of skills and capabilities required may not be obvious until bidders see the detailed RFP. In these circumstances, a consortium may want to bring in specific companies when they see the RFP, or other consortium members may not want to continue when they see the RFP.

Estimated Time Required: The amount of time required to prepare the RFQ depends on the nature, the complexity, and especially the perceived risk exposure and allocation structure of the specific project. For the larger, more complex, or "riskier" PPP projects, this procedure may take up to 1-2 full weeks to complete. However, for smaller, simpler, or less risky projects, this procedure may only need 5 full days to complete.

5.3.4 PREPARATION OF THE REQUEST FOR PROPOSAL

The PPP Proclamation states that the *"PPP Directorate General shall prepare a Request for Proposal (RFP) for the purpose of inviting bidders to submit proposals."*[55] The Proclamation outlines the following minimum requirements to be included in the Request for Proposal:

- General information related to the project necessary for the preparation and submission of a proposal;
- Specifications of the project including the technical and financial conditions that should be met by a bidder;
- Specifications of the final product, level of services, performance indicators and such other requirements as the PPPDG and relevant regulatory bodies shall consider necessary, including the safety, security and environmental preservations requirements to be met by a bidder;
- The contractual terms of the Project Agreement proposed by the PPPDG;
- The criteria and method to be used in evaluating a proposal;
- The value of the bid security required to be submitted by a bidder; and
- The manner and deadline for submitting proposals.

> **BEST PRACTICE TIP**
>
> **TIMING**
>
> Where possible, it is best for the relevant procurement documents for the Request for Proposal (RFP) Phase to be prepared prior to the expiration of the RFQ process.

[55] Article 24(1) of the PPP Proclamation

The Request for Proposal document shall include all the information needed by qualified bidders to submit full and detailed bids for a PPP project to the PPPDG. This includes instructions to bidders for how to present their bids, as well as other relevant background information about the project, including which documentation will be made available in the **Data Room(s) [further discussed in Chapter 6]** – such as technical, economic, financial, legal, environmental and other feasibility analyses, as well as the detailed PPP risk allocation structure of the project.

These also include instructions to bidders about the required formats in which their bids must be submitted and other requirements with which they must comply. These required formats generally encompass the following:

- Cover Letter for the Technical Proposal Submission
- Technical Proposal Forms
- Cover Letter for the Financial Proposal Submission
- Financial Proposal Forms

The preparation of the Request for Proposal represents the culmination of the Feasibility Study and initial Proposal Appraisal activities. The RFP document must be very clear about the minimum output performance requirements of the project; the allocation of key risks between the parties, and the rules by which bidders must present and submit their bids and the evaluation criteria against which those bids will be reviewed.

Without this important step, private bidders would likely submit very different technical and financial bids to the Government, proposing different levels of performance, assuming different allocations of project risks, and featuring different proposed prices. International experience has shown that while it can take a significant amount of time for the Contracting Authority to prepare clear, detailed, and good-quality PPP bid documents, it generally takes even longer to have to respond to numerous requests for clarification or protests from bidders, and to evaluate vastly different PPP bids each based upon differing risk-allocation assumptions.

Best practice suggests the following general structure for the RFP documents:

1. Cover Note in the form of an Invitation to Bidder, providing a brief background and justification for the PPP project, an introduction to the Contracting Authority and the expectations from the private partner;

2. General information required by bidders to prepare and submit their proposals. This will provide overall instructions to bidders regarding the format and organization of the information they submit;

3. PPP Project specific information and performance indicators including the Contracting Authority's standards regarding safety, security, environmental protection, etc.;

4. PPP bid documents shall include not only a detailed risk allocation matrix and description of the PPP modality, but also a complete draft of the full PPP Contract that the preferred bidder will be expected to sign and fulfill. This draft contract shall be complete and sufficiently detailed such that there will be very little the remains to be negotiated once the preferred bidder selection has been announced.

5. The criteria against which proposals will be evaluated, including minimum benchmarks that proposals must meet in order to be deemed responsive.

The RFP document will also include a section on fraud and corruption. More specifically, the RFP will clearly indicate that collusion between bidders is prohibited. Indeed, the PPPDG will not select a Bidder to enter into a PPP Agreement if it determines that the Bidder has, either directly or indirectly, colluded or cooperated with any other Bidder in the preparation of any Proposal except any party that is in the same Bidder consortium. Any Bidder the PPPDG determines has been engaged in such practice will be excluded from the Transaction Process. Similarly, any effort by a Bidder to influence Government officials or other stakeholders involved in the procurement process will result in the rejection of the Bidder's Proposal.

The RFP should include a complete list of documents to be submitted by bidders. This can include:

Technical proposal:

- Preliminary design (not detailed engineering design)
- Construction programme and costs
- Operating programme and costs
- Maintenance programme and costs
- Environmental protection plan

Financial proposal:

- Cash flow projections: although usually not directly used in the evaluation, such projections can show whether the bidder has used reasonable assumptions in the preparation of its bid
- Formal bid: proposed tariff, payment to contracting authority or requested amount of subsidy (as appropriate)

Legal proposal:

- Acceptance of terms of the contract.
- Draft shareholders' agreement, consortium agreement, joint venture agreement
- Letter of conveyance signed by the authorised representatives of the company or consortium submitting the bid
- Term sheets of other main contracts could also be requested (construction contract, operation and maintenance contract, insurance, etc.)

Draft PPP Contract:

- Bidders may be required to submit a signed copy of the draft PPP Contract as part of their bid documents in order to confirm their agreement to the terms of the contract and limit post- selection negotiation to a minimum.

To facilitate the understanding of the requirements by bidders and maximise the chances of all proposals received being fully compliant, it is essential that this list be clear and exhaustive, and that no additional requirements be added in other sections of the RfP document or Bidding Documentation Package.

Technical and Financial Evaluation Criteria

Technical Criteria and evaluation

In accordance with the principle of transparency, and as a mechanism for helping bidders tailor their proposals to the contracting authority's needs and goals, the criteria for the evaluation of bids, their relative weighting and the way they will be evaluated should all be clearly indicated in the RfP.

Typical qualitative and/or technical criteria include[56]:

- Construction matters, which may include criteria such as quality and reliability of the project design offered (usually under a pre-design format), the reliability of the construction period estimated, the quality assurance methods proposed for construction oversight, and so on.
- Operational matters, which may include criteria such as quality and reliability of operating procedures and manuals, commitment of means, service or O&M quality management systems or plans, and so on.
- Maintenance matters, including criteria such as quality and reliability of the maintenance plans and programs, renewal/major maintenance programs, specific plan for hand-back, and so on.
- Evaluation criteria for environmental compliance and environmental sustainability (for example, landscape factors)
- Evaluation criteria on health and safety plans
- Other qualitative criteria such as criteria related to benefits for minority or disadvantaged populations (usually set as a minimum bar or condition, for example, the number of members of the minority community provided with employment by the project or the consortium), financing reliability, and so on.

The list of criteria and sub-criteria should not be so large as to create undue complexity in the evaluation and make it difficult for bidders to focus on the fundamental objectives of the government.

The technical requirements in the RFP should not be too prescriptive as PPPs focus on performance and PPP specifications are mostly based on outputs rather than inputs. The specifications should provide only a reference design in a pre-design form or a "functional design", with the service requirements focusing on the results/quality of the service through key performance indicators (KPIs), rather than the amount of inputs or activities.

Financial criteria and evaluation:

The design of the criteria for the financial evaluation depends to a great extent on the characteristics of project and what the objectives are. Is this a project where for example, the government wants to maximise a concession/service fee, minimize subsidy profiles,

[56] APMG PPP Certification Guide Section Ch 5 s 8 .2 Evaluation Criteria

upfront grants, availability payments or minimize tariffs for the users? Other objectives like fastest completion, best quality of service, shortest duration of the PPP contract, can also impact on the value of the financial evaluation.

In accordance with the principle of transparency, and as a mechanism for helping bidders tailor their proposals to the procuring authority's needs and goals, the criteria for the evaluation of bids, their relative weighting and the way they will be evaluated should all be clearly indicated in the RfP. As a general rule the criteria should be kept as minimal and objective as possible and the general system should remain simple and clear.

In processes with staged evaluation (technical pass/fail then financial), it can be difficult to manage the evaluation of the financial proposal without information on the reliability and robustness of the financial structure that would allow the evaluator to know in advance or infer the price offered. Strict instructions should be provided to bidders, emphasizing that the financial information in the technical proposal should not disclose the overall price offered.

The financial package is sometimes subject to evaluation in terms of reliability, such as the commitment level shown by the equity investor, the level of confidence in the financing availability, and the degree of robustness of the project finance structure.

For the financial proposal evaluation, when only one criterion exists, usually the price (e.g. average tariff), the royalty paid, the level of subsidy, the NPV, or the contract term, the winning bidder corresponds straightforwardly to the bid which presents the lowest price, minimum subsidy, highest value of royalty or net present value (NPV). However, when there are several criteria, the situation is more complicated, and it may be necessary to adopt a multi-criteria decision analysis to choose the winner. In this case, the awarding authority should clearly define the criteria (and eventually sub criteria) and the bid evaluation methodology.

Even when a single criteria is determined, the evaluation may present issues for example, with a minimum tariff competition, two bidders could propose the same tariff the two bidders present very different profiles e.g. one of them demands an equity internal rate of return of 20% and the other requires only 10%. Evaluators may need to refer back to the technical scoring and the sustainability of each of the bids to determine the clear winner.

<u>Local content:</u> A preference margin of up to 7% of bonus may be allocated to the financial proposal when the capital share of the domestic party is more than 50% and the following conditions are fulfilled:

- → for a project involving a construction phase, if at least 30% of the raw materials used are from Ethiopia and 50% of the employees are Ethiopians;
- → when 50% of Ethiopians are involved in the project/company management
- → for a project that doesn't involve a construction phase, the request for proposals shall describe the thresholds that will determine if a bid can benefit from a preference margin

BEST PRACTICE

Practical Tips for Setting Financial Evaluation Criteria

Avoid too many financial parameters: Wherever possible, the contracting authority should avoid having multiple financial parameters as bidding criteria (for example, an upfront fee, a fixed fee and a variable fee). Multiple financial parameters provide bidders with the possibility of arbitrage between the various cash flows, and may ultimately produce unexpected results for the contracting authority. For example, having bidders submit both a completion payment and an availability payment may incentivise one bidder to propose a low completion payment and a high availability payment and another bidder the opposite, depending on how the discount rate that is used for evaluation relates to the cost of capital for individual bidders. The challenge is how to compare the two bids, considering that the risk profile for the contracting authority varies between the two bids. Therefore, it is preferable to set most of the parameters and leave only one parameter open for bidding. Having only one financial parameter will also greatly simplify the bid evaluation process. However, this may not always be feasible, given the nature of the PPP project, the characteristics of the financial flows and the objectives of the government.

Watch out for financial bids based on demand assumptions: Anyone can promise high growth and therefore high payments to a contracting authority in a financial bid. If demand risk is transferred to the private party in the PPP structure, the contracting authority should ensure that it does not have to take a view on the extent to which the demand assumptions used by bidders are realistic. The contracting authority should therefore avoid creating bid parameters that depend on demand assumptions, as this is akin to "awarding the bid on a promise". Alternative approaches are 1) let bidders guarantee all or part of their own revenue projections as a means of "keeping them honest" and 2) using a weighted average of government demand scenario and bidders demand scenarios (in which case the bidder demand scenario would still need to be wholly or partially guaranteed).

Make the financial evaluation transparent and predictable: If there is one single financial criterion and it is not variable in time – for example an upfront payment or a fixed periodic fee – there may not be a need to evaluate based on Net Present Value (NPV). In that case, it is recommended to keep the evaluation simple. If there are multiple financial criteria, an evaluation on the basis of NPV of the various financial flows is required. In that case, the contracting authority should make explicit the discount rate to be used as a fixed parameter by all bidders, and possibly also the demand scenario, in order to ensure that the bids will be comparable. Some contracting authorities share the financial bid model with the bidders, which creates predictability and reduces the risk of confusion over submission and evaluation. This obviously puts the onus on the contracting authority to provide a clear and appropriate financial bid model, but is clearly a best practice, particularly in the case of more complicated financial evaluations.

Expect a wide disparity in bids, even negative bids: In some cases, the contracting authority may be (positively or negatively) surprised by the financial proposals received from bidders. It may be offered a payment from bidders where it had expected to make one, or vice versa (for example, a negative subsidy or negative upfront payment). It is important for the contracting authority to envisage all possible scenarios, even extreme ones, and check whether the financial evaluation system can accommodate them. Especially in the case of extremely low (or high) bids, a proper assessment of the viability of the proposal is recommended.

Source: WB Caribbean PPP Toolkit

A Sample RFP is included in **Appendix D: Part 5.**

Estimated Time Required: The amount of time required to complete this procedure depends on the nature, the complexity, and especially the perceived risk exposure and allocation structure of the specific project. For the larger, more complex, or "riskier" PPP projects, this procedure may take up to 6-12 weeks to complete. However, for smaller, simpler, or less risky projects, this procedure may only need 3-6 weeks to complete.

5.3.5 BID BOND REQUIREMENT

Running a high-quality procurement process for a PPP results in high transaction costs for the contracting authority. There is a risk that bidders will provide insufficiently committed proposals, which can lead to difficult negotiations or delays after the selection of the preferred bidder, and even cancellation of a tender. This will result in even higher costs for the contracting authority, if there is a re-bid. To get more commitment from bidders and prevent the winning bidder from withdrawing without good cause, contracting authorities require bidders to provide a bid bond.

The Request for Proposal document shall include Technical Forms presenting in precise form the wording for any performance bonds or guarantees to be submitted by bidders. The instruction to bidders will state that any bidder who deviates from the provided language will be disqualified from the bidding process. Bidders will have the opportunity to provide comments or request clarification on the language of bonds and any guarantee documents provided in the RFP. If bidders do not request any changes, the PPPDG can assume that bidders have found the language appropriate.

The benefit of this approach is twofold:

- The PPPDG and Contracting Authority has confidence in the enforceability of the bond or guarantee instrument, based on the language in the document
- Verification of the bond or guarantee will only require verification of the underwriting institution providing the instrument

The need for bonds varies during the process and any delays may require extensions to be requested from the bidders to maintain validity over the revised tender period. As a rule of thumb the sizing of the bid bond should reflect the expected cost of running the tender (and potentially additional cost of delay of the project). Bid bonds are usually one to three per cent of the expected contract value. The contracting authority however should listen to feedback from bidders on the size of the bid bond; an unreasonably high bond will deter bidders. Also, bid bonds should be issued only by specified or qualified financial institutions, under the exact template and wording as provided by the contracting authority in the RFP.

Figure 5-5: Bid process – Bid Bond Management

**RFP Stage**	• Bid submissions – must include valid bid bonds (in prescribed format, on a recognized institution and for the correct validity duration)
**Award**	• Convert bid bond to Preferred Bidder bond (as SPV not consortia head) for the duration of the process to financial close (Reserve bidder to increase bid bond validity date if required)
**Commercial to Financial Close**	• Set up Performance Bond prior to Financial close (Condition Precedent)

The bonds will initially be given by the lead consortium member but upon award/preferred bidder announcement, it will have to be provided by the newly formed SPV to cover the finalization process from award, commercial and financial close.

5.3.6 CONTRACT GUIDANCE

The PPP Contract document provides the legally binding agreement allocating risks, responsibilities and roles on a PPP project.

The agreement's purpose is to ensure all parties to the partnership, including the Contracting Authority, private investor and their lenders, end users and other stakeholders have a clear and enforceable understanding of the risks and responsibilities on the project.

BEST PRACTICE TIP

Early Engagement with the Ministry of Finance Legal Directorate

The process of obtaining approval on the Draft PPP Agreement tends to be expedited with the early engagement of the relevant regulatory authorities, for preliminary review and comments. The PPPDG should ensure to coordinate with the Contracting Authority and the Ministry of Finance's Legal Directorate in order to secure comments on the Draft PPP Agreement.

The Contracting Authority and its PMT, with the support of the PPPDG, are responsible for preparing the PPP Contract document. The Transaction Advisor's Scope of Work shall include a requirement to develop a PPP Agreement that is consistent with best practice. The Contracting Authority may provide a template agreement for use by the Transaction Advisor.

The principal sections of PPP agreements include:

1	Definitions and Interpretations	16	Political Event
2	Contract Effectiveness	17	Termination
3	PPP Term	18	Compensation on Termination
4	PPP fees	19	Handback
5	Fees, Rent and licenses	20	Assignment of Agreement
6	Condition Precedent	21	Substitution of Grantee
7	Obligations of the Grantor	22	Grantor Step-in Rights
8	Obligations of the Grantee	23	Dispute Resolution
9	Representations & Warranties	24	Governing Law
10	Liabilities & Indemnities	25	Change of Law
11	Performance Security	26	Waiver of Immunity
12	Insurance	27	Renewal or Extension of PPP Term
13	Force Majeure	28	Long Stop Date
14	Events of Default	29	Amendment
15	Renegotiation		

For many PPP transactions, the contents of the contracts themselves are relatively brief and focus only on legal definitions and general allocations of risk. The important technical details of the PPP contract, such as language specifying the important output levels of service required are included in "Schedules" annexed to the contract. Other earmarked sections of the contract, such as "Long Stop Date", or Nominated Representatives of the Grantor or Grantee" will be left blank until agreement signing.

The contents of the contract agreement dealing with legal definitions and general allocations of risks are built from the relevant legal and regulatory framework in the country and specific sectors/sub-sectors where the PPP is being implemented. The next table presents a description of a longer list of general clauses common that are standard in most PPP agreement and other

clauses that are defined unique for the PPP. The purpose for each of these clauses is also elaborated in table.

Table 5-2: PPP Agreement Heads of Terms

PPP Agreement Heads of Terms	Description	Purpose
**General clauses common to PPP Agreements**		
Definitions and Interpretations	Introduces the meaning of specific words and phrases in the context of the PPP agreement	A shared understanding of words and phrases among the parties to the agreement
Contract Effectiveness	States the date and/or conditions after which the PPP agreement and its obligations are binding on the parties	Useful for evaluating parties' performance of obligations under the agreement
PPP Term	Specifies the duration of the PPP agreement with a reference to the trigger for its commencement	To establish the start and end date for the PPP duration
PPP fees	Specifies any regulatory fees and indirect costs for the PPP	To provide clarity for regulatory agencies and third parties to the PPP agreement
Fees, Rent and licenses	Specifies any utility charges and fees associated with obtaining permits or other authorizations	To provide clarity for regulatory agencies and third parties to the PPP agreement
Condition Precedent	Describes milestones that must be achieved before the PPP agreement becomes effective	To enable parties demonstrate ability to meet obligations in the agreement
Obligations of the Grantor	Describes the roles of the Grantor in the PPP relationship	A shared understanding of the role of the Grantor
Obligations of the Grantee	Describes the roles of the Grantee in the PPP relationship	A shared understanding of the role of the Grantee
Representations & Warranties	Presents a statement attesting to the good standing and eligibility of the contracting parties to enter into agreement	To validate both parties claim to eligibility to sign the PPP agreement
Liabilities & Indemnities	Establishes, if applicable, the limits of liabilities of the parties to the agreement to each other	To provide legal protection/enforcement of penalties for performance from the agreement
Performance Security	Introduces the financial bond posted by the Grantee to underwrite its performance obligations in the PPP agreement	An incentive for the Grantee's performance and punitive measure for non-performance
Insurance	Introduces the financial product to protect against specific losses in the PPP agreement	To provide legal protection for unanticipated events in the performance of the agreement
Technical Changes	Describes the process for making technical changes	Provide clarity for technical changes
Force Majeure	Introduces the concept of some unforeseeable event that may prevent either party to the agreement from performing	To ring-fence unanticipated and unpreventable events that affect performance
Events of Default	Identifies specific actions that are considered a breach of obligation of the PPP relationship	Removes ambiguity over actions and/or inaction that if uncorrected will lead to termination
Renegotiation	Describes the process for the parties to renegotiation in good faith elements of the PPP Agreement	Provides a mechanism for renegotiation unanticipated and unforeseen events in the PPP Term
Political Event	Introduces a category of actions that are political in nature and have an impact on performance under the PPP agreement	To ring-fence events that affect performance that are the result of public sector action
Termination	Introduces a mechanism for the early cessation of the PPP agreement	To allow the early discontinuation of the PPP agreement for any number of reasons
Compensation on Termination	Introduces the mechanism for assessing fair compensation to all parties in the event of early cessation of the PPP term	To provide certainty on liabilities following an unplanned early cessation of the PPP relationship
Handback	Describes the process for the cessation of assigned rights to provide service OR return of public asset from the Grantee to the Grantor	Provide an orderly transfer of public asset or service back to Grantor at end of PPP term
Assignment of Agreement	Establishes the permissions for delegating roles and responsibilities in the PPP agreement to third parties	To enable Grantor exercise oversight on performance of obligations
Substitution of Grantee	Establishes the permissions for replacing the Grantee in the PPP agreement	Enable Grantor maintain the continued performance of public service under PPP regime
Grantor Step-in Rights	Introduces the conditions under which the Grantor can assume the roles of the Grantee and the mechanism for this assumption	Enable Grantor maintain the continued performance of public service under PPP regime
Dispute Resolution	Establishes the protocols for addressing conflicts between the Grantor and Grantee	Provide an orderly process for reviewing and resolving conflicts
Governing Law	Identifies the legal framework providing the enabling authority for the PPP agreement	Provide clarity on legal precedents and procedures agreement interpretation
Change of Law	Describes situations where changes in the enabling legal framework for the PPP agreement affects the ability of the parties to perform	A framework for interpreting events directly caused by changed legal frameworks
Waiver of Immunity	Establishes the forfeiture of any rights to exemption from any form of prosecution for acts performed in relation to the PPP agreement	To eliminate protections from the rightful exercise of the Grantor's rights under the agreement
Renewal or Extension of PPP Term	Provides a mechanism for the lengthening of the PPP Term	To provide certainty for parties under the agreement and other third parties on the PPP term
Long Stop Date	Provides a fixed calendar date which is the last day for the PPP agreement to come into effectiveness	Provide a fixed end period before which parties to the agreement can fulfil condition precedents
Documentation and Audit	Outlines specific templates and processes for regular reporting by the Grantee to Grantor and other regulatory bodies	To fulfil regulatory and other compliance requirements and enable Grantor oversight
Amendment	Describes the process for making amendments to the PPP Agreement	Allows changes to the PPP relationship while maintaining the overall structure of the Agreement
**Other Clauses customized to PPP agreements**		
Object of the Concession	Summarizes the specific rights and obligations assigned by the Grantor to the Grantee as part of the PPP relationship	Should match the public sector objective of PPP and clarifies expectations
Construction Works	References relevant Schedules dealing with capital expenditure works to be carried out by Grantee	Reinforces expectations of Grantee in the PPP relationship
Acquisition Program	References relevant Schedules dealing with capital expenditure items to be purchased by Grantee as part of PPP	Reinforces expectations of Grantee in the PPP relationship
Use of the PPP Asset	Defines the specific uses to which any public asset part of the PPP relationship can be put	Removes ambiguity for non-permitted uses of the public asset

5.3.7 SIGNIFICANT STANDARD AND SPECIFIC CLAUSES

While many of the clauses identified in the long list in the table above are standard, their application in PPP have varied with the increasing sophistication of and variation of PPP schemes. A template PPP Contract, attached in **Appendix D – Part 6**, presents a sample PPP agreement with full legal definition for the clauses identified in the long list above. Please refer to **Sector Specific Guidelines** in **Appendix F** for variation on the language for some of these clauses.

On individual PPP projects, the Contracting Authority will mandate the Transaction Advisor d to review and update a PPP Agreement to be consistent with current best practices. The Contracting Authority shall use the table above as a guide to ensure all relevant clauses allocating risks and responsibilities in the PPP relationship have been elaborated in the agreement.

5.3.8 AGREEING ON LANGUAGE FOR GENERAL CLAUSES

While the general clauses to the PPP Agreement are typically standard and change very little between PPP projects. It is best practice to allow prospective PPP proponents to review the general conditions and satisfy themselves and their financiers/lenders on the language of the general clauses.

The best practice procedure is to share the Draft PPP Agreement with prospective bidders during the tender phase of the PPP. Prospective Bidders shall be invited to provide their comments on the language of the general conditions and identify language that raises concerns. The Contracting Authority and its Transaction Advisor would then be able to review language and determine if changes are warranted. Any change in the PPP Agreement following the Approval at the Feasibility Stage by the PPP Board would need to be re-submitted to the PPP Board for approval. Any changes in the PPP Agreement approved by the PPP Board will have to be reflected in the PPP Project Pipeline maintained by the PPPDG.

5.3.9 PPP SECURITY PACKAGES

Larger PPPs typically require a collection of agreements in addition to the PPP Agreement. The Security Package – the collection of agreements between a number of different parties – refers to this collection of agreements often necessary to make a PPP project bankable. An important distinction shall be made between the contracts between the Contracting Authority and the private service, and other required contracts that the Government is not a party to, such as between the private party and its commercial lenders or suppliers.

PPP security packages also feature a number of other contracts to which the public sector is not a party. While it is not the responsibility of the Contracting Authority to draft or negotiate these agreements, it is recommended that they be aware of the need for these contracts, their general contents, and their role in ensuring the overall security package provides a bankable and sustainable PPP project.

This is particularly relevant with respect to aspects such as lenders' step-in rights. The PPP legal framework allows for instances where a PPP project's lenders can step-in to substitute a Private Party on a PPP contract in instances of a serious breach of the obligations that could justify termination. Such other agreements may include:

Table 5-3: Indicative List of Agreements

Agreement Name	Parties to the Agreement	Purpose of the Agreement
Memorandum of Association and Article of Association	Private Shareholders of the PPP Company	Outline rights and responsibilities among the private shareholders to the PPP Company
Financing Agreement	PPP Company and Lenders	Provide the terms and conditions for the lending of funds to the PPP Company
Inter-Credit Agreement	Among lenders to the PPP Company	Agreement among lenders to provide funds under common terms to the PPP Company
Design-Build Contracts	PPP Company and Construction Engineering Company	Agreement for the design and construction of a facility for the PPP
Equipment Supply Contracts	PPP Company and Equipment Suppliers	Agreement for the long term supply of equipment to the PPP Company
Operations and Maintenance Contracts	PPP Company and Specialized O&M Company	Agreement to provide specialised operations and maintenance services to PPP Company
Insurance contracts	PPP Company and Financial Services Company	Agreement for the provision of financial services products to the PPP Company
Off-take Agreements	PPP Company and Public/Private Entity as long term buyer	Agreement for the purchase over a fixed term services provided by PPP Company
Implementation Agreement	PPP Company and MoF as awarding body	Agreement to facilitate and carry out specific tasks that enable the main PPP agreement
Put Call Option Agreement	PPP Company and Government Party	Gives Private Party rights to sell shares at specified price in Project to Government Party
Partial Risk Guarantee	PPP Company, Development Finance Institution and Government	Covers Private Party against government actions or failure to perform its obligations in PPP Contract as well as other political risks
Raw Inputs or Supply Agreement	PPP Company and Public/Private Entity as supplier	Agreement to provide raw feedstock to the PPP Company over a fixed term
Rolling Stock/Inventory Sale Agreement	PPP Company and Public/Private Entity	Agreement to sale or lease public assets to the PPP Company at the start of a PPP term
Limited Guarantees By Government	PPP Company and Public Entity	Agreement with public sector to provide guarantees over a term of the PPP Agreement
Direct Agreement	PPP Company, Lenders and the Government	Agreement between the lender and the Government. Step-in rights and other lender rights are often reinforced or established through direct agreements.

Figure 5-6: Basic Scheme of a PPP Contract Structure

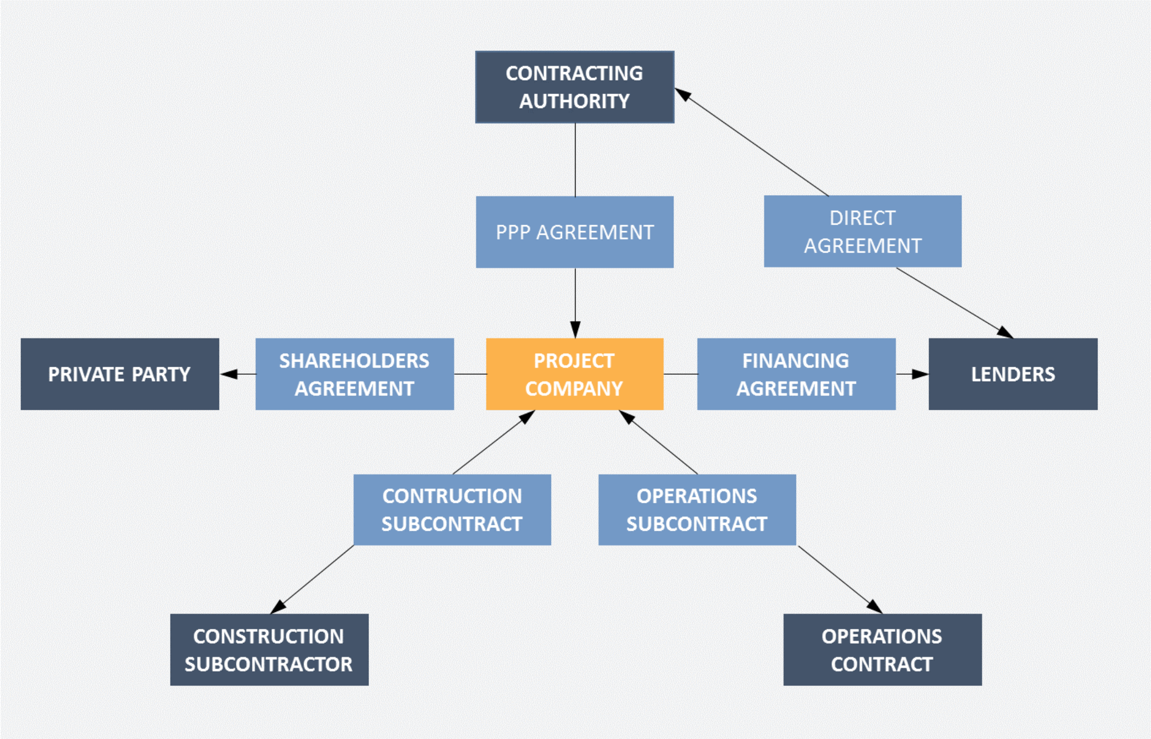

5.3.10 DRAFTING THE PPP AGREEMENT

A Draft PPP Agreement shall be one of the deliverables assigned to the Advisor in the Project Feasibility Scope of Work. The full suit of the Draft PPP Agreement will include:

- The Draft PPP Agreement
- Template Schedules to be populated with the technical and commercial specifications from the winning PPP proponent
- Any other ancillary agreements specified in the Advisor's Scope of Work

One useful way to understand the difference between the contents of the Contract itself and the contents of its attached Technical Schedules, is that the specific data and inputs of the Technical Schedules, which may include payment levels and number specifying levels of service – may be subject to some adjustments during the life of the contract as conditions like inflation, population growth, GDP growth, world energy prices, foreign exchange rates change and even fluctuate over the 10+ year terms of many PPPs. However, the legal definition of the rights and obligations of the parties, which is defined in the body of the PPP contract language itself, generally should not change.

A requested change to a number included in a schedule generally constitutes an adjustment in costs and prices, which is not uncommon during the life of long-term PPP contracts and for which there shall be clear formulas and procedures in the contract to handles these events. However, a request to change the language in the body of a PPP contract, constitutes a more serious renegotiation of the contract – usually an indication that the overall project's sustainability is under threat and an important restructuring and redefinition of the basic allocation of risks is needed.

Additionally, it is to be noted that there should be consistency between the different provisions of the agreements being drafted. Indeed a special emphasis should be put on the need to coordinates effort in order to have coherent, consistent and compatible provisions between the different agreements. Similarly the appropriate provisions of the PPP agreement (Private Party and Contracting Authority) should "flow down" to sub-contracting agreements (Private Party which is the prime contractor and Sub-contractors).

Estimated Time Required: The amount of time required to complete this procedure depends on the nature, the complexity, and especially the perceived risk exposure and allocation structure of the specific project. For the larger, more complex, or "riskier" PPP projects, this procedure may take 3 months or more to draft, review, and approve. However, for smaller, simpler, or less risky projects, this procedure may only need only 1-2 months to complete. The time required can be significantly shortened if a model PPP contract is available. It should be noted that as with several other procedures, this procedure may be carried out concurrently with other procedures.

5.4 BOARD APPROVAL OF FEASIBILITY REPORT AND TENDER DOCUMENTS

5.4.1 GUIDANCE ON SUBMISSION AND REVIEW OF APPRAISAL DOCUMENTS

The Contracting Authority and the Project Management Team will submit the feasibility study and tender documents they have prepared along with a list of required approvals from Public Entities that would be necessary prior to signing of the Project Agreements to the PPP Board.

The PPPDG as secretariat of the PPP Board will review the feasibility study and provide its analysis to the PPP Board.

The PPPDG will evaluate the feasibility study in coordination with the Ministry of Finance and the National Bank of Ethiopia. The MOF Debt Management Directorate will review the impact of the project in terms of contingent liabilities, fiscal affordability and budgetary impact whereas the National Bank of Ethiopia will evaluate the requirements in terms of guarantees and foreign currency. The Debt Management Directorate will advise on the ability or otherwise of the Government to sustain the Fiscal Commitments if any, that may be occasioned by the PPP Project or activities directly related to the PPP Project including both direct and contingent liabilities on Government's finances including guarantees arising from each PPP Project.

Following this analysis, the PPPDG will prepare an evaluation report, to be submitted to the board which will include the following elements as defined in the PPP Directive[57]:

- Explanation on whether the feasibility study is accompanied with relevant documents and proof that the project can be implemented as PPP. More specifically the evaluation report will cover the following points:

[57] 57 Article 14(2) of the PPP Directive

- → Overall quality and completeness of the Project information
- → Description and analysis of the key strengths of the project as a PPP
- → Description and analysis of the key risks and constraints of the project as a PPP
- → Initial identification of options for how these risks and constraints might be successfully resolved in subsequent analyses and structuring of the project as a PPP
- → Summary of Value for Money and Affordability analysis
- → Description of potential Government support/commitments

- As per the specific project, state the appropriate mode of private party selection or put evidence-based selection plan
- Major criteria to select private party and when appropriate including project agreements terms

5.4.2 GUIDANCE ON PPP DG OPINION

The purpose of the PPPDG's review is to ensure that the feasibility study and draft tender/contract documents are complete and of good quality, providing the comprehensive information required by the PPP Board to make an informed decision, on whether or not the project should proceed to the next phase.

The PPPDG needs to make a clear recommendation to the PPP Board, which will ultimately decide if the project proceeds or not to tender. If the quality of the feasibility analysis and draft contract & tender documents submitted by the Contracting Authority is inadequate, incomplete, or unworkable, then the PPPDG may recommend that some of Phases II and III's specific procedures be updated or revised.

Estimated Time Required: The amount of time required to complete this review by PPPDG for each project will vary based upon the complexity of the project, and especially upon the quality and completeness of the work done by the Contracting Authority. Incomplete, problematic, or complex projects will take longer to evaluate and to identify revisions that need to be made. If the PPPDG is regularly briefed and updated by the Contracting Authority throughout the project structuring and tender-contract documentation preparation, then it will already be very familiar with the Feasibility Analysis's quality and its findings, and this review may only take 3-5 days. For more complex and problematic PPPs, this review may require 2 weeks.

BEST PRACTICE

If the information provided in these Feasibility Study and Tender Documents by the Contracting Authority are incomplete or difficult to understand, that is probably a good indication that the PPP Board and the PPPDG should require that the Contracting Authority revise its work. In these cases the PPPDG should try to give clear instructions to the Contracting Authority on specifically what needs to be completed or what tasks should be redone.

Over time, as the overall PPP capacity of the Contracting Authorities increases, the quality of these PPP project reports will improve as well. Initially though, it may be likely that some Contracting Authorities will need more guidance from the PPPDG in how to perform these procedures correctly.

5.4.3 GUIDANCE ON BOARD DISCUSSION AND APPROVAL PROCESS

Following the evaluation of the feasibility study carried out by the PPPDG, a meeting is organized between the PPP Board and the Contracting Authority. During this meeting, the Contracting Authority will present the finding of the feasibility study and answer clarification questions from the PPP Board.

The PPP Board will within 10 working days respond with its approval (and any conditions) or rejection of the PPP project to proceed to the tender phase.

The three possible outcomes are:

1. The PPP Board consider that the project meets the desired expectations of in terms of viability and Value for Money: the PPP Board grants its approval for the project to proceed to tender
2. The PPPDG requires additional studies to be carried out by the Contracting Authority. In this case, the Contracting Authority with the support of the Project Management Team prepares the required additional studies that it submits, after review by the PPPDG, to the PPP Board for final decision.
3. The PPP Board considers that the project does not meet the desired expectations of in terms of viability and Value for Money: the PPP Board denies its approval and the project is not implemented as a PPP.

Prior to launching the tendering process, for a project that has been approved by the PPP Board, the PPPDG is required to obtain approval for Government support[58] and/or specific incentives where applicable. Approval for Government support can be obtained from (i) the PPP Board itself, where there is no express requirement for approval by an authority, or (ii) from the relevant Authority. In the case where approval for Government support is required from the relevant Authority, the request is made by the PPP Board.

[58] Articles 18(4) and 47 of the PPP Proclamation and articles 14(4) and 15 of the PPP Directive

Once the PPPDG has the approval to proceed to tender from the PPP Board and the approval for Government support from the PPP Board or the relevant Ministry, it can implement the PPP tender process.

The procedure followed at the tendering phase will vary based on the type of procurement method which has been recommended in the Feasibility Study and approved by the PPP Board:

I. Single Stage Bidding Process

II. Two-Stage Bidding Process

III. Competitive Dialogue Process

IV. Direct Negotiation Process

V. Unsolicited Proposal Process

Estimated Time Required to approve to proceed to tender: 3 - 5 Weeks.

List of Documents Provided in Appendix D - Phase 3 Structuring, Draft Contract & Tender Documents
1. ***Part 1: Background References – Phase III: Type of Procurement Processes***<br>2. ***Part 2: Sample Request For Expression of Interest (REOI)***<br>3. ***Part 3: Sample Request For Qualification (RFQ)***<br>4. ***Part 4: Sample RFQ Evaluation Form***<br>5. ***Part 5: Sample Request for Proposal (RFP)***<br>6. ***Part 6: Template PPP Agreement:***<br>→ ***6.1 Concession Agreement (Greenfield)***<br>→ ***6.2 Concession Agreement (Brownfield)***<br>→ ***6.3 Management Contract Agreement***<br>7. ***Part 7: Executive Memo for Approval of the Feasibility Study and Tender Documents***

6 PHASE IV: TENDER & AWARD (IMPLEMENTING PPP TENDER)

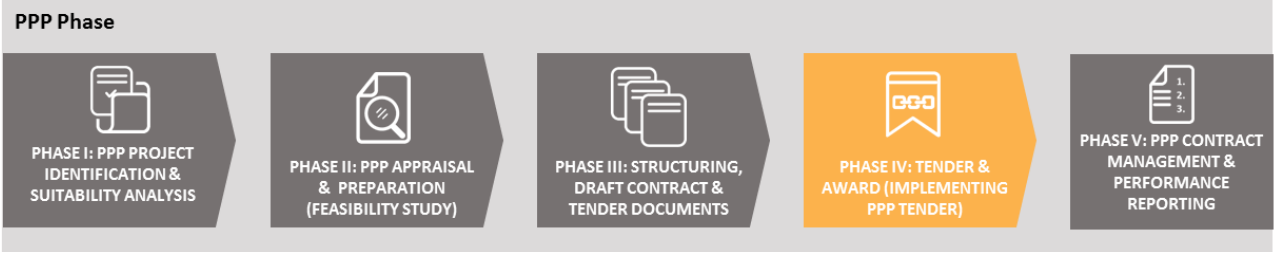

Phase IV presents the procedures and tools for tendering the PPP project and procuring the contract for the private partner to deliver the new services.

Figure 6-1: Overview of Tasks under Phase IV

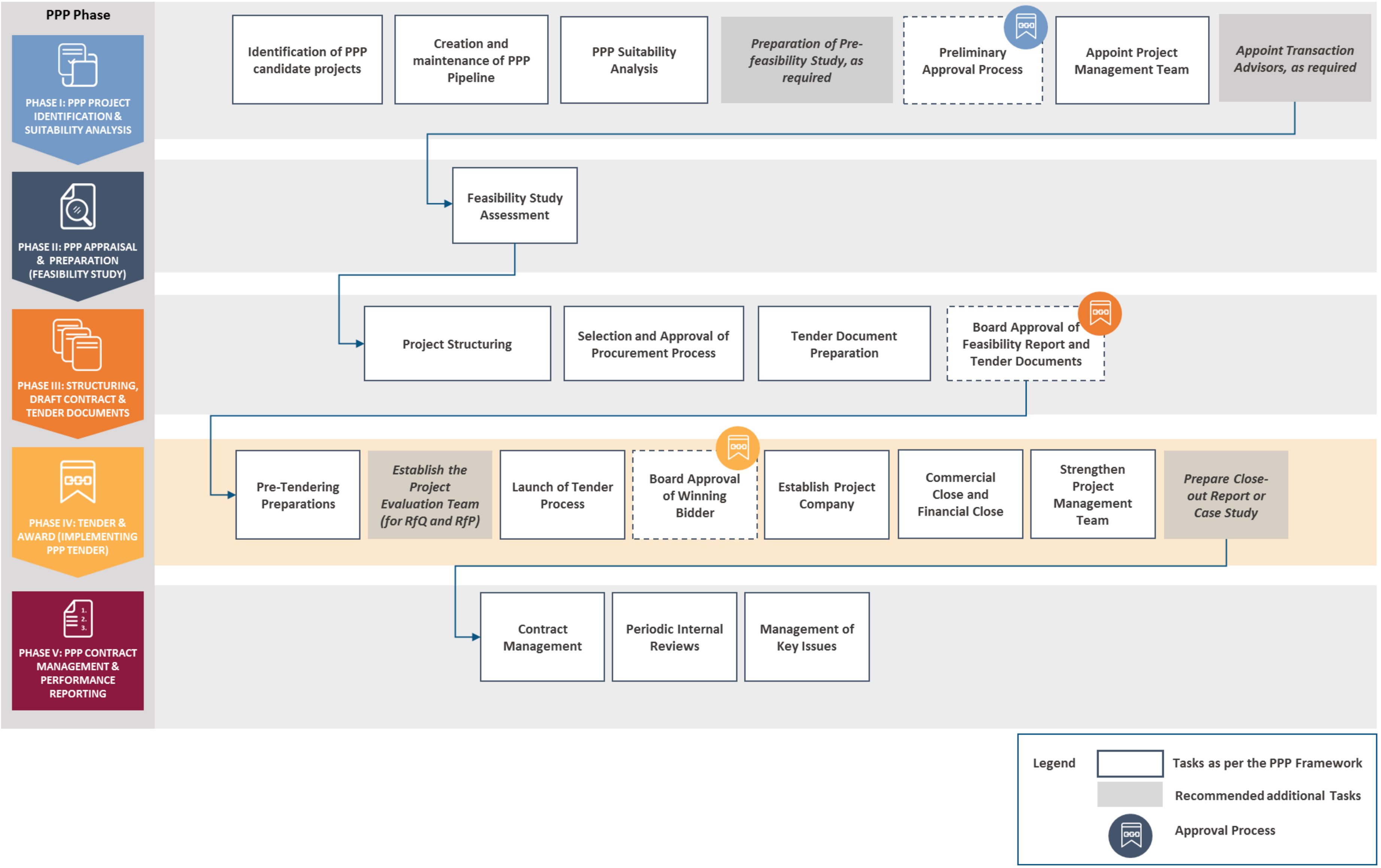

Figure 6-2: Phase IV Process and Key Stakeholders

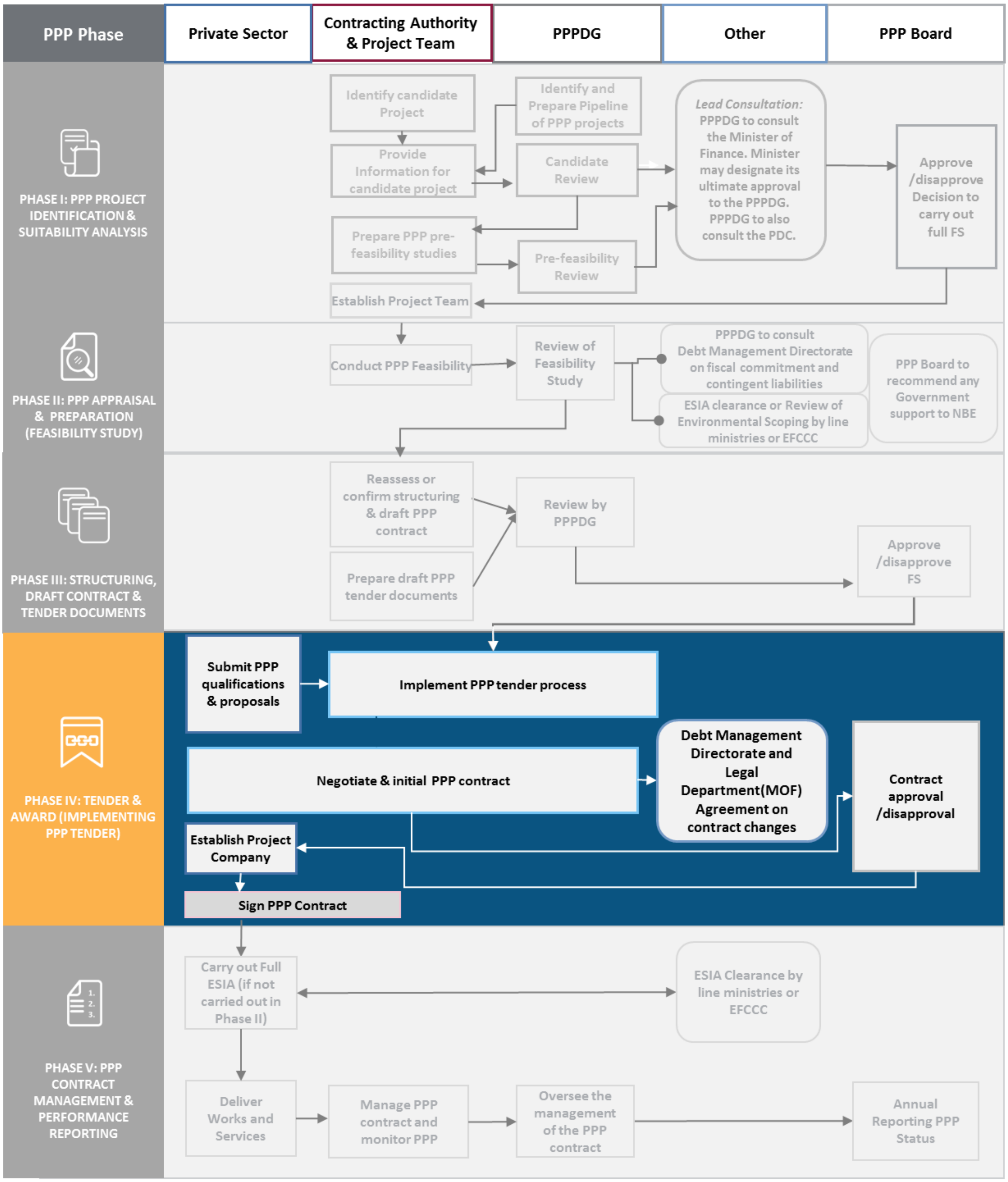

6.1 PRE-TENDERING PREPARATIONS AND RECONFIRMATION OF PROCUREMENT PLAN & TENDER RESPONSIBILITIES

6.1.1 FINALIZE TENDER DOCUMENTS TO INCORPORATE COMMENTS OF PPP BOARD

The Contracting Authority will review with the PPPDG the recommendations and instructions provided by the PPP Board and PPPDG and incorporate this in the draft tender documents and plan, to produce the following:

- Revised PPP Feasibility Study and proposed risk-allocation structure;
- Revised Draft PPP Contract, Draft RFQ and Draft RFP; and
- Revised PPP Tendering Implementation Schedule.

This procedure is performed by the Contracting Authority's Project Management Team, with the support of the Transaction Advisor – and requires all members to have comprehensively reviewed the following, as relevant:

- PPP Pre-Feasibility Study;
- PPP Feasibility Analyses, Draft Contract & Draft Tender Documents Preparation;
- All procedures for Phase III, and requirements of Phase IV.

The purpose of this procedure is to ensure that the process of tendering and procuring the PPP contract is completed in an effective and timely manner. It is important that PPP tender documents are clear and detailed, and that PPP procurement implementation plans feature realistic deadlines and provide the PPP transaction skills required. Specifically, this process identifies the financial and human resources that the PPPDG, with the support of the Contracting Authority and the Project Management Team, will require in order to complete Phase IV: Tender and Award.

The PPP procurement process is led by the PPPDG[59] with the technical support of the Transaction Advisor. The Contracting Authority's[60] has a consultation role throughout the procurement process and participate in the evaluation of the bids as part of the Project Evaluation Team. The Project Management Team'[61]s role is to provide support to the Contracting Authority and to keep a record of all relevant documentation.

6.1.2 CONFIRM SCOPE OF WORK FOR PPP TRANSACTION ADVISORS

If the project requires two separate advisors, one for the feasibility study and the other one for the procurement, the PPPDG will at this stage hire a Transaction Advisor for the tendering phase. If the same advisor who carried out the Feasibility Study is going to continue with the Transaction Advisory, the PPPDG will re-confirm its scope of work for this Phase, which, as presented in **Appendix B** (Part 4: – Indicative TOR for the External Advisor), shall endeavor to address the following key categories:

---

[59] Chapter 5 to 9 of the PPP Proclamation
[60] Articles 7(2-b), 7(2-c) of the PPP Directive
[61] Articles 2(b),2(h), 2(j) of the PPP Proclamation and article 10(4) of the PPP Directive

- Review all PPP feasibility analyses and recommended PPP risk structuring option, and incorporate PPP Board/PPPDG/PMT comments and instructions into the revision of the PPP Feasibility Study, PPP Tender Documents, and Draft PPP Contract;
- Support the PPPDG to ensure that necessary preconditions are completed in advance of the tender (e.g. confirming Government fiscal support, confirming ancillary services and infrastructure will be ready for the project, etc.);
- As relevant, draft and prepare the Information Memorandum, General Procurement Notice, Request for Expression, Request for Qualification and Evaluation Criteria, Request for Proposal and Evaluation Criteria, and PPP Agreement;
- Assist the PPPDG in the coordination and management of tendering activities such as investment road-shows, Bidders' Conference, site inspections/site visits, as well as the setting-up and management of physical and /or virtual project data rooms as needed;
- Assist the PPPDG in identifying interested, potential private sector providers for similar projects (i.e. size, location, etc.);
- Assist the PPPDG in drafting responses to all questions submitted by interested and short-listed firms, including making any needed amendments and/or modifications to the PPP tender documents and PPP Agreement;
- Provide support to ensure that the PPPDG complies with all relevant PPP legal requirements and regulations;
- Assist the PPPDG in managing the evaluation process for the submitted RFQ and RFP received from Bidders;
- Assist the PPPDG and the Project Management Team with monitoring the progress of the PPP contractor during the subsequent PPP contract management and performance monitoring phase, including reaching financial closure, project construction & commissioning, and during the initial operational/service delivery phase.

6.1.3 PREPARE AND IMPLEMENT ACTION PLAN

Prior to the launch of the tender process, the PPPDG must ensure that the project is **'market-ready'** for tendering to private bidders. This procedure involves completing the required pre-tendering preparations; often, the PPP Feasibility Study indicates that project viability is contingent on the completion of specific tasks, such as acquiring land; obtaining licenses and permits; adjusting end-user tariffs; clarifying ownership of existing assets; enforcing specific laws/ordinances; and establishing the PPP Contract Management Plan. As such preparations can be time-consuming and challenging, they shall be addressed well before this phase of the project life cycle.

Given that this procedure often involves the participation of other public authorities and government organizations (ministries, regulators, other public authorities, etc.), the PPPDG will also act as a facilitator in the completion of these preparations.

At this stage the procurement plan prepared in Phase II will be revisited by the PPPDG with the support of the Contracting Authority.

The **revisited procurement plan** will include the following:

- Detailed description of all required preparations;
- Descriptions of specific activities for each reform;
- Key stakeholders involved;
- Listing and notification of the organizations whose approvals and/or decisions are required;
- Identification of any new the human or financial resources required to complete preparations;
- Proposed schedule for completion of each task.

In practice, some required pre-tendering preparations may be difficult to complete or unpopular. Some Governments have tried to simply make them the responsibility of the new private service provider. This should only be considered if they are clearly part of the risks that the service provider can manage and control better than the Government. Otherwise, such a practice ends up undermining the viability of the PPP by seeking to transfer too much risk to the service provider.

Estimated Time Required: The amount of time required to complete the pre-tendering preparation depends significantly on the nature, the complexity, the need to hire a separate transaction advisor, and especially the "readiness" of the specific PPP project. For the larger, more complex, or "less ready" PPP projects, this procedure could take up to 1-3 months or longer to complete. For simpler PPP projects that are clearly "ready" this procedure may only take 1 month to confirm its readiness. It should be noted that as with several other procedures, the planning for this procedure should start very early-on and it can be carried out concurrently with other procedures.

6.2 ESTABLISHMENT OF PROJECT EVALUATION TEAM/PROCEDURES

The PPPDG is responsible for the evaluation of bids[62]. In order to carry out this task, it will establish a Project Evaluation Team in coordination with the Contracting Authority. The PPPDG, supported by the Transaction Advisor will choose the optimal structure and membership of the Project Evaluation Team.

6.2.1 ROLE AND COMPOSITION OF THE PROJECT EVALUATION TEAM

The Project Evaluation Team will carry out the evaluation of the RFQ (as relevant), and the evaluation of the Technical and Financial Proposal submissions. The Project Evaluation Team shall evaluate the RFQ submissions to identify those bidders that are 'qualified' and place these qualified bidders on a 'shortlist' of bidders permitted to compete in the RFP stage. The objective of the evaluations is to provide an objective and defensible process to assist in the prequalification of bidders.

[62] Articles 23, 27, 28 of the PPP Proclamation

The Evaluation Team shall comprise of a wide range of qualified participants and subject matter experts with the requisite expertise to (i) assess general qualifications, such as the relevant industry experience of the Bidder, and to (ii) scrutinize the viability of the Operational /Business Plan and legal framework presented in the Technical Proposal.

Members of the Project Evaluation Team shall be unbiased and free from conflicts of interest. Members of the team must also commit to remaining independent throughout the evaluation period, maintaining confidentiality of the proceedings and their evaluation.

For larger or complex PPP, with significant representation from various Public Entities, it may be beneficial to group the various stakeholders within the Project Evaluation Team. Procedurally, over the course of the evaluations, each group would be responsible for reaching a consensus and completing one Evaluation Sheet for each Bidder's Proposal on behalf of the group.

Assigning Sub-Groups, and Evaluators/Observers

For larger and complex PPPs, the composition of the Project Evaluation Team for the prequalification and RFP stage may be grouped based on representation from the various key stakeholders involved. For instance, the nominated evaluation committee may be structured to represent the following:

- A group representing the PPPDG;
- A group representing the Procurement Department of the MDA/Contracting Authority;
- A group representing the Legal Department of MDA/Contracting Authority;
- A group representing the relevant regulatory body or regulatory authority overseeing concessions in the country; and
- A group representing the PPP Transaction Advisors.

Members of the Project Evaluation Team should also be identified as either **Evaluators** or **Observers.** For instance, a regulatory body with a limited consulting role, may be best suited as an Observer of the evaluation procedures.

6.2.2 SUPPORT FROM TRANSACTION ADVISOR IN ESTABLISHING THE PROJECT EVALUATION TEAM

The Transaction Advisor will support the PPPDG in the preparation of the confidentiality agreement and provide support to the Project Evaluation Team in managing the evaluations.

6.2.2.1 Prepare Confidentiality Agreement

As part of the preparation process, and ahead of the evaluation sessions, the Transaction Advisor shall prepare a Confidentiality Agreement for the PPPDG to distribute to all members of the Project Evaluation Team, for review and signature. This will commit each Member to refrain from disclosing any aspect of the process to any third party not part of the evaluation process. This is an important steps towards maintaining the confidentiality and integrity of the procurement process. Bidders may not be allowed to contact members of the Project Evaluation

Team whether directly or indirectly during the evaluation period to inquire about the outcomes of the evaluation.

The Confidentiality Agreement is to be executed between the individual members of the Project Evaluation Team, and a selected representative of the PPPDG. This Confidentiality Agreement will encompass both the prequalification and Proposal stages of the project (as relevant).

Upon the initial convening of the Project Evaluation Team, the first agenda item shall be to confirm that all members of the Project Evaluation Team have signed the Confidentiality Agreement; the PPPDG shall maintain the hard copies for recordkeeping.

6.2.2.2 Managing the Evaluations

The Transaction Advisor will be responsible for supporting the Project Evaluation Team throughout the evaluations, including preparing all relevant documentation: (i) Evaluation Criteria, (ii) Evaluation Guidelines for the Project Evaluation Team, and (iii) Scoring Sheet templates, (iv) Carrying out an evaluation training. The PPPDG will be in charge of moderating the evaluation sessions towards reaching a consensus.

The Transaction Advisor will also provide secretarial services to record the outcomes of the evaluation process – followed by the preparation of an Evaluation Report at both RFQ and Proposal stages (as relevant), summarizing the results and recommendations of the Project Evaluation Team.

During the evaluation sessions, these secretarial services will also include the maintenance of a Microsoft-Excel version of the Scoring Sheets for each Bidder. For instance, upon the completion and signature of the Scoring Sheets for Bidder #1, the Transaction Advisor's Secretary may input these results in the soft-copy spreadsheet during the Project Evaluation Team's evaluations of Bidder #2. This will allow for the expedient inputting of the final scores and quick tabulation of the average scores, and allow for the announcement of the results upon the close of the evaluation session.

Estimated Required Time: The establishment of the Project Evaluation Team may take 2 to 4 weeks.

6.3 LAUNCH OF TENDER PROCESS

This procedure marks the official start of the PPP tendering process from the perspective of interested private service providers. This consists of the PPPDG's first official announcement of the PPP project opportunity to the private sector and invites prospective bidders to submit statements of their interest and brief summary of their capacity and relevant experience.

The tender procedures to follow will rely on the procurement route which has been selected and approved, as discussed in Chapter 5. The procedures outlined below correspond to what is described in the PPP framework as **Open Bidding Process**, comprising a pre-qualification stage and a Request for Proposal stage.

Section 6.7 presents a matrix illustrating the differences in procedures for other procurements processes outlined in the PPP framework.

6.3.1 PUBLICIZE THE GENERAL PROCUREMENT NOTICE AND THE EXPRESSION OF INTEREST (AS RELEVANT)

The PPPDG with the support of the Contracting Authority and the Project Management Team – will lead the procedure of publicizing the GPN and the Request for Expression of Interest (REOI) and carrying out an assessment of the information gathered.

Planning Ahead: The process of liaising with vendors, reserving and paying for the simultaneous adverting of this EOI in several different journals and publications at once, may require several weeks of preparation time.

The REOI is generally published in relevant trade journals, industry magazines, newspapers and on webpages so that the maximum number of potential private bidders can be reached. The REOI shall be publicized in a suitable number of local and/or international journals, as relevant to the project scope and eligibility requirements:

- **Local circulation:** Local newspapers and engineering & construction-related journals shall be considered.
- **International circulation:** International online portals may be considered, such as the following:
  - i. UNDB Development Business
  - ii. DevelopmentAid
  - iii. Devex International Developments
  - iv. Africa Gateway
  - v. Global Tenders

Online Publications: Many of the international online portals publish tender notices free of charge – and provide an opportunity to significantly expand the circulation of the opportunity.

Informing the commercial attachés of foreign embassies and in-country representatives of Multilateral Development Banks – such as the World Bank/ International Finance Corporation and European Bank for Reconstruction and Development (EBRD) – can be an effective means of garnering support to publicize the project opportunity.

The Transaction Advisor will prepare a **Market Sounding Report**, summarizing the responses to the GPN received / the outcome of any stakeholder engagement activities carried out. This will include the Transaction Advisor's recommendations on whether any feedback from the market should be incorporated in the project going forward.

Estimated Time Required: The process of reserving and paying for the simultaneous adverting of this REOI in several different journals and publications at once, may require several weeks to prepare, reserve, and complete. The review of submitted EOIs may take only 2-3 days to review and summarize them.

6.4 MANAGEMENT OF PREQUALIFICATION PROCESS

6.4.1 ISSUING THE RFQ AND RECEIVING PREQUALIFICATION SUBMISSIONS

This procedure describes how the RFQ document will be released to interested bidders by the PPPDG with consultation from the Contracting Authority and the Project Management Team, and how submissions will be evaluated in order to produce a limited number of capable and experienced bidders ('shortlist') to submit a full tender.

At this stage, the RFQ notice should already have been drafted, and be ready for the PPPDG to issue the notice. The PPPDG shall issue the RFQ notice in a widely circulated publication and, as relevant, to potential bidders that it has identified.

Procedures for Hard Copy Submissions:

- In preparation for the receipt and collection of submissions for the RFQ, the Transaction Advisor will provide a **Bid Registry Form**, to be maintained at the desk of the specified address for submission – at the offices of the PPPDG – in order to record the name of the bidder; the name and signature of the representative submitting the submissions for RFQ; and the time at which it was received.
- On the day of closing of the bid process, the PPPDG shall manage the process of receiving and registering all submissions.
- The Transaction Advisor will be required to oversee the bid submission process and completion of the Bid Registry on the day of closing of the bid process. The PPPDG will then take custody of the bid submissions for safekeeping at a designated secured location, inside its offices, until the official Bid Opening ceremony and evaluations take place.

Procedures for Soft Copy Submissions:

The PPPDG can allow for soft copy submission along with hard copy submission through pen drive or CD-ROM.

Estimated Time Required: The amount of time required to issue and receive RFQ may take up to 45 days to complete. However, for smaller, simpler, or less risky projects, this procedure may only need 3 week to complete.

6.4.2 EVALUATING THE SUBMITTED RESPONSES FOR THE RFQ

The Project Evaluation Team will evaluate, based upon the quantity and quality of submission received, whether or not there is sufficient private sector interest in PPP to proceed to tendering. If the quantity and quality of the prequalification submission is especially limited, then the PPPDG and the Contracting Authority may need to consider other options before proceeding to PPP tender, such as increasing public sector contributions, offering additional credit enhancements, or other restructuring options to make the project more attractive/'bankable' as a PPP.

Managing Cases of Limited Responses:

If only one or two responses are received, it may indicate that the PPP project was not conceived or structured well. The PPPDG and the Contracting Authority/PMT should ascertain the possible reasons for the lack of private market interest. The PPP Feasibility Study and proposed PPP risk-allocation structure may have to be reviewed and amended. Another round of the RFQ process may be required, under revised project parameters or with wider publicity for increased market interest.

Upon the launch of the RFQ notice – Bidders are provided with a specified response time, leading up to the submission deadline. The PPPDG will work with the Transaction Advisor during this intervening period to prepare for the prequalification submission Evaluations, such as:

- Scheduling the Training Workshop and prequalification Evaluation Sessions with the Project Evaluation Team;
- Circulating the agreed upon Evaluation Criteria, Evaluation Guidelines and Scoring Sheets to the Project Evaluation Team;
- Circulating the Confidentiality Agreement for all members of the Project Evaluation Team to review, for their hard copy signature upon the convening of the Team at the Workshop/Evaluation Session.

6.4.2.1 Schedule Training Workshop and Prequalification Evaluations with Project Evaluation Team

Depending on the volume of submissions and complexity of the project, prequalification Evaluations are typically carried out by the Project Evaluation Team over full-day sessions of 1-2 days.

It is best practice to schedule a Training Workshop one day prior to the Evaluations, or as the first agenda item on Day 1 of Evaluations.

6.4.2.2 Carry Out Training Workshop

At this stage, the Transaction Advisor will provide a training workshop on the prequalification evaluation process for the Project Evaluation Team. A PowerPoint deck will be prepared, covering the following:

- Overview of the prequalification stage and objectives;

- Overview of prequalification process carried out and RFQ publications, including the number of submissions received;
- Review of evaluation criteria, including (i) guidance on any minimum thresholds or requirements and (ii) guidelines for the basis of how each evaluation criterion will be scored; and
- Review of procedures and quality assurance processes.

6.4.2.3 Carry Out Prequalification Submission Evaluation

i. All submissions will be brought together into a single room at a designated location and opened in the presence of appropriate representatives;

ii. A ***designated Secretary*** provided by the PPPDG will note the name and contact person details of each Bidding Entity submission. The full list will be designated as the ***List of* prequalification submissions *received***;

iii. One copy of each prequalification submission will be retained by the PPPDG to enable the scanning and capture of the pages in the prequalification submission for the Project Evaluation Team;

iv. The remaining copies will then be moved to the Evaluation Room under the direction of the ***designated Chairman*** of the Project Evaluation Team.

v. The ***designated Secretary*** will count and confirm that the *prequalification submissions* received in the Evaluation Room conforms to the *List of prequalification submissions Received*. The available copies of each prequalification submission can then be accessed by/distributed to the individual members of the Project Evaluation Team;

vi. Each member of the Project Evaluation Team will review each of the prequalification submissions.

vii. Upon discussion and evaluation – which may be chaired by a designated representation from the Transaction Advisor – the Evaluation Sheets may be completed. Depending on the number of submissions and complexity of the project, it may be possible to complete one Evaluation Sheet for each prequalification submission, to be signed by each member of the Project Evaluation Team once a consensus is reached.

viii. If, following this discussion, the Project Evaluation Team is unable to reach a unanimous conclusion, the final decision will be made by majority vote.

Sample Agenda

Technical Proposal Evaluations – Day 1

- All participants to complete the Attendance Sheet;
- All participants in the Project Evaluation Team to sign the Confidentiality Agreement, if not already carried out at the prequalification stage (as relevant);
- Transaction Advisor to review of the evaluation forms to be completed by the Project Evaluation Team and related Guidelines;
- Transaction Advisor to provide a brief description of how the evaluation process would proceed;
- E.g. Assessment of basic compliance of bids received; Assessment of the Relevant Experience criteria of all bids received; Assessment of the Business Plan/Operating Plan criteria of all bids received.

Technical Proposal Evaluations – Day 2

- E.g. Assessment of the Business Plan/Operating Plan criteria of all bids received (Con't);
- Review of results of evaluations, based on the aggregate average scores tabulated.

Guidance regarding references and integrity checks

As a general rule bidders will have been asked to provide references regarding experience and sign declarations concerning the validity of the statements made as part of the submission. Clearly it would not make sense for the evaluation team or the PPPDG to check up on all the information provided. Some of the bidders may be well known to the Contracting Authority and others may be totally unknown and require some basic checking to validate who they are and that their claims stack up (web searches, credit check, take up references). Much depends on the number of responses and time available but some limited checking would be recommended.

6.4.2.4 Prepare PPP Bidder Prequalification (Shortlist) Report

Following evaluations, a **PPP Bidder Prequalification (Shortlist) Report** will be prepared, towards ensuring transparency of the procurement process and facilitating subsequent decision-making by the PPPDG. This Report will include the following:

- Copy of the RFQ publication and list of the relevant publications/journals;
- List of interested bidders who requested copies of the RFQ, including an accounting of any required payments received from interested bidders for copies of the RFQ (as relevant);
- Description of the evaluation criteria applied, including any minimum benchmark criteria;

- Description of the evaluation process, including the final list of pre-qualified bidders and unsuccessful bidders – including the reason why these bidders did not qualify. This will include a copy of the completed Scoring Sheets (refer to RFQ sample template provided in **Appendix D – Part 3**) for each prequalification submission;
- Description and assessment of all comments and questions received from interested bidders about the proposed PPP (e.g. regarding the risk allocation structure, the project's output standards, etc.). This will inform the subsequent procurement stage for which the PPPDG will need to decide whether and how the final structure of the PPP project will be amended or clarified prior to the release of the final tender documents.
- **The PPPDG's recommendations on which Bidders should be included on the Shortlist and invited to submit a Proposal, for Approval.**

RECOMMENDED PROCESS
In some countries, certain PPPs are so sensitive that the public sector may choose to regularly report on the PPP process to maintain public support; this may be the case railway, port or power concessions. Article 12.10 of the PPP Proclamation provides for such public announcements to be made regarding the PPP, which indicates that the PPP Director General shall *"at certain intervals, determined by the Board provide information to the public on the implementation of public private partnership projects."* Depending on the magnitude of the project, the PPPDG may consider preparing a press statement, for instance, to communicate to all key stakeholders that interest in and competition for the PPP is expected to be strong.

Once the RFQ process is finished the PPPDG, as relevant, will publicize an announcement of shortlisted bidders in widespread print and online newspapers and address a letter to shortlisted bidders announcing the result.

Estimated Time Required: The evaluation of the prequalification submissions and the publication of results to shortlisted bidders may take 1 to 3 weeks.

6.5 MANAGEMENT OF REQUEST FOR PROPOSAL PROCESS

The PPPDG will manage the bid process, with support, as needed, from the Contracting Authority and the Project Management Team. The bid management process will include the following key tasks:

i. Set up virtual and physical Data Rooms

ii. Support client in answering bidders' questions, managing the data rooms, and providing regular updates to bidders;

iii. Manage updates to bid documents and distribution to prequalified bidders, as required; and

iv. Update the bid process schedule, confirming milestones, task allocation, critical decision points and responsibility allocation.

6.5.1 SETTING UP THE PHYSICAL/VIRTUAL DATA ROOM

Data rooms are typically libraries of numerous volumes of detailed records, reports, and plans relevant to a specific (usually large) infrastructure project that bidders may access while they are preparing their bids. For concession-type PPPs that require private partners to take over the operation and management of existing facilities, having access to historical design plans, asset registries, and operating and maintenance data will be critical. For PPP projects in which the bidders will be assuming end-user demand risks, having access to detailed demand, billing, collection, and revenue records will also be critical.

'Greenfield' PPPs that feature the construction of brand-new facilities on vacant land and have a single public sector off-taker (such as for the construction of new schools or hospitals or public buildings) usually require smaller levels of background information (and therefore smaller physical and/or virtual data rooms) than do **'brownfield' PPPs** or PPPs that transfer market demand risks onto private partners.

'Virtual' data rooms (VDR) typically use webpages to make this information available electronically to qualified bidders. The VDR is developed in order to house the tender documentation, and other supporting materials for Bidders in their proposal preparation.

The PPPDG will give confidence to interested bidders that all relevant information has been systematically collected, verified, and is readily available for their review. The contents of physical and/or virtual project data rooms for the PPP project may include:

- Existing project designs and construction engineering reports
- Updated infrastructure network maps and expansion plans
- Asset and equipment registries
- Inventory records
- Equipment maintenance and other maintenance records,
- Customer databases and cadastral studies
- Existing service contracts held by the client Contracting Authority, etc.
- Audited financial statements of the Contracting Authority, if available
- Information on other liabilities, such as debts, held by Contracting Authority
- Other detailed technical, financial, and legal reports and records relevant to the specific PPP project

The PPPDG with the support of the Contracting Authority and the PMT will lead this task, including the following:

- Shortlisted Bidders shall be provided with a **Confidentiality Agreement** in order to gain access to the tender package. Upon return of the signed Confidentiality Agreement, shortlisted bidders would then be provided with the Request for Proposal (RFP) and Draft PPP Contract, and any other relevant documents;
- Develop and grant access to the VDR to the shortlisted Bidders;

- A physical data room shall also be maintained in a secured location. The physical data room will be located within the PPPDG.

Lessons Learned and Good Practices:

While bidders should be given access to the technical and sector background records, reports, and plans they will need to prepare competitive, innovative, and detailed PPP bids – one area of information that they should NOT be given, as part of the virtual and/or physical project data room is related to the Government's affordability limit for the PPP.

- The reason is that PPPs work best for Governments when private bidders prepare their financial bids on a "zero-budget basis" in response to the required output levels of service from the PPP project – as opposed to preparing a financial bid based upon someone else's estimation of what it should cost.

- **Zero-based budgeting** means that the bidder begins from "zero" and proposes costs for each element of the project (design, construction, operation, refurbishment, etc.) based upon the most innovative, competitive, and cost-efficient design management, construction management, and overall service delivery management strategy they can produce. Such zero-based budgeting takes more time and effort to produce.

- By contrast, if a bidder is told that for a specific new project the Government's affordability limit is US$5 million, then bidders will tend to automatically determine they should propose their own bid around 10% lower than this announced benchmark price, such as US$4.5 million in order to win the PPP procurement – regardless of what their actual costs are. Their actual costs based upon a "zero-based" approach and using the most competitive, innovative approach, could be much less, such as US$3 million.

Estimated Time Required: The implementation of the Data room may take to 1-3 full weeks.

6.5.2 ISSUE BID DOCUMENTATION TO APPROVED SHORTLISTED ENTITIES

Once the Bidders have access to the tender package, they may be requested to return a set of Technical Forms provided within the RFP (either in hard copy or soft copy – to be sent to the designated email address), by a specific deadline. This may be related to the following preliminary tasks:

i. Confirm intent to submit a Proposal;

ii. Confirm participation at Site Visit;

iii. Confirm participation at the Bidders' Conference.

6.5.3 MANAGE REQUESTS FOR CLARIFICATION

Upon the release of the bid package, Bidders may submit any questions/comments towards informing their preparation of the Proposal. Bidders will be given two to three weeks to submit their comments to the PPPDG after the official release of the bid documents.

The bid package will include a Comment Matrix template, so that any comments received are systematically recorded. The PPPDG will manage the process of drafting responses to all such queries with support from the Project Management Team, the Contracting Authority and the Transaction Advisors. The completed Comment Matrix will be uploaded in the Data Room(s) – so that all prospective Bidders are provided with the same level of information. In order to assure equal treatment, the final Comment Matrix, including all responses, will be uploaded in the Data Room(s) – so that all prospective Bidders are provided with the same level of information.

6.5.4 ARRANGE SITE VISIT(S)

Some PPP projects that require complex construction activities, such as the construction of a new road along a lengthy corridor, may include ‘site inspections’ and ‘walk-throughs’ hosted by the Contracting Authority and the PPP Transaction Advisors for Bidders’ technical staff to physically inspect key project sites first-hand. Site visits should be conducted between 4 – 8 weeks after the issuance of the RFP, to allow sufficient time for logistics and planning.

A site inspection or site visit may be coordinated during the Proposal preparation period. Depending on the extent of shortlisted Bidders, it may be beneficial to either coordinate site visits for each shortlisted Bidder, or to coordinate one collective group visit.

The Contracting Authority with support from the Project Management Team and the Transaction Advisors will oversee the site visit(s) and respond to any questions/requests for clarification raised during the visits. A Site Visit Attendance Log will be completed for recordkeeping.

Any questions that emerge from these visits will be submitted to the PPPDG in writing, and both the full text of the question as well as the full text of the answer or response by the PPPDG will be distributed to all short-listed bidders.

6.5.5 HOST A BIDDERS’ CONFERENCE

The PPPDG has the option of hosting a Bidders’ Conference for shortlisted Bidders and their partners, should it be deemed valuable and relevant. The Bidders’ Conference is an important milestone in any transaction process. Typically, the Bidders’ Conference provides Bidders with an opportunity to raise any comments or questions for clarification in response to the bid package and any other relevant issues, and to discuss the project’s key parameters.

Bidders are usually provided with an opportunity to submit comments and questions for clarification on the bid documents, in the run up to the Bidders’ Conference.

The Bidders’ Conference will include a presentation by the Transaction Advisor, to provide an overview of the bid process, evaluation criteria, and revised transaction schedule.

Discussion about disclosure of information during the tendering phase is detailed in section 6.10.

6.5.6 MANAGE ANY REVISIONS TO THE BID DOCUMENTS WITH PPPDG/CONTRACTING AUTHORITY

Following the receipt of clarification questions, and following the site visit/Bidders' Conference, there may be a need to incorporate specific revisions to the tender documents and/or the Draft PPP Agreement in the Data Room(s). The PPPDG, in consultation with the Contracting Authority and its Project Management Team, is responsible for making these revisions. All Bidders will be notified by the PPPDG if revised documentation is re-issued through the Data Room(s).

Initialling of the Draft PPP Agreement in Bid Submission

- The PPPDG will require Bidders to review the Draft PPP Agreement and to send back marked-up copies of these agreements in advance of the Bidders' Conference. This allows the Transaction Advisor with sufficient time during the bid preparation process to review comments on the PPP Agreements and to discuss these with the PPPDG, the Contracting Authority and its Project Management Team.
- This approach may save considerable time during the transaction process, specifically, during negotiations. This is because after the Bidders' Conference, final copies of the PPP Agreements are sent to all shortlisted bidders. Bidders would be required to submit initialed original copies of the PPP Agreements with their Technical and Financial Proposals – essentially agreeing to the terms, conditions and clauses of the PPP Agreement. This original, initialled copy, serves as the document that is used for negotiations when a bidder is identified as the Preferred Bidder.
- Since that bidder would be already familiar with the PPP Agreement, this minimizes the need for lengthy negotiations over the terms, conditions and clauses to the PPP Agreement. The only elements that are negotiated are the schedules to the PPP Agreement, which are frequently extracted from the Technical and Financial proposal submissions of bidders.

6.5.7 RECEIPT AND REGISTRATION OF SUBMISSIONS

Procedures for Hard Copy Submissions:

- In preparation for the receipt and collection of submissions, the Transaction Advisor will provide a **Bid Registry Form**, to be maintained at the desk of the specified address for submission – inside the offices of the PPPDG – in order to record the name of the bidder; the name and signature of the representative submitting the Proposal; and the time at which it was received.
- On the day of closing of the bid process, the PPPDG will manage the process of receiving and registering all submissions.
- A bidder who intends to bid for a project shall complete and submit a technical proposal and a financial proposal and enclose each proposal in a separate sealed envelope as may be specified in the request for proposals.

- The Transaction Advisor will be required to oversee the bid submission process and completion of the Bid Registry on the day of closing of the bid process. They may then take custody of the bid submissions for safekeeping at a designated secured location, such as the PPP Transaction Advisor's offices, until the official Bid Opening ceremony and evaluations take place.

Procedures for Soft Copy Submissions:

The PPPDG can allow for soft copy submission along with hard copy submission through pen drive or CD-ROM.

Estimated Time Required: Issuing RFP and receiving bids may take up to 8 weeks.

6.5.8 BOND COMPLIANCE

The PPPDG will be required to contact the institution underwriting the bid instrument and confirm with the instrument signatory the validity of the document. The PPPDG with support from the Transaction Advisor will provide a written statement confirming communication with the institution and acknowledgment of the validity of the instrument.

6.5.9 PROPOSAL EVALUATION AND APPROVAL

6.5.9.1 Host Training Workshop

At this stage, the PMT with support from the Transaction Advisor will provide a training workshop on the Proposal evaluation process for the Project Evaluation Team. A PowerPoint deck will be prepared, covering the following:

- Overview of the proposal stage and objectives;
- Overview of the shortlist/number of Bidders which have confirmed intent to bid;
- Review of evaluation criteria, including (i) guidance on any minimum thresholds or requirements and (ii) guidelines for the basis of how each evaluation criterion should be scored; and
- Review of procedures and quality assurance processes.

6.5.9.2 Technical Proposal Bid Opening and Evaluations

Bid Opening:

Technical bid opening will be carried out at the time of the deadline of submission in a place to be communicated to bidders in the RFP. All private bidders who submitted bids can attend this event, as should other relevant stakeholders, including interested lenders, relevant donor agency officials, and the local business press. This public event serves to strengthen the transparency of the overall tendering, procurement, and bid selection process, and improve the confidence of all parties that the process is being conducted fairly. Usually such events feature the Chairperson of the proceedings, a member of the PPPDG, announcing the following for each technical bid package received:

- Announce the name of the party that is submitting the bid written on the outside of the box or envelope containing the Technical Proposal;

- Break the seal of the box/envelope; Account for all volumes of the Technical Proposal enclosed, their titles, and the number of copies provided;

- Announce that if any of the bidders have questions about this final receipt of technical proposals they must submit them in writing to the PPPDG;

- Announce any planned dates for next steps, such as: When the Project Evaluation Team expects to complete its first review of Technical Proposals; Planned dates for the opening of financial bids and the announcement of the preferred bidders. Such announcements should strive to be accurate and should not "over-promise and under-deliver." Making such announcements helps gives confidence to bidders, lenders, and other stakeholders that procurement process will continue to move smoothly, transparently, fairly and effectively.

Evaluation Tasks:

The evaluation of technical bids for PPP project is conducted by the Project Evaluation Team on the relevant technical conditions being proposed by each bidder, assigning specific scores to each of these conditions. This allows the evaluators to determine first whether a PPP technical proposal adequately addresses the project's specific technical needs, and is therefore deemed "responsive", or whether it does not address these PPP technical performance standards, and should therefore be deemed "non-responsive."

Note that this evaluation of technical proposals is being done before and separately from the subsequent evaluation of financial proposals.

The methodology to be followed in performing the technical evaluation is provided to each evaluator prior to beginning the evaluations.

i. All submissions shall be brought together into a single room at a designated location and opened in the presence of appropriate representatives;

ii. A ***designated Secretary*** provided by the PPPDG shall note the name and contact person details of each Bidding Entity submitting the Proposal. The full list will be designated as the ***List of Proposals Received***;

iii. One copy of each Proposal shall be retained by the PPPDG to enable the scanning and capture of the pages in the Proposal for the Project Evaluation Team;

iv. The remaining copies shall then be moved to the Evaluation Room under the direction of the ***designated Chairman*** of the Project Evaluation Team.

v. The ***designated Secretary*** shall count and confirm that the Proposals received in the Evaluation Room conforms to the *List of Proposals Received*. The available copies of each proposal can then be accessed by/distributed to the individual members of the Project Evaluation Team;

vi. Each member of the Project Evaluation Team shall review each of the Proposal submissions.

vii. Upon discussion and evaluation – which may be chaired by a designated representation from the Transaction Advisor – the Evaluation Sheets may be completed.

viii. The evaluation process should begin immediately after opening of the Proposal with a preliminary examination to verify the overall completeness of the Proposal received as required by the RFP before undertaking their detailed technical and financial evaluation. All Proposals should be subjected to a preliminary examination. This action enables evaluation committee to identify and reject Proposals that are incomplete, invalid or substantially non-responsive. The results of preliminary examination should be presented in the Evaluation Form with the reasons for failure being be clearly explained, as necessary. Rejection at this stage puts the Proposal out of any further considerations and t should be ensured that the decision to reject is justifiable.

ix. Depending on the number of submissions and complexity of the project, it may be possible to complete one Evaluation Sheet for each Proposal submission, to be signed by each member of the Project Evaluation Team once a consensus is reached.

x. If, following this discussion, the Project Evaluation Team is unable to reach a unanimous conclusion, the final decision will be made by majority vote.

As part of the evaluations, the Project Evaluation Team needs to ensure that the overall technical approach of the bidder is in fact viable and would be capable of meeting the required technical output performance standards of the project.

Guidance regarding references/integrity checks

Bidders will have already been prequalified and additional checks on the bidders should not be necessary unless there have been changes in the bidders profile, consortium membership, other changes etc. that would be subject to approval by the contracting authority and require some checks to take place. Checks on submitted bid bonds in terms of amount, format, issuing bank/organisation will have to be made and any queries/follow up made on a timely basis. Clearly validation of supporting documentation may also prove necessary where the organisation is not readily known e.g. foreign bank support letter.

6.5.9.3 Prepare Evaluation Report on Technical Evaluation

Following the technical evaluations, an Evaluation Report will be prepared by the Transaction Advisor, towards ensuring transparency of the procurement process and facilitating subsequent decision-making by the PPPDG. This Report will include the following:

- Record of the Bidders' Conference and Site Visits, their attendance, clarification questions raised, and any subsequent revisions to the tender documentation and/or PPP Agreement;

- Description of the evaluation criteria applied, including any minimum benchmark criteria;
- Description of the evaluation process – reasons for passing/not passing the minimum technical score.

6.5.9.4 Announcement of Technical Evaluation Results

The announcement of the technical evaluation results will follow this review. The PPPDG will communicate via courier and/or email the result of the technical evaluation to all the bidders. The announcement of the technical evaluation of PPP proposals is done prior to and distinctly separate from the evaluation of financial and cost proposals. It is usual at this stage, for the unopened financial envelopes of all bidders deemed to have failed the Technical evaluation to be returned to each bidder by the PPPDG.

6.5.9.5 Financial Bid Opening

This is the procedure to be followed for the transparent opening of the financial and cost proposals submitted by bidders who have been found to be "technically responsive." Bidders who have passed the technical evaluation will be invited to assist the opening of the financial proposal. This activity leads directly to the determination of the preferred bidder. Note that this procedure, to be carried out by the Project Evaluation Team, comes after, and is distinctly separate from the previous evaluation of the technical proposals.

The purpose of this procedure is to ensure full transparency in both revealing the important final prices that have been proposed by each bidder, and in the process for determining the ranking of the preferred bidders. In accordance with the RfP the evaluation team will still have to assess

Clear Required Formats for Financial Proposals: This procedure re-emphasizes the importance of the PPPDG, its Transaction Advisors and Contracting Authority's PMT to prepare very clear, unambiguous instructions in the tender documents for precisely how bidders should present their financial bids.

If, for example, bidders propose different format for their bids, such as Bidder A proposes a single "Availability Payment" to be paid each month for the duration of the contract, while Bidder B proposed a combination of an "availability payment" plus a variable "usage payment" that are to be paid once per quarter – it become very difficult for evaluators to select which is the preferred bid. Moreover, such uncertainty over how to fairly evaluate such fundamentally different bids almost encourages unsuccessful bidders to file protests against the process. It therefore, critically important for the PPPDG and its Transaction Advisor to provide very clear instructions, including templates, for bidders to use when preparing their bids. If, for example, the tender documents require bidders to submit a single "availability payment" on a quarterly basis for each 3-month period of the contract, and a bidder decides to propose some combination of availability, variable payments, plus end users, that bidder's financial proposal should be rejected as "financially non-responsive."

the financial bids submitted for compliance for instance with instructions, arithmetical correctness, consistency with technical proposal and plans.

6.5.9.6 Prepare Evaluation Report on the Technical and Financial Evaluation

The main outputs from the financial evaluation will include a Financial Bid Evaluation and Contract Award Report, summarizing the following:

- Names of bidders whose technical and financial proposals were opened;
- The technical score of each bidder;
- For each bidder the prices bid for each year of the PPP contract;
- A clear Net Present Value analysis of the total costs to the Government of these proposed prices at the Governments published discount rate;
- An empirical ranking of the financial bids received and an announcement of who the preferred bidder is based upon the NPV analysis and ranking;
- Detailed timeline on moving forward with the negotiations, signing and implementation of contract(s);
- A record of and plan for addressing any protests by the unsuccessful bidders.

If no bids have been submitted, this indicates that the tender process was not well-planned and well-structured. If this situation is caused by insufficient time to prepare bids, the PPPDG shall extend the deadline for bid submission**.**

Otherwise the PPPDG will carry out a due diligence on the viability of the project with the support of the Transaction Advisor. The three outcomes of this assessment are:

(i) If after the due diligence, the PPPDG estimates that adjusting the structure of the project and or requirements can lead to bid submissions, it can restructure the project without compromising Value for Money and carry out a Market Sounding by issuing a General Procurement Notice. If the PPPDG estimates that there is an appetite for this project, it will then seek approval from the PPP Board to proceed to tender.
(ii) The PPPDG enters into Direct Negotiation after approval by the PPP Board.
(iii) If there is no appetite for the project and the project does not seem to be viable, the PPPDG can suspend the tendering process.

If all bids submitted fail to meet the evaluation criteria set forth in the request for proposals, the PPPDG can, upon carrying out a due diligence to assess the viability of the project and market appetite:

(i) Re-tender the project after re-adjusting the structure of the project and confirming market appetite.
(ii) Enter into Direct Negotiation after approval by the PPP Board, if it estimates that issuing a new invitation to the prequalification proceedings and a new request for proposals would be unlikely to result in a project award within the required time frame.
(iii) Suspend the tendering process if it estimates that there is no market appetite for the project and the project does not seem to be viable.

If only one bid has been submitted, the PPPDG will carry out an evaluation of the bid as per the procedure defined in section 6.5.9. If the proposal meets the evaluation criteria, the PPPDG will award the project, as relevant after negotiation, to the bidder. If the bid does not meet the evaluation criteria, similarly to when all bids fail the evaluation criteria, the PPPDG will carry out a due diligence with the support of the transaction advisor to assess the viability of the project and the market appetite. Following this assessment the PPPDG can:

(i) Re-tender the project after re-adjusting the structure of the project and confirming market appetite.
(ii) Enter into Direct Negotiation after approval by the PPP Board, if it estimates that issuing a new invitation to the prequalification proceedings and a new request for proposals would be unlikely to result in a project award within the required time frame.
(iii) Suspend the tendering process if it estimates that there is no market appetite for the project and the project does not seem to be viable.

The procedure for Direct Negotiations is detailed in section 6.7.

Estimated Time Required: Much depends on the number of bids received and the complexity of the project (size, technology, innovation, etc.) together with the amount of technical and financial criteria being evaluated. For the larger, more complex PPP projects, the evaluation may take up to 2-5 full weeks. However, for smaller, simpler PPP projects with simple technical output standards using well-established technologies, the evaluation and announcement process may only require 1-2 weeks to complete.

6.5.10 HANDLING OF COMPLAINTS

The Request for Proposal document will include procedures for lodging complaints or comments on the tender process and outcome. The procedures shall allow for submission of both anonymous and public comments and complaints.

6.5.10.1 Complaints lodged by Private Parties

1. The PPP Directive[63] allows for private parties to lodge complaints against actions of the PPPDG or the Contracting Authority within five days of when the party became aware (or should have reasonably become aware) of the circumstances given rise to the complaint.

    a. This complaint shall be in the form of a written correspondence directed to the Director General of the PPPDG or the highest ranking office in the Contracting Authority. The Team Leader on the Project Management Team shall be copied on this correspondence.

    b. The notice will outline the nature of the complaint, the party that has been aggrieved, and the party that has caused the grievance.

    c. The notice will outline the specific sections of the PPP legal framework or the rules for the private party selection process that has been contravened

2. The PPPDG shall respond to the complaint within ten (10) days of the submission of the complaint.

    a. The PPP process shall be put on hold while the PPPDG addresses the complaint.

    b. The private party is entitled to file a complaint with the PPP Board within five (5) days of a response (or when a response should have been received from the PPPDG), if it is unsatisfied with the response of the PPPDG.

3. Upon receipt of a complaint from a private party, the PPP Board shall issue a notice of receipt of complaint to the PPPDG and the Contracting Authority.

4. Such notice shall be in the form of an official correspondence and shall include the name of the party making the complaint, the nature of the complaint, the party that has been aggrieved, and the party that has caused the grievance.

[63] Article 30 of the PPP Directive

5. The PPPDG and the Contracting Authority shall put on hold the PPP process while the PPP Board resolves the complaint.

    a. Until the PPP Board dismisses the complaint, the legal framework authorizes the PPP Board to:

        i. Prohibit the PPPDG and the Contracting Authority from acting lawfully

        ii. Order the PPPDG and the Contracting Authority to resume the PPP process (with the exception of signing of project agreement and award related decisions

        iii. Annul in whole or in part, an unlawful act or decision of the PPPDG or Contracting Authority

6. The PPP Board shall issue its decisions within twenty-one (21) working days of receiving a complaint.

    a. This decision shall be in the form of written correspondence addressed to the party that filed the complaint

    b. The PPPDG and the Contracting Authority shall be copied on this correspondence

6.5.10.2 Complaints lodged by the PPPDG and Contracting Authority

4. The PPPDG or Contracting Authority may file notice of complaints against PPP project candidates or Private Party implementing PPPs, if it believes its legitimate interests have been harmed or that an unlawful act has been committed.

5. This notice shall be in the form of a written correspondence outlining and shall outline the nature of the complaint, the party that has committed the grievance and the date of said grievance.

6. Upon receipt of this notice, the PPP Board will write to the PPP project candidate or Private Party against whom the complaint has been lodged.

    f. In this written correspondence, the PPP Board will outline the alleged complaint.

    g. The PPP Board may, if it chooses to, invite both the PPPDG or Contracting Authority and the Private Party to appear in person to present their case.

    h. The PPPDG or Contracting Authority and the Private Party are permitted to engage professional assistance in presenting their case to the PPP Board.

    i. The PPP Board shall review and give a decision on the complaint within twenty-one (21) days of receiving of such a complaint.

        i. The PPP Board may suspend the PPP project candidate or Private Party implementing the PPP Project for a definite or indefinite period

            - In instances where a suspension of the Private Party implementing the PPP project may cause disruptions in Public Service Activity, the

PPP Board may elect to adopt other penalty measures available in the PPP Agreement or similar punitive measure available under the Laws of Ethiopia

ii. The PPP Board may also give a written warning

iii. The PPP Board may also dismiss the complaint

The PPP Proclamation provides that "the complaints and review mechanism provided under the Ethiopian Federal Government Procurement and Property Administration Proclamation, as they may be amended or replaced, shall be applicable mutatis mutandis to the PPP Proclamation".

To avoid uncertainty within the procurement process, the Contracting Authority must ensure that the Request for Proposal provides for a clear procurement timetable and defined periods where bidders will be informed of the status of the procurement process. Bidders shall be provided a fixed window within which to lodge complaints and a clear timetable for those complaints to be addressed.

6.6 ACTIONS ON APPROVAL OF EVALUATION REPORT BY THE PPP BOARD

6.6.1 INITIAL NOTIFICATION OF PREFERRED BIDDER

Prior to submitting the evaluation report to the PPP Board, the PPPDG will carry out a verification of qualification to confirm that the preferred bidder still possesses the adequate financial and technical qualifications presented in its prequalification submission and/or Proposal.

The PPPDG will contact the preferred bidder and in exceptional circumstances enter into negotiation with the support of the Contracting Authority and its PMT.

Even after bidders have commented on draft versions of the PPP agreement during the procurement phase, there might still be other important details that will need to be resolved and agreed upon before the final PPP contract can be signed between the Contracting Authority and the private partner. These can include updating cost figures according to clear cost escalation formula and asset condition following pre-handover inspection. During this process, it is important that the PPPDG not allow the proposed value for money gains that have been achieved through the competitive pressures of open tendering to be eroded (or transferred back onto the Government) by the preferred bidder. The PPPDG can achieve this objective by:

- Providing a detail list of items to be negotiated with the private partner and inviting the private partner to do the same. Items shall not include any general or specific clauses in the agreement, unless items were marked during the procurement phase as to be concluded post-award
- Working with the Transaction Advisor to prepare adequately for the negotiation. A pre-negotiation workshop shall be carried out with the Contracting Authority adopting a position on each item proposed to be raised by the private partner
- Continuously assessing the economic advantage of the Preferred Bidder's offer throughout the negotiation. If the value of the offer falls below the proposition of the

Reserved Bidder, then the Contracting Authority shall communicate this depreciation in value to the Preferred Bidder. The Preferred Bidder can re-then examine and improve its offer during the negotiations to maintain the economic advantage of its offer.

BEST PRACTICE

Best practice suggests that bidders be invited to provide complete comments on the PPP agreement during the tender procurement stage. If this procedure is not implemented, then it is much more likely that the preferred bidder will request more changes and modifications during negotiations to finalize the PPP contract after the award decision. Usually, such negotiation can include the preferred private bidder seeking to address any uncertainties in the contract by insisting that the Government accept responsibility for more of the project's risks. Therefore, implementing this procedure during tender procurement stage can also shorten the time required to actually sign PPP contracts after the preferred bidder has been selected.
The PPPDG should avoid requests by the preferred bidder to "re-open" project issues to negotiation and to consider material changes to the scope and risk allocation structure of the project. This could lead to effectively removing the important economic benefits that the original PPP project and the successful bidder's bid offered to the client Government.

If negotiations take longer than expected, the Government has the option to either continue the negotiations, if it believes that real progress is being made with the preferred bidder, or it may declare the PPP contract negotiations unsuccessful. If negotiations are unsuccessful, the Government may choose to begin negotiations with the second-ranked bidder, or it may evaluate whether there are fundamental problems with the structure, completeness, and quality of the project and its PPP contract that could require restructuring and retendering the project.

The PPPDG will ensure that the final terms, conditions, risk transfer and any rates, tariffs or fees are affordable for the Government of Ethiopia.

Estimated Time Required: The amount of time required to carry out negotiation depends on the nature, the complexity, and especially the perceived risk exposure and allocation structure of the specific project. For the larger, more complex, or "riskier" PPP projects, this procedure may take up to 4 – 6 weeks to complete. However, for smaller, simpler, or less risky projects, this procedure may only need 2- 3 weeks to complete.

6.6.2 RE-EVALUATING THE GOVERNMENT'S FISCAL COMMITMENT

Section 4.1.4 introduced the concept of Fiscal affordability. PPPs typically create fiscal obligations for the Government, which can in some cases be similar to those arising from traditionally-procured projects financed by Government debt. PPP fiscal obligations may be direct—that is, where the payment need is known (availability payments, subsidies, grants, etc.) —or contingent—where the occurrence, timing, and magnitude of a payment depends on some uncertain future event (guarantees for specific risks, termination payments, etc.). The Government of Ethiopia is committed to responsible management of its fiscal commitments arising from PPP projects. This includes identifying and appraising the fiscal implications of all proposed PPPs and ensuring these are in line with fiscal priorities.

At the feasibility stage an estimate will have been carried of the impacts the PPP project will have on Fiscal affordability over the life of the project. This resulted in a Fiscal Affordability report being produced (see **Appendix C – Part 3**) for the MOF Debt Management Directorate for assessment and clearance to proceed as part of the PPP Board approval of the Feasibility report and PPP structuring.

During this pre-award phase, the Contracting Authority and the Ministry of Finance need to monitor the evolution of its direct and contingent liabilities under the project's contracts through post award resolution of minor issues and negotiations.

It should be remembered that in the case of PPPs, the fiscal commitments of the Government can be real or conditional:

- A direct commitment is "a quantifiable and scheduled payment by the government to the private sector that flows from a negotiated clause in the PPP contract"
- A contingent commitment is "an obligation that may give rise to a payment in the future" and it is conditional on how certain events transpire.

The difference between direct and contingent is the degree of probability of the obligation to pay. Among the direct commitments, we find (i) direct support (in cash or in kind), (ii) waiving fees, taxes or other payments to the private party, (iii) subsidies for viability gap funding and (iv) availability based payment. Among the contingent liabilities, there are guarantees and financial obligations in specific events, for example:

- guarantee to third parties (banks and other lenders) on the payment of the debt contracted by the private partner to undertake the PPP project;
- payments due to provisions such as minimum revenue guarantees and take or pay provisions
- guarantee to the private partner regarding the minimum income it will receive from the PPP project (which may lead the Government to pay a certain sum if the private partner fails to realize the revenue in question); and
- obligations resulting from the early termination of the PPP contract, including its termination due to a force majeure event.

Significant changes from the impacts identified at the feasibility stage are required to be passed on to the Debt Management Directorate for assessment and clearance (see Fiscal Affordability report form in **Appendix E – Part 4**) as part of the process for PPP Board approval of the preferred bidder.

The Fiscal Affordability discussion provided an indication of the process undertaken by the Debt Management Directorate in assessing and monitoring PPP impacts. The Contracting Authority and the Ministry of Finance with its Debt Management Directorate will implement a process for the reporting, budgeting and accounting for these liabilities for each PPP contract into the annual reports, budget and public accounts.

6.6.3 BOARD APPROVAL FOR PROJECT AWARD

Upon successful negotiation of the PPP agreement and review of the project's fiscal affordability, the PPPDG will forward the final negotiated PPP Agreement along with the bid

evaluation report to the PPP Board for approval. In its report to the PPP Board, the PPPDG will outline:

- The objectives of the PPP process
- The Value for Money proposition of the Preferred Bidder
- Government non-fiscal obligations under the PPP Agreement
- The total sum of any fiscal commitments by the Government of Ethiopia under the PPP
- The Key Performance Indicators for the private partner under the PPP agreement
- Any changes to the PPP structure during the tender process

The PPP Board will review the PPPDG's report and the final negotiated PPP Agreement and confirm that it represents Value for Money (VfM) for the Government of Ethiopia.

Following this review, the PPP Board may communicate its approval of the evaluation report and the PPP Agreement.

Once the evaluation report has been approved by the PPP Board, the PPPDG will officially notify the bidders of the result.

6.6.4 PROJECT AWARD

The PPPDG will officially notify the preferred bidder upon approval of the evaluation report by the PPP Board and prior to the expiry of the bid validity.

The process for notifying bidders of the outcome of the tender process will follow procedures outlined in the Request for Proposal for the PPP project. Generally, these procedures would include:

- Written correspondence to losing bidders confirming an award of the PPP to the winning bidder. The communication shall include:
  - → The name of the winning bidder
  - → The duration of the PPP term
  - → The output specifications for the PPP term
  - → Grounds for selection
  - → Right to appeal the award decision
- Written correspondence confirming as 1st Ranked Bidder, the bidder submitting the most economically advantageous offer

- Written correspondence confirming as 2nd Ranked Bidder, the bidder submitting the second most economically advantageous offer
- In the correspondence to the 1st Ranked Bidder, the Contracting Authority will indicate the actions the 1st Ranked Bidder must take, to be nominated the Preferred Bidder. Usually this will include the presentation of a valid Preferred Bidders Guarantee.
- The template, including language, for the Preferred Bidder's Guarantee must be included in the Technical Proposal forms included in the Request for Proposal for the PPP.
- The Preferred Bidder's Guarantee differs from the Proposal Bond or Guarantee in that it underwrites the 1st Ranked Bidder's commitment to work in good faith to complete negotiations with the Contracting Authority. If the Preferred Bidder walks away from negotiations, not as a result of a failure to come to terms with the Contracting Authority, the Contracting Authority can call the guarantee and receive compensation. If the Contracting Authority and the 1st Ranked Bidder fail to agree to terms on the PPP agreement, then the 1st Ranked Bidder's Preferred Bidder's Guarantee is returned.
- The conditions under which the Contracting Authority can call the Preferred Bidder's Guarantee have to be specified in the guarantee instrument.

The PPPDG will also send the PPP Agreement to the Contracting Authority within two days from the day of the award and authorize the signing of the PPP Agreement by the Contracting Authority.

The Project Agreement cannot be signed before seven days from the date bidders are notified of the result of their bid to enable unsuccessful bidders to submit a complaint. The procedure to follow for complaints is detailed in section 6.5.10.

6.6.5 ESTABLISHMENT OF PROJECT COMPANY AND PPP AGREEMENT SIGNING

The Project Agreement will be executed by the Project Company that will fulfil the project and the Contracting Authority. Clearly the preferred bidder (lead consortium member) will have to establish the Project Company, in which a Public Entity may be a minority shareholder, and confirm its formation and membership (in accordance with the tender and all documentation submitted). The Project Company, incorporated under the laws of Ethiopia, is a legal entity with no activity other than those connected with its borrowing and whose sole purpose is to implement the PPP Agreement. It is understood that company formation in Ethiopia will take around two months and this timing will have to be included in the plan for contract finalization. Registration and permits are obtained with the Ministry of Trade and the Ethiopian Investment Commission. The Private Party is accompanied in this process by the Contracting Authority with the support of the Ministry of Finance. In addition, the PPPDG will need to confirm the Project Company is established in accordance with the bid and relevant bid bonds are effected and valid.

The legal framework[64] indicates that the PPP Agreement may:

- Establish requirements around the share capital of the Project Company;
- Require approval of changes to by-laws and shareholders' agreement and other related agreements of the Project Company;
- Impose restrictions around the transfer of ownership interests, such as shares,

Similarly, the PPP Proclamation (article 31(4)) indicates that except as otherwise provided in the Project Agreement, the transfer of any ownership interests in the Project Company, which would result in a change of control of the Project Company will need the consent of the Contracting Authority and the PPPDG. The conditions requiring the consent of the Contracting Authority and the PPPDG for the transfer of assets will be included in the Project Agreement.

The preferred bidder's bond must be valid during Project Company establishment process. Once established, the Project Company will sign the PPP Agreement with Contracting Authority. After the signature of the PPP Agreement, the PPPDG will publish the result of the tender on the Ministry Finance's website and in national newspaper and indicate:

BEST PRACTICE TIP

Consultation with the Ministry of Finance
Approval of MoF of the PPP Agreement before signature of the contract should be required to confirm project is fiscally affordable.

- the purpose of the contract,
- its duration,
- the name of the parties,
- the fee structure and
- the main characteristics of the contract.

The PPPDG shall use reasonable efforts to publish the same information on its and the Ministry of Finance's website.

[64] Article 31(2) of the PPP Proclamation

Figure 6-3: Importance of the Project Company in the PPP Process

PPP Tender

Government announces that it intends deliver a project by way of a PPP contract. Government prepares a draft PPP contract detailing the outcomes it is wanting to achieve, and the risk allocation it is wanting to achieve, and calls for tenders from private sector for the award of the PPP contract.

The PPP contract typically requires the counterparty to design and construct the infrastructure facility and then to operate and/or maintain it, and perhaps provide other services, over a relatively long period (typically between 15 and 30 years).

For service–payment PPPs, the PPP contract will require the government to pay a monthly or quarterly service payment commencing when construction is completed and the provision of the infrastructure enabled services commences. For user–charge PPPs, the PPP contract gives the counterparty the right to levy specified tolls or other charges on users of the infrastructure once construction is complete. Either way, the PPP contract typically requires the counterparty to raise private sector finance to cover all or most of the project costs that it incurs before receiving the government service payment or user charges.

Bidding consortia

As PPP contracts typically require the counterparty to provide a range of skill sets that are typically beyond the capabilities of any one company, companies that can provide one or more of these skill sets team up with other companies that can provide the balance of the required skill sets to form a consortium to bid for the PPP contract. The composition of a bidding consortium will depend on the nature of the PPP contract and the skill sets it requires, but a typical consortium will include a design and build (DB) contractor, an operations and maintenance (O&M) contractor, equity investors and debt financiers.

Project Company Establishment

The successful bidding consortium establishes a Project Company to become the counterparty to the PPP contract with the government. The Project Company will also enter into a Construction contract with the Construction contractor, an O&M contract with the O&M contractor, equity contribution agreements with the equity investors and a loan facility agreement with the debt financers.

The Project Company uses the debt and equity that it raises from the debt financers and equity investors to pay the monthly progress payments payable to the Construction Contractor under the Construction Contract. Once construction is completed, the O&M contractor commences provision of the operation and/or maintenance services to enable the Project Company to either provide the required services to government and earn the service payment (in the case of a service-payment PPP) or to provide the required services to users and earn the user charges (in

The Project Company then uses the service payment or user charges to pay the fee payable to the O&M contractor under the O&M Contract, and to repay the debt financers in accordance with the loan facility agreement. If the Project Company's revenues and costs are as forecast, its revenues should exceed its costs so that it can distribute the surplus back to the equity investors as a return on their equity investment.

Source: Reimagining Public Private Partnerships Oct 2017 PwC adapted by the consultant

Estimated Time Required: 1½ -3 months to establish Project Company

6.7 OTHER PRIVATE PARTY SELECTION METHODS

The PPP framework identifies five procedures by which private parties may be selected for the implementation of PPP projects. These are presented below:

1. Open Bidding Process
2. Two-Stage Bidding
3. Competitive Dialogue
4. Direct Negotiations
5. Unsolicited Proposals

Sections 6.3 to 6.6 above describes the procedures for an Open Bidding Process. The next table presents a matrix showing the difference in procedures for each of the other selection methods (with the exception of unsolicited proposals).

	Launch of Tender Process	Management of Prequalification	Management of RFP Process	Approval of Evaluation	Commercial Close
Single-Stage Bidding Process	▪ Publicize GPN and REOI	▪ Issue RFQ<br>▪ Evaluate and shortlist Prequalification submissions	▪ Design and manage Bid Process<br>▪ Issue RFP to shortlisted bidders<br>▪ Receive and evaluate Proposals	▪ PPP Board approves Evaluation Report<br>▪ PPPDG negotiate with Preferred Bidder<br>▪ PPP Board approves award of PPP Project to Preferred Bidder	▪ Project Signing<br>▪ Fulfilling Condition Precedents
Two-Stage Bidding	▪ Publicize GPN and REOI	▪ Issue RFQ<br>▪ Evaluate and shortlist Prequalification submissions	▪ Design and manage Bid Process<br>▪ Issue **Round 1 RFP** to shortlisted bidders (Technical bid)<br>▪ Receive and evaluate Proposals<br>▪ Issue **Round 2 RFP** to shortlisted Bidders (Technical bid and financial bid)<br>▪ Receive and evaluate Final Proposals	▪ PPP Board approves Evaluation Report<br>▪ PPPDG negotiate with Preferred Bidder<br>▪ PPP Board approves award of PPP Project to Preferred Bidder	▪ Project Signing<br>▪ Fulfilling Condition Precedents
Competitive Dialogue	▪ Prepare and publicize **Contract Notice**	▪ Issue RFQ<br>▪ Evaluate and shortlist Prequalification submissions	▪ Design and manage Bid Process<br>▪ Issue Preliminary Contract Notice for **Round 1 Dialogue**<br>▪ Issue Draft Contract Notice for **Round 2 Dialogue**<br>▪ Issue Final Contract Notice for **Round 3 Dialogue**	▪ PPP Board approves Evaluation Report<br>▪ PPP Board approves award of PPP Project to Preferred Bidder	▪ Project Signing<br>▪ Fulfilling Condition Precedents
Direct Negotiations	▪ **Publicize GPN and REOI (if required)**	▪ **Issue RFQ(if required)**<br>▪ Evaluate and shortlist Prequalification submissions	▪ Design and manage Bid Process<br>▪ Prepare and issue Terms of Reference and PPP Agreements and evaluation criteria<br>▪ Evaluate Proposals and invite qualified Bidders for negotiations<br>▪ **Round 1 negotiations**<br>▪ **Round 2 negotiations**	▪ PPP Board approves Evaluation Report<br>▪ PPPDG negotiate with Preferred Bidder<br>▪ PPP Board approves award of PPP Project to Preferred Bidder	▪ Project Signing<br>▪ Fulfilling Condition Precedents

The key differences in selection procedure are described in the section below.

6.7.1 TWO STAGE BIDDING

The PPPDG shall indicate the selection method for the PPP in the General Procurement Notice/REOI or in the Request for Qualification. This is good practice and part of maintaining a proactive disclosure philosophy for PPPs. The Bid Process, irrespective of the selection method adopted, will require investments by Bidders. Bidders shall be informed about the potential selection method so can make an informed judgement as to whether to participate or not.

The procedures for Two-Stage Bidding are similar to those for Single-Stage Bidding process with the exception of the Request for Proposal stage where there is a requirement for the issuance of a Round 2 Request for Proposal. In a Two-Stage process, the PPPDG will issue RFPs in Two Rounds. The PPPDG shall prepare a Round 1 Request for Proposal and accompanying Evaluation Criteria.

Round 1 Request for Proposal. This procedure to develop this Request for Proposal will be similar those describe in Section 5.3.4. However, Two-Stage Bidding is adopted in instances where the PPPDG does not have sufficient information about the project specifications (even after the delivery of the Project Feasibility) to allow prospective Bidders submit comprehensive proposals. In this instance, the Round 1 Request for Proposal will allow prospective Bidders respond to available project requirements and the indicative PPP structure. This initial submission will be reviewed following the same procedure for proposal evaluation under a Single-Stage Bidding Process. As required, the PPPDG may call Bidders to clarify their submissions.

Round 2 Request for Proposal. Following submission and review of Bidders' proposals to the Round 1 Request for Proposal, the PPPDG will issue a Round 2 Request for Proposal. This second Request for Proposal will include better defined project requirements and a revised PPP project structure in the PPP Agreement. The PPPDG will revise, update or delete elements of the Round 1 Request for Proposal and PPP Agreement to better reflect its expectations following the review of Bidder's proposals from the Round 1 Request for Proposal. The remainder of the bid process following the submission of proposals to the Round 2 Request for Proposal is similar to the process under the Single-Stage Bidding Process.

6.7.1.1 Procedures for Managing the Request for Proposal Rounds in Two-Stage Bidding

The PPPDG shall prepare a Bid Process timetable outlining the schedule for the Two-Stage bidding process. This timetable shall be included in the Request for Proposal for both rounds of the bid process. As with the Single-Stage Bidding process, the schedule will present a duration for each element of the bid process, including the Two Rounds of the Request for Proposal process. Other elements of the Request for Proposal document shall be consistent with the Request for Proposal for Single-Stage Bidding process:

- The PPPDG may hold Bidders Conferences or other meetings to clarify and/or answer Bidders' questions or comments on the Round 1 Request for Proposal
- The PPPDG can facilitate site visits, data rooms and other elements of the Bid Process

Bidders' proposals submitted to the Round 1 Request for Proposal shall be evaluated in the same manner as under the Single-Stage Bidding Process. In this evaluation process, the PPPDG shall score Bidders in accordance with the evaluation criteria presented in the Round 1 Request for Proposal. The objective of the scoring process is to identify the strong elements in each proposal. The PPPDG will then develop the Round 2 Request for Proposal and PPP Agreement borrowing from the strengths of each of the earlier submitted proposals.

The PPPDG shall issue the Round 2 Request for Proposal and PPP Agreement at the pre-set time indicated in the Bid Process timetable. The remainder of the Round 2 Request for Proposal phase follows the same process as in a Single-Stage Bidding process.

6.7.2 COMPETITIVE DIALOGUE

The PPP framework permits the PPPDG to use competitive dialogue in the selection process for a PPP Partner, when the PPPDG makes a determination that the Single-Stage or the Two-Stage Bidding process is not appropriate.[65] The PPPDG shall use the competitive dialogue in place of the Single-Stage bidding or Two-Stage bidding process in instances when it determines that:

- There are several potential PPP solutions for a service, each confirmed by the feasibility study as potentially offering Value for Money
- A Bid Process will require multiple rounds between the PPPDG and prospective bidders to arrive at an optimal outcome
- Two or more prospective PPP solutions may offer the same Value for Money but require different risk allocations

The PPPDG can use the competitive dialogue process to identify a PPP project structure that offers Value for Money, presents a bankable project for the Private Party and assigns to the Contracting Authority the risk it is best able to manage. In a competitive dialogue process, the PPPDG intends to maximize the value to the public sector and this means maximizing Value for Money while minimizing risks assigned to the Contracting Authority.

A Competitive Dialogue process follows the same general procedures as a Single-Stage or Two-Stage Bidding Process. However, unlike the Two-Stage process, in a Competitive Dialogue process, the PPPDG can invite Bidders to submit proposals in several rounds, sufficient to allow the PPPDG to define the project specifications, outcomes and a PPP structure (described in a PPP Agreement) that meets the needs of the PPPDG. Competitive Dialogue is therefore less formal than the Two-Stage process with several stages of discussion with bidders.

6.7.2.1 Procedures for Managing the Request for Proposal Rounds in Competitive Dialogue

Unlike the Single-Stage or Two-Stage processes, the Competitive Dialogue process is not time-bound. The PPPDG can have as many dialogues with individual Bidders as is necessary to arrive at a project solution that addresses the Contracting Authority's requirement.

[65] PPP Proclamation Article 31 (1)

The PPPDG shall commence the Competitive Dialogue process by presenting a schedule of proposed transaction activities. The schedule will indicate the commencement of the Competitive Dialogue Process and the sequence of activities and procedures that will follow upon the completion of the dialogue part of the process.

An indicative schedule is presented below.

	**Bid Process Events**	**Date(s)**
1.	Issue Contract Notice and Draft PPP Agreement	
2.	Commence Dialogue	
3.	Start of Site Visits	
4.	End of Dialogue	*TBD*
5.	Issue Final Contract Notice and PPP Agreement	
6.	Invite Final Proposals from Bidders	
7.	Clarification Period Ends	
8.	Proposal Submission Deadline	
9.	Announcement of Proposal Evaluation Result	
10.	Clarification of Winning Proposal	
11.	Award of Project	
12.	Execution of PPP Agreement	

The bid process timetable shall have a firm date for each of the items in the bid process, with the exception of the date for completion of the dialogue.

The PPPDG commences the Competitive Dialogue process by issuing a Contract Notice, which shall be structured as a PPP project Terms of Reference outlining the requirements of the PPP. The PPPDG shall also issue a Draft PPP Agreement that Bidders can review/comment and modify with the objective of presenting a PPP solution that address the Contracting Authority's requirements.

The PPPDG shall hold as many discussions with prospective bidders as necessary until it is reasonably satisfied that it has arrived at a project specification and design of PPP Agreement that addresses the Contracting Authority's needs.

In the dialogue with Bidders, the PPPDG shall be careful not to share information in proposals with competing bidders. The PPPDG shall accomplish this by structuring the dialogue as a series of question and answer sessions.

1. Bidders shall present written questions on the Contract Notice of PPP Agreement to the PPPDG.

2. Bidders may also present written proposals on the PPPDG's Contract Notice and PPP Agreements.
3. At any time during the dialogue, the PPPDG may modify an element of the Contract Notice or PPP Agreement and make the information available to all competitors in the dialogue process.
4. When the PPPDG is satisfied that the Contract Notice and PPP Agreement sufficiently reflects the requirements of the PPP service, it will declare the dialogue concluded.
5. The PPPDG shall request Final Proposals from all competitors on the dialogue. The proposals shall be based on a Final Contract Notice and PPP Agreement to be issued by the PPPDG following completion of the dialogue. The Final Contract Notice shall include an evaluation criteria for proposals to be submitted.
6. Bidders may, for a period of time, request clarification on any element of the Contract Notice and PPP Agreement.
7. All Bidders shall submit their final proposals at a predetermined deadline indicated in the Bid Process timetable.
8. The PPPDG shall review the proposal submissions utilizing the same process as described in Section 6.5.9.
9. Following proposal evaluations, the PPPDG shall declare as Preferred Bidder, proposal offering receiving the highest score according to the evaluation criteria.
10. The PPPDG may clarify elements of the winning proposal, but no negotiations of the proposal of PPP Agreement are permitted by the legal framework
11. The PPPDG will presents its evaluation report to the PPP Board, with its recommendation to award a PPP contract to the Preferred Bidder.

6.7.3 DIRECT NEGOTIATIONS

Direct negotiations, also known as sole sourcing, directly sourced, non-competed contracts, single invited etc., usually do not undergo a competitive process.

Similarly to other bidding procedures, Direct Negotiations are also a lengthy and complex procedure. The PPPDG may adopt a Direct Negotiation selection process if the following conditions are met:

- Where there is an urgent need and none of the other selection methods would be practical. Direct Negotiation may be adopted for example when there is a need for rapid procurement in the case of emergencies and where quickness may exceed value for money considerations. However, Direct Negotiation, similarly to other procurement routes, a structured approach with different steps and may not be substantially faster than open bidding.
- Where the PPP service being procured is of a duration five years or shorter.

- Where the project involves infrastructure projects related to national defence or national security
- Where there is only one source capable of providing the PPP solution
- When an earlier open competitive selection process did not result in credible bids or any bids at all. When there is good reason to believe there would be no market interest, then Direct Negotiation can be considered.
- When the proposed PPP service is ancillary to an industrial activity by an incumbent in a sector and that service must be carried out by the current incumbent in order to realize any economic advantages from that service

Being a non-competitive process may drive up costs. If the private party is known by the Contracting Authority/PPPDG and if these parties have worked together previously there may be advantages that lead to a fast procurement. However if Direct Negotiation is being undertaken for any other reason, the process will involve detailed work and may prove to be a lengthy process

The PPPDG may publish a General Procurement Notice/EOI and carry out a prequalification if it makes the determination that this activity is required. The PPPDG must follow the procedures described in Section 5.3.2 to prepare the Request for Qualification.

The PPPDG with the support of the Contracting Authority and its PMT shall prepare the Terms of Reference and a Draft PPP Agreement outlining its expectations and needs for the PPP Service. The PPPDG shall invite prequalified bidders (if bidders were shortlisted) to submit proposal in response to this Terms of Reference and Draft PPP Agreement. The Terms of Reference will include a detailed Bid Process timetable, and clear instructions to bidders about how the Direct Negotiations would be conducted. Following submission of Proposals, the PPPDG shall:

1. Evaluate proposals received and may call specific bidders to review and negotiate elements of their proposal. Bidders will be allowed to resubmit their revised proposals.
2. Bidders who do not submit proposals that meet the PPPDG’s requirement will be eliminated from the Direct Negotiation process.
3. The PPPDG shall carry out this process until only one proposal remains.
4. The PPPDG will prepare an Evaluation Report for the PPP Board with a summary of the merits of this proposal along with a recommendation to award a PPP.

6.8 FINANCIAL CLOSE

6.8.1 PROCESS OVERVIEW

Financial close occurs when all the project and financing agreements have been signed and all the required conditions contained in them have been met. It enables funds (e.g. loans, equity, grants) to start flowing so that project implementation can actually start.

The Private Party will provide the financial agreements to the Contracting Authority for validation and to check that it (i) is aligned with the provisions of the PPP Agreement and (ii) does not change the risk allocation and the responsibilities of the different parties.

BEST PRACTICE
Reaching financial closure for larger and complex PPPs is a very demanding process.<br>The activities involved in these steps often deal with detailed fine-tuning matters. Close interaction between the Contracting Authority, the Project Company, its sponsors and its financiers is essential. This stage requires thorough organisation and management for it to proceed efficiently. It should be planned carefully, generally making use of experienced advisers. Many PPP projects have experienced difficulties at a later stage as a result of a lack of adequate planning or expert advice during this critical stage.<br><br>The consequences of private contractor not reaching financial closure are very costly for both parties. While the private contractor may have to forfeit their bid bond, the implementation of the planned project can be greatly delayed or cancelled altogether.

6.8.2 ACCOUNTING/BUDGETING

6.8.2.1 Budgeting for Direct Commitments and Contingent Liabilities

Budgeting for Short-Term Direct Commitments

If the Government of Ethiopia is required, as per the PPP Agreement, to provide capital subsidies to the private party, it will provide the payments usually during the first years of the project. These payments can therefore be integrated within the annual budget and medium-term fiscal plan of the Ministry of Finance. If these payments are financed through loans, then interest and loan repayments should also be integrated with the budget.

Budgeting for Long-Term Direct Commitments

Long term direct commitments, such as availability based payments, imply multi-year payment commitment by the Government of Ethiopia. This dichotomy between an annual budget and payments commitments which go beyond the fiscal year might create a risk of non-payment to the private party. As such the Ministry of Finance is responsible for the approval of appropriations as per the requirements of the PPP Agreement. The Ministry of Finance could also decide to treat subsidies to PPPs similarly to debt service payments, which means that they are systematically appropriated.

Best practice requires for the Ministry of Finance to include long-term direct commitments in its medium term fiscal plan. Additionally, the Ministry of Finance will establish a register for all of the Government's PPP commitments (direct and contingent) in order to centrally monitor them.

Budgeting for Contingent Liabilities

Since the occurrence of contingent liabilities is not certain, budgeting for them can be difficult. When a contingent liability occurs, if there are no availabilities within the appropriations of that fiscal year, the Contracting Authority will be required to request for additional appropriation, which can be complex and difficult.

In order to avoid this contentious situation, two options are available:

1. The Ministry of Finance could create a PPP contingency line in the annual budget dedicated to PPP contingent liabilities. This flexibility in the budget will help cover unexpected payments.

2. A contingent liability fund could be established by the Government to which contributions are made from the Federal Budget, Public Entities and the returns generated, and from which payments for contingent liabilities will be made.

The MOF Budget Preparation and Administration Directorate and the PPPDG will consult, as relevant, the MOF Debt Management Directorate, on the level of such provisions, taking into account overall perceived risks from the PPP portfolio of projects.

6.8.2.2 Fiscal Accounting of PPPs

International standards for accounting and reporting on fiscal commitments from PPPs focus on both direct and contingent liabilities. These standards focus on the treatment of PPP liabilities in national accounts.

Direct Contributions

PPP projects are typically not reported in national accounts for countries using cash based accounting systems[66] where revenue is reported on the income statement only when cash is received and expenses recorded when cash is paid out. Under cash based accounting systems liabilities are not reported. Ethiopia uses a modified cash basis of accounting where revenue is recognized on receipt and expenditure recognized on a cash basis during the year, but accrued at the end of the year for the annual statements.

However, International accounting standards such as the International Public Sector Accounting Standards (IPSAS) Standard 32 provide guidance for countries implementing accrual accounting. Under this standard, PPP assets and liabilities are reported on national accounts provided[67]: (1) the government controls or regulates the services the operator must provide with the PPP asset,

---

[66] As opposed to accrual based accounting which records revenue when it is earned and expenses when they are incurred regardless of when cash is exchanged.

[67] PPP Reference Guide version 3, World Bank (2017)

to whom, and at what price; and (2) the government controls significant residual interest in the asset at the end of the contract. Using this standard, “government-pays” PPPs would appear on the national accounts. On the other hand the treatment of “user-pays” PPPs is less clear, and depends on the details provided in the PPP Agreement.

Contingent Liabilities

Accounting and international reporting standards do not require liabilities to be recognized in public debt until the contingency has occurred and payments need to be made.

Even though Ethiopia uses a modified cash basis accounting system which does not require PPP liabilities to be recognised in national accounts, it is considered best practice for the Ministry of Finance to disclose PPP direct and contingent labilities in national accounts and budget documentation.

6.8.3 ROLE OF LENDERS, POTENTIAL NEED FOR DIRECT AGREEMENTS

Private capital will have to come through a combination of equity and debt. Equity refers to the paid-capital by investors and debt refers to the loans from banks. It is to be noted that there are limitations to available capital for PPP in Ethiopia. So the Public Entity proposing the PPP needs to provide some indication of the availability of private capital including non-recourse financing. Some informal sounding of financial institutions is encouraged.

The process of negotiating the projects financing agreements is done by the private contractor and by the lenders that they decide to try to borrow from for the project. Therefore, this is not the direct responsibility of the Contracting Authority, or of the PPPDG. However, the Contracting Authority and the PPPDG have a clear interest in seeing that these negotiations between private investors and lenders has a successful and timely outcome.

Therefore, there are certain, limited actions that Government can take to help facilitate this process when it is needed.

Table 6-1: Actions to take by the Contracting Authority and the PPPDG to facilitate the process of projects financing agreements negotiation

Actions	Description
**Making Information Readily Available to Lenders**	One specific thing that the Contracting Authority, the PPPDG, and their PPP Transaction Advisors can do is to stand ready to respond to requests from lenders for additional information about projects. Typically lenders will insist on hiring their own advisors and sector experts to carefully verify all background information about a project as part of carrying out their own due diligence. The Contracting Authority and the PPPDG can facilitate this process by making all relevant sources of information readily available to lenders as needed.
**Requiring Regular Progress Reports from the Private Partner**	The Contracting Authority shall ask for regular progress reports from the awardees on their project financing negotiations. If after the first 3-6 months it appears that little actual progress has been made, and that there is little likelihood of a successful outcome by waiting another 6 months, Public Authorities may consider options for mutually agreeing to terminate the contract, or to consider awarding the contract to the second ranked bidder.

Actions	Description
**Managing Instances of Failure to Reach Financial Closure**	If, the private contractor is unable to reach financial closure within the time limit set by the Government within the PPP contract, there are several options that the Government should evaluate. The Government can call the preferred bidder bond that the contractor has posted. However, the Government should carefully evaluate the real reasons as to why financial closure was not reached. These could include:<br>▪ The selected private bidder did not have prior experience in actually raising project-backed<br>▪ financing for PPP projects and was thus unable to successfully negotiate with lenders,<br>▪ The project appeared too risky to lenders to finance,<br>▪ One of private sponsors of the new Project Company dropped out of the consortium and the rest of members decided they could not proceed in both financing and operating the project without them.<br>Failure to reach financial closure is costly experience for all parties. While private bidders risk losing their performance bond as well as writing-off the value of several years of preparation and development costs, the Government faces significant new delays in the provision of important new public facilities and services. Even lenders risk losing the value of their own time and expenses spent on due diligence and negotiating with investors.

In some instances, the lenders may require step-in rights to take over the project in the event that the Project Company is unable to execute its obligations under the PPP Agreement. The lenders will have the ability to take over the project and/or find a substitute entity to continue the project and try to remedy the breaches of the PPP Agreement. In such cases, the Contracting Authority agrees under a **direct agreement** that it will not terminate the PPP Agreement and will give a chance to the lender to resolve the issues in the implementation of the project.

The direct agreement, defining the step-in rights of the lenders, is signed between the Contracting Authority, the Project Company and the lenders. Step-in rights are allowed by the PPP Proclamation (article 57) which indicates that the Contracting Authority may agree with the lenders and the Private Party to provide for the substitution of the Private Party by a new entity or Person appointed to perform under the existing Project Agreements upon serious breach by the Private Party.

6.8.4 FOREIGN EXCHANGE

All foreign-currency related capital inflows (including foreign loans) must be registered at the Ethiopian Investment Commission (EIC). For projects entailing significant capital investments, the private-sector partner will typically access debt to finance a majority of the CAPEX and/or initial operations. Most lenders/financiers would require adequate insurance as a condition for lending. They would require that their interests are adequately protected, and the insurance framework provides appropriate protection to the Project assets and services

After registering any capital investment brought into Ethiopia with the Ethiopia Investment Commission, the Project Company will:

(i) Apply to the National Bank of Ethiopia to get authorization to open a foreign currency account with an authorized bank inside Ethiopia. The Project Company will transfer its foreign exchange into the account and use it to import capital goods, inputs necessary for its core business activity and other local payments as required.

(ii) To acquire a loan from abroad, the Project Company has to present the draft loan agreement to the National Bank of Ethiopia and get prior consent in line with the directives set by the National Bank of Ethiopia. The Project Company will then have the obligation to register the loan acquired with the National Bank of Ethiopia by attaching the required documentation.

OPENING AN OFFSHORE ACCOUNT FOR THE PROJECT COMPANY

- Some lenders may require the sponsors of the Project Company to maintain banking relationships in the country where the lender is domiciled in order to provide banking services that may not easily be available from domestic banks in Ethiopia.
- Foreign Lenders may sometimes require that receipts from on-going operations be escrowed in offshore accounts in politically and economically stable jurisdictions with lesser risk of political turmoil
- Offshore banks may sometimes offer the Project Company banking services with a lower cost base and potentially provide favorable interest rates than the acceptable standards in Ethiopia.

According to Ethiopia's Investment law, foreign investors are allowed to repatriate foreign currency from Ethiopia for:

1. profits and dividends accruing from investment;
2. principal and interest payments of external loans;
3. payments related to technology transfer agreements;
4. proceeds from the sale or liquidation of an enterprise;
5. proceeds from the transfer of shares or of partial ownership of an enterprise to a domestic investor; and
6. compensation paid to an investor under the investment laws.

The Project Company shall request approval from the National Bank of Ethiopia to remit the foreign currency abroad by attaching the necessary required documents, as per the National Bank of Ethiopia's directives

In case the Project Company is engaged in the export of goods and services, the foreign currency shall be fully repatriated to the country through the banking system. The Project Company can open a foreign currency retention account and retain fund, in line with the directives set by the National Bank of Ethiopia, from the foreign currency earning in foreign currency and utilize the fund for import of goods and service payment that is directly related to the export business.

Discussion on foreign currency risks and mitigation measures can be found in sections 4.1.11 and 4.1.13.

6.8.5 MANAGEMENT OF GOVERNMENT CONDITION PRECEDENTS

PPP Agreements may specify the conditions precedent that are to be fulfilled by the Government including:

- Confirmation of the legality of the procurement;
- Approval of derogations from any standard contracting terms;
- Confirmation of Government support
- Verification that post award qualification check has been carried out;
- Land, buildings, office space, or other existing assets needed for the installation of the new project may need to be made available by the Government prior to the PPP tender. The Contracting Authority or other Public Entity under the terms of the law and the PPP Agreement shall make available to the Private Party or, as appropriate, shall assist the Private Party in obtaining land rights related to the project site (article 49 of the PPP Proclamation).
- Retail tariffs for certain services may need to be raised from their current levels to a new baseline level that is at or near cost-recovery before a PPP in the sector could become viable and sustainable.
- Resolution of issues about the legal ownership of individual assets and equipment related to the new PPP project. In some cases existing assets, equipment, and public facilities are co-owned by mixtures of local governments, public enterprises, and ministries.

6.8.6 MONITORING OF PRIVATE PARTY CONDITION PRECEDENTS

Conditions Precedent are to be fulfilled by the project company before drawing on the debt, or before project contract becomes effective include:

- Proof that the selected bidder has incorporated a new project company in Country to both sign the PPP contract and to undertake the PPP project
- Provide a performance guarantee or bond to Contracting Authority to ensure it delivers its performance obligations throughout the term of PPP contract
- Provides a final design plan to Contracting Authority for review by the Independent Engineer prior to construction(who provides an unbiased and objective evaluation of the design plan)
- Demonstrated proof that the Project Company has reached financial closure with its lenders and therefore has the financial resources to both construct and to operate the new project

- Proof that the private contractor has received all of the permits and licenses that it requires to both construct and operate the project
- In the case of a USP that is being implemented as a PPP, if the successful bidder is not the proponent, the successful bidder should demonstrate proof that it made the financial compensation to the proponent
- Carry out full ESIA and get clearance from the relevant line Ministry or EFCCC, if not done at the feasibility study stage
- Land acquisition by the Project Company. If the lands currently have structures on them or habitants living on them, they shall be relocated and compensated by the private party. This Condition Precedent could lead to delays in achieving Financial close – delay in agreeing compensation for land for construction/development.

Figure 6-4:Closing the PPP Transaction

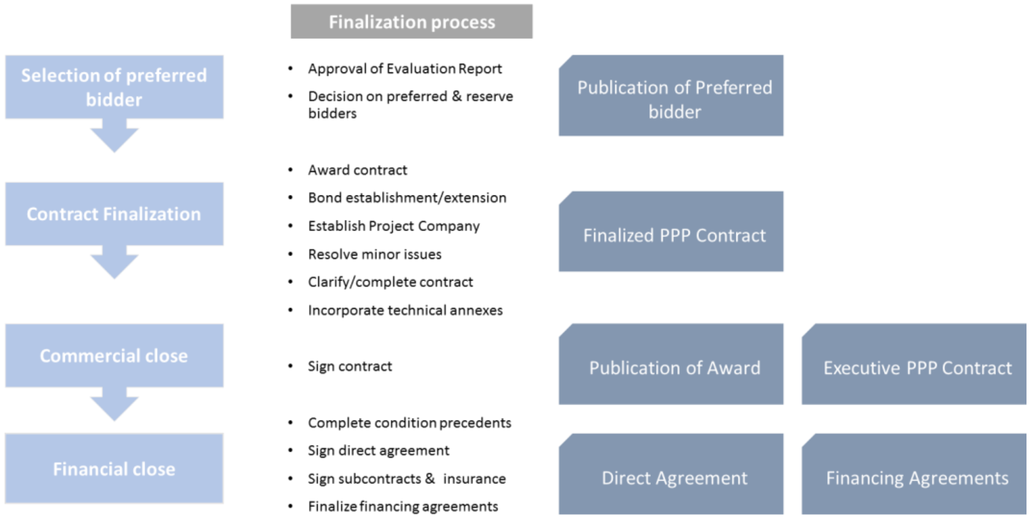

Estimated Time Required to reach financial close varies from 2 to 6 months.

6.9 NEXT STEPS

6.9.1 STRENGTHEN THE CONTRACTING AUTHORITY'S PPP PROJECT MANAGEMENT TEAM

In all PPPs, the public sector always remains ultimately responsible for regulating the services being provided within the sector, regardless of whether the delivery of services is being provided by public or private sector providers. Without this function, it would not be possible to accurately determine if the original goals and the contracted requirements of the PPP project are actually being achieved.

The legal framework provides for the functions of monitoring the contract are to be performed by the Project Management Team[68]. After the end of the procurement process, the Contracting Authority can supplement, adjust the staffing of the Project Management Team for it to fulfill its new role of PPP contract management.

The Project Management Team, on behalf of the Contracting Authority will keep an electronic and physical record of the PPP Agreement and all other relevant documentation from phase I to IV of the PPP cycle.

BEST PRACTICE

In practice, most private service providers prefer "regulation-by-contract" strategies instead of regulation by commission. Regulatory commissions place more discretionary powers in the hands of a panel of local commissioners or board members. Contract monitoring units, however, generally ensure the contract's key payment formulas are followed when approving requested tariff adjustments in the future. Such tariff formulas generally identify which costs may be "passed-through" to the client ministry. These may include:

- Changes in inflation, such as tracked by a Consumer Price Index (CPI)
- Changes in Foreign Exchange Rates and international energy prices, if applicable to the specific PPP project
- Changes in laws or regulations that can be shown to increase the private service provider's costs.

It is recommended to have the PPP contract management team designed and planned before the PPP contract is signed. Waiting until after the project has become operational is too late, and set the poor precedent that the PPP project is not really performance-based – as the private service provider would receive payment before its actual level of performance is verified by a monitoring unit.

In practice many PPP contract teams, especially for new pilot PPP projects, are tempted to request as much information as possible from private services providers to measure their performance. There is often an institutional concern that the new private service provider is secretly making money profits from public money, and therefore the monitor's mission should be to inspect every decision, activity, and expenditure to uncover how these hidden private returns are being made. As a result, PPP monitors often find themselves overwhelmed by the volume of data they must process and analyze. Generally, it is best to limit the performance reporting data required to only the most important measures of overall performance being delivered, and not to the details that might reveal how the private service provider's input decisions are being made.

[68] Articles 14(2-d), 14(2-f), 14(2-g), 14(2-h), 14(2-i) of the PPP Proclamation, article 10(6) of the PPP Directive

6.9.2 CLOSE-OUT REPORT OR CASE STUDY

The purpose of the close-out reporting is to summarize the activities carried out on the project, particularly the rationale underlying the specific structure for the PPP and its implication regarding post-contract monitoring.

The project close-out summary is intended to present a concise case study of the PPP project, highlighting key outcomes, decisions taken and lessons learned. The benefit of the close-out summary is informing future PPP transactions and improving the knowledge base of PPPs. The report, which will be prepared by the PMT, may be structured into the following parts:

- Summary of Project Deliverables: provide an overview on the integrity of tendering process, the evaluation, negotiations with the "preferred bidder", the award and contract signature.
- Key Decisions: provide the critical details from enforceable and effective contracts, any conditions precedent to be fulfilled, financial close structuring and any important details around construction, delivery and commissioning.
- Lessons Learned: review of the tender process to identify examples of good practices and areas of improvement in future projects. The PPPDG should share lessons learned, where relevant with the Contracting Authority and entities that may be undertaking PPPs. In some instances, it may be beneficial to publish a subset of the lessons to the public to better inform bidders on future projects.
- Outstanding Issues: as the project progresses to the construction stage, there could be additional risks that may have been identified, potential disputes, and potential changes in scope or in the service requirements which are still to be discussed.

6.10 DISCLOSURE OF INFORMATION AT PROJECT TENDER AND AWARD PHASE

At PPP project tender, the frequency of PPP project announcements may reduce as the PPPDG and prospective private bidders engage in the bid process. During this process, bidders are prevented by confidentiality agreements from talking public about projects. The PPPDG shall equally minimize its public communications until the outcome of the bid process is confirmed.

The PPPDG shall maintain a timetable of the bid process on its website so interested parties can follow the progress of the transaction. Events that are open to the public, such as road shows and market sounding events (can be publicized) and open to the general public. Any information shared at such venues shall be non-sensitive and not commit the government of private parties to an unconfirmed risk allocation.

In many emerging economics, the general public perceive PPP transactions as corrupt and a way for the civil service to divert State assets to political office holders or other Politically Exposed Persons (PEPs). The PPPDG must make all efforts to ensure that private parties for PPPs are not shell vehicles for PEPs. The tender documents must request detailed information (including shareholding information and names of directors) of all companies

that are bidding for projects. While the information collected cannot be grounds for disqualifying bidders, unless the tender documents expressly indicated that certain categories of bidders would be disqualified, the PPPDG should be aware of the identify of individuals behind prospective bidders for PPPs.

List of Documents Provided in Appendix E - Phase 4 Tender and Award
1. ***Part 1: Sample Confidentiality Agreement***<br>2. ***Part 2: Template RFP Evaluation Report***<br>3. ***Part 3: Notification Letter for Preferred Bidder***<br>4. ***Part 4: Fiscal Affordability Forms at Award / Commercial Close / Signature Approval Stage***

.

7 PHASE V: PPP CONTRACT MANAGEMENT & PERFORMANCE REPORTING

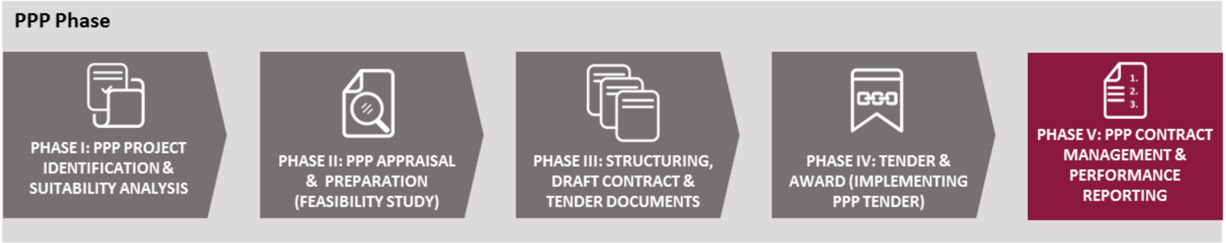

This chapter summarizes the important role of the Contracting Authority to actively monitor the performance being delivered by the private partner, both during the construction and operating phases of the contract. It includes managing financial close, future reviews and renegotiation and termination where applicable, and project closing. This process presents particular challenges as many infrastructure Public Entities have little proven experience.

Figure 7-1: Overview of Tasks under Phase V

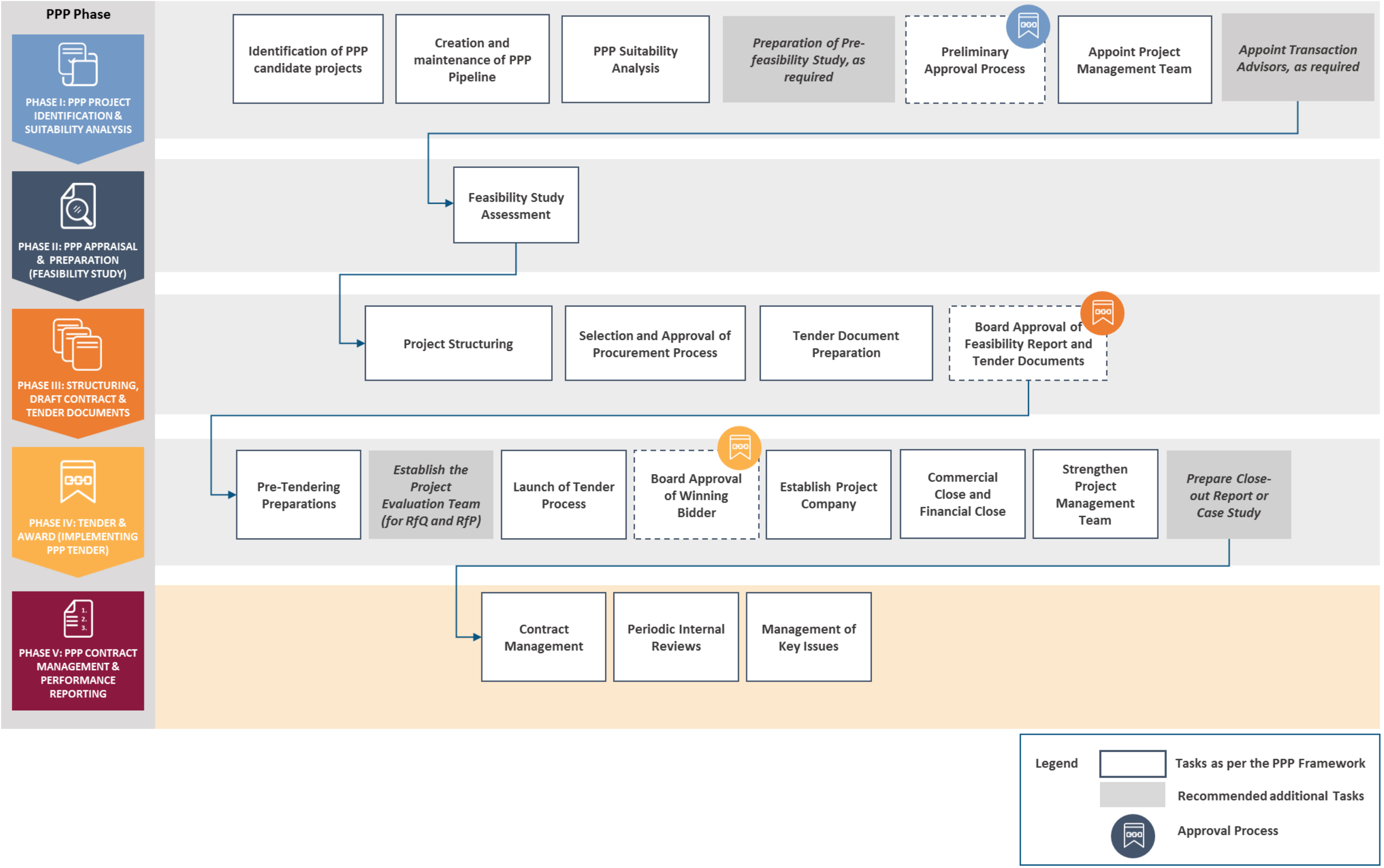

Figure 7-2: Phase V Process and Key Stakeholders

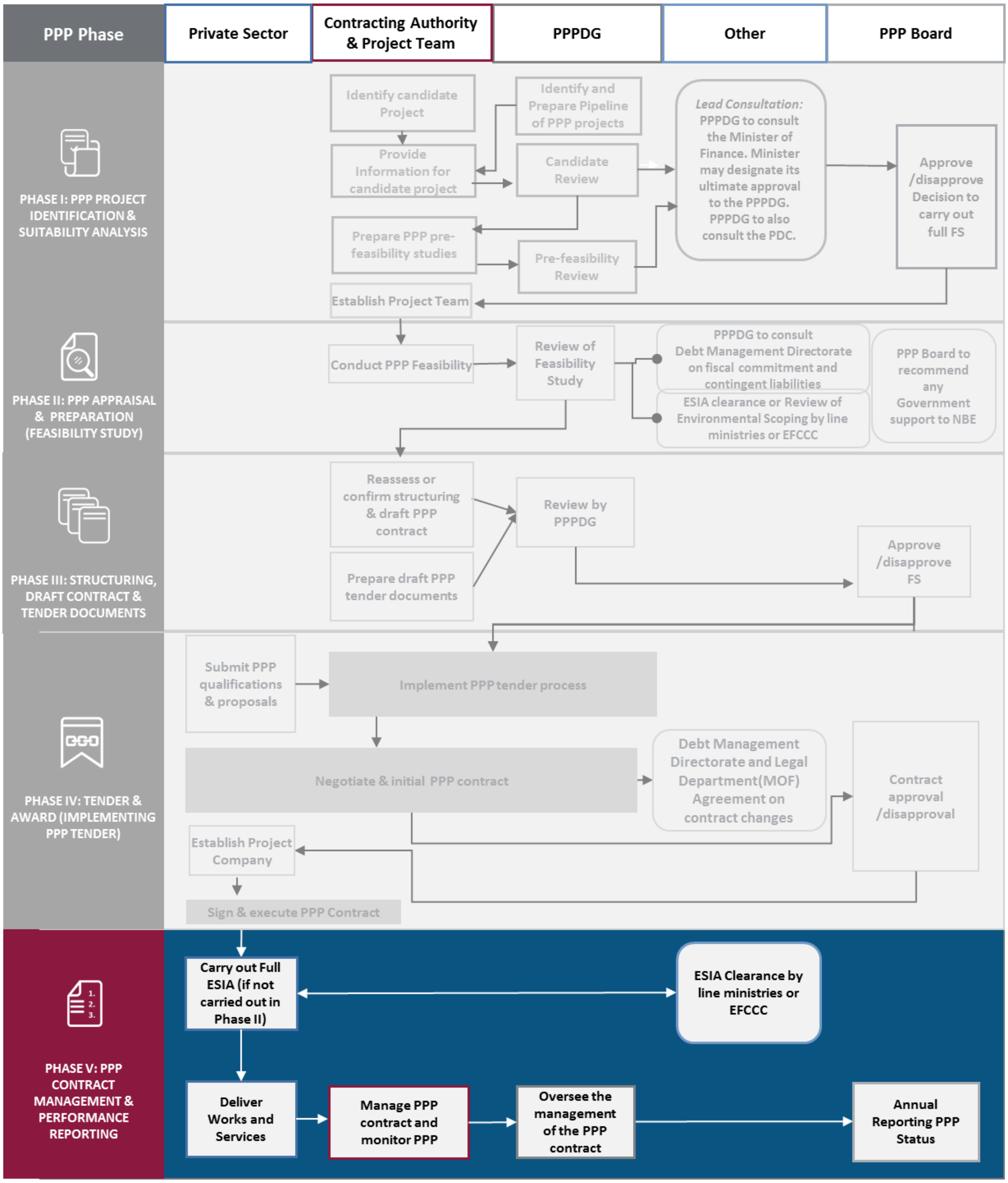

7.1 OVERVIEW OF CONTRACT MANAGEMENT

Even with the adoption of PPP for the provision of Public Service Activity, the public sector still is accountable for that service within the economy. The public sector therefore still has a role to play in terms of oversight over the private partner in a PPP.

The detailed arrangements for this oversight is the Contract Management stage of the PPP cycle. The PPPDG will expend a considerable amount of effort to define a PPP project structure that yields Value for Money for the Contracting Authority and a positive Net Present Value for a private counterpart. The contract management phase ensures that during the delivery of the PPP project (i.e. the construction and operation phases), both the Contracting Authority and the Private Partner retain and manage the respective risks agreed in the PPP Contract, and that any arising unforeseen risks are effectively allocated within the framework of the PPP Agreement so that the Value for Money proposition of the PPP is maintained.

Section 4.1.9 described the broad approaches to oversight over PPPs. The PPP Proclamation reserves for the Council of Ministers the power to issue regulations for the implementation of the PPP Proclamation, but does not create a separate entity to regulate PPPs. The existence of specific sector bodies such as the Ethiopian Energy Authority, the Transport Authority, and the Ethiopian Civil Aviation Authority, all with regulatory oversights over their respective sectors but accountable to their line ministries undermines the possibility of regulation by independent commissions. In Ethiopia, this structure means PPPs will have to be regulated in the individual PPP Contracts, amalgamating regulatory requirements within the specific sector where the PPP service is being delivered and also from the general PPP regulatory framework.

7.1.1 PROJECT MANAGEMENT TEAM

The PPP legal framework assigns the responsibility of PPP contract management to the Project Management Team. The Project Management Team, drawing from its intimate knowledge of the PPP project design, risk allocations and project stakeholders and counterparts, having been involved from the feasibility stage to the tender, is the best party to be charged with this responsibility. As mentioned in section 6.9.1, at the beginning of this phase, the Contracting Authority can adjust the staffing of the Project Management Team with different skill sets for it to fulfill its new role of PPP contract management. As the PPP moves towards the construction and operations stages, it is important to empower members of the Project Management Team familiar with EPC and operational elements of the Public Service Activity.

To achieve this outcome, the Contracting Authority must, in selecting the project team ensure the following:

- Most Contracting Authorities or line ministries responsible for a Public Service Activity will be structured into divisions, with specific divisions responsible for planning, others for engineering, operations and even project monitoring. The members of the Project Management Team who will be best fit for the contract management phase are those drawn from the operations and project monitoring directorate.

Part of the responsibility of the Project Management Team in PPP contract administration will be developing the protocols for institutional communication and managing the relationship between the public sector, the beneficiaries of the PPP service (including the Contracting Authority) and the Private Party. Relationship and institutional channels should be maintained between:

- the private Public Service Activity provider and user groups;
- the private Public Service Activity provider and the Contracting Authority; and
- the private Public Service Activity provider and the Project Management Team.

Managing these relationships provides for the strategic direction and positive evolution of the PPP (continuous innovation and service performance), bridging the plans and expectations of the parties to the PPP Agreement and providing a mechanism for end-user feedback on the Private Party's performance.

In Ethiopia, the PPP legal framework creates the PPP Board, a 9-member body with 2 representatives from the private sector and the remainder from the public sector. The Minister of Finance is chairperson of this PPP Board. The PPP Board has the overall mandate to approve projects to be procured as PPPs, changes to existing PPP risk allocations and review reports of PPP project performance. The PPP Board also has the mandate to approve disclosure of information about PPPs to the public and is the final arbiter of any dispute involving participants to a PPP project. This PPP Board thus has the legal authority to manage communication between all parties to the PPP process.

The PPP Board is legally mandated to meet quarterly. Such meetings shall be the venue to discuss performance of Contracting Authorities and Private Parties to PPP Agreement. The Project Management Team is mandated to prepare a Project Management Checklist, which serves as a scorecard for monitoring the performance of parties to the PPP Agreement. The PPP Board will review with the support of the PPPDG this scorecard and discuss important items brought up for discussion by the Project Management Team.

7.1.2 CONTRACT ADMINISTRATION PROCESS

The Project Management Team's responsibilities are from commercial close (when the PPP Agreement is signed) until handback (when the Private Partner hands back the rights to providing the Public Service Activity). Over the PPP Term, membership of the Project Management Team is expected to change. The only way the Project Management Team will be able to perform its functions effectively over PPP terms sometimes lasting 20 years is by following a consistent process. This process will be designed to effectively make sure that parties to the PPP Agreement effectively manage their assigned risks and that any unforeseen risks that arise are quickly and efficiently allocated so Value for Money in the PPP project is maintained. For such a process to be consistent over a long-term PPP contract, the process must be institutionalized.

The PPPDG and the Contracting Authority will delegate the responsibility of designing the architecture of the contract administration process to the Project Management Team. This contract administration process shall be designed by the Project Management Team (and its advisors) during the project appraisal and structuring phase. This architecture shall describe:

- The make-up of the Project Management Team to ensure successful delivery of the PPP following commercial close.
    - This might require that membership of the Project Management Team setup at the commencement of the feasibility stage would change

    - An organogram of the Project Management Team designed to suit the contract management and administration phase. This organogram should illustrate clear reporting and accountability lines

- A detailed Project Management Checklist, the legally required document, for monitoring PPP parties' performance. The Project Management Checklist must include:
    - A contract management work plan listing all the roles and responsibilities of the parties to the PPP Agreement and other stakeholders. Some tasks will be within the responsibility of the Project Management Team, others will require the intervention or approval of the PPPDG or the PPP Board. The contract management work plan shall clearly spell out the scope of each party and stakeholder and the process to involve such party.
    - A contract management manual with all the procedures for the Project Management Team to perform its role. This will include elements like:
        - How formal communications are sent from the Project Management Team to the Private Partner or the Contracting Authority
        - How requests or complaints from either party to the PPP Agreement are handled
        - Duration, time-limits for handling tasks and requests from stakeholders
    - A PPP project library and information management plan presenting a coherent system for receiving, sorting, documenting, tagging and archiving PPP project correspondences, notices issued and reports. It will be critical for the Project Management Team to track all project information over the entire period, and the Project Library and accompanying information management system will be indispensable to this task.
- Protocols for communications between the parties to the PPP, the PPP Board, the end-user community and other stakeholders

There is significant detail required in the contract administration process and it is essential this task be carried out during the project appraisal and structuring phase to ensure that elements of the contract administration plan which will require legal enforcement are included in the PPP Agreement.

7.1.3 RELATIONSHIP MANAGEMENT

The third philosophy of the PPP contract management process is the need to ensure continuous relationship management between the parties to the agreement. The success of this element will depend to an extent on how rigorous the framework governing the administration of the PPP Agreement (i.e. the activities described in 7.1.2 are designed). The essence of this is ensuring constructive and productive dialogue in all instances between the parties.

The PPPDG shall review the Project Management Checklist to ensure that the tone of the different components of the checklist does not project an "us" versus "them" attitude between the public sector and the private party. The effective management of the PPP relationship is the only means to ensure success and thus the preservation and continued generation of Value for

Money. The PPPDG has a leadership role to play in this aspect, but projecting neutrality and emphasizing the belief that Value for Money for the Government of Ethiopia can only be generated and maintained when all the parties to the PPP Agreement work collaboratively.

To this end, the PPPDG must undertake the following:

- Prepare a stakeholder engagement plan. This will involve identifying all stakeholders to the PPP Agreement and preparing a plan for engaging each stakeholder.
    - During the feasibility study, as project stakeholders are identified the PPPDG must create a record of these stakeholders and their interests in the PPP Project
    - The PPPDG must ensure that the stakeholder engagement complements rather than contradict or duplicate initiatives in the Project Management Checklist
    - Create a communication plan tailored to the requirement of each stakeholder group and ensure that stakeholders are kept abreast of project developments
- Continuously build and develop capacity. PPPs may encounter challenges due to inadequate capacity to deal with arising or unforeseen challenges.
    - The PPPDG must be a thought-leader in the PPP space, continually making available to the Project Management Team, Contracting Authorities and other stakeholders, information on best practices on PPPs.
    - The PPPDG must approve Project Management Team membership ensuring that the team include personnel with the experience and availability to take on the role of monitoring.
- Be vigilant in contract monitoring. The PPPDG must take its role of contract monitoring very seriously and not relent in the belief that the PPP Agreement will be self-enforcing.
    - The PPPDG must request monthly reporting from the Project Management Team
    - Monthly activities should include a review of the Project Management Checklist to ensure the Project Management Team is carrying out all its obligations

7.1.4 PPP PROJECT PERFORMANCE MEASUREMENT AND MONITORING

PPP project performance measurement and monitoring is the procedure of evaluating project outcomes and determining if those outcomes are consistent with specifications in the PPP Agreement. The objective of the measurement and monitoring activity is to ensure that precise project information that is necessary to evaluate performance is available on time and in the quality that allows the Project Management Team make an unambiguous determination about the status of performance. Therefore, project reporting is an intrinsic part of this task.

To avoid disputes about the detail and extent of reporting that will be required as part of the project performance measurement and monitoring, the reporting requirements must be communicated to bidders during the tender phase of the PPP. To the extent possible, same reporting templates or the key performance indicators (and how they are to be computed) must be indicated to the project Request for Proposal.

→ In the project Request for Proposal, the PPPDG must indicate the project outcomes and how those outcomes will be measured

→ The annexes to the Request for Proposal should include template reporting requirements and a list of the Key Performance Indicators (KPI) part of the project performance monitoring

The requirements for this performance measurement activity are different for each of the stages of the PPP life cycle. Often non-compliance with performance measures will result in penalties defined in the PPP agreement. The table below presents some common issues that arise during the different stages of the PPP life cycle and how performance measures can be designed to provide oversight. The list is not intended to be exhaustive but to highlight some of the typical issues and what mechanisms the Project Management Team will have at its disposal to ensure effective performance monitoring.

PPP Life Cycle State	Typical Issues	Performance Measurement Tools
Commercial close	Meeting CPs<br>▪ Receiving operating permits<br>▪ Completing ESIA studies<br>▪ Acquiring land<br>▪ Interconnection facilities<br>▪ Guarantees secured	▪ Project Management Team hold weekly meetings to monitor each party's progress to meeting CPs<br>▪ PPPDG mandated to ensure all public sector permits and approvals secured before PPP goes to tender
Construction	EPC<br>▪ Contractors and suppliers procured and mobilized<br>▪ Obtain construction permits<br>▪ Complete ESIA other studies<br>▪ Changing specifications<br>Commissioning and Testing<br>▪ Assets/equipment perform below specifications	▪ Engage competent Project Owner's Engineer<br>▪ Ensure quality requirements and duty of care imposed on contractors and service providers<br>▪ Supervise inspection and testing of materials and equipment<br>▪ Determine spec changes on performance<br>▪ Ensure adequate testing period, with warranties for defects and other liabilities<br>▪ Allow for independent certifier
Operations	▪ Performance below specifications<br>▪ Request for changes in project output	▪ Monthly and quarterly reporting and tracking of performance<br>▪ Mechanisms in contract to address project variations

Following commercial close, the PPP Agreement becomes the reference document for discussing and addressing any complications (anticipated or not) that arise during implementation. It is therefore critical that the PPP Agreement be as comprehensive in its assessment of the risks to a PPP. This places a high importance on the quality of the feasibility study and the importance of the PPPDG and Project Management Team working with capable and experienced advisors to develop a thorough feasibility.

In general, designing the PPP contract management plan involves developing process, procedures and mechanisms for:

- Gathering the data necessary to determine what level of performance is being provided by the private partner;
- Ensuring that the terms and conditions of the PPP contract are being properly enforced,

- Ensuring that specific requested changes within the PPP contract, such as adjustments in tariffs or prices, are properly analyzed and decided upon; and
- Many other tasks as required on a case-by-case basis in different PPP agreements.

PPP Contract Monitoring Teams typically require additional resources from the Contracting Authority in terms of capable staff, adequate information technology systems, and the budget resources needed to gather and verify technical, operational, financial, and legal performance data. For large complex PPPs, such as BOTs and PFI-type projects, a project monitoring team may require a team of different specialists responsible for monitoring technical, financial, and legal, consumer service or other areas of performance. The size of the staff required for a PPP Contract Monitoring Team depends on the size and the number of different project sites to be monitored.

For smaller PPPs, such as shorter-term service contracts and management contracts, the project monitoring team could consist of just a single individual. A single contract management officer could be supported with the ability to hire qualified, part-time, outside specialists, as needed, to address specific technical, legal or other performance issues.

7.2 PPP CONTRACT MANAGEMENT

7.2.1 PROJECT MANAGEMENT CHECKLIST

One of the responsibilities of the Project Management Team is preparing a Project Management Checklist. This checklist itemizes tasks to be undertaken by the Project Management Team and the PPPDG to monitor the implementation of the responsibilities of the Contracting Authority and the Private Partner under the PPP Project Agreement.

The PPP Proclamation identifies four broad tasks to be carried out by the Project Management Team as part of the PPP Contract Management responsibilities:

i. Submit to the PPPDG annual or such other period reports requested by the PPPDG. Reporting will cover project progress and performance assessment, quality control; financial information and performance; report on the contingent fiscal obligations accruing to the government from the project; stakeholders feedback; lessons learned for the project;

ii. Maintain a record of all documentation and agreements entered into by the Contracting Authority relating to the PPP Project;

iii. Ensure the transfer of assets at the expiry or early termination of the PPP Agreement ;

iv. Other functions as may be assigned to it by the Contracting Authority.

The legal framework[69] requires the Project Management team to submit its reports on its activities to be approved by the PPPDG. One of the objectives of PPPs outlined in the PPP Proclamation is to improve the quality of Public Service Activity. To ensure an objective assessment of this metric, the Project Management Team compile and submit for public

[69] Article 10(6) of the PPP Directive

disclosure the performance report of the private party. This reinforces the proactive disclosure philosophy that is essential for widespread public support of PPPs. The PPPDG shall maintain a public database of current and pipeline PPPs, where private parties' performance, assessed according to objective metrics, can be presented for public review and comment.

A copy of the private party's performance on the Public Service Activity will also be submitted to the Contracting Authority.

The Project Management Checklist shall be prepared by the Project Management Team and the PPPDG [with the support of Transaction Advisors] as part of the Project Close Report for the PPP transaction. The checklist shall derive from a precise scope of work for PPP post-close monitoring and shall detail, in addition to the requirements outlined in the PPP Proclamation, other requirements for the successful monitoring of the PPP Agreement. Generally, elements of the PPP Project that will require monitoring include:

- The Contracting Authority and the Private Partner fulfilling the Condition Precedents in the PPP Agreement. Each party to the PPP Agreement will have explicit objectively verifiable conditions it must meet in order for the PPP Agreement to be effective. These conditions, specified in the PPP Agreement, must be individually indicated in the Project Management Checklist and the Project Management Team must track the progress towards successful completion of all these conditions by both the Private Party, Contracting Authority and other parties to the PPP Agreement. PPP performance during the construction, operation and service delivery stages. Detailed guidance on PPP performance monitoring is provided in section 7.5.1.

> Typical Condition Precedents include:
> - Permitting and planning approvals
> - Land resettlement or acquisition
> - Outstanding technical design issues
> - Signing project finance agreements
> - Funding approvals
> - Registration of security for any loans
>
> The specific Condition Precedents for PPP Projects are identified during the tender phase and are approved as part of the PPP Board approval of the Feasibility Study or the PPP Evaluation Report.

- PPP performance during the construction, operation and service delivery stages. Detailed guidance on PPP performance monitoring is provided in section 7.5.1.
- Managing requests and instances of PPP price adjustment within the context of the existing PPP Agreement.
- Managing requests to revise or renegotiate the PPP Agreement including clarification changes and risk-transfer changes. The Legal Framework directs the Project Management Team to inform the PPPDG if it anticipates a need to renegotiate the PPP Agreement. PPP Agreement changes could be in the form of clarification changes, i.e. changes that provide clarity to previously ambiguous wording, or changes that result in risks-transfer. In its advice to the PPPDG, the Project Management Team is required to indicate the likely consequences of this renegotiation and if the impact would be result in risks-transfer. The Project Management Team should support the PPPDG in preparing an analysis of the impact for the PPP Agreement change for presentation to the PPP Board for consideration and approval.

- Managing PPP contract disputes. Dispute Resolution generally refers to the mechanism for the solving larger differences that may arise between the Private Party and the Contracting Authority in connection with the PPP Agreement. Discussion on dispute resolution is detailed in section 7.5.4.

- End of PPP contract activities. Discussion about end of contract activities is detailed in section 7.5.6.

The reporting done by the Project Management Team will contribute to the knowledge management. Indeed, the collection, review and dissemination of project information would contribute to knowledge transfer to the PPPDG, Contracting Authority and PMT. The PPP contract should specify whether specific knowledge transfer elements are to be included. Staff from the Project Company, the PPPDG, the Contracting Authority and the PMT will be subject to confidentiality agreements. Additionally, only selected non-commercial information will be publicized to the general public.

7.3 PERIODIC INTERNAL REVIEWS

The Project Management Team and the PPPDG will carry out periodic internal reviews (once a year) of the PPP project with the support of the Contracting Authority. The objective of this exercise is to:

- Re-evaluate the project risks and liabilities
- Confirm that the project delivers value for money
- Fiscal Accounting and Reporting for PPP (with the support of the MoF Debt Management Directorate): The PMT and the PPPDG with the support of the MoF Debt Management Directorate will monitor any fiscal commitments from the Government. The PMT and the PPPDG will support the MOF Debt Management Directorate collect and record existing guarantees on debt and other types of contingent liabilities from PPP projects. The MoF Debt Management Directorate will maintain a schedule of Fiscal Commitments for all PPP Projects.

The PPPDG will present the findings of the review to the PPP Board, which will decide if certain information contained in the review shall be provided to the public.

Management of information throughout the PPP lifecycle

The proper management of information allows easy access to the project information, helping the PPPDG meet its obligations in regards to storing, disclosing and protecting information.

The PPPDG may set up and maintain a "PPP in Ethiopia" website which will be a link to individual projects as well as a place to access framework documentation (including the guidelines) and other relevant information on the pipeline, tenders, news and developments. The website could be used to access virtual data rooms or alternatively provide the link to the individual project data room. The site could also be used to upload, share, archive files regarding PPP development. At the tendering phase, virtual data rooms can be hosted on this website to share with bidders the tender documentation, and other supporting materials. At the contract management phase, the website will facilitate (i) monitoring of the private party, (ii) record keeping, (iii) managing documentation version controls, (iv) reporting, as relevant, to the public.

7.4 DISCLOSURE OF INFORMATION AT CONTRACT MANAGEMENT PHASE

The perception of fairness and a transparency in the process is important to building private sector confidence in the PPP regime in any country. In countries like Ethiopia without a history of many PPPs, these perception issues are very critical. In the World Bank Group "A Guide to Disclosure in Public-Private Partnerships Projects", the authors states, "…the private sector will only be interested (on a significant scale rather than opportunistically) in PPP programs if the procurement rules provide and protect transparency and fairness in selection, as well as access to meaningful information and studies on the projects to enable potential bidders to assess the opportunity in an efficient manner."

The World Bank Group advocates a proactive disclosure policy, where policy makers proactively circulate information about PPPs to the investor community as well as general stakeholders. This would be in different from a reactive disclosure approach, where information is provided either in response to a request from participants in a PPP process, in accordance with public procurement laws or following from a Freedom of Information legislation type request.

In Ethiopia, the PPP legal framework provides the foundation for a proactive disclosure policy. The PPP Proclamation gives the PPP Board the Authority to compel the PPPDG to provide information about the implementation of PPP projects to the general public (Article 12).

The PPPDG shall develop a policy for disclosure of information about PPP in line with mandate under the legal framework. This policy, when developed, shall be presented to the PPP Board for approval, consistent with the requirements of the PPP Proclamation. The policy will seek to set parameters for when disclosure on PPP project should commence, what sort of information should be disclosed, what media to use for the disclosure and how to safeguard commercially sensitive and other intellectual property of private parties to PPP projects or opportunities.

Once the PPP reaches commercial close and parties are in the process of fulfilling condition precedents to reach financial close, the PPPDG can share more information about the PPP. At this stage, any information disclosure is essential for ensuring that both the public Contracting Authority and the private party are maintaining and managing their assigned risks under the PPP Agreement.

The PPPDG, with the approval of the PPP Board, shall publish the PPP Agreements, redacting commercially sensitive information such as cost of capital for private firms, names and contact details of any personnel named in the contract. Very generally, the information in the general conditions of the contract can be disclosed with minimal reductions. These clauses in that part of the agreement speak to general terms that will be common across many PPPs and agreements in Ethiopia. Under the Special Clauses, company intellectual property, such as costs basis as well as third party or supplier information referenced in the agreement shall be redacted. The PPPDG will also, after approval by the PPP Board, at certain intervals, provide information to the public on the implementation of PPP projects.

7.5 GUIDANCE ON MANAGING KEY ISSUES

7.5.1 REPORTING AND PERFORMANCE MONITORING

PPP contract monitoring requires whole-life performance supervision and continuous monitoring, as opposed to simply confirming the construction of new infrastructure assets and facilities. This activity ensures that the Private Party complies with contract provisions throughout the life of the PPP Agreement, ensuring that physical assets meet proposed specifications and service levels are as committed in the PPP Agreement. Performance monitoring is carried out by the Project Management Team. The Project Agreements may also specify security to be provided by the Private Party to guarantee the performance of any of its obligations under the Project Agreements (article 51 of the PPP Proclamation).

7.5.1.1 Key Issues to monitor in PPP Agreements

The table below presents the sections of three PPP Agreement templates provided with this guidelines document. The next section discusses some of the key clause in these sections.

7.5.1.1.1 Concession Agreement for Brownfield Asset

Revenue and Currency Risk. The Private Party assumes the revenue risk under this PPP structure. Article V – Facility Fees assigns the risk for collecting user fees for the use of the service. The clause identifies all the elements of the revenue risks (billing, collection, receiving and depositing) assigned to the Private Party. The clause also states in which currency denomination project revenues are to be collected.

Payments to Contracting Authority. Article VI specifies payments to be made to the Contracting Authority under the PPP. In the example of the brownfield concession, the Private Party is taking over an operational activity (i.e. a going concern) and thus the concession is structured with concession fees paid from the concessionaire to the Contract Authority from the first anniversary of the PPP and over the term of the lease.

Performance Security. The Private Party is also obliged to present a performance bond as security for its performance under the concession agreement. Article XI describes a performance security that can be called by the Contracting Authority for among others, the following:

- Contracting Authority's losses as a result of concessionaire's breach of obligations under the agreement
- Compensating the Contracting Authority to remedy physical defects
- Compensating the Contracting Authority in case it exercises its step-in-rights
- Satisfying penalties to be paid by Private Party in accordance with the agreement

No major capital expenditures are anticipated under the brownfield concession agreement and thus there are no specific clauses dealing with the assignment of construction risk.

Concession Agreement for Brownfield Asset	Concession Agreement for Greenfield Asset	Management Contract Agreement
1. Article I – Definitions And Interpretation	Article 1 Definitions And Interpretation	1. Definitions And Interpretation
2. Article II – Concession	Article 2 The Concession	2. Nature Of Agreement
3. Article III –Term	Article 3 Conditions Precedent	3 Services
4. Article IV – Concession Fees	Article 4 Annuity Payment	4 Contract Monitoring
5. Article V – Facility Fees	Article 5 Warranties	5 Personnel
6. Article VI – Conditions Precedent	Article 6 Grantor's Administration Of The Project	6 The Operator's General Obligations
7. Article VII – Other Obligations Of The Concessionaire	Article 7 Coordinating Committee	7 Contracting Authority Responsibilities
8. Article VIII – Obligations Of The Grantor	Article 8 Land Use Right	8 Compensation
9. Article IX – Representations And Warranties	Article 9 Design	9 Reporting
10. Article X – Liability And Indemnification	Article 10 Construction Works	10 Limitations On Authority
11. Article XI – Performance Security	Article 11 Testing And Completion	11 Force Majeure
12. Article XII – Insurance	Article 12 Delay In Completion And Abandonment	12 Termination
13. Article XIII – Force Majeure	Article 13 Operation Of The Project Highway	13 Insurance
14. Article XIV – Political Event	Article 14 Maintenance Of The Project Highway	14 Change In Law
15. Article XV – Termination Of Agreement	Article 15 Project Highway Toll Fees	15 Indemnification And Liabilities
16. Article XVI – Handback	Article 16 Labour	16 Limitation Of Liability
17. Article XVII – Assignment Of Agreement	Article 17 Financing Of The Project And Financial Management	17 Confidentiality
18. Article XIII – Substitution Of Concessionaire	Article 18 Transfer Of The Project	18 Title, Documents And Data
19. Article XIX – Grantor's Step-In Right	Article 19 Insurance	19 Governing Law And Dispute Resolution
20. Article XX – Governing Law And Dispute Resolution	Article 20 Other Obligations Of The Grantor	20 Miscellaneous Provisions
21. Article XXII – Miscellaneous Provisions and Change in Law	Article 21 Other Obligations Of The Concessionaire	
	Article 22 Obligations And Rights Common To The Grantor And The Concessionaire	
	Article 23 Force Majeure	
	Article 24 Termination	
	Article 25 Compensation For Breach Of This Agreement	
	Article 26 Liability And Indemnification	
	Article 27 Assignment Of The Agreement And Approval By The Grantor Of Contracts	
	Article 28 Miscellaneous Provisions and Change in Law	
	Article 29 Governing Law And Dispute Resolution	

7.5.1.1.2 Concession Agreement for Greenfield Asset

Land Use Right. Under the Greenfield Agreement, significant capital works are required and the Contracting Authority and Private Party are both responsible for elements of this development. The Contracting Authority is required to make the land available for the development. Article 8 defining land use, identifies the key risks involved in securing land and allocates the risks and responsibilities among the parties. Article 8 assigns to the Contracting Authority:

- The obligation to make land available for the private party to acquire
- Providing and maintaining main roads, fit for purpose, to the site

The agreement places limitations of the use of the site by the Private Party. For instance, the private party shall only use the site for the purpose defined in the agreement.

Oversight over Project Design Specifications. Article 9 elaborates on the role and responsibilities of each of the parties to the agreement of the preparation of the project specifications. The Private Party is assigned the responsibility to design, but requires the approval of the Contracting Authority. However, to ensure that private sector efficiency is not stifled by public sector bureaucracy, the Private Party is given the authority to propose changes to the design is certain conditions are met. In all instances, the Contracting Authority would have to review and approve changes.

Project Construction Works. The risks for project construction works are assigned to the Private Party. Article 10 defines the roles and responsibilities of both parties. The Private Party is required to carry out the construction works in accordance with the design requirements and complete construction on or before applicable milestone dates. The Contracting Authority is given oversights powers to carry out quality assurance on the construction works being carried out by the Private Party. If the Contracting Authority determines that the quality of work is not in accordance with the project requirements, it has the authority to:

- Compel the Private Party to carry out the remedial works
- Engage a third party to carry out the remedial works at the Private Party's cost

Payment Obligations. Two potential payment clauses are included in the template agreement:

1. An annuity payment clause prescribing annual payments to the Private Party for meeting the requirements of the Agreement. The Annuities are payable in monthly installments and are only payable subject to the Private Party performing its obligations to a specified standard. Payment can also be quarterly as per the PPP Agreement.

2. An annual concession fee in the scenario where the Greenfield PPP structure will involve concession fees from the Private Party to the Contracting Authority (e.g. inland port terminal PPPs or other freight traffic PPP).

Revenue Risk. Part of the Private Party's obligation under the agreement is the collection of user fees. Under this template agreement, the revenue risk for the user fees is on the Contracting Authority.

7.5.1.1.3 Management Contract Agreement

The Management Contract Agreement is designed to provide short term managerial and technical expertise to the Contracting Authority. Typically, the Contracting Authority assumes most of the revenue risk associated with such PPPs.

Under the template agreement, the Private Party assumes the revenue risk for the project. Article 8 defines the Private Party's compensation and it is fixed over the duration of the PPP Term. Thus the Private Party assumes risks associated with inflation and cost increases. The agreement, however, makes provisions for a renegotiated increase in fees, if the Private Party is able to objectively demonstrate losses over successive reporting periods.

The agreement also provides for a revenue-sharing mechanism whereby if the revenues are above a target threshold, the Private Party shares the gain with the Contracting Authority.

The Private Party under the template agreement is subject to extensive reporting to prove that its performance is in line with Key Performance Indicators specified in the agreement. There are no financial penalties associated with non-attainment of the Key Performance Indicators, however, non-performance provides grounds for the Contracting Authority to terminate the Management Contract.

BEST PRACTICE
1. **Selecting performance indicators, during the structuring of the project,** that are objective, quantitative, & verifiable: When the Contracting Authority and the Project Management Team decide which indicators to monitor, it is important to select ones that can be readily verified and are not subject to interpretation.<br><br>2. **Select the most important performance data to monitor and analyze** rather than the most numerous. In practice many PPP regulators, especially for brand-new pilot PPP projects, are tempted to request as much information as possible from contractors about their performance.<br><br>There is often an institutional concern that the new private service provider is secretly making profits from the public's money, and that the monitor's job is to inspect all input decisions, activities, and expenditures to discover how these hidden private returns are being achieved. Often such PPP monitors soon find themselves overwhelmed by the volume of information and data they must process and analyze. Often after about 2 annual business cycles or years, they have come to realize that it is better to limit performance data only to the most important ones that cover the biggest areas of the contractor's performance.

7.5.2 ANTICIPATED CHANGES AND TRANSITIONS

Changes addressed in the PPP agreement will be handled by way of the terms and conditions of the PPP agreement.

It is nearly impossible to write a single PPP Agreement that can foresee with certainty all changes to the delivery of a service, particularly in the long term. All parties to a PPP Agreement, essentially agree at the outset that there may be changes in the conditions within which a PPP project operates.

Focus on price adjustments

Therefore, some predictable procedures shall be agreed upon in advance in the PPP Agreement for how instances of any possible adjustments to PPP prices and tariffs should be requested, reviewed, and decided.

The key decision about which price adjustment technique a PPP project shall adopt depends upon what types of performance the Government would like to incentivize from the private partner; such as more efficient operations or increased investments. There are two main options of setting and adjusting prices for long-term PPP projects. Each technique provides different incentives for the private partner to either make new investments in the project or to improve the operating efficiency of project. Neither technique is necessarily superior to the other. Rather each should be applied to meet the unique needs and priorities of the project or of the given infrastructure sector and the specific PPP project. These techniques include:

- Rate of Return Regulation
- Price-Cap Regulation

Rate of Return Regulation. In rate of return regulation, the Project Management Team must first review all of the assets that the PPP project company has invested in and determine if they are used and useful. The Project Management Team then reviews the operating expenses of the PPP project and determines if they are all prudent and necessary. Monitoring shall focused on what the Private Party's return on capital should be. Prices are then adjusted to meet the revenue requirement that provides this approved rate of return.

Price-Cap Regulation. The second major technique of managing price adjustment issues for PPPs is price-cap regulation. Under price-cap regulation, the Project Management Team will fix the maximum price(s) that the Private Party may charge during a given period, say 5 years. The Project Management Team allows the Private Party to earn and keep whatever returns they achieve from their efficiency improvement for that 5-year period. This approach has been a more effective way of incentivizing private service providers to improve efficiency and reduce any excess operating expenses. However, this technique has not necessarily encouraged Private Parties to make new investments in areas like system reliability and security. While a price-capped private partner would choose to invest in a new asset that reduced operating costs it would be reluctant to invest in new assets that do not directly reduce operating costs. Under price-cap technique, monitors often need to be more vigilant in their performance monitoring to make sure that private service providers are not being over-aggressive in reducing their operating costs at the expense of the technical quality and reliability of their service.

7.5.3 UNANTICIPATED CHANGES AND RENEGOTIATION

The PPP Proclamation[70] and Directive[71] detail the process to follow for renegotiation which can be structured into three phases:

- Phase I: In the event that the Contracting Authority considers renegotiating a PPP agreement, the Project Management Team will inform the PPPDG of the expected need to renegotiate and likely consequences.

[70] Article 9(1)(c)
[71] Articles 14(5) and 10(7)

- Phase II: Based on the PMT's initial assessment the PPPDG will prepare an appropriate analysis of the impact of proposed material changes to the project, with the involvement of the Contracting Authority and Project Management Team.
- Phase III: The PPPDG submits its analysis to the PPP Board for decision.

Renegotiation involves a change in the PPP agreement itself, as opposed to changes addressed by adjustment mechanism provided for within the PPP agreement itself. Renegotiation leads to changes in.

- Output specifications;
- Refinancing (this concept is developed in the box below);
- Project scope;
- Duration;
- Tariff levels beyond the ones stipulated in the contract.

KEY CONCEPTS AND DEFINITIONS

Refinancing

"Refinancing refers to changing or replacing the existing lenders or terms on which debt obligations have been agreed between the Project Company and its lenders. The Project Company will have typically raised debt capital for the project and where it has taken the risk on the debt financing it is generally entitled to rearrange it (though often subject to restrictions). The financial structure of the Project Company is also of interest to the Procuring Authority as it can affect the financial integrity of the Project Company and the project. For example, a refinancing has the potential to raise additional debt which can overleverage the project and/or increase the government's contingent liabilities." Source Global Infrastructure Hub

"Generally, refinancing is not considered a downside risk but an upside benefit. When the project performs well, the private partner will have the ability to renegotiate the debt on better terms or refinance the debt, including an increase in the debt level so as to amortize share capital early ("recap"). Even if the project is performing simply as anticipated, there is likely to be an option to refinance since the risk profile will improve by entering into the Operations Phase.

Although not common worldwide, a number of countries, especially developed countries, introduce provisions in their contracts so that the authority has access to a portion of the refinancing gains. When a government wants to reserve for itself access to the refinancing gains (usually by a decrease in the service payments, although sometimes by means of an upfront payment), it is not common practice to request more than 50 percent of the upside.

Refinancing may also be an adverse risk when the financing strategy of the private partner relies entirely on refinancing: instead of financing from the outset on a long-term basis, the project company enters into a short-term debt (typically under a "mini-perm structure") with the intention of substituting that financing with a long-term financial agreement (maybe in the form of project bonds when the development of the country-market allows for this). In such situations, there is a

risk of refinancing being under worse (or much worse) conditions than anticipated in the financial plan." Source: APMG PPP Guide

Governments tend to take share of upside benefit of refinancing and not of downside risk, which is a private sectors risk. The PPP Agreement includes clauses regarding refinancing and upside sharing.

Due to their long-term nature and complexity, PPP agreements contain provisions that allow changes and adjustments to be made within the framework of the contract. However, renegotiation, which are not provided for with the PPP framework, can be initiated by both the Government and the private operator.

Table 7-1: Main Reasons for Renegotiation

Government Initiated Renegotiation	Private Party Initiated Renegotiation
Opportunistic reasons (example: wanting tariff decrease prior to election)	Mainly rent- seeking behaviour
Change of priority/ new policy objective	Exogenous shocks (domestic and international) affecting financial health of the project company
Incompleteness of the contract	Incompleteness of the contract

In some instances, renegotiations could be beneficial where new opportunities arise over the contract period (for example availability of new technology, unforeseen increase in demand) that justify changes to the PPP contract. However, renegotiations often result in increased cost for the Contracting Authority and are more advantageous to the private party (i.e. increased tariffs, reduced investments, or lowered service requirements)[72].

Since renegotiations have considerable impact on both parties, they require careful examination and analysis by the Contracting Authority and the PPPDG. The Contracting Authority shall monitor the financial health of the private party in order to identify risks and potential issues that could lead to renegotiation.

If the Contracting Authority and/or the private party wish to engage into negotiation, the Contracting Authority will first inform the PMT of such request. The PMT will then prepare an initial analysis that will cover:

i. The reason for renegotiation (causation, significance)

ii. Expected outcome on risk allocation

iii. Potential changes to the project scope

iv. Potential impact on value for money

This assessment is then submitted to the PPPDG.

The PPPDG based on the PMT's initial review will decide whether to carry out an in-depth analysis of the outcomes of the renegotiation or to reject the demand for renegotiation. The

[72] PPP Reference Guide, Version 3 (2017)

Contracting Authority's and/or the private party's demand can be rejected if the reason for negotiation:

- was foreseeable at financial close
- is due to the materialisation of a risk allocated to the parties
- is to correct excessively risky/aggressive bids by the private party
- is the poor performance of the project company

In the event that the PPPDG decides to carry out a detailed assessment of the appropriateness of renegotiation, this study will include legal, financial, commercial and technical aspects and will include the following:

Table 7-2: Assessment of the Need for renegotiation

i.	Causation
ii.	Fault
iii.	Materiality
iv.	Financial performance of project company
v.	Non-financial performance of project company
vi.	Financial cost assessment
vii.	Adverse consequence assessment
viii.	Risk assessment
ix.	Potential impact of renegotiation on all stakeholders including lenders
x.	Impact on value for money
xi.	Opportunity costs
xii.	Costs of alternative scenario (termination, step-in rights)
xiii.	Recommendation

The PPPDG will also seek clearance with the MOF Debt Management Directorate and the National Bank of Ethiopia to determine the fiscal and budgetary implication of renegotiation and its impact in terms of foreign currency requirement. The PPPDG submits the assessment to the PPP Board which decides on the appropriateness of renegotiation. In formulating its decision to proceed to renegotiation, the PPP Board shall consider:

- the long-term risk, costs and potential benefits
- that there is evidence demonstrating that the project is likely to default under the current agreement and the distress is material.
- that there is evidence demonstrating that the distress will be adverse consequence on the end user and/or the public sector.

The PPP Board can decide to reject the renegotiation request. In this case the PPP Board can instruct that the current PPP contract be implemented or suggest alternative option like termination of contract.

If the PPP Board approves the renegotiation, the PPPDG with the support of the Contracting Authority and the PMT will enter into renegotiation with the private party. During renegotiation,

the PPPDG will insure that (i) the value for money of the project is preserved, (ii) the original bid is respected, (iii) the project is affordable and (iv) the risk allocation matrix is unchanged.

Before the renegotiation agreement is signed by the Contracting Authority and the private party:

- → the PPPDG submits it to the PPP Board and explain the changes to the original contract.
- → The PPPDG will present the long term costs, potential benefits and risks
- → The PPP Board will grant approval to sign amendment or request for further renegotiation.

BEST PRACTICE
<u>**Need for Adequate Resourcing during Renegotiation**</u><br>Renegotiation requires as much preparation as the initial negotiation during the procurement phase. The PPPDG must ensure it has the adequate resources to carry out (i) the analysis to determine the need for renegotiation and (ii), if approval by the PPP Board, the renegotiation.<br>The Project Company will employ experts for the renegotiation. The public party must also have in its team legal, technical and financial experts. If the PPPDG, does not possess these resources internally, it should hire, using the traditional procurement process, external advisors with the appropriate competencies and expertise.<br><br><u>**A Process that should not be Systematic**</u><br>Some of the reasons why renegotiations should not be systematic include the following:<br>▪ Absence of competition mechanisms in play since the Contracting Authority negotiates directly with the private party<br>▪ Potential moral hazard from the private party who will strategically bid during the tendering process and renegotiates the PPP agreement during implementation<br>▪ Potential reduction of the economic benefit of the PPP<br>▪ In the case of renegotiations initiated by the Contracting Authority, potential loss of confidence amongst the private sector regarding the PPP program and the Government's commitment with the assumption that bidders will not get their expected benefits for projects as the Government will renegotiate contract during implementation.<br><br>The renegotiation process should be made transparent in order to avoid loss of confidence in the PPP process.

7.5.4 DISPUTES AND RELATIONSHIP MANAGEMENT

Three types of disputes can occur during the implementation of a PPP project:

- Disputes the Contracting Authority and the private party: such disputes are settled through the dispute agreement mechanism provided in the project agreement which

could include arbitration and other types of alternative dispute resolution mechanisms[73].

- Disputes between the private party and the end-user: the Contracting Authority may require the private party to establish a mechanism to handle claims submitted by end-users[74].

- Disputes between (i) the private party and its shareholders, (ii) the private party and its business partners (suppliers, contractors and other partners): the private party shall be free to agree on the appropriate mechanism for settling disputes[75].

The PPP Agreement establishes the protocols for addressing conflicts between the Contracting Authority and the Private Party.

Dispute resolution provides the general mechanisms and procedures by which the parties to the PPP Agreement will communicate, discuss, and agree on reasonable contract modifications.

Modern PPP projects focus on five dispute resolution mechanisms, between the Contracting Authority and the Private Party, described below:

> The legal framework in Ethiopia does not provide for an oversight body comprising representatives of the Private Party and Contracting Authority. If such a body were to be established its authority would be founded in the PPP Agreement.

1. **PPP Project Negotiation Committee**. PPP Agreements could formally establish a working group of middle and senior-level officials from both sides that regularly meets to discuss overall project progress, especially new problems that have arisen and possible solutions. This is called the PPP Project Oversight Committee. The Oversight Committees shall represent the senior managers of both the Contracting Authority and the Private Party, who should be given a chance to review these issues within the context of the overall partnership relationship of the project. These senior representatives on the committee shall then decide it if is really worthwhile to declare an official dispute and to take its resolution to the next level of seeking mediation.

2. **Dispute Resolution Board.** A Dispute Resolution Board is a form of alternate dispute resolution that allows for "real time" prevention or resolution of disputes. It typically consists of a panel of impartial technical and legal professionals, independent of the contracting parties, put in place at the beginning of the implementation of the project. Both the Contracting Authority and the Private Party select a board member and a third member who will chair the panel is jointly selected by the parties. The objective of the DRB is to resolve technical and legal issues as they arise during the course of the Project. If such a body were to be established its authority would be founded in the PPP Agreement.

3. **Mediation of PPP Disputes**. Mediation requires both sides to agree to appoint a qualified official, independent of either of the two parties, who is knowledgeable about PPPs in general and about the given infrastructure sector in particular. In practice such mediators often have legal backgrounds as lawyers or even former judges. It is common that both sides

---

[73] Article 61 of the PPP Proclamation
[74] Article 62 of the PPP Proclamation
[75] Article 63 of the PPP Proclamation

will request that the mediator be independent of all national and political pressures, and therefore not be a national of the host country Government, nor of the home country of lead private service provider or its investors. The mediator hears both sides' testimonies and then issues a recommended solution. The key to the success of mediation of a PPP is that it should be able to done quickly and without significant expense for either party. However, the defining characteristic of mediation is that neither side is legally bound to accept the mediator's finding. Mediations are successful because both parties accept the recommendation of the mediator as being close to what any binding arbitration or litigated outcome would decide. Mediation thus saves both parties months or years and thousands of dollars in legal fees to arrive at pretty much the same binding decision. A mediation should be a precondition to Arbitration or Litigation.

4. **Binding Arbitration**. Under binding arbitration procedures, both sides agree to appoint a single arbitrator or a panel of arbitrators to hear their case. Such arbitrators and their organizations shall be agreed to and stipulated in the PPP Agreement. Unlike mediation, where both parties agree that they are free to ignore the recommended solution of the mediator, arbitration is usually classified as "binding" – meaning that both parties commit in advance that they will accept and abide by the decision of the arbitrator. In this sense, arbitration resembles an official court of law. However, for PPPs, arbitration has the advantage that it can still be significantly quicker and cheaper than going through official litigation procedures required by courts. Arbitration panels can be set up in a matter of a few weeks or months. Additionally, sector specialists who understand the technical, operational, and even contractual issues could be selected as arbitrators. If arbitration for a PPP dispute is not binding, then often the only opportunity for a party to appeal the decision is to seek resolution through official litigation. Arbitration should be preferred to Litigation since most international entities simply would not agree to be bound by litigation in Ethiopia. Arbitration should proceed in accordance with internationally accepted rules such as London Court of International Arbitration, International Chamber of Commerce to be specified in the Project Agreement. The Disputes Provision will specify the binding nature and enforceability of the arbitration award.

5. **Litigation**. PPP disputes may also be resolved through formal litigation in courts of law. This tends to be the costliest and time consuming dispute resolution approach, even for the "winner". For PPPs that involve foreign investors and lenders, the dispute resolution mechanisms of the PPP Agreement almost always require that a third country's law must be relied upon in the litigation of any disputes.

7.5.5 CONTRACT TERMINATION

The Contracting Authority may terminate the PPP agreement with the private party. Events that shall trigger the termination of the contract (including material breach, force majeure event, and payment default) are defined in the PPP Agreement[76].

[76] Article 28 of the PPP Directive

Similarly, the PPP Agreement will include the process for how compensation due to the different parties is calculated[77]. It may also provide the procedure for the transfer of asset to the Contracting Authority or another Public Entity[78].

The Contracting Authority will be responsible for ensuring that the assets transferred in good condition subject to normal wear and tear, while the PMT is responsible for ensuring that the transfer of assets at termination is consistent with the terms and conditions of the PPP Agreement[79].

In the case of early termination, the Contracting Authority may agree with the financiers of the project to replace the private party with a new entity that will continue to execute the existing Project Agreement[80].

All PPP Agreements shall specify in detail the procedures for PPP agreement termination. PPP agreement termination must be formally sought or requested by one of the parties. PPP Agreement termination shall have very detailed procedures for how all the accumulated costs of the project, including past investments, outstanding project-backed loans, and current inventories of assets will be allocated and recovered. Generally, in larger PPPs with project-backed financings, lenders require that if the Contracting Authority seeks termination for any reason other than non-performance, then the full amount of their outstanding loans plus additional fees (covering their “loan pre-payment risks”) be paid by the Contracting Authority. Additionally, project-backed lenders generally insist on asserting their own “step-in rights” for PPPs. This means that if the Private Party fails to perform, then the lenders may first “step in” and take-over the project and appoint their own management team and operating contractor to successfully operate the project and thereby pay-off all outstanding project loans.

PPP Contract terminations are expensive for all sides involved, and that is why they are relatively rare in large, capital-intensive PPPs. Almost all sides find it is more cost-effective to try to restructure and renegotiate these deals and to share the costs of revising these contracts when compared to the cancellation of PPP contracts.

The key preventing expensive cancellations of PPP contracts usually lies in the thoroughness and transparency of the many months (or years) of time spent by Governments and by private bidders in conducting detailed project analyses and due diligence. PPP contracts that are not competitively tendered and that have not had detailed due diligence analyses completed – have a higher probability of either needing to be renegotiated or of being terminated, when compared to openly tendered PPPs. The reason for this is that open competitive tendering requires both Governments and private bidders to complete better and more detailed analyses of a PPP project’s feasibility, the bankability of its risk-allocation structure, and the ability of the contract to manage the resolution of reasonable disputes. These due diligence and tendering procedures provide very important long-term benefits to the PPP: they ensure that all possible, material risks have been identified, analyzed, and properly allocated to avoid PPP contract termination.

---

[77] Article 59 of the PPP Proclamation

[78] Article 27 of the PPP Directive

[79] Article 14(2)(i) of the PPP Proclamation and article 27 of the PPP Directive

[80] Article 57 of the PPP Proclamation

7.5.6 END OF PPP CONTRACT

End of contract activities include inspections, residual assets valuation which consists of the remaining book value of the asset at the end of PPP Term, asset transfer techniques, contract close-out procedures, termination of performance/surety bonds, PPP re-tendering options among others. It is important to ensure that the process that follows the end of the PPP Term is clear to all parties at the very beginning. This procedure helps ensures that the project's minimum performance levels continue to be delivered all the way through the end of the PPP Term. The Project Management Team will ensure that the transfer of assets at the expiry or early termination is consistent with the terms and conditions of the PPP Agreement. This agreement will provide the manner in which the Contracting Authority will ensure that the assets are transferred in good condition subject to normal wear and tear. Many long term PPPs (where residual assets are transferred to the Contracting Authority at the end of the PPP Term) may provide the Private Party with the incentive to reduce maintenance and asset rehabilitation expenditures during the final years of the PPP Agreement, in the hopes of increasing profits during those last few years. This would mean that when the project's assets are transferred over to the Contracting Authority at the end of the PPP Term, assets are not only fully depreciated but possibly non-functional. Having to completely renovate such old assets will require great expense by the Contracting Authority as well as likely disruptions in service for end users. While Contracting Authorities should generally expect that at the end of a PPP Term, project assets will need new investments, these assets should still be functional. Contracting Authorities, therefore, shall be prepared with a plan for the rehabilitation of the project's assets at the end of the contract term, whether they opt to take-over the project and deliver the services themselves publicly, or seek to contract a new private partner to do it, or if they wish to extend the contract with the existing Private Party.

> Four options available to the Project Management Team at the end of the PPP Term include:
> 1. Contracting Authority takes over the project residual assets and continues delivery of services
> 2. Contracting Authority carries out competitive process for Private Party to take over/renew residual assets and delivery services
> 3. Contracting Authority negotiates with the existing private service provider to extend termination date of the PPP Contract and renew assets
> 4. If the PPP is structured such that the Private Party owns residual assets, then the Contracting Authority may wait for PPP Agreement to expire and then let Private Party dispose of residual assets.
