# Supplementary material for: Range of hurdles and opportunities for developing PPPs in diagnostics: a contextual analysis, Ethiopia
Source: BMC Health Serv Res. 2026 Mar 28;26:651. doi: 10.1186/s12913-026-14354-z (PMC13151300; doi:10.1186/s12913-026-14354-z)
Supplement: Supplementary file 4 — Supplementary Material 4 [file 12913_2026_14354_MOESM4_ESM.pdf]

A DIRECTIVE ISSUED TO IMPLEMENT PUBLIC PRIVATE PARTNERSHIP

CHAPTER ONE

GENERAL

1. This Directive is issued by the Ministry of Finance and Economic Cooperation pursuant to Article 65/2 of the Public Private Partnership Proclamation number 1076/2018.

2. Short Title

This directive may be cited as a "directive issued to implement Public Private Partnership directive number 55/2010/2018".

3. **Definitions: -**

Unless the context changes otherwise, in this directive, words defined under PPP Proclamation shall have the meaning given to them by the proclamation. In addition to this: -

1/ "Proclamation" means the Public Private Partnership Proclamation no 1076/2018;

2/ "Government Support" means the economic support and guarantees that may be provided in accordance with Article 47 of the Proclamation;

3/ "PPP Directorate General Representative" means the representative of the PPP Directorate General designated by the PPP Directorate General to serve on the Project Management Team;

4/ "PPP Project Pipeline" means a list of projects that have been identified as potential Public Private Partnership projects;

5/ "Project Management Team" means the team established by the Contracting Authority in accordance with Article 14 of PPP proclamation and Article 9 sub article 1 of this directive.

6/ "Project Management Checklist" means a checklist designed to assist the Project Management Team and the PPP Directorate General in monitoring the responsibilities of the Contracting Authority and Private Partner under the Project Agreements for a specific project.

4. Scope of Application

This Directive shall apply to all Public Private Partnerships to which the Proclamation is applicable.

CHAPTER TWO

ADMINISTRATION OF PUBLIC PRIVATE PARTNERSHIPS

5. Members of the Public Private Partnership Board

1/ The board shall have nine members total. Seven of them are assigned from government sectors while the two members are represented from the private sector.

2/ The board chair shall appoint two persons serving in institutions representing the private sector that will serve on the Board and ensure that these positions are occupied.

a. The board chair shall request from the two private sector persons considered to be appointed as Board members to declare their professional activities, including consultancy services, management positions at public or private companies, retention of shares or other interests in companies, and volunteer activities.

b. The two private sector persons will be appointed to serve on the Board for a renewable term of three years.

3/ Any Board member may be dismissed by the board chair at anytime without notice if it is proved that they are in breach of the ethics obligations provided in Article 29. When overnment sectors representing board, members are found in breach of similar ethics oblgations, they shall be made accountable as per the law that governs public sector.

4/ A member of the PPP Directorate General shall act as secretariat to the Board. The PPP Directorate General may attend and participate, as a non-voting participant, in all meetings of the Board.

6. **Meetings of the Public Private Partnership Board**

1/ all decisions of the Board shall be adopted at meetings of the Board in the form of a written approval.

2/ The Board shall meet quarterly or at any time if called by the board chair.

3/ All notifications of meetings of the Board shall include the meeting agenda, place and time. Unless the Board agrees otherwise and decide as per sub-article 6 of this article, all sessions of the Board shall be held in the Ministry.

4/ At any meeting of the Board, the presence of at least six members of the board shall constitute a quorum

5/ All members of the board who represent government organs shall be Minister or higher official.

6/ Members of the Board may participate in any of the meetings of the Board in person. However, it is also to attend meetings by using suitable communications technology enabling distance communication in real time. When the board members are unable to to participate the meeting of the board in person for sufficient reasons, they have the duty to notify the board chair three days before the date of the meeting.

7/ Without prejudice to sub-aricle 6 of this article the Board members should appear in person to adopt decisions for approving a project to proceed to tender in accordance with Article 18 of the Proclamation. However, if at least one Board meeting has been conducted in person and the Board had the opportunity during the Board meeting to discuss the project with the Contracting Authority, members may participate by using suitable electronic communications.

8/ Each Board member shall have one vote.

9/ Decisions of the Board shall be adopted by a simple majority of the members of the Board present at the relevant meeting or in the case of per rollam voting, by a simple majority of all Board members.

10/ Should a decision come before the Board in which a Board member has any potential or perceived conflict of interest, the Board member in question shall be responsible for notifying the Chair of such conflict and shall not participate in the vote.

11/ Any person attending a Board meeting shall keep confidential all information marked as confidential.

7. PPP Directorate General

1/ In providing technical assistance and support to Contracting Authorities, as per article 12 sub-article of PPP proclamation, the PPP Directorate General shall: -

a. retains, in conjunction with the Contracting Authority, transaction advisors as required in accordance with the decision to identify a project as a PPP by the Board;

b. nominates a staff member of the PPP Directorate General Representative, to participate actively in the Project Management Team throughout the project development and implementation process;

c. ensure that the PPP Directorate General Representative has access to the necessary specialist resources within the PPP Directorate General as required to fulfill his/her obligations to the Contracting Authority.

d. assist the Project Management Team in preparing the tender documentation and Project Agreements with transaction advisors and ensure that these documents are of appropriate quality;

e. ensures that the transaction advisors retained report both to the PPP Directorate General and the Contracting Authority;

f. ensure that, once a PPP project has been awarded, the Project Management Team understands the Project Agreements and provide the support necessary to assist the Project Management Team in implementing the Project Agreements.

2/ When selecting a Private Party on behalf of the Contracting Authority, the PPP Directorate General shall:

a. rely on the Project Agreements and tender documents prepared by the transaction advisors and the Project Management Team, as may be amended to ensure quality, as a basis for any selection process;

b. ensures that it reasonably takes into account the needs and concerns of the Contracting Authority at all stages of the selection process;

c. integrates the contracting authority in the discussions, meetings, exchanges within the PPP Directorate General and with the Ministry, advisors, bidders and other parties;

d. ensure that any modifications to tender documentation arising as a result of communications with bidders are discussed with the Project Management Team, and that Project Management Team comments are taken into account before the issuance of modified documentation;

8. Guidelines

1/ The PPP Directorate General shall issue guidelines designed to foster the development of Public Private Partnerships covering topics deemed appropriate by the PPP Directorate General including at least:

a. Identification and selection of potential PPP projects;

b. Project Feasibility screening and appraisal manual፣

c. Value for Money;

d. Requirements for the request and use of Government Support;

e. Model Project Agreements;

f. PPP contract management practices;

2/ The PPP Directorate General shall update the Guidelines on a periodic basis to take into account the evolution of the development of PPP projects in Ethiopia;

3/ The PPP Directorate General shall make the Guidelines publicly available.

CHAPTER THREE

DUTIES AND RESPONSIBILITIES OF CONTRACTING AUTHORITIES

9. Contracting Authority

1. Following the approval by the Board of the implementation of a project through a PPP, in accordance with Article 16 of the Proclamation, the Contracting Authority shall establish the Project Management Team. In establishing the Project Management Team, the Contracting Authority shall:

   a. Request the PPP Directorate General to avail aasistance and nominate a representative (the PPP Directorate General Representative) to serve on the Project Management Team;

   b. Choose from among its personnel the persons with the requisite financial, technical and legal skills it considers necessary to handle the management and implementation of the PPP project;

   c. Nominate a Team leader, from the Contracting Authority personnel assigned to the Project Management Team, who is expected to be the main focal point for leading the work of the Project Management Team and who may take project development decisions on behalf of the Contracting Authority;

   d. Consider whether additional financial, technical and legal skills are necessary in order to be able to appropriately supervise transaction advisors, and if deemed necessary or if mandated by the Board or PPP Directorate General, retain such additional skills.

2. In establishing the Project Management Team, the Contracting Authority may include representatives of other key stakeholders as may be appropriate to facilitate the implementation of the Project. These representatives are not required to be permanent members of the Project

Management Team and may be conveyed only for meetings where their particular expertise will be required.

3. The Contracting Authority shall at all times be responsible for ensuring that the Project Management Team fulfills its responsibilities under the Proclamation.

4. Prior to the signing of Project Agreement forwarded by PPP Directorate General, the Contracting Authority is responsible for ensuring that all approvals of Public Authorities required for the signature by the Contracting Authority of any Project Agreements have been obtained.

10. Project Management Team

1/ The Project Management Team shall hold meetings as required, to discuss the development of the feasibility and other studies and the development of the project structure, Project Agreements and tender documentation during the preparation of the PPP Project in addition to the the discussion to be held on the existing project implementation.

2/ Though it is supposed to be determined by the schedule of the Project Management Team, the Project Management Team shall the following: -

a. The team leader should notify all members of the Project Management Team sufficiently in advance of any meeting to enable their attendance.

b. All members of the Project Management Team are expected to use their best efforts to regularly attend such meetings. Notify the team leader on time if unable to attend meeting for justifiable reason.

c. Members of the Project Management Team who have a potential or a perceived conflict of interest or become aware of such conflict at any time during the development or implementation of the project, are required to inform the team leader.

3/ The Project Management Team shall produce tender documentation and Project Agreements with the support of the PPP Directorate General and transaction advisors.

4/ The Project Management Team shall review all documentation associated with the preparation of the project, whether produced internally or by transaction advisors and submit to the Contracting Authority or PPP Directorate General. Team members are expected to actively participate in Project Management Team discussions, including through the provision of comments on areas of the project documentation within their respective area of expertise.

5/ Project Management Team members are expected to keep all information provided to them confidential.

6/ In addition to those responsibilities listed in the Proclamation, the Project Management Team will also be responsible to develop a Project Management Checklist that helps to monitor if the project is going as planned and if both the Contracting Authority and the Private Party are performing their obligations as per the project agreement and submit the document to be approved by PPP Directorate General. The report which is prepared as per the Project Management Checklist shall be submitted to PPP Directorate General and Contracting Authority.

7/ After signing of the Project Agreement, if the Contracting Authority envisages the renegotiation of any Project Agreement, the Project Management Team shall inform the PPP Directorate General of the expected need to renegotiate and likely consequences. To the extent that the signing of an amended PPP Agreement will require Board approval as contemplated in Article 9 sub article 1 (c) of the Proclamation, the PPP Directorate General shall take steps to ensure such Board approval.

CHAPTER FOUR

PUBLIC PRIVATE PARTNERSHIP PROJECT DEVELOPMENT AND APPROVAL

11. Project Identification

1. As part of its normal infrastructure or public services planning process, the Contracting Authority or a Public Entity or PPP Directorate General may identify a Public Service Activity that may be carried out as a PPP.
2. The PPP Directorate General shall keep a PPP Project Pipeline which shall include a list of projects that have been identified as potential Public Private Partnership projects by the PPP Directorate General, Contracting Authorities or other Public Entity and shall periodically update this list.
3. The PPP Directorate General shall determine which projects included on the PPP Project Pipeline should be recommended to the Board for approval based on the likelihood of the projects that meet the criteria. The PPP Directorate General shall take into account policy guidance from the Board in making such determination.

12. Pre-Approval of the Board

1. The Board shall consider the following in approving any project for implementation as a PPP in accordance with Article 16 of the Proclamation:

    a. Whether the project is consistent with Ethiopia’s development objectives;

b. Whether there is sufficient demand for the project outputs;

c. Whether the project will gives substantial benefit to the public, if pivate sector is preferred to provide the service and if the project is expected to be able to transfer meaningful and appropriate risks to a private sector partner;

d. Whether a project is likely to deliver Value for Money;

e. Whether sufficient financial resources, including those required for expected Government Support, are expected to be available for the project; and,

f. Whether the Contracting Authority has sufficient capacity and resources to appropriately prepare and implement the project.

2. When approving a project for implementation as a PPP, the Board may include stipulations related to the preparation of the project including, but not limited to:

a. Conditions which the Project Management Team must adhere to in the preparation of the project as a PPP;

b. Staffing and expertise requirements for the Project Management Team.

13. Feasibility Study

1. The Project Management Team shall ensure that it prepares the feasibility study in accordance with Guidelines to be issued by the PPP Directorate General.
2. In addition to the topics listed in Article 17 sub-article (2) of the Proclamation, the Project Management Team shall also evaluate the options for local private sector participation in any project.
3. Based on the results of the feasibility study, the PPP Directorate General, in coordination with the Ministry and the National Bank of Ethiopia, shall prepare an analysis of the proposed commitments under the PPP project and highlight potential constraints associated with any financial commitments.
4. The feasibility study which is prepared by the Contracting Authority shall be submitted to the board for approval after being evaluated by PPP Directorate General.

14. Approval of the Board to Proceed to Tender

1. In addition to the submission of a feasibility study for approval by the Board pursuant to Article 18 of the Proclamation, the Contracting Authority should also submit:

a. A procurement plan for the tender of the project, including an outline of the proposed tender process, and heads of terms for Project Agreements and conditions or draft Project Agreements.

b. a list of required approvals from Public Entities that would be necessary prior to signing of the Project Agreements as proposed.

c. To the extent that approvals required from Public Enities can be obtained at the feasibility stage, the Contracting Authority should do so prior to seeking Board approval.

2. The PPP Directorate General shall evaluate the feasibility study submitted by the Contracting Authority and ensure that the following points are included in the pinion to be submitted for the board decision: -

a/ Explain the fact that the feasibility study is accompanied with relevant documents and proof that the project can be implemented as PPP.

b/ As per the specific project, state the appropriate mode of private party selection or put evidence-based selection plan;

c/ Major criteria to select private party and when appropriate including project agreements terms;

3. The Contracting Authority shall have the right to defend the project to the Board at the meeting where approval is being considered and may respond to any questions directed to it by the Board when considering its approval.

4. To the extent that the PPP Directorate General, is authorized to proceed to tender, it may not launch the tender without having obtained approvals of the incentives or Government support that the Board has submitted to other authorities in accordance with Article 18 sub-article 4 of the Proclamation.

5. In the event of a material change to the project structure either during tendering or as a result of renegotiation after the signing of contracts, the PPP Directorate General shall prepare an appropriate analysis of the impact of proposed material changes to the project, by involving the Project Management Team of the Contracting Authority and submit this to the Board for decision.

15. Government Support

In accordance with Article 47 of the Proclamation and to the extent Government support is required in the Project Agreements, and, where there is no express requirement for approval by

an authority of such Government Support otherwise prescribed by Law, then Board approval of the project pursuant to Articles 18 or 32 of the Proclamation shall be deemed satisfactory approval of such Government support.

CHAPTER FIVE

SELECTION OF PRIVATE PARTY

16. Requalification

1/ Request for qualifications shall be published at least once in newspaper that has wide circulation to ensure participation of as many bidders as possible. The PPP Directorate General shall use reasonable efforts to publish the same information on it's, the Ministry and/or the Contracting Authority's websites.

2/ The PPP Directorate General may in addition to the newspapers publications; send the request for qualifications to potential bidders.

3/ The content of the potential requests for qualifications shall at least include the following:

a. general information related to the project;

b. an indication of other essential elements of the project, such as the services to be delivered and the financial arrangements envisaged;

c. where already known, a summary of the main required terms of the project agreements;

d. the prequalification criteria; and

e. the manner and the deadline for submitting the qualifications.

17. Prequalification criteria

1/ The prequalification criteria stated in the request for qualifications shall include at least the following:

a. Adequate professional and technical qualifications as necessary to carry out all the phases of the project;

b. Sufficient ability to manage the financial aspects of the project and capability to sustain its financing requirements; and

c. Appropriate managerial and organizational capability, reliability and experience, including previous experience in operating similar infrastructure facilities.

2/ Such qualifications should be evidenced through:

a. the demonstration of previous experience, such as the undertaking of projects similar in nature technically, financially or managerially;

b. with respect to financial capacity, net worth, net turnover or other financial criteria as may be deemed appropriate, the submission of audited financial statements or other appropriate financial documents;

c. other mechanisms as are deemed appropriate by the PPP Directorate General.

3/ Appropriate prequalification criteria for typical types of PPP projects will be provided in Guidelines to be issued by the PPP Directorate General.

18. Request for proposals

1/ In addition to the details listed in the Proclamation, the request for proposals shall:

a. Clearly lay out the procurement process that will be followed;

b. If applicable, the process for bidders to provide comments on the terms of the request for proposals and the Project Agreements; and,

c. The process and timing for bidders to raise questions.

d/ Clearly set the criteria that the bidders need to fulfill;

f/ Ensure the clear technical and financial proposal

g/ State the date and time to submit the proposal;

h/ Put the address where bidders could get clarification and information;

i/ Mention the means of submitting technical and financial proposal and the evaluation procedure as well.

19. Bid Securities

1/ The PPP Directorate General must require that bidders submit a bid security.

2/ The PPP Directorate General shall ensure that the nature, form, amount and other principal terms requested for the bid security are within market practice.

3/ The amount of the bid security should be set in a manner that incentivizes bidders to comply with their undertakings but shall not be so high as to deter bidders from participating or unduly increase bid preparation costs.

4/ A bidder shall not forfeit any bid security that it may have been required to provide, other than in cases of:

a. Withdrawal or modification of a proposal after the deadline for submission of proposals;

b. Failure to enter into final negotiations with the PPP Directorate General pursuant to Article 31 of the Proclamation;

c. Failure to sign the Public Private Partnership agreement, if required by the PPP Directorate General to do so, after the proposal has been accepted; or,

d. Failure to provide required security for the fulfillment of the Project Agreements after the proposal has been accepted or to comply with any other condition prior to signing the Public Agreements specified in the request for proposals.

20. Evaluation of Proposals

1/ The criteria for the evaluation and comparison of the technical proposals shall include at least the following:

a. Technical soundness;

b. Compliance with environmental standards;

c. Operational feasibility;

d. Quality of services and measures to ensure their continuity.

2/ The criteria for the evaluation and comparison of the financial proposals shall depend on the project. The PPP Directorate General shall issue guidelines in this regard.

3/ The request for proposals may provide that a preference margin of up to 7% of bonus may be allocated to the financial proposal provided by the bidders when the capital share of the domestic party is more than 50% and the following conditions are fulfilled:

a. for a project involving a construction phase, if at least 30% of the raw materials used are from Ethiopia and 50% of the employees are Ethiopians;

b. when 50% of Ethiopians are involved in the project/company management;

b. for a project that doesn't involve a construction phase, the request for proposals shall describe the thresholds that will determine if a bid can benefit from a preference margin.

4/ Bidding consortia with foreign bidders may benefit from a preference margin if the conditions provided in Sub-Articles 2 and 3 are met.

21. Notification of Award and Signing of Contract

1/ The Contracting Authority shall not sign a Project Agreement before seven days from the date bidders are notified of the result of their bid to enable unsuccessful bidders to submit a complaint in accordance with Article 30 of the Directive.

2/ Upon signing of a Public Private Partnership Agreement by the parties, the PPP Directorate General shall publish the results of the tender in a national newspaper of wide circulation indicating the purpose of the contract, its duration, the name of the parties, the fee structure and the main characteristics of this contract. The PPP Directorate General shall use reasonable efforts to publish the same information on its and the Ministry's website.

3/ The PPP Directorate General and the Contracting Authority shall keep an appropriate record of information pertaining to the selection and award proceedings.

CHAPTER SIX

COMPETITIVE DIALOGUE

22. Conditions of use of Competitive Dialogue

For the purpose of recourse to the competitive dialogue mentioned in Article 37 sub-article 1 of the Proclamation, a project is considered to be 'particularly complex' where the Contracting Authorities and the PPP Directorate General:

a. are not objectively able to define the technical means capable of satisfying their needs or objectives, and/or

b. are not objectively able to specify the legal and/or financial make up of a project.

CHAPTER SEVEN

DIRECT NEGOTIATION

23. Conditions for use of Direct Negotiations

1/ In accordance with Article 39 sub-article1 (b) and (f) of the Proclamation, the PPP Directorate General may engage in direct negotiation in respect of a Public Private Partnership, upon approval by the Board, only where the following conditions are satisfied:

a. Where a project has duration of five years maximum and the anticipated initial investment value does not exceed one hundred forty million Ethiopian birr;

b. Where a project is ancillary and the necessary complement of, industrial or other type of investment, must be carried out by the private sponsor of such industrial or other type of investment and it is very unlikely that other bidders would be interested by pursuing such a project or would be in a position to offer better terms and conditions. The PPP Directorate General shall provide evidence to the Board that it has requested letters of interest, quotes or other forms of interest from potential bidders and an analysis of the responses received. The PPP Directorate General shall have contacted at least three reputable international sponsors who have conducted similar projects.

CHAPTER EIGHT

UNSOLICITED PROPOSALS

24. Preliminary Agreement with Proponent of an Unsolicited Proposal

1/ If the Unsolicited Proposal is accepted by the PPP Directorate General in accordance with Article 43 sub-article 1 of the Proclamation, the proponent and the Contracting Authority shall enter in an agreement with the support of the PPP Directorate General prior to submission of the project to the Board. This agreement shall at least provide for:

a. Confidentiality obligations for both parties relating to the content of the studies prepared by the proponent;

b. The terms and conditions for the transfer of all studies and rights over the project to the Contracting Authority or another Person should the proponent not be awarded the project;

c. The allocation of bonus points during the tender process or the allocation of a financial compensation for studies undertaken by the proponent should the proponent not be awarded the project in accordance with Article 24; and

d. The next steps for the development of the project and if appropriate the allocation of responsibilities to carry out these steps.

2/ The PPP Directorate General shall establish a template agreement for the purpose of Sub-Article 1/.

25. Allocation of Bonus and Financial Compensation

1/ The identity of the proponent, the detailed allocation of bonus points or the amount of the potential compensation to the proponent shall be indicated in the competitive selection procedure documents, preferably at the prequalification stage, if applicable.

2/ Given potential difficulties in allocating fairly bonus technical or financial points, the PPP Directorate General should be privileged with the power of allocation of a financial compensation paid to the proponent and bonus points should the proponent not be awarded the project.

3/ such financial compensation to the proponent should be paid by the successful bidder who is awarded the project as a condition precedent to financial close and commencement of the works and/or services. This obligation should be mentioned at the request for proposal.

CHAPTER NINE

CONTENT AND IMPLEMENTATION OF THE PUBLIC PRIVATE PARTNERSHIP AGREEMENT

26. Duration

1/ Subject to the type and nature of PPP projects and as per article 45 of PPP proclamation, duration of PPP projects shall be twenty to thirty years.

2/ Notwithstanding Sub-article (1) of this article, project agreement for duration of less than twenty years can be entered when the special nature of the project demands,

3/ When determining the duration of Project Agreements, the PPP Directorate General shall take into consideration the useful life of the assets of the project, the availability of financing and standard market practice for similar projects.

27. Transfer of Project Assets

The Project Agreements may provide that upon early termination or expiration of the Project Agreements, the assets of the project are transferred to the Contracting Authority or another Public Entity. In this event, the Project Agreements shall provide the manner in which the Contracting Authority will ensure that the assets are transferred in good condition subject to normal wear and tear.

28. Termination of the Project Agreement

The Project Agreements shall stipulate events that may or shall trigger the termination of the Project Agreements, including, as appropriate, a material breach of the Project Agreements, the occurrence of the force majeure event, a payment default or any other event as may be stipulated in the Project Agreements.

Chapter Ten

Rules of Ethics of Public Private Partnership

29. Ethics

1/ Persons engaged in Public Private Partnership process shall have to observe the following rules of ethics in the discharge of their duties:

a/ the obligations to notify any actual or possible conflict of interest and isolate oneself from any processes involving such conflict;

b/ to give candidates and project implementer equal opportunity of competition and performance;

c/ keep in secret any confidential information concerning the PPP project, candidate project implementer which he/she came to know on account of his/her duty;

d/ to reject any gift or offer of an employment opportunity or anything of monetary value or service;

e/ to report to the law enforcement agencies any intended or completed action of corruption and contribute to the effort to fight corruption and malpractice;

f/ demonstrate concern to public resource and property.

2/ Any private party shall have to refrain from any act contravening the process of PPP private party selection. Any private party shall have the responsibility:

a./ with an intention to influence the decision or action of the head or staff member of the public body or persuade the public body to change its established practice of private party selection, not to give directly or indirectly gifts of any kind in the form of inducement, not to promise to give gifts, not to offer employment opportunity or anything of monetary value or service;

b/ with an intention to mislead a procurement proceeding not to present a falsified document or not to withhold information he/she should have disclosed;

c/ not to connive with another candidate in an act of false competition in order to get unfair advantages;

d/ to report to the law enforcement agencies any intended or completed action of corruption which he came to know during the procurement proceedings or private party selection and contribute to the effort to fight corruption and malpractice.

CHAPTER ELEVEN

MISCELLANEOUS

30. Complaints

1/ A candidate private party shall be entitled to submit a complaint to PPP Directorate General or to the Board against an act or omission of the public body in regard to PPP selection proceeding where he believes that such an act or omission violates PPP laws.

2/ No complaint may be lodged in accordance with sub- article (1) of this Article in respect of the following matters:

a) private party selection method pursuant to PPP Proclamation;

b) the rejection of bids, proposals or quotations.

3/ Complaints against an act or omission of PPP Directorate General or Contracting Authority pertaining to a proceeding leading to an award after the contract has been signed with the successful bidder may not be brought before PPP Directorate General or Contracting Authority or to the Board.

4/ The provision of sub- article (3) of this Article shall apply where the following conditions are satisfied:

a. Where the contract has been signed without a complaint being filed within the time limit prescribed in this directive;

b. Where the PPP Directorate General responds to the complaint lodged and a contract is signed because of the expiration of the time limit for the signing of contract after the award without the candidate pursuing its complaint further.

5/ The procedure to be followed in resolving complaints brought with regard to private party selection proceeding shall not strictly apply the provisions of civil procedure code of Ethiopia, rules of complaint hearing. It will be flexible.

31. Review of Complaints by the PPP Directorate General

1/ A complaint against an act or omission by PPP Directorate General or Contracting Authority shall in the first instance, be submitted to the PPP Directorate General.

2/ A private party shall have to submit the complaint within five working days from the date he knew or should have known the circumstances giving rise to the complaint.

3/ Unless the complaint is resolved by mutual agreement, the PPP Directorate General shall suspend private party selection proceedings and shall, within 10 days after submission of the complaint, issue a written decision, stating the reasons, and, if the complaint is upheld, indicating the corrective measures to be taken.

4/ If the PPP Directorate General does not issue a decision within the time stated in sub-article (3) of this Article, or if the private party is not satisfied with the decision of PPP Directorate General the private party is entitled to submit a complaint to the Board within five working days from the date on which the decision has been or should have been communicated to the private party.

32. Reviews by the Board

1/ Upon receipt of a complaint, the Board shall promptly give notice of the complaint to PPP Directorate General and/or Contracting Authority. Such action automatically suspends further action by the PPP Directorate General or Contracting Authority until the Board has settled the matter.

2/ The Board, unless it dismisses the complaint may:

a. prohibit PPP Directorate General or Contracting Authority from acting or deciding unlawfully;

b. order the PPP Directorate General or Contracting Authority to proceed performing their works, except the signing of project agreement and award related decisions;

c. annul in whole or in part, an unlawful act or decision by the PPP Directorate General or Contracting Authority.

3/ The Board shall, before taking any decision regarding a complaint, notify relevant bodies of the complaint and shall take into account information and arguments received from such bodies.

4/ The Board shall issue its decision within twenty-one (21) working days of receiving the complaint, stating the reasons for its decision and remedies granted, if any.

33. Complaint Instituted by PPP Directorate General or Contracting Authority

1/ Where a PPP Directorate General or Contracting Authority believes that an unlawful act or an act prejudicial to its legitimate interest has been committed by a candidate or project implementer, it shall without prejudice to the measures it is entitled to take against such person in accordance with the bidding document or the contract, notify the matter in writing to the board.

2/ Upon receipt of the complaint the board shall send a written notice of the complaint and the content of such complaint to the candidate or project implementer, against whom the complaint has been lodged.

3/ Where the board finds it appropriate to the resolution of the complaint it may require persons concerned with the matter to appear in person and give evidence or seek professional assistance from any appropriate body.

4/ The board shall review and give decision on the complaint within twenty-one (21) working days of receipt of such complaint.

5/ The decision to be rendered by the board in accordance with sub-article (4) of this Article may be one of the followings:

a. suspend the candidate or project implementerfor a definite or indefinite period, which it finds to be at fault from participation in any public private partnership bids or projects;

b. give a written warning;

c. dismiss the complaint.

34. Power to Issue Directive

When deemed appropriate this directive will be repealed or amended by the Ministry.

35. Effective Date

This directive shall enter into force on July 19, 2018. The date it is signed by the Minister.

Abraham Tekeste (PhD)

Ministry of Finance and Economic Development, Minister

Date: July 19, 2018

Addis Ababa
