# Supplementary material for: Range of hurdles and opportunities for developing PPPs in diagnostics: a contextual analysis, Ethiopia
Source: BMC Health Serv Res. 2026 Mar 28;26:651. doi: 10.1186/s12913-026-14354-z (PMC13151300; doi:10.1186/s12913-026-14354-z)
Supplement: Supplementary file 5 — Supplementary Material 5 [file 12913_2026_14354_MOESM5_ESM.docx]

African Health Diagnostics Platform Multi-Country Evaluation Process Evaluation – PPP Procurement

Key Informant Interview Guide – V1 / 7 June 2021

NOTE: Key informant interview (KII) questions are derived from the process evaluation questions and sub-questions developed to-date. This guide is meant to be comprehensive but generic. The KII guide will need to be adapted to country contexts, and respondent-specific versions will likely be needed (see below). Two rounds of KIIs are planned to capture how the PPP process progresses in each country. **This guide is focused on PPP tender and procurement.**

# **Introduction**

**INTEVIEWER:** Thank you for taking the time to meet with me today. I want to talk you again about the public-private partnership (PPP) being developed under the African Health Diagnostics Platform (AHDP) to improve the access and quality of laboratory services in sub-Saharan Africa. Our team is conducting an evaluation to describe the design and implementation of the PPP, assess the impact of the PPP on delivery of laboratory diagnostics in-country, and generate lessons for the future. During today’s interview, I am going to focus on asking questions related to the tender and procurement process of the PPP.

Let’s start!

1. IF REPEAT RESPONDENT 🡪 Has your involvement in the PPP process under the AHDP changed since we last spoke? If so, how has it changed?
   1. IF NEW RESPONDENT 🡪 Please describe your involvement in the PPP process under the AHDP.

# **PPP tender/procurement**

**INTERVIEWER**: First, I want to ask you some questions about how the specifics of the PPP procurement were negotiated and managed, and whether private partners have shown interest in the PPP.

## Specifics of the PPP procurement negotiation

1. Who has been involved in the tender and procurement process of the PPP?

PROBE: CHAI, national gov’t, sub-national gov’t, MOH, MOF, facilities participating in AHDP, private partners, beneficiaries/civil society, etc.

- 1. Have there been any champions supporting the PPP?
  2. Have there been any actors trying to block the PPP?

1. Did negotiations take place as part of existing structures or did a new group/structure have to be established?
2. How has gender influenced PPP tender and procurement?

## Effectiveness and transparency of the PPP tender and procurement process

1. Who is responsible for managing the tender process and ensuring timely progress?
   1. How effectively are these actors managing the process?
   2. How could the tender process management be improved?
   3. Who is **not** currently involved in managing the tender process but should be?
2. How long did it take to move from agreement in the PPP design to approval and publication of the tender?
3. Is this a typical timeline for PPPs of this nature?
4. How closely has the tender process followed government guidelines on these types of procurements?
5. Have there been any difficulties in implementing the tender process?
6. Where there any efforts to push ahead with the tender process despite objections?
7. How transparent has the tender process been thus far?
8. How could the tender process be made more transparent?
9. How did the tender process affect the procurement/awarding process?
10. How did the tender and procurement process change the PPP design, if at all?

## Private partners engagement in the tender process

1. How interested have private partners been in participating in the PPP? [REPEAT FROM 1^ST^ ROUND]
   1. What are private partners’ main questions around participating in the PPP?
   2. What are the biggest incentives for private partners to participate in the PPP?
   3. What are the biggest drawbacks for private partners to participate in the PPP?
   4. Do you think that private partners working in laboratory diagnostics in-country have the capacity to participate and deliver on the PPP? Why/why not?
   5. Are you aware of the financial instruments that AHDP is offering? If so, what are they?
   6. How interested have private partners been in taking up the available financial instruments offered by the AHDP? Why?
      1. IF NOT INTERESTED 🡪 How could the financial instruments from AHDP be changed to incentivize private sector engagement in the PPP?
2. Of the eligible private partners, how many private partners bid on the tender?
3. How many private partners are actually able to meet the tender requirements?
   - 1. Which is the hardest tender requirement to meet?

# **Influence of context over PPP the tender and procurement process**

**INTERVIEWER**: The next series of questions are about how the policy context and different stakeholders shaped PPP design.

## Policy context and historical relationship influence over the PPP

1. How do the health system governance arrangements (e.g., decentralization, devolution) affect the PPP tender and procurement process?
   1. Who is accountable for the success of the PPP?
      1. To whom are they accountable?
2. How has COVID-19 affected the PPP tender and procurement process?

PROBE: timeline, stakeholder interest/bandwidth, changes to financial risks of the PPP, political risks to the PPP, etc.

1. How has the PPP tender procurement process adapted to COVID-19?
2. How has COVID-19 affected the viability of the PPP going forward?

## Actors affected by the PPP and their interests

1. Have there been any changes to the key stakeholders in the PPP and why are they involved? 🡪 USE SHOWCARD TO SHOW RESPONDENT LIST OF STAKEHOLDERS

PROBE: global, national, sub-national, facility, patients; private, public, technical assistance, funders, etc.

- 1. How did these changes in stakeholders emerge?
  2. How do they perceive the advantages and disadvantages of the PPP?
  3. What are their incentives to participate in the PPP?

1. What is the relative influence of these actors on each step of the tender and procurement process?
2. How is the role and relative influence of these actors changing over time?
3. How have people’s perceptions about the potential costs and benefits of pursuing a PPP for laboratory diagnostics changed?
4. How are AHDP and its partners addressing any emerging threats to the PPP’s success?
5. How has the structure of the AHDP project itself affect dynamics at the country level?
6. How have BMGF, CHAI and EIB influenced country-level PPP dynamics?
7. To what degree is there cross-country learning within the AHDP project?

# **Pathway to sustainability and scale up**

**INTERVIEWER**: The last set of questions are focused on sustainability and scale-up of services under the PPP, and how these factors have influenced the process.

## Scale-up and sustainability considerations’ influence on the PPP tender and procurement process

1. To what extent in the PPP process have scale-up and sustainability been part of the discussion around PPP procurement?

PROBE: influence on tender, procurement, implementation.

- 1. Which have been the key topics that have been discussed around scale-up and sustainability? E.g., geographic scale-up, expanding to other facilities, adding new diagnostics, etc.
     - Have these been resolved? If so, how?
     - Which topics are still unresolved? Why?

1. How are the plans in place to support future public sector financing of laboratory diagnostics evolving?
2. Has any changed since the start of the PPP process? If so, how?
3. Which different sources of funding are expected to be used for financing diagnostics?
4. How has government increased financing support to diagnostics during the PPP period (e.g., national health insurance)?
5. Are these financing plans sustainable? Why/why not?
6. How viable are the different sources of funding being considered? E.g. politically viable, practically viable, etc.
7. Is there anything else you would like to discuss about the PPP that we have not talked about?

THANK YOU FOR YOUR TIME
