# Supplementary material for: Range of hurdles and opportunities for developing PPPs in diagnostics: a contextual analysis, Ethiopia
Source: BMC Health Serv Res. 2026 Mar 28;26:651. doi: 10.1186/s12913-026-14354-z (PMC13151300; doi:10.1186/s12913-026-14354-z)
Supplement: Supplementary file 6 — Supplementary Material 6 [file 12913_2026_14354_MOESM6_ESM.docx]

African Health Diagnostics Platform Multi-Country Evaluation

Process Evaluation – PPP Design

Key Informant Interview Guide – V1 / 7 June 2021

NOTE: Key informant interview (KII) questions are derived from the process evaluation questions and sub-questions developed to-date. This guide is meant to be comprehensive but generic. The KII guide will need to be adapted to country contexts, and respondent-specific versions will likely be needed (see below). Two rounds of KIIs are planned to capture how the PPP process progresses in each country. **This guide is focused on PPP design and pre-tender process.**

# **Introduction**

**INTERVIEWER**: Thank you for taking the time to meet with me today. I want to talk about a new public-private partnership (PPP) being developed under the African Health Diagnostics Platform (AHDP) to improve the access and quality of laboratory services in sub-Saharan Africa. Our team is conducting an evaluation to describe the design and implementation of the PPP, assess the impact of the PPP on delivery of laboratory diagnostics in-country, and generate lessons for the future. During today’s interview, I am going to focus on asking questions related to the design of the PPP and the pre-tender process.

Let’s start!

1. Please describe your involvement in the PPP process under the AHDP.

# **PPP design**

**INTERVIEWER**: First, I want to ask you some questions about how the specifics of the PPP design were negotiated, and the different options that were considered during the design process.

## Specifics of the PPP design negotiation

1. What was the process of negotiation over PPP specifics?
   1. How does this process vary by topic? E.g., facility selection, procurement model/contract type, including referral network, etc.
2. What were the sticking points around the PPP design, and how were they resolved?
3. Did negotiations take place as part of existing structures or did a new group/structure have to be established?
4. Who has been involved in the design of the PPP?

PROBE: CHAI, national gov’t, sub-national gov’t, MOH, MOF, facilities participating in AHDP, private partners, beneficiaries/civil society, etc.

- 1. How did the actors involved in the design process change between the initial design and the final agreement?
     1. Why did they change?
     2. How did these changes affect the process?
  2. Have there been any champions supporting the PPP?
  3. Have there been any actors trying to block the PPP?

1. Who is/was ultimately responsible for approving the PPP design?

## Different options or factors considered during the PPP design process

1. What options were considered when designing the model?

PROBE: different contracting options, regulatory considerations, geographic spread/focus, referral network, etc.

- 1. Does the PPP design deviate from any existing regulatory norms? If so, why?
  2. How were public facilities/sites for the PPP selected?
  3. How does the PPP design distribute risks between government and the private sector?
  4. How does the PPP design address the risks identified in the feasibility reports?

1. How does key PPP stakeholder capacity affect PPP design, tender/procurement and implementation?

PROBES:

1. Government capacity to manage the PPP life cycle, incl. draft tender, bid review, procurement, monitoring and payment.
2. Private partner capacity to deliver on terms of the PPP
3. Public facility laboratory management capacity (e.g., work within PPP arrangement, for laboratory management, etc.)
4. Individual laboratory capacity: provider capacity for test ordering, laboratory capacity to conduct tests.
5. How appropriate is the design to the local context? (e.g., political, socio-economic, technological, legal, environmental, health system, diagnostics market)
6. How disruptive is the PPP to the current system and how much change will it require?
7. How does the PPP design accommodate the needs of the focus populations? E.g., poorest households, complicated patients (e.g., co-morbidities, prone to loss to follow up), etc.
   1. How has gender influenced the design of the PPP? E.g. different laboratory needs of men and women, etc.
8. How well does the PPP design address key considerations to improve access of laboratory diagnostics?

PROBE: availability, quality, efficiency and affordability

1. Which consideration is best served by the current design? Why?
2. Which consideration is currently receiving the least attention in the current design? Why?
3. What are the perceived costs and benefits of pursuing a PPP for laboratory diagnostics at this time?
4. AHDP is providing technical support and facilitating financing for this PPP, how did the availability of this support affect the PPP design?
5. What are current features of the PPP design that would not be possible without AHDP technical support?
6. What are current features of the PPP design that would not be possible without the financial instruments facilitated by AHDP?
7. How is AHDP’s technical support being used to address any gaps or weakness in capacity among key stakeholders?

# **Influence of context over PPP design and the tender process**

**INTERVIEWER**: The next series of questions are about how the policy context and different stakeholders shaped PPP design.

## Policy context and historical relationship influence over the PPP

1. How supportive is the existing policy, legal and regulatory framework for PPPs?
   1. How much resistance among policymakers will this PPP need to overcome to garner the necessary support?
   2. How flexible is the PPP to changes in government or government priorities?
2. How have public and private sectors for health related historically?
3. How trusting, collaborative is the relationship?
4. How has the historical relationship between public and private sectors for health influenced the current PPP arrangements?
5. What other, earlier experiences or projects have there been in-country that can provide lessons for AHDP? E.g., health PPPs, programs to improve laboratory or diagnostic services, etc.
6. How do the health system governance arrangements (e.g., decentralization, devolution) affect the PPP design and pre-tender process?
7. Who is accountable for the success of the PPP?
   - 1. To whom are they accountable?
8. What has been the historic investment and interest from government in laboratory diagnostics?
9. How does improvement in laboratory diagnostics align with government priorities for the health sector?
10. What is profile of health workers relative to the diagnostic system, incl. training, cadres, unionization, etc.?
11. How has COVID-19 affected the PPP design and pre-tender process?

PROBE: timeline, stakeholder interest/bandwidth, changes to financial risks of the PPP, political risks to the PPP, etc.

1. How has the PPP design process adapted to COVID-19?
2. How has COVID-19 affected the viability of the PPP going forward?

## Actors affected by the PPP and their interests

1. Who are key stakeholders in the PPP and why are they involved? 🡪 USE SHOWCARD TO SHOW RESPONDENT POTENTIAL LIST OF STAKEHOLDERS

PROBE: global, national, sub-national, facility, patients; private, public, technical assistance, funders, etc.

- 1. How do they perceive the advantages and disadvantages of the PPP?
  2. What are their incentives to participate in the PPP?
  3. How threatening to the success of the PPP is the potential for turnover in key positions among national actors?

1. What is the relative influence of these actors on the design and pre-tender process?
2. How is the role and relative influence of these actors changing over time?
3. Whose incentives are served or undermined by laboratory diagnostics happening in the public vs. the private sector?
4. Who benefits the most from a joint public-private venture in laboratory diagnostics?
5. Who is potentially threatened by the PPP arrangement and why?
   - 1. How are AHDP and its partners seeking to address those threats?
6. How does the structure of the AHDP project itself affect dynamics at the country level?
7. How have BMGF, CHAI and EIB influenced country-level PPP dynamics?
8. To what degree is there cross-country learning within the AHDP project?
9. How interested are private partners in participating in the PPP?
   1. What are private partners’ main questions around participating in the PPP?
   2. What are the biggest incentives for private partners to participate in the PPP?
   3. What are the biggest drawbacks for private partners to participate in the PPP?
   4. Do you think that private partners working in laboratory diagnostics in-country have the capacity to participate and deliver on the PPP? Why/why not?
   5. Are you aware of the financial instruments that AHDP is offering? If so, what are they?
      1. If so, are these financial instruments from AHDP sufficient to incentivize private sector engagement in the PPP? Why/why not?
      2. Are private partners interested in taking up the available financial instruments offered by the AHDP? Why/why not?

# **Pathway to sustainability and scale up**

**INTERVIEWER**: The last set of questions are focused on sustainability and scale-up of services under the PPP, and how these factors have influenced the process.

## Scale-up and sustainability considerations’ influence on the PPP design and pre-tender process

1. To what extent in the PPP process have scale-up and medium-to-long-term sustainability been part of the discussion around PPP design?

PROBE: influence on design and pre-tender.

- 1. Which have been the key topics that have been discussed around scale-up and sustainability? E.g., geographic scale-up, expanding to other facilities, adding new diagnostics, etc.
     - Have these been resolved? If so, how?
     - Which topics are still unresolved? Why?

1. What are the plans in place to support public sector financing of diagnostics in the future?
2. Which different sources of funding are expected to be used for financing diagnostics?
3. How has government increased financing support to diagnostics during the PPP period (e.g., national health insurance)?
4. Are these financing plans sustainable? Why/why not?
5. How viable are the different sources of funding being considered? E.g. politically viable, practically viable, etc.
6. Is there anything else you would like to discuss about the PPP that we have not talked about?

THANK YOU FOR YOUR TIME
